# Friedel–Crafts Type Methylation with Dimethylhalonium Salts

**DOI:** 10.1002/chem.202001457

**Published:** 2020-10-05

**Authors:** Sebastian Hämmerling, Patrick Voßnacker, Simon Steinhauer, Helmut Beckers, Sebastian Riedel

**Affiliations:** ^1^ Institut für Chemie und Biochemie Freie Universität Berlin Fabeckstr.34/36 14195 Berlin Germany

**Keywords:** electrophilic substitution, halonium ions, methylation, reaction mechanism, weakly coordinating anion

## Abstract

The dimethylchloronium salt [Me_2_Cl][Al(OTeF_5_)_4_] is used to methylate electron‐deficient aromatic systems in Friedel–Crafts type reactions as shown by the synthesis of *N*‐methylated cations, such as [MeNC_5_F_5_]^+^, [MeNC_5_F_4_I]^+^, and [MeN_3_C_3_F_3_]^+^. To gain a better understanding of such fundamental Friedel–Crafts reactions, the role of the dimethylchloronium cation has been evaluated by quantum‐chemical calculations.

## Graphical abstract

**For the peace of it**: The dimethylchloronium salt [Me_2_Cl][Al(OTeF_5_)_4_] is used to methylate electron‐deficient aromatic systems in Friedel–Crafts type reactions. The role of the dimethylchloronium cation in a classical Friedel–Crafts reaction is evaluated by quantum‐chemical calculations. Furthermore, the *N*‐methylated cations [MeNC_5_F_5_]^+^, [MeNC_5_F_4_I]^+^ and [MeN_3_C_3_F_3_]^+^ are presented.

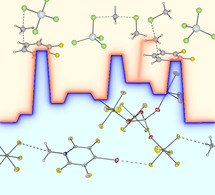

## Introduction

**Scheme 1:**
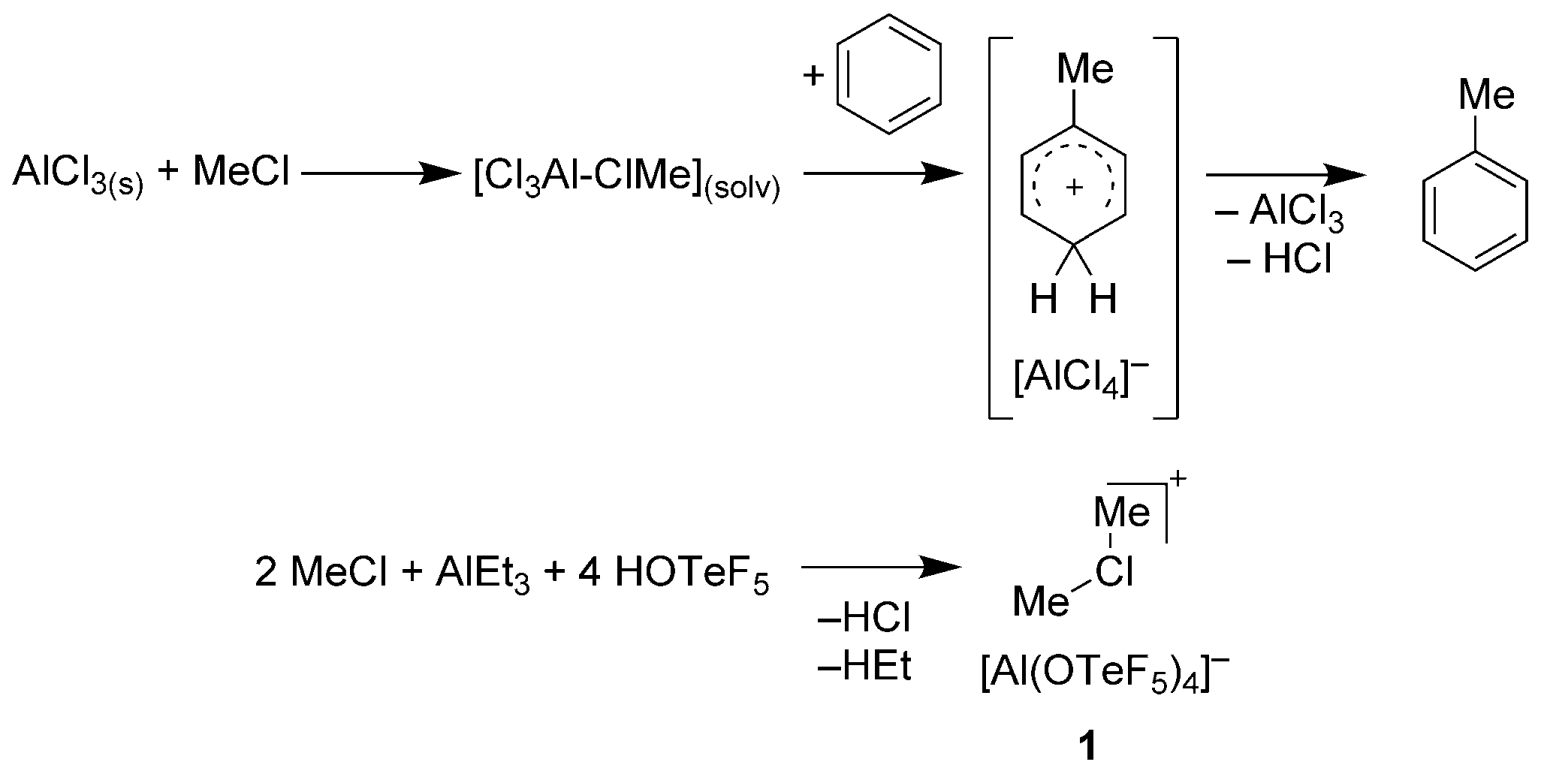
Friedel–Crafts alkylation of benzene with [Cl_3_Al–ClMe] (top) and formation of the dimethylchloronium salt [Me_2_Cl][Al(OTeF_5_)_4_] (**1**, bottom).

The Friedel–Crafts alkylation is a well‐known and widely used but also challenging synthetic tool.^[1]^ The Friedel–Crafts alkylation reaction is based on the polarization of an alkyl halide by a Lewis acid like AlCl_3_ which further reacts as an electrophilic reagent for example, with an aromatic molecule to form an arenium ion (Wheland intermediate, Scheme 1, top).^[2]^ Rearomatization of the Wheland intermediate occurs through elimination of HCl and recovers the catalyst. Depending on the stabilizing effects of the alkyl group the alkylating species has a distinct carbocationic character. The tendency to form a free CH_3_^+^ cation^[3]^ is low, therefore Olah and DeMember suggested the formation of the dimethylchloronium cation as an intermediate for Friedel–Crafts reactions^[4]^ and isolated [Me_2_Cl][SbF_6_]^[5]^ as a thermally labile compound. The only other known anion which is able to stabilize the dimethylchloronium cation so far is the carborate anion [CHB_11_Cl_11_]^−^. The salt [Me_2_Cl][CHB_11_Cl_11_] is considerably more thermally stable.^[6]^ We recently synthesized the easily accessible and room temperature‐stable dimethylchloronium salt [Me_2_Cl][Al(OTeF_5_)_4_] (**1**, see Scheme 1 bottom).^[7]^

In this work we report on the role of the dimethylchloronium cation in Friedel–Crafts type methylation reactions, especially in the system MeCl–AlCl_3_; and the reaction of **1** with electron‐deficient aromatic systems.

## Results and Discussion

### Role of the dimethylchloronium cation in Friedel–Crafts type methylation reactions

The electrophilic intermediate in Friedel–Crafts type methylation reactions is still controversial. While it seems well established that this alkylation proceeds via an Lewis‐acid activated alkyl halide such as the [Cl_3_Al–ClMe] intermediate shown in Scheme 1, the role of dialkylhalonium intermediates in this process and the relationship between these two intermediates has to our knowledge not been fully elucidated. Previous investigations on the mechanism only considered the direct methylation of the aromatic compound by the Lewis acid‐alkyl halide complex.^[8]^

We have measured ^27^Al and ^1^H NMR spectra of a slurry of aluminum trichloride in chloromethane. The solubility of aluminum trichloride at room temperature in pressurized chloromethane is low. However, a broad resonance at *δ*=108.6 ppm (FWHM=357 Hz) can be detected in the ^27^Al NMR spectrum, which we assign to a [Cl_3_Al–ClMe] complex. The signal is broadened by quadrupolar interactions and is in a typical range for donor stabilized tetrahedral coordinated AlCl_3_.^[9]^ This chemical shift is comparable with that of the [AlCl_4_]^−^ anion at *δ*=104.2 ppm, which shows however a significantly lower linewidth of 3 Hz. The ^1^H NMR spectrum of this solution shows only the signal of MeCl at *δ*=3.35 ppm. The addition of 1,2‐difluorobenzene to this [Cl_3_Al–ClMe]/MeCl mixture results in a slow methylation of the aromatic compound within days at room temperature. For comparison, a solution of [Me_2_Cl][Al(OTeF_5_)_4_] (**1**) in chloromethane revealed a distinct signal of the cation at *δ*=4.75 ppm in the ^1^H NMR spectrum. The cross signals in an EXSY (exchange spectroscopy) NMR spectrum indicates a slow exchange between [Me_2_Cl]^+^ and the free MeCl at room temperature.

**Figure 1:**
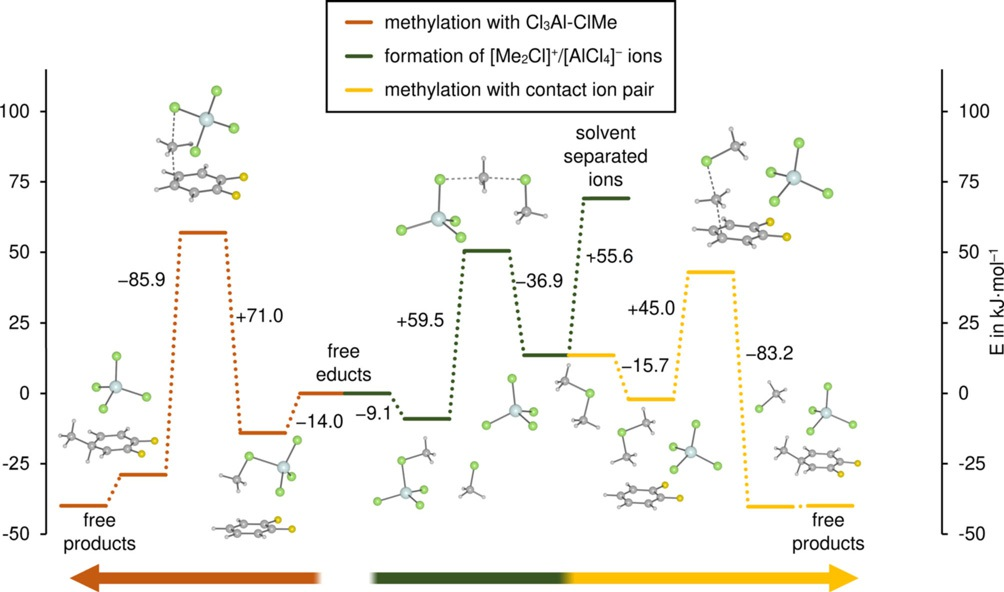
Reaction profiles for the methylation of 1,2‐difluorobenzene with the AlCl_3_–MeCl system. Energies (ZPE corrected) in kJ mol^−1^ on the RI‐B3LYP‐D3/def2‐TZVPP level of theory with COSMO (*ϵ*_R_ MeCl). The final steps (rearomatization and catalyst recovery) are omitted for clarity, and energy differences between linked energy levels are indicated.

We carried out quantum‐chemical calculations at the RI‐B3LYP‐D3/def2‐TZVPP (COSMO, *ϵ*_R_ (relative permittivity) MeCl) level of theory to obtain further information about the relationship between the two intermediates, the activated [Cl_3_Al–ClMe] complex and the dimethylchloronium ion [Me_2_Cl]^+^. These calculations support our NMR spectroscopic observation of a [Cl_3_Al–ClMe] complex (Figure 1). In the presence of 1,2‐difluorobenzene this complex is further stabilized by 14.0 kJ mol^−1^ with respect to the free educts. However, in contrast to common textbook knowledge these calculations disclose that the methylation of 1,2‐difluorobenzene does not take place via the electrophilic [Cl_3_Al–ClMe] complex but via the contact ion pair [Me_2_Cl][AlCl_4_]. The contact ion pair is 22.6 kJ mol^−1^ less stable and separated from the [Cl_3_Al–ClMe] complex by a barrier of 59.5 kJ mol^−1^. Starting from the contact ion pair the methylation reaction of 1,2‐difluorobenzene has a barrier of 45.0 kJ mol^−1^. The direct methylation of 1,2‐difluorobenzene with [Cl_3_Al–ClMe] has a higher barrier of 71.0 kJ mol^−1^ (see Figure 1 and S39). The two intermediates are linked by the equilibrium reaction (**1**).

(1) $$\left[\mathrm{Cl}{}_{3}\mathrm{Al}-\mathrm{ClMe}\right]+\mathrm{MeCl}\vec{\leftarrow }\left[\mathrm{Me}{}_{2}\mathrm{Cl}\right]\left[\mathrm{AlCl}{}_{4}\right]$$

We point out that the choice of the solvent model or the dispersion correction as well as the selected functional does not change the main conclusion drawn from the computed reaction Scheme (see Figure S40). Therefore, the [Me_2_Cl]^+^ cation, as part of a contact ion pair, is a more reasonable intermediate for the Friedel–Crafts methylation reaction in chloromethane.

### Methylation of electron‐deficient aromatic systems

**Scheme 2:**
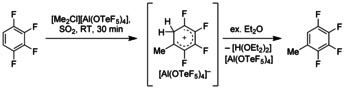
Reaction of [Me_2_Cl][Al(OTeF_5_)_4_] (**1**) with 1,2,3,4‐tetrafluorobenzene.

To further investigate the reactivity of the [Me_2_Cl]^+^ cation we treated a series of deactivated aromatic compounds with [Me_2_Cl][Al(OTeF_5_)_4_] (**1**). Upon addition of a twofold excess of 1,2,3,4‐tetrafluorobenzene to a solution of [Me_2_Cl][Al(OTeF_5_)_4_] (**1**) in SO_2_ (Scheme 2) a slow color change from colorless to a pale yellow color is observed within 30 min at room temperature. The yellowish color of the reaction mixture is typical for fluorinated arenium cations.^[10]^ After adding a slight excess of diethyl ether to the reaction mixture the mixture decolorizes immediately, and the formation of protonated and methylated diethyl ether ([H(OEt_2_)_2_]^+^ and [Me(OEt_2_)]^+^ (ratio 1:9)) is confirmed by ^1^H NMR spectroscopy (Scheme 2). Attempts to isolate the Wheland intermediates were so far unsuccessful. In contrast to Friedel–Crafts methylation using the [Cl_3_Al–ClMe]/MeCl system, where rearomatization of the Wheland intermediate takes places instantaneously during the reaction by liberation of HCl, the rearomatization of the arene intermediate formed in Scheme 2 occurs by the addition of the ether.

**Scheme 3:**
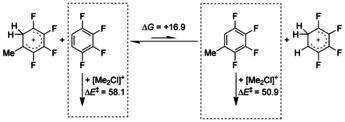
Proposed equilibrium between protonated arene intermediates in the reaction mixture of [Me_2_Cl][Al(OTeF_5_)_4_] (1) with 1,2,3,4‐tetrafluorobenzene; calculated relative transition‐state energies for the methylation reactions (not shown) are given in kJ mol^−1^ on the RI‐B3LYP‐D3/def2‐TZVPP level of with COSMO (*ϵ*_R_ SO_2_).

The NMR spectroscopic analysis of the purified product mixture in CD_2_Cl_2_ confirms the formation of 5‐methyl‐1,2,3,4‐tetrafluorobenzene as well as an excess of the starting compound 1,2,3,4‐tetrafluorobenzene which is in agreement with the H^+^/Me^+^ ratio described above. Increasing the reaction time from 30 min to 3 h at room temperature yields a product mixture of C_6_F_4_H_2_, C_6_F_4_HMe, and C_6_F_4_Me_2_ in the ratio 18:2.4:1. The formation of a two times methylated product can be explained by a fast equilibrium between methyltetrafluorobenzene and protonated tetrafluorobenzene (see Scheme 3). The more basic methyltetrafluorobenzene (see proton affinities in Table 1) reacts faster with the dimethylchloronium cation than tetrafluorobenzene. This observation is also supported by calculated transition state energies, which is 7.2 kJ mol^−1^ lower in energy for the methylation of methyltetrafluorobenzene by [Me_2_Cl]^+^ than that for tetrafluorobenzene. For longer reaction times unspecific decomposition reactions occurred.

**Table 1:** Experimental and calculated proton affinities (PAs) and methyl cation affinities (MCAs).^[a]^

Compound	PA [kJ mol^−1^]	MCA^[b]^ [kJ mol^−1^]
MeCl	647.3^[11]^	260,^[12]^ *279.2* ^[7]^
MeBr	647.3^[11]^	260,^[12]^ *279.2* ^[7]^
1,2,3,4‐tetrafluorobenzene	700.4,^[11]^ *714.9*	*310.0*
MeI	691.7^[11]^	*323.7* ^[7]^
methyl‐1,2,3,4‐tetrafluorobenzene	*746,1*	*338.3*
1,2,3‐trifluorobenzene	724.3,^[11]^ *738.0*	*361.4*
1,2‐difluorobenzene	731.2,^[11]^ *742.9*	*369.0*
4‐methyl‐1,2,3‐trifluorobenzene	*756.0*	*373.8*
4‐methyl‐1,2‐difluorobenzene	*775.3*	*397.6*

[a] Values in *italics* are calculated at the RI‐B3LYP‐D3/def2‐TZVPP level of theory. [b] MCA=−Δ*H* ^0^ for the reaction B+Me^+^→BMe^+^ (B=base).

1,2,3‐Trifluorobenzene and 1,2‐difluorobenzene react within 30 min under quantitative consumption of **1**. After adding diethyl ether to the reaction mixtures, the intense yellow color of the solution vanishes and a quantitative formation of protonated ether is proved ^1^H NMR spectroscopically. The methylation takes place preferentially in *para* position to a fluorine atom (Table 2). This is in agreement with our quantum‐chemical calculations on the RI‐B3LYP‐D3/def2‐TZVPP level of theory with COSMO (*ϵ*_R_ SO_2_) where the transition state for the methylation of 1,2‐difluorobenzene with [Me_2_Cl]^+^ in 4‐position is by 3.1 kJ mol^−1^ lower in energy than in 3‐position. All multi‐methylated isomers up to 4,5,6‐trimethyl‐1,2,3‐trifluorobenzene and 3,4,5,6‐tetramethyl‐1,2‐difluorobenzene are identified by GC/MS and for 1,2,3‐trifluorobenzene also by NMR spectroscopy. Detailed analysis of the spin systems of most methylation products were performed and are given in the supporting information.

**Table 2:** Main products for the methylation with **1** after 30 min of reaction time at room temperature.

Substrate	Consumption of **1**	Main products (percentage in product mixture)			
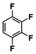	10 %	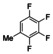	(>90 %)		
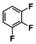	100 %	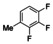	(73 %)		
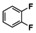	100 %	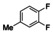	(60 %)	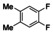	(20 %)

### Reactivity of dimethylbromonium and dimethyliodonium salts

To compare the reactivity of the dimethylchloronium cation with that of the heavier homologues we treated 1,2,3‐trifluorobenzene with the corresponding dimethylbromonium and the dimethyliodonium salts. The dimethylbromonium salt [Me_2_Br][Al(OTeF_5_)_4_] reacts slower with 1,2,3‐trifluorobenzene than the dimethylchloronium salt **1**. The addition of diethyl ether to the reaction mixture after 1 h reaction time yields protonated and methylated diethyl ether in the ratio 1:2.3. The ratio of the product isomers is similar to that for the reaction of **1** with 1,2,3‐trifluorobenzene after 30 min. The dimethyliodonium salt [Me_2_I][Al(OTeF_5_)_4_] does not react with 1,2,3‐trifluorobenzene or 1,2‐difluorobenzene during 24 h at room temperature or during 2 h at 50 °C. While this observation disagrees with calculated (gas‐phase) methyl cation affinity (MCA) values (see Table 1) it is in agreement with our computed transition state energies on the RI‐B3LYP‐D3/def2‐TZVPP level of theory using the COSMO solvent model (*ϵ*_R_ SO_2_). These calculations revel increasing transition state energies for the corresponding methylation reaction of 1,2‐difluorobenzene from 51.7 kJ mol^−1^ (Me_2_Cl^+^) to 56.6 kJ mol^−1^ (Me_2_Br^+^) and 73.6 kJ mol^−1^ (Me_2_I^+^) (see Figure S41 and S42).

### Methylation of weak nitrogen bases

As recently shown, methylation of the weak base PF_3_ with the dimethylchloronium salt (**1**) yields highly electrophilic [MePF_3_]^+^, which readily reacts with the weakly coordinating anion [Al(OTeF_5_)_4_]^−^_._ ^[7]^ We wanted to expand the scope of methylation reactions using **1** to weakly basic *N*‐heteroaromatic compounds. Pentafluoropyridine is known to be a very weak base. The pentafluoropyridinium cation could so far only be isolated as [HNC_5_F_5_][EF_6_] (E=As, Sb)^[13]^ salts. We found that **1** reacts in a fast and quantitative reaction with the fluorinated pyridines NC_5_F_4_X (X=F, I) under formation of the *N*‐methylated products, [MeNC_5_F_4_X][Al(OTeF_5_)_4_] (**2F**/**2I**) [Eq. (2)]. The products, isolated as off‐white solids, are, unlike **1**, stable in dichloromethane solution (see below). The ^1^H NMR spectrum of **2F** shows a triplet of doublets at *δ*(^1^H)=4.35 ppm with couplings to the fluorine atoms in 2‐, 6‐ and 4‐position (^4^ *J*(^19^F,^1^H)=3.3 Hz, ^6^ *J*(^19^F,^1^H)=1.2 Hz) and ^13^C satellites with ^1^ *J*(^13^C,^1^H)=152.7 Hz. The resonance of **2I** is detected in the ^1^H NMR spectrum at *δ*(^1^H)=4.25 ppm and is split into a triplet (^4^ *J*(^19^F,^1^H)=3.3 Hz, ^1^ *J*(^13^C,^1^H)=152.1 Hz). The ^19^F NMR spectra are of higher order featuring A_3_MM'SXX’ (**2F**) and A_3_MM'XX’ (**2I**) spin systems (M, S, X=F; A=H).^[14]^

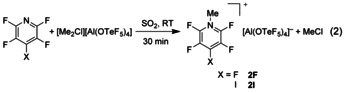



**Figure 2:**
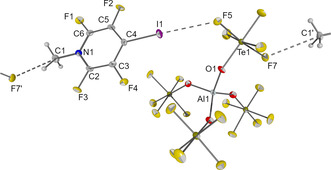
Molecular structure of **2I** in the solid state. Thermal ellipsoids set at 50 % probability. Selected bond lengths [pm] and angles [°]: C1‐N1 150.1(4), N1‐C2 134.4(3), C2‐C3 136.6(4), C3‐C4 138.5(4), C4‐C5 138.6(4), C5‐C6 137.0(4), C6‐N1 134.9(3), C2‐F3 131.4(3), C3‐F4 132.8(3), C4‐I1 205.9(3), C5‐F2 132.9(3), C6‐F1 130.7(3), I1‐F5 320.6(2), C1‐F7’ 307.2(4); C4‐I1‐F5 172.2(1), C2‐N1‐C6 118.5(2), C3‐C4‐C5 116.9(3), F7’‐C1‐N1 170.2(2).

Colorless crystals of **2I** were grown by slowly cooling a dichloromethane solution to −80 °C. It crystallizes in the triclinic space group *P*1(see Figure 2). The shortest contacts between the cation and the anion are a F−C contact (*d*(F7’−C1)=307.2(4) pm, ∢(F7’–C1–N1)=170.2(2)°) and a halogen bond between the iodine and one of the fluorine atoms (F5) of the OTeF_5_ group (*d*(F5−I1)=320.6(2) pm, ∢(C4−I1−F5)=172.2(1)°). The normalized contact (observed distance divided by sum of van der Waals radii)^[15]^ is with 0.92 quite large, indicating a weak interaction. For comparison, in the solid state structure of tetrafluoro‐*para*‐iodopyridine a rather strong I–N interaction with a normalized contact of 0.80 is found.^[16]^ No cocrystals were obtained when **2I** was crystallized from dichloromethane solution at −80 °C in the presence of pentafluoropyridine.

An even less basic and therefore more challenging substrate is cyanuric fluoride,^[17]^ which is methylated with **1** by formation of [MeN_3_C_3_F_3_][Al(OTeF_5_)_4_] (**3**) [Eq. (3)]. This is confirmed by the observed triplet of doublets with ^13^C satellites in the ^1^H NMR spectrum at δ(^1^H)=4.34 ppm (^4^ *J*(^19^F,^1^H)=1.6 Hz, ^6^ *J*(^19^F,^1^H)=0.9 Hz, ^1^ *J*(^13^C,^1^H)=153.0 Hz). The ^15^N NMR signals of all heterocycles shift downfield upon methylation (see Table 3).
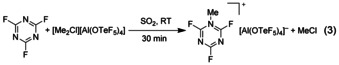



**Table 3:** Experimental and calculated PAs and MCAs^[a]^ in kJ mol^−1^ as well as ^15^N NMR chemical shifts (in ppm) of neutral (*δ* ^15^N, educt) and methylated (*δ* ^15^N, Me^+^) compounds.^[a]^

Compound	PA	MCA	*δ* ^15^N, educt	*δ* ^15^N, Me^+^
CH_2_Cl_2_	628±8,^[18]^ *643.1*	*265.4*	–	–
CH_2_Cl(OTeF_5_)	*683.0*	*267.3*	–	–
MeCl	647.3^[11]^	*279.2* ^[7]^	–	–
N_3_C_3_F_3_	*758.5*	*358.5*	−166.3^[b]^	−220.0 (N1)^[b]^
NC_5_F_5_	764.9,^[11]^ *781.6*	*376.7*	−145.5^[c]^	−213.2^[d]^
NC_5_F_4_I	*807.4*	*400.0*	−131.4^[c]^	−208.0^[d]^

[a] Values in *italics* are calculated at the RI‐B3LYP‐D3/def2‐TZVPP level of theory. [b] SO_2_, ext. [D6]acetone. [c] CDCl_3_. [d] CD_2_Cl_2_.

**Figure 3:**
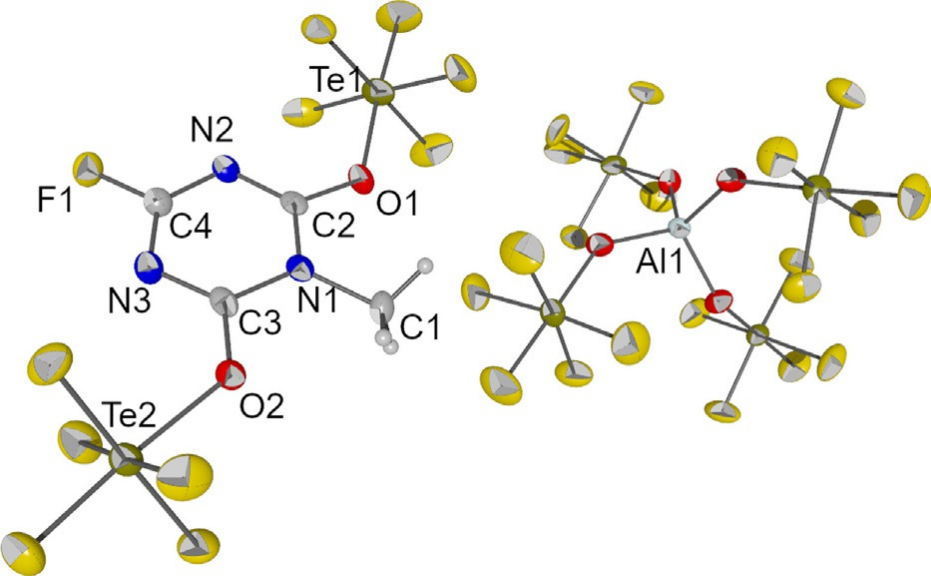
Molecular structure of [MeN_3_C_3_F(OTeF_5_)_2_][Al(OTeF_5_)_4_] in the solid state. Thermal ellipsoids are shown at 50 % probability. Selected bond lengths [pm] and angles [°]: C1‐N1 148.7(8), N1‐C2 135.6(8), C2‐N2 131.3(8), N2‐C4 131.7(9), C4‐N3 131.6(8), N3‐C3 130.8(8), C3‐N1 137.3(9), C2‐O1 131.3(9), C4‐F1 129.9(7), C3‐O2 129.8(8), O1‐Te1 193.8(5), O2‐Te2 194.0(5); C2‐N1‐C3 116.1(6), N1‐C2‐N2 123.7(6), C2‐N2‐C4 114.0(6), N2‐C4‐N3 129.0(6), C4‐N3‐C3 114.2(6), N3‐C3‐N1 123.1(6), C2‐O1‐Te1 127.9(5), C3‐O2‐Te2 126.9(5), N2‐C2‐O1‐Te1 9.3(9), N3‐C3‐O2‐Te2 4.0(9).

From a solution of **3** in dichloromethane crystals of [MeN_3_C_3_F(OTeF_5_)_2_][Al(OTeF_5_)_4_] were grown by slowly cooling a dichloromethane solution to −40 °C. The compound crystallizes in monoclinic space group *P*2_1_/*c* (see Figure 3). At room temperature a colorless solution of **3** in dichloromethane decomposes within one day to a two‐phase system with a dark and oily lower phase. This decomposition shows the highly Lewis‐acidic character of the [MeN_3_C_3_F_3_]^+^ cation.

Also the dimethylchloronium salt **1** decomposes in SO_2_ solution within days at room temperature under formation of MeOTeF_5_.^[7]^ In contrast to this, the addition of dichloromethane at room temperature to solid **1** results in an immediate decomposition to a dark brown suspension. At −40 °C the [Me_2_Cl]^+^ salt **1** has a poor solubility in dichloromethane and decomposes upon slow warming with a quantitative consumption of the weakly coordinating anion [Al(OTeF_5_)_4_]^−^ to a yellow solution. Using solvent suppression pulse sequences CH_2_Cl(OTeF_5_) and CH_2_(OTeF_5_)_2_ (ratio 10:1, 1000‐fold excess of CH_2_Cl_2_) can be identified NMR spectroscopically as the only pentafluoro‐*ortho*‐tellurate‐containing species [see Eqs. 4 and 5].
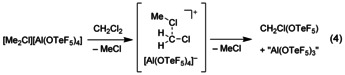


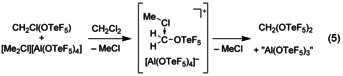



**Figure 4:**
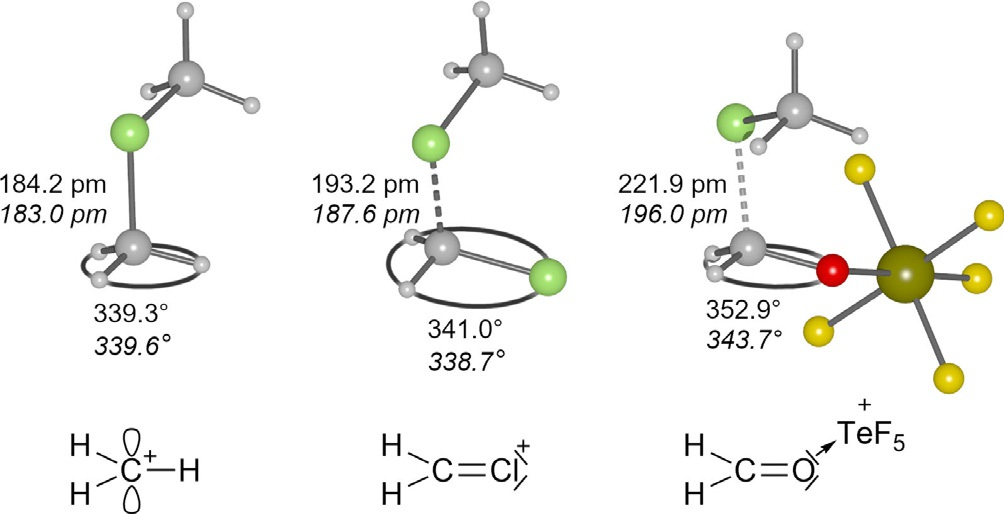
Calculated structures of [Me_2_Cl]^+^, [MeCl⋅⋅⋅CH_2_Cl]^+^ and [MeCl⋅⋅⋅CH_2_OTeF_5_]^+^ on the RI‐B3LYP‐D3/def2‐TZVPP level of theory; values in *italics* are with COSMO (*ϵ*_R_ CH_2_Cl_2_); and Lewis structures of underlying [CH_3_]^+^, [CH_2_Cl]^+^ and [CH_2_OTeF_5_]^+^ according to the NBO analysis.

These species are likely formed via the intermediates [MeCl⋅⋅⋅CH_2_Cl]^+^ and [MeCl⋅⋅⋅CH_2_OTeF_5_]^+^, which both can be described as carbenium cations coordinated by a MeCl molecule (see Figure 4). These highly reactive carbenium cations are much less stabilized than the methyl groups in the dimethylchloronium ion. They exhibit considerably longer C−Cl distances than [Me_2_Cl]^+^ and less deviation from a planar geometry at the carbon atom as expressed by their higher angular sums. According to an NBO analysis^[19]^ the [CH_2_OTeF_5_]^+^ cation is described as a CH_2_O moiety coordinating to a TeF_5_^+^ group. Thus, the formation of such highly electrophilic carbenium ions ([MeCl⋅⋅⋅CH_2_OTeF_5_]^+^) can probably explain the fast decomposition of the [Me_2_Cl]^+^ salt **1** in dichloromethane.

## Conclusions

We were able to show that dialkylhalonium ions are the key intermediates during the classical Friedel–Crafts methylation reactions. In addition, we reported the methylation of weakly basic oligofluorobenzenes with the dimethylchloronium salt [Me_2_Cl][Al(OTeF_5_)_4_] (**1**),^[7]^ where the electrophilic attack, that is, the methylation step, and the rearomatization are separated in contrast to typical Friedel–Crafts reactions using the [Cl_3_Al–ClMe]/MeCl system.

The reaction of **1** with weakly basic fluorinated nitrogen‐containing heterocycles leads to the formation of *N*‐methylated products. It has been shown that the rapid decomposition reaction of **1** in dichloromethane results in the formation of CH_2_Cl(OTeF_5_) and CH_2_(OTeF_5_)_2_. Further investigations on methylation reactions as well as attempts to isolate Wheland intermediates are continuing in our group.

## Experimental Section

The experiments were performed under exclusion of air and moisture using standard Schlenk techniques. The solvent SO_2_ was dried over CaH_2_. The oligofluorobenzenes, CH_2_Cl_2_ and CD_2_Cl_2_ were dried over Sicapent while diethyl ether was dried over Solvona. MeCl (purchased from abcr) was used without further purification. Triethylaluminium was purchased from abcr and handled in a glovebox under a dry argon atmosphere. Teflic acid was prepared according to literature^[20]^ as well as cyanuric fluoride^[21]^ and tetrafluoro‐*para*‐iodopyridine.^[16]^ The salts [Me_2_Cl][Al(OTeF_5_)_4_], [Me_2_Br][Al(OTeF_5_)_4_] and [Me_2_I][Al(OTeF_5_)_4_] where synthesized as already described.^[7]^ The salt [NEt_4_][AlCl_4_] was synthesized according to the literature with MeCl as a solvent instead of thionyl chloride.^[22]^ IR spectra were recorded on a Bruker ALPHA FTIR spectrometer inside a glovebox equipped with a diamond ATR attachment (resolution 4 cm^−1^). Raman spectra were recorded on a Bruker MultiRAM II equipped with a low‐temperature Ge detector (1064 nm, 30–80 mW, resolution 2 cm^−1^). NMR spectra were recorded on a JEOL 400 MHz ECS, 400 MHz ECZ or 600 MHz ECZ spectrometer or on a Bruker 700 MHz AVANCE700. For strongly coupled spin systems all chemical shifts and coupling constants were reported as simulated in gNMR.^[14]^ Spin–spin coupling constants calculated with Gauge‐Independent Atomic Orbital method (GIAO)^[23]^ on B3LYP/aug‐cc‐pVTZ‐J^[24]^ level of theory and literature data for non‐methylated species^[25]^ provided a reasonable first guess, signs of coupling constants were used directly from these sources. All reported chemical shifts were referenced to the Ξ values given in IUPAC recommendations of 2008^[26]^ using the ^2^H signal of the deuterated solvent as internal reference. For ^14^N/^15^N MeNO_2_ is used as reference. For external locking [D6]acetone was flame sealed in a glass capillary and the lock oscillator frequency was adjusted to give *δ*(^1^H)=7.26 ppm for a CHCl_3_ sample. Mass spectra were recorded on an Advion Compact mass spectrometer expression L with a quadrupole mass filter. Samples were dissolved in a dry solvent (CH_3_CN or CH_2_Cl_2_) for ESI (electrospray ionization). GC‐MS were measured on a Saturn 2100 GC/MS system from Varian Inc. equipped with a „HP‐5 ms Ultra Inert“ (length 30 m) column, injection volume 1 μL, split 100. The following temperature program was used: 50 °C for 0.5 min, ramp with 20 °C min^−1^ to 80 °C, hold for 1 min, ramp with 10 °C min^−1^ to 120 °C, ramp with 20 °C min^−1^ to 250 °C, constant helium gas flow of 280 L min^−1^. Ionization voltage for EI (electron ionization): 80 eV. Crystal data were collected on a Bruker D8 Venture diffractometer with a Photon 100 CMOS area detector with Mo K_α_ radiation. Using Olex2,^[27]^ the structures were solved with the ShelXT^[28]^ structure solution program by intrinsic phasing and refined with ShelXL^[29]^ refinement package using least square minimization. Crystal structures were visualized with Diamond.^[30]^

For density functional calculations the program package TURBOMOLE^[31]^ was used with its implementations of RI,^[32]^ MARI‐J,^[33]^ B3LYP,^[34]^ Grimme‐D3^[35]^ together with the basis set def2‐TZVPP.^[36]^ SCF energies were corrected with chemical potential taken from TURBOMOLE implemented in the freeh script to get free enthalpies. For single point calculations the functionals m06^[37]^ and B2‐PLYP^[38]^ were used as implemented in TURBOMOLE. For COSMO^[39]^‐optimized structures vibrational spectra were calculated numerically. For MeCl *ϵ*_R_=10^[40]^ and for SO_2_ *ϵ*_R_=17.6^[41]^ were used as 20 °C near values. NBO analysis was performed with NBO 7.0^[42]^ executed from Gaussian 16^[43]^ as well as GIAO calculations.

***Caution***! Chloromethane and SO_2_ give a pressure of 4.9 bar and 3.3 bar, respectively, at room temperature; care must be taken that reaction vessels resist this pressure.

Chloromethane and SO_2_ were treated as ideal gases and measured via their pressure in a known volume. When cooled to −70 °C liquefied SO_2_ can be easily transferred using inert PFA (perfluoroalkoxy alkane) or PTFE tubes; however, a small amount of the solvent evaporates by cooling the tube so concentrations are changing. This is not possible with MeCl.

### Methylation of oligofluorobenzenes: general procedure

To a solution of [Me_2_Cl][Al(OTeF_5_)_4_] (**1**) in SO_2_ a slight excess of the substrate was added at −30 °C. The initially colorless reaction mixture changed color to intense yellow while stirring at room temperature for 30 min. Afterwards, diethyl ether was added at −30 °C, resulting in a decolorization. The [H(OEt_2_)_2_]^+^ and [MeOEt_2_]^+^ ratio was determined from this solution by ^1^H NMR spectroscopy. All volatiles were condensed into a second flask. SO_2_ was carefully removed under reduced pressure at −20 °C. The resulting clear colorless liquid was diluted with CD_2_Cl_2_ and analyzed by NMR spectroscopy.

**[MeOEt_2_][Al(OTeF_5_)_4_]**: ^1^H NMR (400 MHz, CD_2_Cl_2_, 20 °C): *δ*=4.70 (q, 4 H, C*H*_2_, ^3^ *J*(^1^H,^1^H)=7.1 Hz), 4.24 (s, 3 H, OC*H*_3_), 1.68 (t, 6 H, CH_2_C*H*_3_, ^3^ *J*(^1^H,^1^H)=7.1 Hz) ppm. ^1^H NMR (400 MHz, SO_2_, ext. [D6]acetone, 20 °C): *δ*=5.84 (q, 4 H, C*H*_2_, ^3^ *J*(^1^H,^1^H)=7.2 Hz), 5.37 (s, 3 H, OC*H*_3_), 2.74 (t, 6 H, CH_2_C*H*_3_, ^3^ *J*(^1^H,^1^H)=7.2 Hz) ppm ppm^−1^. ^1^H,^13^C‐HMQC NMR (400 MHz/101 MHz, CD_2_Cl_2_, 20 °C): *δ=*4.70/88.7 (C*H*_2_/*C*H_2_), 4.24/70.3 (OC*H*_**3**_/O*C*H_3_), 1.68/11.7 (CH_2_C*H*_**3**_/CH_2_ *C*H_3_)  ppm ppm^−1^. ^1^H,^13^C‐HMBC NMR (400 MHz/101 MHz, CD_2_Cl_2_, 20 °C): *δ*=4.70/11.7 (C*H*_**2**_, CH_2_ *C*H_3_), 4.24/88.7 (OC*H*_**3**_/*C*H_2_), 1.68/88.7 (CH_2_C*H*_**3**_/*C*H_2_)  ppm.

**[H(OEt_2_)_2_][Al(OTeF_5_)_4_]**: ^1^H NMR (400 MHz, SO_2_, ext. [D6]acetone, 20 °C): *δ*=17.38 (s br, 1 H, H), 5.23 (q, 8 H, C*H*_2_, ^3^ *J*(^1^H,^1^H)=7.1 Hz), 2.55 (t, 12 H, C*H*_3_, ^3^ *J*(^1^H,^1^H)=7.1 Hz).

### 1,2,3,4‐Tetrafluorobenzene

The following amounts were used for a 30 min reaction: **1**: 372 mg, 0.356 mmol, SO_2_: 22 mmol, approx. 1.0 mL, 1,2,3,4‐tetrafluorobenzene: 0.05 mL, 0.467 mmol.

The following amounts were used for a 3 h reaction: **1**: 401 mg, 0.383 mmol, SO_2_: 22 mmol, approx. 1.0 mL, 1,2,3,4‐tetrafluorobenzene: 0.05 mL, 0.467 mmol.

**5‐Methyl‐1,2,3,4‐tetrafluorobenzene**: ^1^H NMR (400 MHz, CD_2_Cl_2_, 20 °C): *δ*=6.81 (m, 1 H, Ar*H*, ^3^ *J(* ^19^F,^1^H)=10.65 Hz, ^4^ *J*(^19^F,^1^H)=8.11 Hz (F2), ^5^ *J*(^19^F,^1^H)=−2.61 Hz, ^4^ *J*(^19^F,^1^H)=6.46 Hz (F4)), 2.23 (m, 3 H, C*H*_3_, ^4^ *J*(^19^F,^1^H)=2.41 Hz, ^6^ *J*(^19^F,^1^H)=1.38 Hz) ppm. ^19^F NMR (377 MHz, CD_2_Cl_2_, 20 °C): *δ*=−142.0 (dddd, 1F, *F*1, ^3^ *J*(^19^F,^19^F)=−21.02 Hz, ^4^ *J*(^19^F,^19^F)=−1.87 Hz, ^5^ *J*(^19^F,^19^F)=12.49 Hz, ^3^ *J*(^19^F,^1^H)=10.65 Hz), −143.9 (dddtd, 1F, *F*4, ^3^ *J*(^19^F,^19^F)=−20.15 Hz, ^4^ *J*(^19^F,^19^F)=−1.67 Hz, ^5^ *J*(^19^F,^19^F)=12.49 Hz, ^4^ *J*(^19^F,^1^H)=6.46 Hz (H5), ^4^ *J*(^19^F,^1^H)=2.41 Hz (CH_3_)), −158.3 (dddd, 1F, *F*3, ^3^ *J*(^19^F,^19^F)=−20.15 Hz (F4), ^3^ *J*(^19^F,^19^F)=−19.5 Hz (F2), ^4^ *J*(^19^F,^19^F)=−1.87 Hz, ^5^ *J*(^19^F,^1^H)=−2.61 Hz), −161.3 (ddddt, 1F, *F*2, ^3^ *J*(^19^F,^19^F)=−21.02 Hz (F1), ^3^ *J*(^19^F,^19^F)=−19.45 Hz (F3), ^4^ *J*(^19^F,^19^F)=−1.67 Hz, ^4^ *J*(^19^F,^1^H)=8.11 Hz, ^6^ *J*(^19^F,^1^H)=1.38 Hz) ppm. GC‐MS: *t*_R_=2.72 min, *m*/*z=*163.1 (calc: 163.0 [M−H]^.+^).

**Dimethyl‐1,2,3,4‐tetrafluorobenzene**: A_3_A’_3_MM'XX’ spin system. ^1^H NMR (400 MHz, CD_2_Cl_2_, 20 °C): *δ*=2.15 (m, 6 H, C*H*_3_, H_A_/H_A’_) ppm. ^19^F NMR (377 MHz, CD_2_Cl_2_, 20 °C): *δ*_MM’_=−144.6 ppm (F1/F4), *δ*_BB’_=−162.7 ppm (F2/F3), *J*_MX_=*J*_M'X’_=^3^ *J*(^19^F,^19^F)=−21.79 Hz, *J*_MX’_=*J*_M'X_=^4^ *J*(^19^F,^19^F)=1.61 Hz, *J*_MM’_=^5^ *J*(^19^F,^19^F)=12.68 Hz, *J*_XX’_=^3^ *J*(^19^F,^19^F)=−19.30 Hz, *J*_XA_=*J*_X'A’_=^6^ *J*(^19^F,^1^H)=1.43 Hz, *J*_MA’_=*J*_M'A_=^4^ *J*(^19^F,^1^H)=2.48 Hz. GC‐MS: t_R_=4.25 min, *m*/*z=*178.1 (calc: 178.0 [M]^.+^).

### 1,2,3‐Trifluorobenzene

The following amounts were used for a 30 min reaction: **1**: 495 mg, 0.473 mmol, SO_2_: 27 mmol, approx. 1.3 mL, 1,2,3‐trifluorobenzene: 0.05 mL, 0.485 mmol.

**Main product—4‐methyl‐1,2,3‐trifluorobenzene**: ABM_3_SVZ spin system. ^1^H NMR (600 MHz, CD_2_Cl_2_, 20 °C): *δ*=6.89 (m, 1 H, *H*5, H_B_), 6.86 (m, 1 H, *H*6, H_A_), 2.21 (m, 3 H, C*H*_3_, H_M_) ppm. ^19^F NMR (565 MHz, CD_2_Cl_2_, 20 °C): *δ*=−139.0 (m, 1F, *F*3, F_S_), −139.8 (m, 1F, *F*1, F_V_), −163.0 (m, 1F, *F*2, F_Z_) ppm. Coupling constants: *J*_VZ_=^3^ *J*(^19^F,^19^F)=−20.19 Hz, *J*_SZ_=^3^ *J*(^19^F,^19^F)=−19.85 Hz, *J*_S*V*=_^4^ *J*(^19^F,^19^F)=5.62 Hz, *J*_A*V*=_^4^ *J*(^19^F,^1^H)=5.63 Hz, *J*_AZ_=^5^ *J*(^19^F,^1^H)=−2.53 Hz, *J*_AS_=^4^ *J*(^19^F,^1^H)=7.92 Hz, *J*_B*V*=_^3^ *J*(^19^F,^1^H)=9.89 Hz, *J*_BZ_=^4^ *J*(^19^F,^1^H)=7.24 Hz, *J*_BS_=^5^ *J*(^19^F,^1^H)=−2.40 Hz, *J*_AB_=^3^ *J*(^1^H,^1^H)=8.62 Hz, *J*_AM_=^4^ *J*(^1^H,^1^H)=−1.00 Hz, *J*_BM_=^5^ *J*(^1^H,^1^H)=0.40 Hz, *J*_SM_=^4^ *J*(^19^F,^1^H)=2.32 Hz, *J*_VM_=^6^ *J*(^19^F,^1^H) 1.33 Hz. GC‐MS: t_R_=2.78 min, *m*/*z=*145.1 (calc: 145.0 [M−H]^.+^).

### 1,2‐Difluorobenzene

The following amounts were used for a 30 min reaction: **1**: 1.75 g, 1.67 mmol, SO_2_: 110 mmol, approx. 5 mL, 1,2‐difluorobenzene: 0.17 mL, 1.71 mmol.

**4‐Methyl‐1,2‐difluorobenzene**: ABCM_3_SX spin system. ^1^H NMR (400 MHz, CD_2_Cl_2_, 20 °C): *δ*=7.01 (m, 1 H, *H*6, H_A_), 6.95 (m, 1 H, *H*3, H_B_), 6.85 (m, 1 H, *H*5, H_C_), 2.27 (m, 3 H, C*H*_3_, H_M_) ppm. ^19^F NMR (377 MHz, CD_2_Cl_2_, 20 °C): *δ*=−140.1 (m, 1F, *F*2, F_S_), −144.4 (m, 1F, *F*1, F_X_) ppm; coupling constants: *J*_SX_=^3^ *J*(^19^F,^19^F)=−21.17, *J*_AS_=^3^ *J*(^19^F,^1^H)=10.60 Hz, *J*_AX_=^4^ *J*(^19^F,^1^H)=8.36 Hz, *J*_BS_=^4^ *J*(^19^F,^1^H)=7.73 Hz, *J*_BX_=^3^ *J*(^19^F,^1^H)=11.58 Hz, J_CS_=^4^ *J*(^19^F,^1^H)=4.18 Hz, J_CX_=^5^ *J*(^19^F,^1^H)=−1.44 Hz, *J*_MS_=^6^ *J*(^19^F,^1^H)=1.33 Hz, *J*_AC_=^3^ *J*(^1^H,^1^H)=8.92 Hz, *J*_AB_=^5^ *J*(^1^H,^1^H)=0.30 Hz, *J*_BC_=^4^ *J*(^1^H,^1^H)=2.10 Hz, *J*_BM_=^4^ *J*(^1^H,^1^H)=0.75 Hz, J_CM_=^3^ *J*(^1^H,^1^H)=−0.75 Hz. GC‐MS: *t*_R_=2.82 min, *m*/*z=*127.1 (calc: 127.0 [M−H]^.+^).

**4,5‐Dimethyl‐1,2‐difluorobenzene**: AA'M_3_M_3_’XX’ spin system. ^1^H NMR (400 MHz, CD_2_Cl_2_, 20 °C): *δ*=6.90 (m, 2 H, Ar*H*, H_A_/H_A’_), 2.16 (m, 6 H, C*H*_3_, H_M_/H_M’_) ppm. ^19^F NMR (377 MHz, CD_2_Cl_2_, 20 °C): *δ*=−144.7 (m, 2F, Ar*F*, F_X_/F_X’_) ppm; coupling constants: J_XX’_=^3^ *J*(^19^F,^19^F)=−20.00 Hz, *J*_AX_=*J*_A'X’_=^3^ *J*(^19^F,^1^H)=9.90 Hz, *J*_AX’_=*J*_A'X_=^4^ *J*(^19^F,^1^H)=9.90 Hz, *J*_MX’_=*J*_M'X_=^6^ *J*(^19^F,^1^H)=1.00 Hz, *J*_AA’_=^5^J(^1^H,^1^H)=1.20 Hz. GC‐MS: *t*_R_=4.28 min, *m*/*z=*142.1 (calc: 142.1 [M]^.+^).

### 1,2,3‐Trifluorobenzene with [Me_2_Br]^+^

The following amounts were used for a 60 min reaction: [Me_2_Br][Al(OTeF_5_)_4_]: 332 mg, 0.304 mmol, SO_2_: 22 mmol, approx. 1.0 mL, 1,2,3‐trifluorobenzene: 0.05 mL, 0.485 mmol. Product ratio according to NMR identical to the activation with **1**. However, [MeOEt_2_]^+^/[H(OEt_2_)_2_]^+^ ratio in residual solid was 2.3/1.

### Methylation attempts with [Me_2_I]^+^

The following amounts were used for a 24 h reaction: [Me_2_I][Al(OTeF_5_)_4_]: 407 mg, 0.357 mmol, SO_2_: 27 mmol, approx. 1.3 mL, 1,2,3‐trifluorobenzene: 0.05 mL, 0.485 mmol. No color change was observed. No methylation was observed by NMR spectroscopy. To a sample in a Young NMR tube an excess of 1,2‐difluorobenzene is added. No color change was observed. No methylation was observed by NMR spectroscopy.

The following amounts were used for 2 h reaction at 50 °C. ***Caution**! SO_2_ has a vapor pressure of approximately 8 bar at 50 °C*! [Me_2_I][Al(OTeF_5_)_4_]: 201 mg, 0.177 mmol, SO_2_: 22 mmol, approx. 1.0 mL, 1,2,3‐trifluorobenzene: 0.05 mL, 0.485 mmol. No color change was observed. No methylation was observed by NMR spectroscopy. After addition of diethyl ether only [MeOEt_2_]^+^ was detected.

### [MeNC_5_F_4_I][Al(OTeF_5_)_4_] (2I)

Tetrafluoro‐*para*‐iodopyridine (103 mg, 0.372 mmol, 1.05 equiv) was sublimed in vacuum onto a frozen solution of **1** (370 mg, 0.354 mmol) in SO_2_ (33 mmol, approx. 1.5 mL). The reaction mixture as allowed to melt and stirred at room temperature for 30 min. Removal of all volatiles under reduced pressure at room temperature yielded [MeNC_5_F_4_I][Al(OTeF_5_)_4_] (**2I**, 451 mg, 0.354 mmol) as an off‐white powder. ^1^H NMR (400 MHz, CD_2_Cl_2_, 20 °C): *δ*=4.25 (t, 98.9 %, N^12^C*H*_3_, ^4^ *J*(^19^F,^1^H)=3.27 Hz; dt, 1.1 %, N^13^C*H*_3_, ^1^ *J*(^13^C,^1^H)=152.1 Hz, ^4^ *J*(^19^F,^1^H)=3.27 Hz) ppm. ^1^H NMR (400 MHz, SO_2_, ext. [D6]acetone, 20 °C): *δ*=5.59 (t, 98.9 %, N^12^C*H*_3_, ^4^ *J*(^19^F,^1^H)=3.3 Hz; dt, 1.1 %, N^13^C*H*_3_, ^1^ *J*(^13^C,^1^H)=152.1 Hz, ^4^ *J*(^19^F,^1^H)=3.3 Hz) ppm. ^13^C{^19^F,^1^H} NMR (101 MHz, CD_2_Cl_2_, 20 °C): *δ*=146.7 (*C*3/*C*5), 142.6 (*C*2/*C*6), 107.2 (*C*4), 37.1 (*C*H_3_)  ppm. ^1^H,^15^N HMBC NMR (400 MHz/41 MHz, CD_2_Cl_2_, 20 °C): *δ*=4.25 ppm/−208 ppm. ^19^F NMR (377 MHz, CD_2_Cl_2_, 20 °C): cation: A_3_MM'XX’ spin system. δ_MM’_=−99.7 (F2/F6) ppm, δ_XX’_=−112.2 (F3/F5) ppm, *J*_MM’_=^4^ *J*(^19^F,^19^F)=−18.26 Hz, *J*_MX_=*J*_M'X’_=^3^ *J*(^19^F,^19^F)=16.64 Hz, *J*_M'X_=*J*_MX’_=^5^ *J*(^19^F,^19^F)=−12.73, *J*_XX’_=^4^ *J*(^19^F,^19^F)=−2.16 Hz, *J*_MA_=*J*_M'A_=^4^ *J*(^19^F,^1^H)=3.27 Hz; anion: *δ*=−38.5 (m, **A**B_4_X, 1F, ^2^ *J*(^19^F,^19^F)=187.8 Hz, ^1^ *J*(^125^Te,^19^F)=3366.0 Hz), −46.1 (m, A**B_4_**X, 4F, ^2^ *J*(^19^F,^19^F)=187.8 Hz, ^1^ *J*(^125^Te,^19^F)=3478.0 Hz) ppm. ^27^Al{^19^F} NMR (104 MHz, CD_2_Cl_2_, 20 °C): *δ*=46.8 (s, 73.2 %, [Al(OTeF_5_)_4_]^−^; d, 22.2 %, [Al(OTeF_5_)_3_(O^125^TeF_5_)]^−^, ^2^ *J*(^125^Te,^27^Al)=73.2 Hz; d, 2.8 %, [Al(OTeF_5_)_3_(O^123^TeF_5_)]^−^, ^2^ *J*(^123^Te,^27^Al)=61.2 Hz; t, 2.6 %, [Al(OTeF_5_)_2_(O^125^TeF_5_)_2_]^−^, ^2^ *J*(^125^Te,^27^Al)=73.2 Hz; t, 0.04 %, [Al(OTeF_5_)_2_(O^123^TeF_5_)_2_]^−^, ^2^ *J*(^123^Te,^27^Al)=61.3 Hz) ppm. IR (ATR, 25 °C): $\tilde{\nu }$ =1657 (m), 1587 (vw), 1527 (m), 1489 (vw), 1438 (w), 1315 (w), 1285 (w), 1135 (vw), 987 (sh), 945 (sh), 928 (s, ν(Al‐O)), 818 (m, ν(C‐I)), 687 (vs, ν(Te‐F)), 641 (w), 580 (w), 543 (m) cm^−1^. FT‐Raman (25 °C): $\tilde{\nu }$ =2992 (m), 2964 (w), 1660 (s), 1439 (m), 1387 (m), 1286 (m), 989 (m), 821 (w), 719 (w), 697 (vs), 647 (s), 584 (m), 515 (m), 457 (m), 421 (m), 375 (w), 354 (w), 335 (m), 302 (m), 205 (w), 134 (w) cm^−1^.

### [MeNC_5_F_5_][Al(OTeF_5_)_4_] (2F)

Pentafluoropyridine (0.06 mL, 0.547 mmol) was added to a solution of **1** (476 mg, 0.455 mmol) in SO_2_ (44 mmol, approx. 2.0 mL) at −30 °C. The reaction mixture was allowed to warm to room temperature and stirred for 30 min. All volatiles were removed under reduced pressure at room temperature to yield [MeNC_5_F_5_][Al(OTeF_5_)_4_] (**2F**, 530 mg, 0.455 mmol) as an off‐white powder. ^1^H NMR (400 MHz, CD_2_Cl_2_, 20 °C): *δ*=4.35 (td, 98.9 %, N^12^C*H*_3_, ^4^ *J*(^19^F,^1^H)=3.32 Hz, ^6^ *J*(^19^F,^1^H)=1.24 Hz; dtd, 1.1 %, N^13^C*H*_3_, ^1^ *J*(^13^C,^1^H)=152.7 Hz, ^4^ *J*(^19^F,^1^H)=3.32 Hz, ^6^ *J*(^19^F,^1^H)=1.24 Hz) ppm. ^1^H NMR (400 MHz, SO_2_, ext. [D6]acetone, 20 °C): *δ*=5.68 (td, 98.9 %, N^12^C*H*_3_, ^4^ *J*(^19^F,^1^H)=3.3 Hz, ^6^ *J*(^19^F,^1^H)=1.2 Hz; dtd, 1.1 %, N^13^C*H*_3_, ^1^ *J*(^13^C,^1^H)=152.7 Hz, ^4^ *J*(^19^F,^1^H)=3.3 Hz, ^6^ *J*(^19^F,^1^H)=1.2 Hz) ppm. ^13^C{^19^F,^1^H} NMR (101 MHz, CD_2_Cl_2_, 20 °C): *δ*=155.9 (*C*4), 146.3 (*C*2/*C*6), 136.5 (*C*3/*C*5), 37.0 (*C*H_3_) ppm. ^1^H,^15^N HMBC NMR (400 MHz/41 MHz, CD_2_Cl_2_, 20 °C): *δ*=4.35 ppm/−213.2 ppm. ^19^F NMR (377 MHz, CD_2_Cl_2_, 20 °C): cation: A_3_MM'SXX’ spin system, *δ*_MM’_=−93.3 (F2/F6) ppm, *δ*_S_=−103.5 (F4) ppm, *δ*_XX’_=−150.8 (F3/F5) ppm, *J*_MM’_=^4^ *J*(^19^F,^19^F)=−20.00 Hz, *J*_MS_=*J*_M'S_=^4^ *J*(^19^F,^19^F)=25.59 Hz, *J*_MX_=*J*_M'X’_=^3^ *J*(^19^F,^19^F)=−11.72 Hz, *J*_M'X_=*J*_MX’_=^5^ *J*(^19^F,^19^F)=12.69 Hz, *J*_SX_=*J*_SX’_=^3^ *J*(^19^F,^19^F) −22.95 Hz, *J*_XX’_=^4^ *J*(^19^F,^19^F)=4.42 Hz, *J*_MA_=*J*_M'A_=^4^ *J*(^19^F,^1^H)=3.32 Hz, *J*_SA_=^6^ *J*(^19^F,^1^H)=1.24 Hz; anion: *δ*=−38.6 (m, **A**B_4_X, 1F, ^2^ *J*(^19^F,^19^F)=187.4 Hz, ^1^ *J*(^125^Te,^19^F)=3332.0 Hz), −46.3 (m, A**B_4_**X, 4F, ^2^ *J*(^19^F,^19^F)=187.4 Hz, ^1^ *J*(^125^Te,^19^F)=3470.0 Hz) ppm. ^27^Al{^19^F} NMR (104 MHz, CD_2_Cl_2_, 20 °C): *δ*=46.8 (s, 73.4 %, [Al(OTeF_5_)_4_]^−^; d, 22.2 %, [Al(OTeF_5_)_3_(O^125^TeF_5_)]^−^, ^2^ *J*(^125^Te,^27^Al)=73.4 Hz; d, 2.8 %, [Al(OTeF_5_)_3_(O^123^TeF_5_)]^−^, ^2^ *J*(^123^Te,^27^Al)=61.9 Hz; t, 2.6 %, [Al(OTeF_5_)_2_(O^125^TeF_5_)_2_]^−^, ^2^ *J*(^125^Te,^27^Al)=73.4 Hz; t, 0.04 %, [Al(OTeF_5_)_2_(O^123^TeF_5_)_2_]^−^, ^2^ *J*(^123^Te,^27^Al)=61.3 Hz) ppm. IR (ATR, 25 °C): $\tilde{\nu }$ =1683 (m), 1605 (w), 1548 (s), 1401 (w), 1352 (w), 1289 (w), 1152 (m), 981 (m), 930 (s, ν(Al‐O)), 688 (vs, ν(Te‐F)), 641 (m), 614 (w), 550 (s), 450 (w) cm^−1^. ESI‐MS (acetonitrile, positive mode): *m*/*z=*184.0 ([MeNC_5_F_5_]^+^, calc: 184.1).

### [MeN_3_C_3_F_3_][Al(OTeF_5_)_4_]

To a solution of **1** (392 mg, 0.375 mmol) in SO_2_ (44 mmol, approx. 2.0 mL) cyanuric fluoride (0.07 mL, 0.816 mmol, 2.2 equiv) was added at −30 °C. The reaction mixture was allowed to reach room temperature and stirred for 30 min. All volatiles were removed under reduced pressure at room temperature. The resulting yellowish oil was dissolved in 0.5 mL CH_2_Cl_2_. 5.0 mL *n*‐pentane were added quickly at room temperature to precipitate the salt. The solution was separated by filtration to leave [MeN_3_C_3_F_3_][Al(OTeF_5_)_4_] after drying in vacuum as a white powder (396 mg, 0.350 mmol). Cooling a solution of [MeN_3_C_3_F_3_][Al(OTeF_5_)_4_] in dichloromethane to −40 °C yielded crystals of [MeN_3_C_3_F(OTeF_5_)_2_][Al(OTeF_5_)_4_] suitable for X‐ray diffraction. ^1^H NMR (400 MHz, CD_2_Cl_2_, 20 °C): *δ*=4.34 (td, 98.9 %, N^12^C*H*_3_, ^4^ *J*(^19^F,^1^H)=1.6 Hz, ^6^ *J*(^19^F,^1^H)=0.9 Hz; dtd, 1.1 %, N^13^C*H*_3_, ^1^ *J*(^13^C,^1^H)=153.0 Hz, ^4^ *J*(^19^F,^1^H)=1.6 Hz, ^6^ *J*(^19^F,^1^H)=0.9 Hz) ppm. ^1^H NMR (400 MHz, SO_2_, ext. [D6]acetone, 20 °C): *δ*=5.62 (s br, 98.9 %, N^12^C*H*_3_; d br, 1.1 %, N^13^C*H*_3_, ^1^ *J*(^13^C,^1^H)=153.0 Hz) ppm. ^13^C{^19^F,^1^H} NMR (101 MHz, SO_2_, ext. [D6]acetone, 20 °C): *δ*=177.7 (*C*4), 166.1 (*C*2/*C*6), 37.7 (*C*H_3_)  ppm. ^14^N NMR (29 MHz, SO_2_, ext. [D6]acetone, 20 °C): *δ*=−167.4 (3 N/5 N), −220.0 (1 N). ^1^H,^15^N HMBC NMR (400 MHz/41 MHz, CD_2_Cl_2_, 20 °C): *δ*=4.34 ppm/−220.3 ppm. ^19^F NMR (377 MHz, CD_2_Cl_2_, 20 °C): cation: *δ*=0.9 (t br, *F*4, ^4^ *J*(^19^F,^19^F)=16.0 Hz), −26.9 (d br, *F*2/*F*6, ^4^ *J*(^19^F,^19^F)=16.0 Hz); anion: *δ*=−38.3 (m, **A**B_4_X, 1F, ^2^ *J*(^19^F,^19^F)=187.4 Hz, ^1^ *J*(^125^Te,^19^F)=3350.0 Hz), −46.0 (m, A**B_4_**X, 4F, ^2^ *J*(^19^F,^19^F)=187.4 Hz, ^1^ *J*(^125^Te,^19^F)=3462.0 Hz) ppm. ^27^Al{^19^F} NMR (104 MHz, CD_2_Cl_2_, 20 °C): *δ*=46.8 (s, 72.9 %, [Al(OTeF_5_)_4_]^−^; d, 22.2 %, [Al(OTeF_5_)_3_(O^125^TeF_5_)]^−^, ^2^ *J*(^125^Te,^27^Al)=72.9 Hz; d, 2.8 %, [Al(OTeF_5_)_3_(O^123^TeF_5_)]^−^, ^2^ *J*(^123^Te,^27^Al)=61.4 Hz; t, 2.6 %, [Al(OTeF_5_)_2_(O^125^TeF_5_)_2_]^−^, ^2^ *J*(^125^Te,^27^Al)=72.9 Hz; t, 0.04 %, [Al(OTeF_5_)_2_(O^123^TeF_5_)_2_]^−^, ^2^ *J*(^123^Te,^27^Al)=61.3 Hz) ppm. IR (ATR, 25 °C): $\tilde{\nu }$ =1687 (m), 1652 (w), 1626 (w), 1557 (m), 1537 (m), 1522 (w), 1510 (w), 1466 (m), 1440 (w), 1424 (w), 1392 (w), 1196 (m), 1128 (w), 1084 (w), 1060 (w), 933 (s, ν(Al‐O)), 817 (w), 802 (m), 689 (vs, ν(Te‐F)), 629 (m), 548 (s), 496 (w) cm^−1^.

### Reaction with of 1 CH_2_Cl_2_

A sample of 6 mL precooled dichloromethane was added to **1** (425 mg, 0.406 mmol) at −40 °C. The initially colorless suspension was allowed to slowly warm up forming a brown solution at room temperature. The ^19^F NMR spectrum showed the complete decomposition of the anion. All volatiles were condensed into a second flask, yielding CH_2_Cl(OTeF_5_) and CH_2_(OTeF_5_)_2_ in a 10:1 ratio.

**CH_2_Cl(OTeF_5_)**: ^1^H NMR (400 MHz, CH_2_Cl_2_, ext. [D6]acetone, 20 °C): *δ*=5.99 (quintet‐d, 92.8 %, ^4^ *J*(^19^F,^1^H)=2.7 Hz, ^4^ *J*(^19^F,^1^H)=0.6 Hz; d‐quintet‐d, 7.1 %, ^3^ *J*(^125^Te,^1^H)=214.7 Hz, ^4^ *J*(^19^F,^1^H)=2.7 Hz, ^4^ *J*(^19^F,^1^H)=0.6 Hz; d‐quintet‐d, 1 %, ^3^ *J*(^125^Te,^1^H)=180.2 Hz, ^4^ *J*(^19^F,^1^H)=2.7 Hz, ^4^ *J*(^19^F,^1^H)=0.6 Hz) ppm. ^1^H,^13^C HMQC NMR (400 MHz, CH_2_Cl_2_, ext. [D6]acetone, 20 °C): *δ*=5.99 ppm/77.2 ppm. ^19^F NMR (377 MHz, CH_2_Cl_2_, ext. [D6]acetone, 20 °C): *δ*=−43.7 (m, **A**B_4_X, 1F, ^2^ *J*(^19^F,^19^F)=181.2 Hz, ^4^ *J*(^19^F,^1^H)=0.6 Hz, ^1^ *J*(^125^Te,^19^F)=3502 Hz), −49.4 (m, A**B_4_**X, 1F, ^2^ *J*(^19^F,^19^F)=181.2 Hz, ^4^ *J*(^19^F,^1^H)=2.7 Hz, ^1^ *J*(^125^Te,^19^F)=3765 Hz, ^1^ *J*(^123^Te,^19^F)=3123 Hz) ppm.

**CH_2_(OTeF_5_)_2_**: ^1^H NMR (400 MHz, CH_2_Cl_2_, ext. [D6]acetone, 20 °C): *δ*=6.12 (nonet‐t, 83 %, ^4^ *J*(^19^F,^1^H)=2.5 Hz, ^4^ *J*(^19^F,^1^H)=0.4 Hz; d‐nonet‐t, 14 %, ^3^ *J*(^125^Te,^1^H)=207.0 Hz, ^4^ *J*(^19^F,^1^H)=2.5 Hz, ^4^ *J*(^19^F,^1^H)=0.4 Hz; d‐nonet‐t, 2 %, ^3^ *J*(^125^Te,^1^H)=172.0 Hz, ^4^ *J*(^19^F,^1^H)=2.5 Hz, ^4^ *J*(^19^F,^1^H)=0.4 Hz) ppm. ^19^F NMR (377 MHz, CH_2_Cl_2_, ext. [D6]acetone, 20 °C): *δ*=−44.6 (m, **A**B_4_X, 1F, ^2^ *J*(^19^F,^19^F)=181.2 Hz, ^4^ *J*(^19^F,^1^H)=0.4 Hz, ^1^ *J*(^125^Te,^19^F)=3540 Hz), −49.2 (m, A**B_4_**X, 1F, ^2^ *J*(^19^F,^19^F)=181.2 Hz, ^4^ *J*(^19^F,^1^H)=2.5 Hz, ^1^ *J*(^125^Te,^19^F)=3749 Hz, ^1^ *J*(^123^Te,^19^F)=3110 Hz) ppm.

## Conflict of interest

The authors declare no conflict of interest.

## Supporting information

As a service to our authors and readers, this journal provides supporting information supplied by the authors. Such materials are peer reviewed and may be re‐organized for online delivery, but are not copy‐edited or typeset. Technical support issues arising from supporting information (other than missing files) should be addressed to the authors.

Supplementary
