# Supplementary material for: Friedel–Crafts Type Methylation with Dimethylhalonium Salts
Source: Chemistry. 2020 Oct 5;26(63):14377–84. doi: 10.1002/chem.202001457 (PMC7702172; doi:10.1002/chem.202001457)
Supplement: Supplementary file 1 — Supplementary [file CHEM-26-14377-s001.pdf]

Chemistry–A European Journal

Supporting Information

Friedel–Crafts Type Methylation with Dimethylhalonium Salts

Sebastian Hämmerling, Patrick Voßnacker, Simon Steinhauer, Helmut Beckers, and Sebastian Riedel*[a]

Table of Contents

1 Crystallographic Data 2
2 GC/MS and simulated spectra 3
2.1 $AlCl_3$ in MeCl 3
2.2 $[NEt_4][AlCl_4]$ in MeCl 4
2.3 Methylation of oligofluorobenzenes 5
2.3.1 Methylation of 1,2,3,4-tetrafluorobenzene – 3 hours reaction time 5
2.3.2 Methylation of 1,2,3-trifluorobenzene 9
2.3.3 Methylation of 1,2-difluorobenzene 17
2.4 $[MeNC_5F_4I][Al(OTeF_5)_4]$ (2I) 23
2.5 $[MeNC_5F_5][Al(OTeF_5)_4]$ (2F) 24
3 Quantum Chemical Calculations 25
3.1 Reaction path for methylation of *o*DFB with $AlCl_3$/MeCl 25
3.2 Reaction path for methylation with $[Me_2X]^+$ 27
3.3 Fluoride ion affinity of $[MeN_3C_3F_{3-x}(OTeF_5)_x]^+$ 29
3.4 Optimized structures for reaction paths in the $AlCl_3$ system – COSMO MeCl 30
3.5 Optimized structures for reaction paths in the $[Me_2X]^+$ system – COSMO SO_2 42
3.6 Optimized structures for methyl cation affinities 75
3.7 Optimized structures for fluoride ion affinities 122
4 References 133

1 Crystallographic Data

Compound	[MeNC$_5$F$_4$I][Al(OTeF$_5$)$_4$]	[MeN$_3$C$_3$F(OTeF$_5$)$_2$][Al(OTeF$_5$)$_4$)]
CCDC	1971172	1971173
Formula	$C_6H_3AlF_{24}INO_4Te_4$	$C_4H_3AlF_{31}N_3O_6Te_6$
$D_{calc.}$/ g cm^{-3}	3.264	3.178
μ/mm^{-1}	5.875	5.494
Formula Weight	1273.37	1570.67
Color	colorless	colorless
Shape	block	block
Size/mm^3	0.179·0.125·0.121	0.52·0.323·0.163
T/K	104	100
Crystal System	triclinic	monoclinic
Space Group	$P\bar{1}$	$P2_1/c$
a/Å	9.0178(5)	13.3684(4)
b/Å	10.2547(6)	16.7755(5)
c/Å	14.8980(8)	15.7156(5)
α/°	72.621(2)	90
β/°	89.768(2)	111.357(1)
γ/°	80.618(2)	90
V/Å^3	1295.75(13)	3282.38(17)
Z	2	4
Radiation	Mo-K$_\alpha$ (0.71073 Å)	Mo-K$_\alpha$ (0.71073 Å)
Θ_{min}/°	2.645	2.666
Θ_{max}/°	31.000	25.355
Measured Refl.	72062	37114
Independent Refl.	8192	6023
Refl. (I > 2σ(I))	7144	5577
R_{int}	0.0344	0.0297
Parameters	371	461
Restraints	0	0
Largest Peak	0.646	2.985
Deepest Hole	−1.029	−2.039
GooF	1.137	1.082
wR_2 (all data)	0.0381	0.0865
wR_2	0.0365	0.0842
R_1 (all data)	0.0307	0.0385
R_1	0.0209	0.0353

2 GC/MS and simulated spectra

2.1 $AlCl_3$ in MeCl

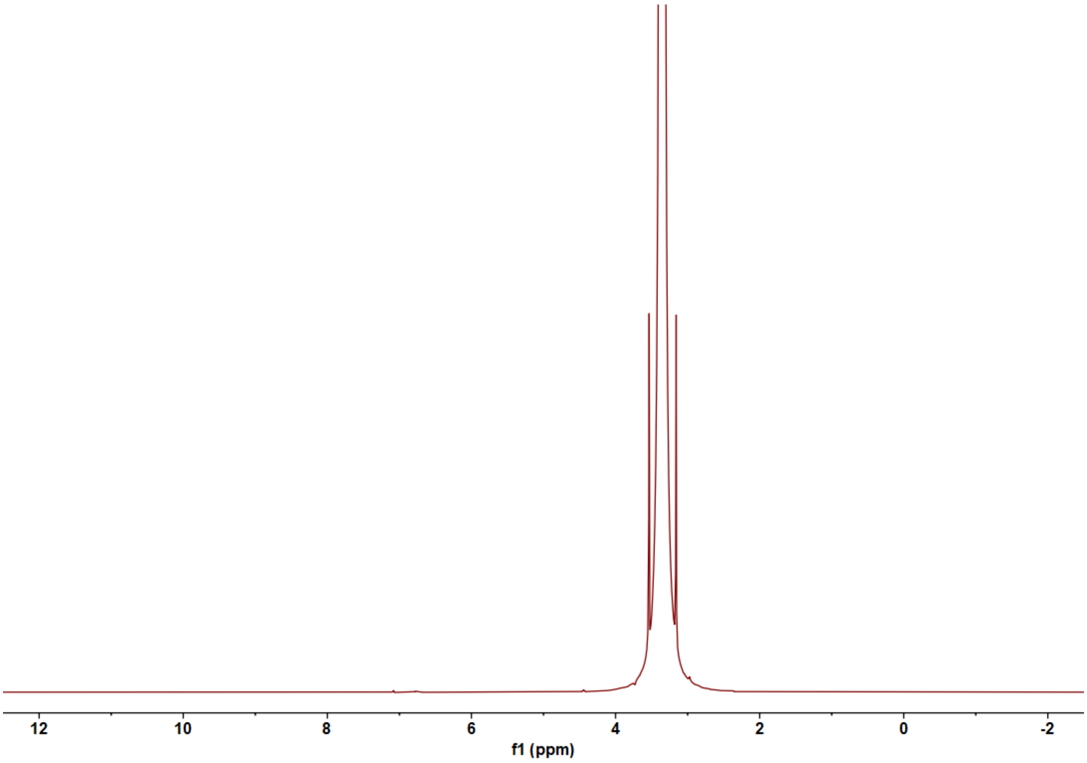

Figure S1: ^{1}H NMR spectrum (400 MHz, MeCl, no lock, r.t.) of $AlCl_3$ in MeCl.

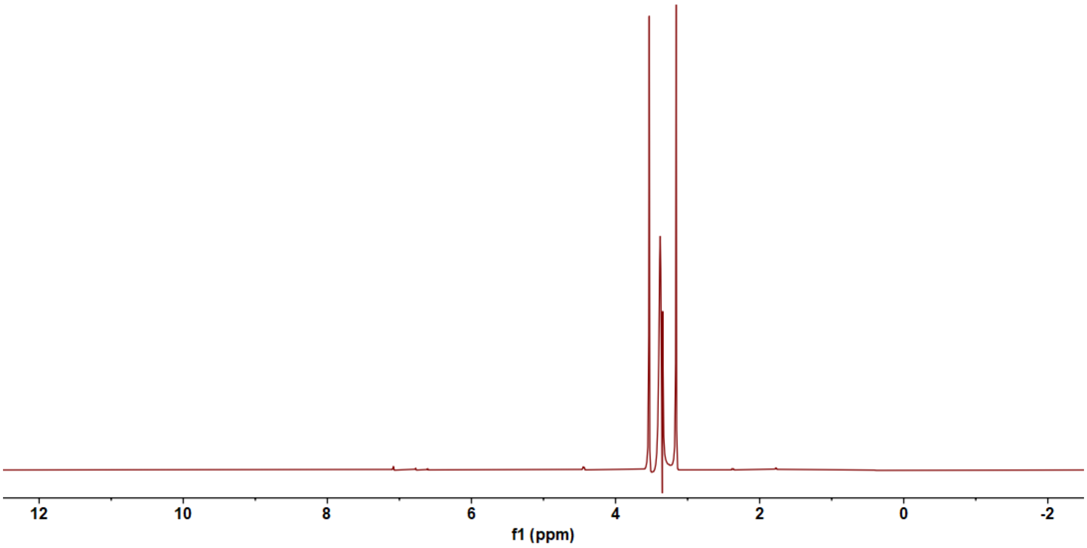

Figure S2: ^{1}H NMR spectrum (400 MHz, MeCl, no lock, r.t.) of $AlCl_3$ in MeCl with presaturation showing the absence of a $[Me_2Cl]^+$ resonance.

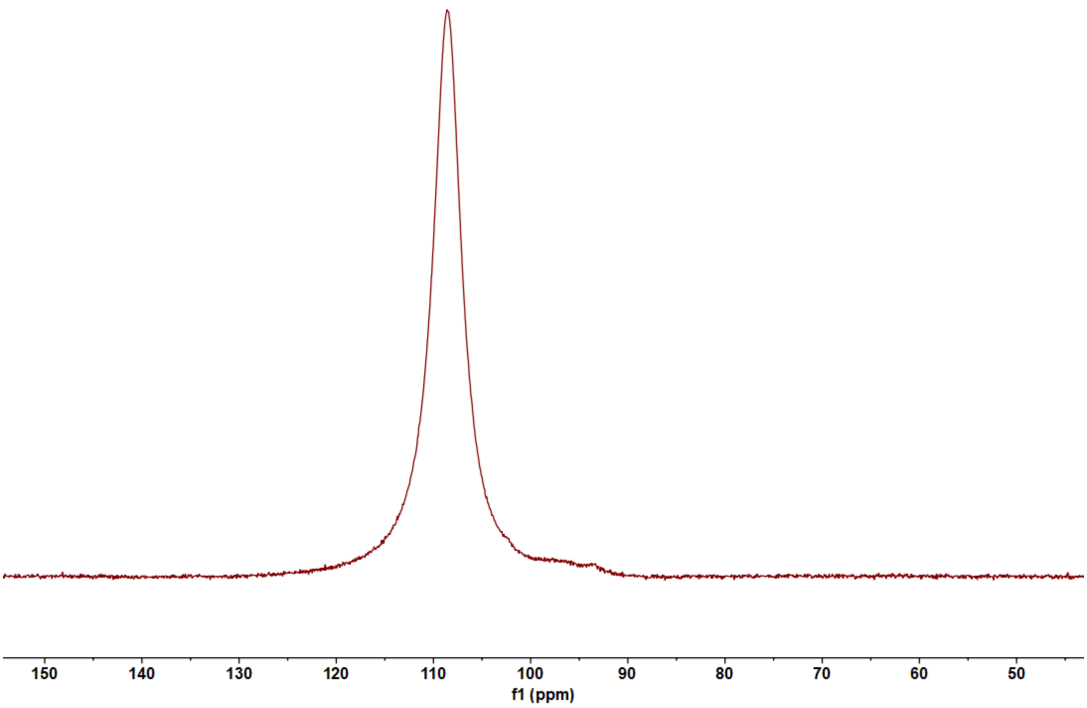

Figure S3: ^{27}Al NMR spectrum (104 MHz, MeCl, no lock, r.t.) of $AlCl_3$ in MeCl.

2.2 $[NEt_4][AlCl_4]$ in MeCl

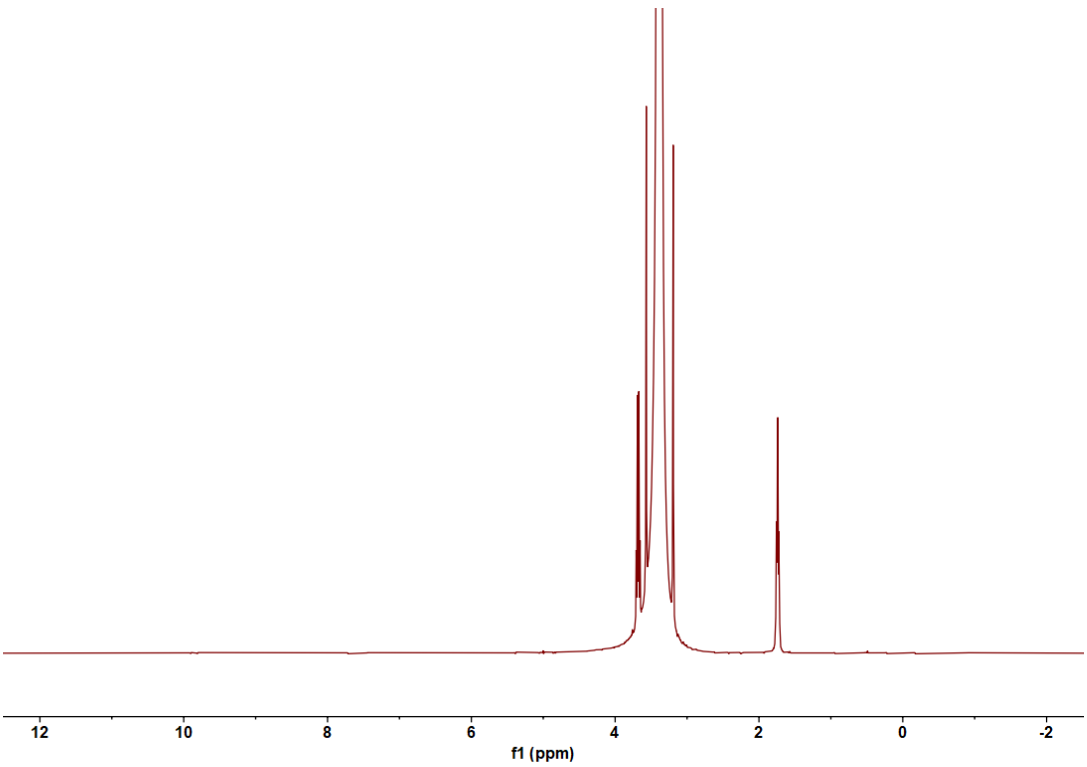

Figure S4: ^{1}H NMR spectrum (400 MHz, MeCl, no lock, gradient shim on MeCl resonance, r.t.) of $[NEt_4][AlCl_4]$ in MeCl.

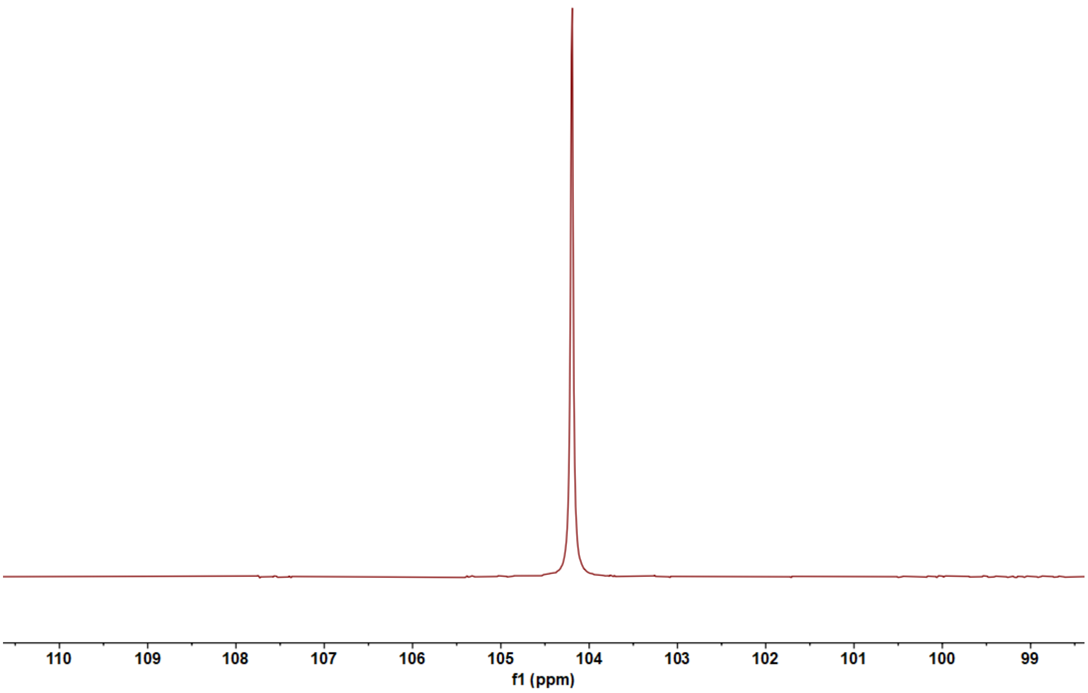

Figure S5: ^{27}Al NMR spectrum (104 MHz, MeCl, no lock, gradient shim on MeCl resonance, r.t.) of $[NEt_4][AlCl_4]$ in MeCl.

2.3 Methylation of oligofluorobenzenes

2.3.1 Methylation of 1,2,3,4-tetrafluorobenzene – 3 hours reaction time

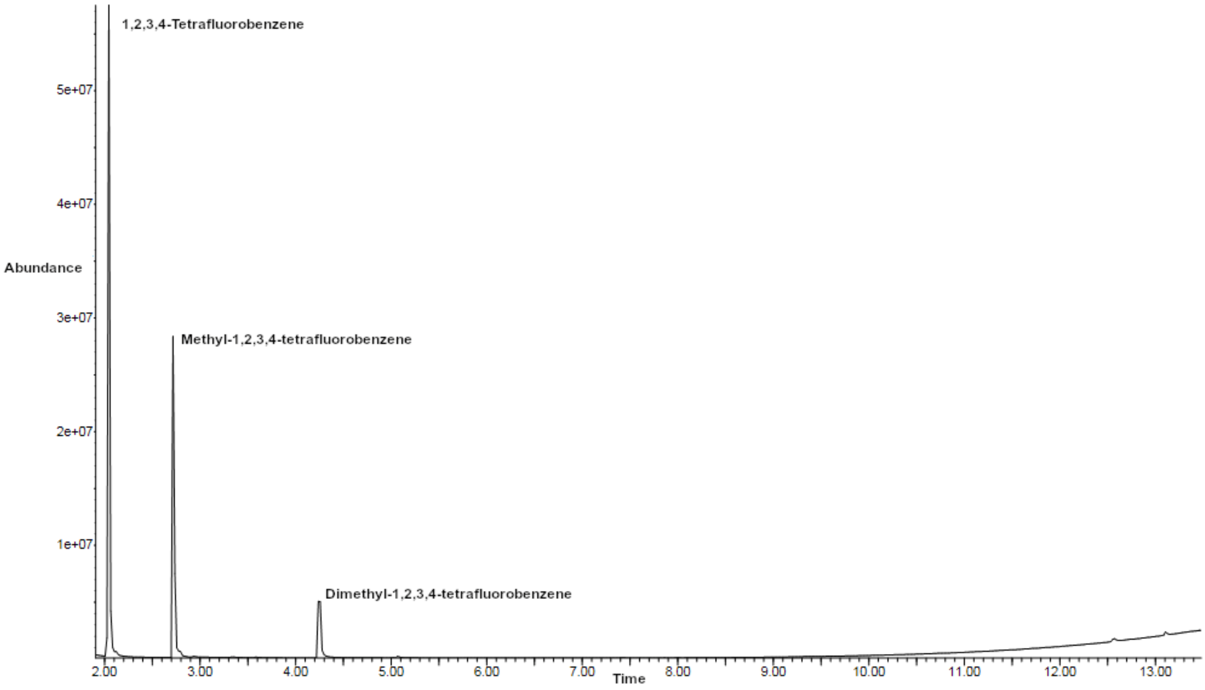

Figure S1: GC/MS TIC of reaction products.

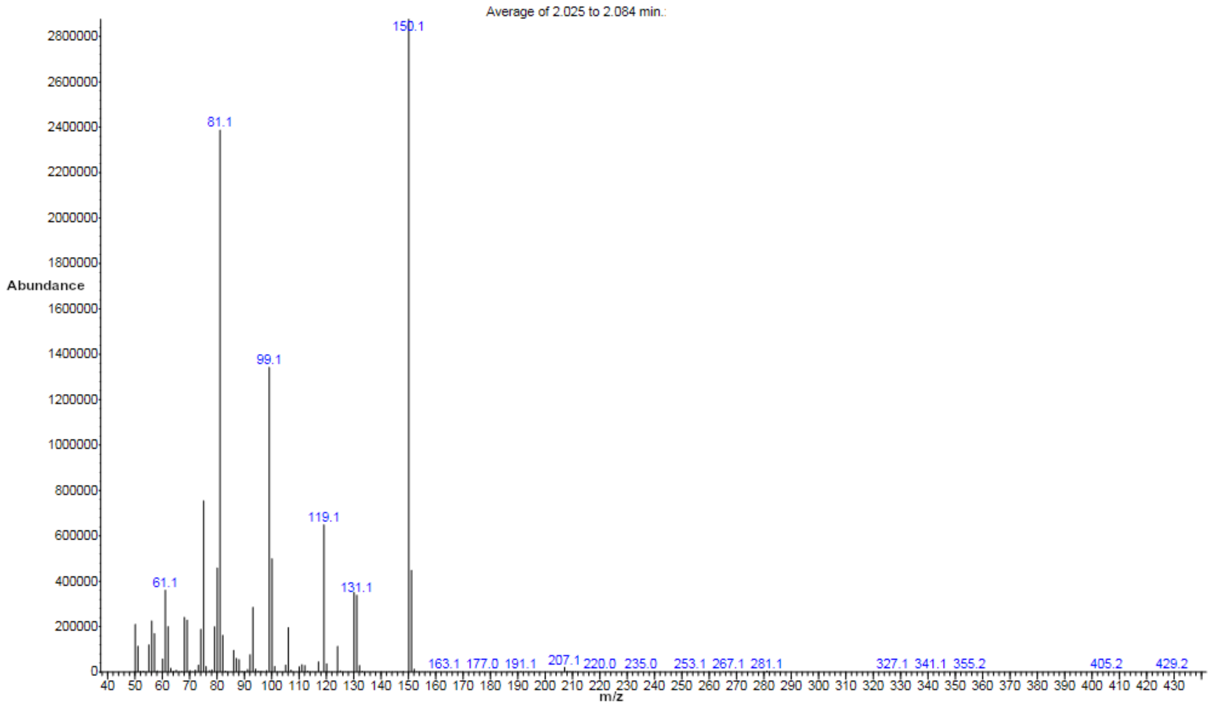

Figure S2: Extracted mass spectrum of 1,2,3,4-tetrafluorobenzene.

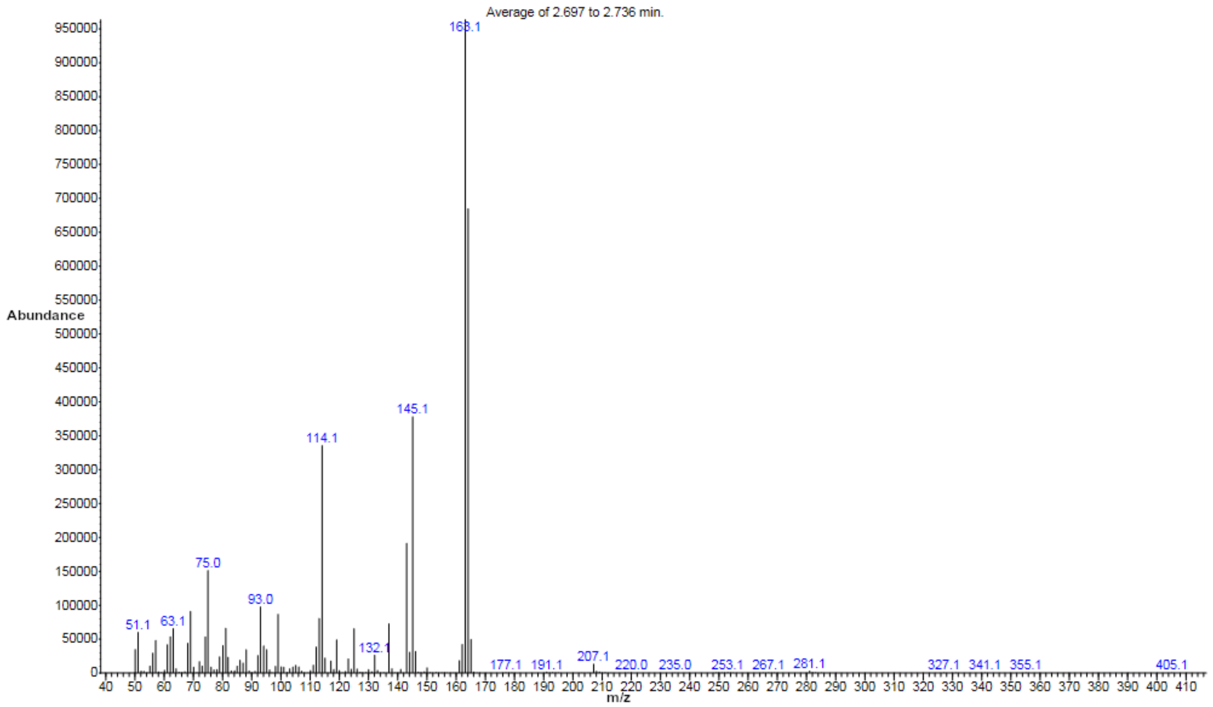

Figure S3: Extracted mass spectrum of methyl-1,2,3,4-tetrafluorobenzene.

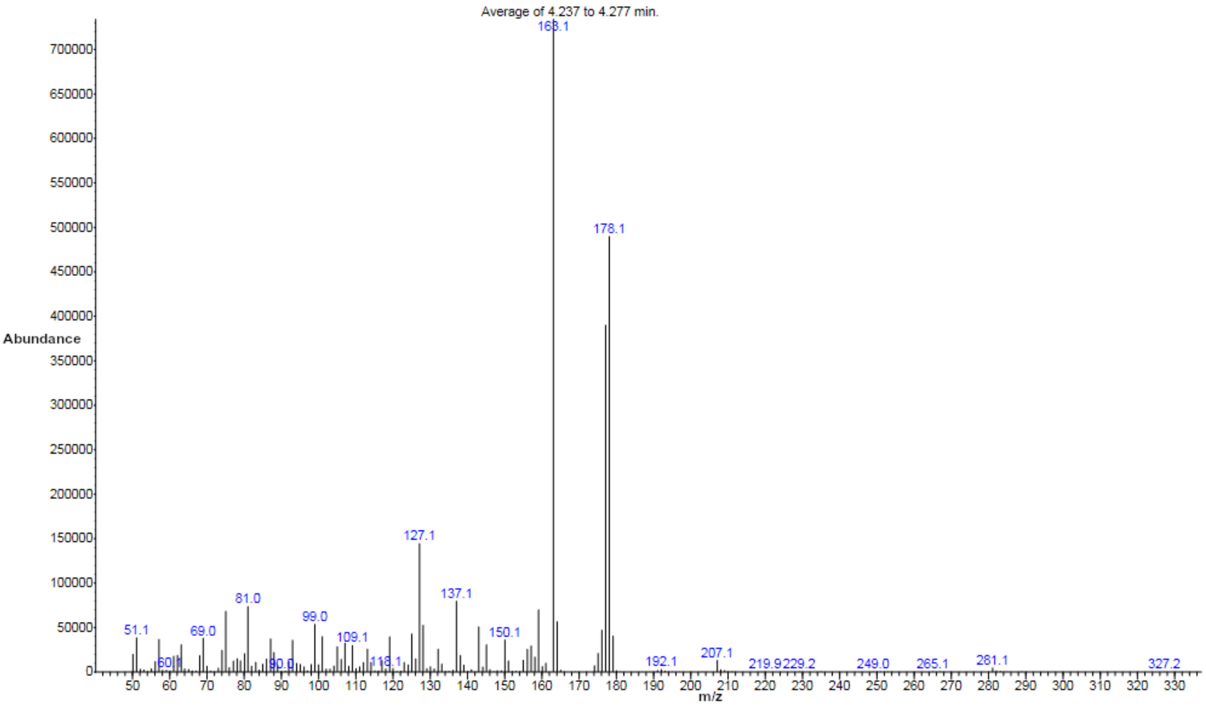

Figure S4: Extracted mass spectrum of dimethyl-1,2,3,4-tetrafluorobenzene.

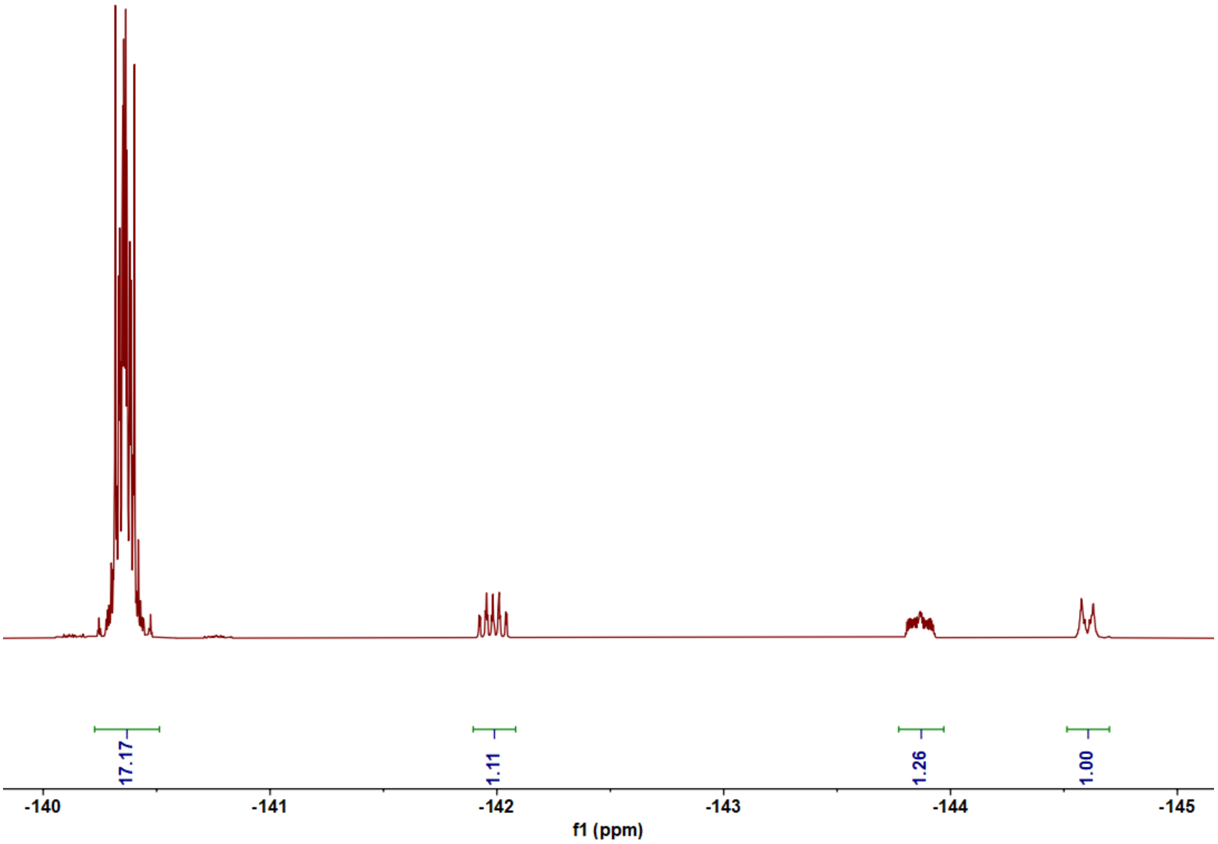

Figure S5: Section of ^{19}F NMR spectrum (377 MHz, CD_2Cl_2, r.t.) of product mixture.

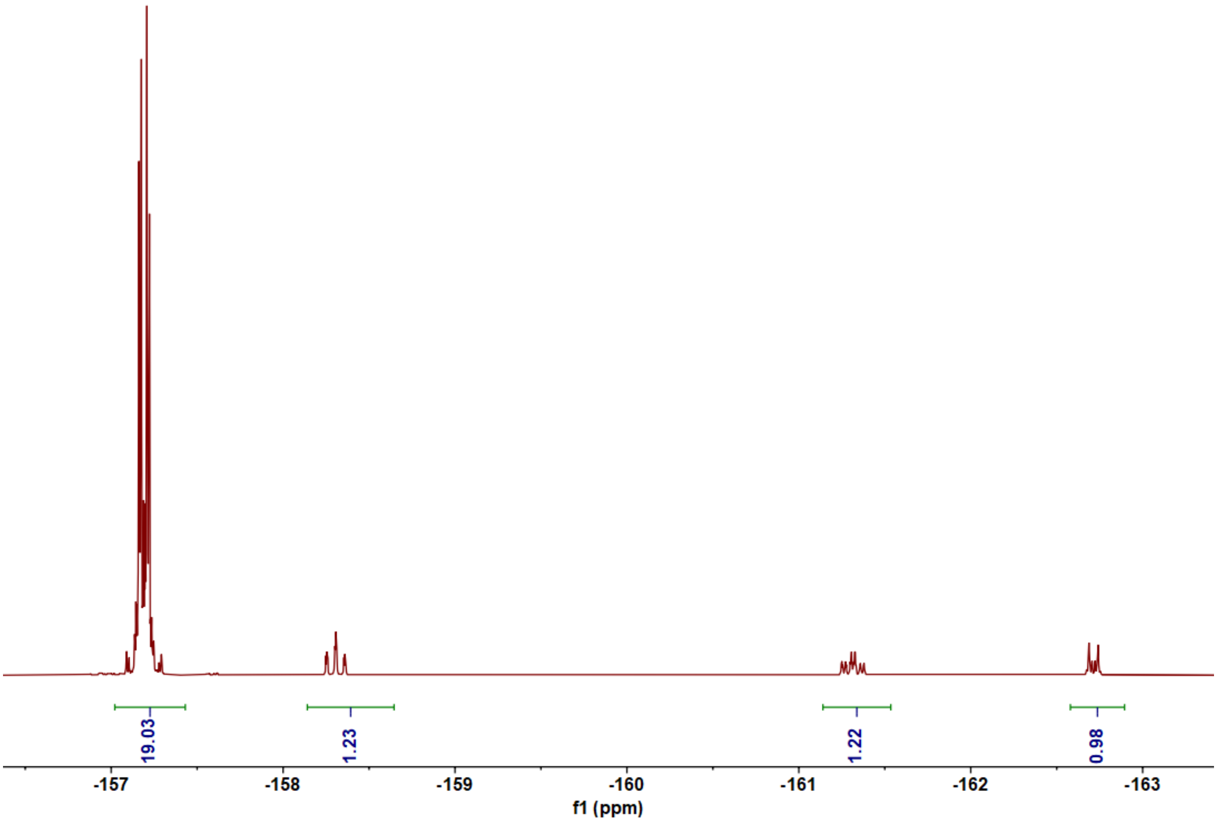

Figure S6: Section of ^{19}F NMR spectrum (377 MHz, CD_2Cl_2, r.t.) of product mixture.

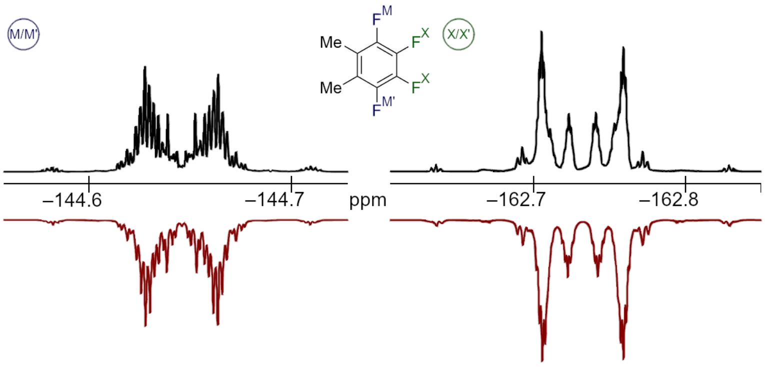

Figure S7: Experimental (top; 377 MHz, CD_2Cl_2, r.t.) and iterated (bottom) ^{19}F NMR resonances of dimethyl-1,2,3,4-tetrafluorobenzene.

2.3.2 Methylation of 1,2,3-trifluorobenzene

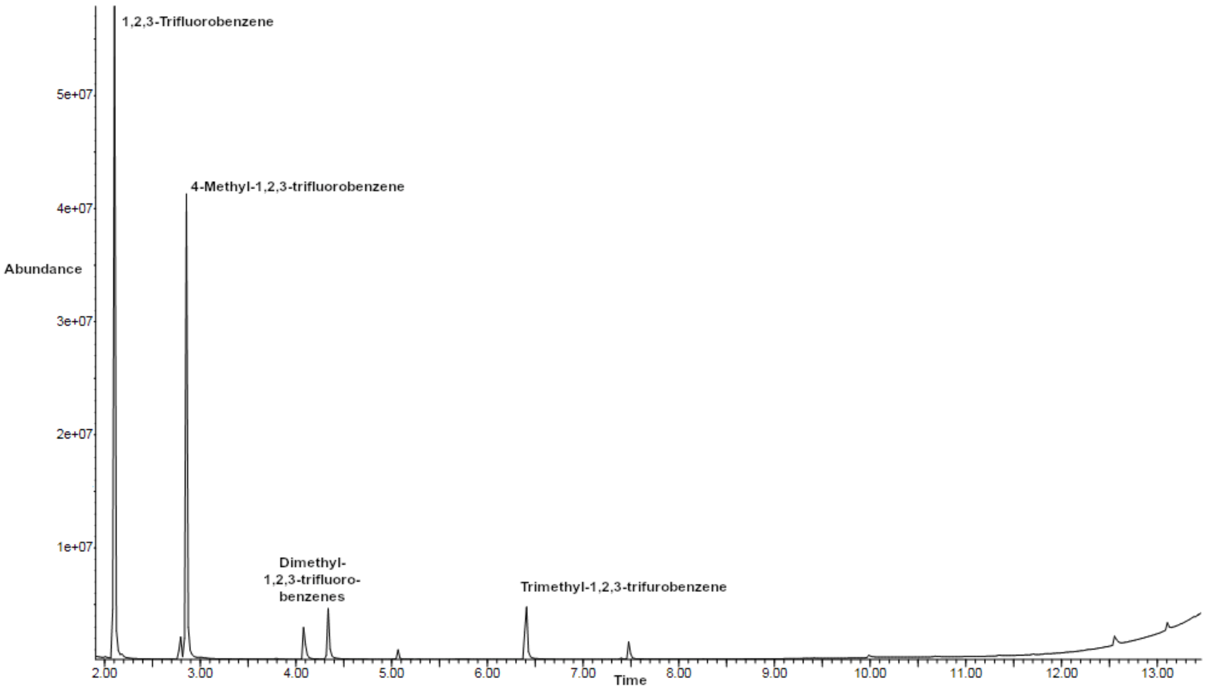

Figure S8: GC/MS TIC of reaction products.

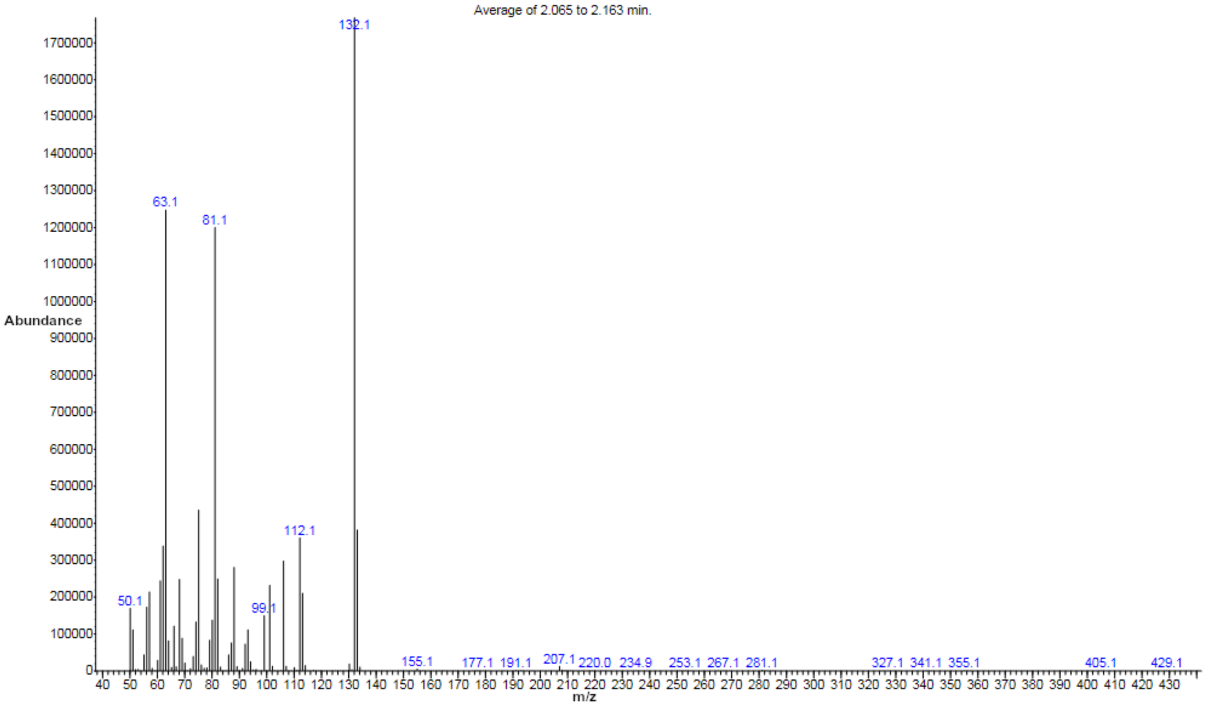

Figure S9: Extracted mass spectrum of 1,2,3-trifluorobenzene.

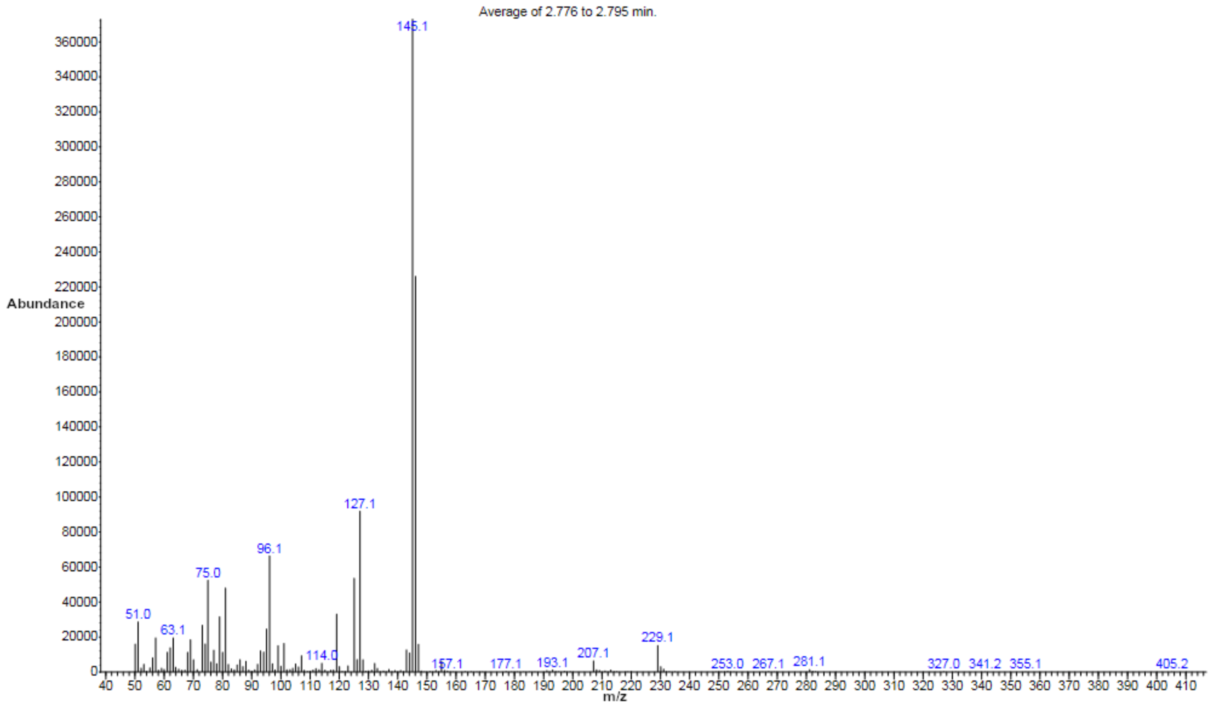

Figure S10: Extracted mass spectrum of 5-methyl-1,2,3-trifluorobenzene.

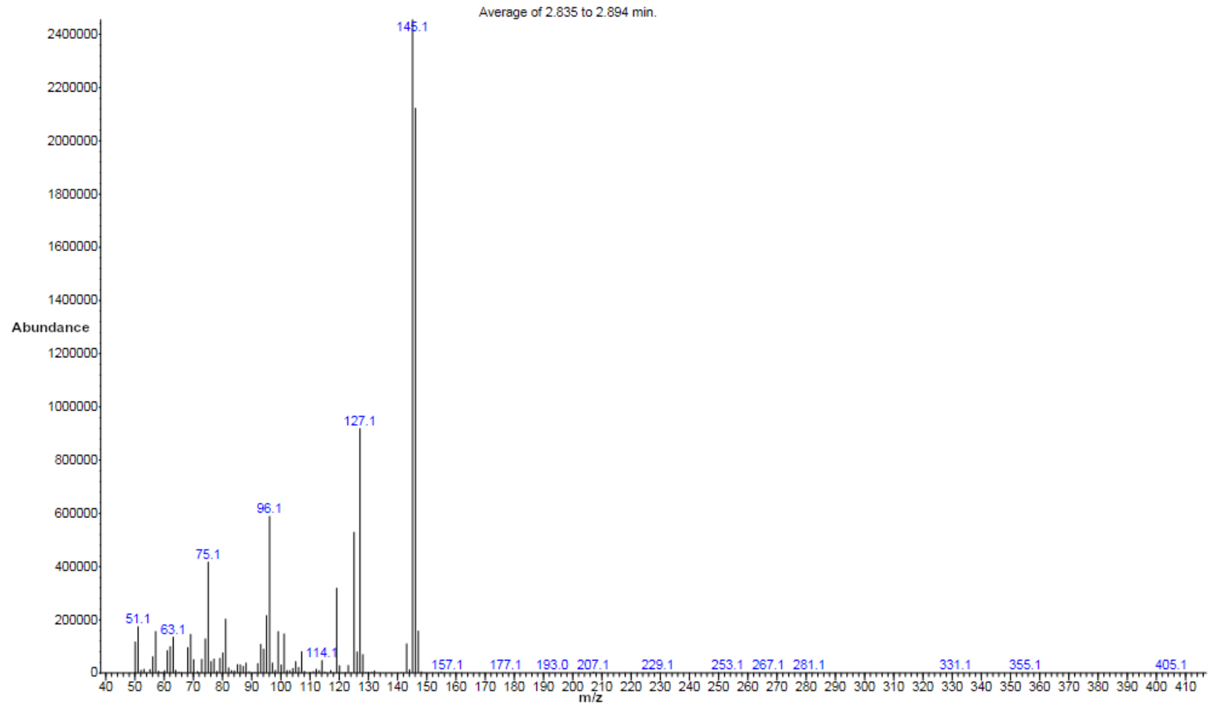

Figure S11: Extracted mass spectrum of 4-methyl-1,2,3-trifluorobenzene.

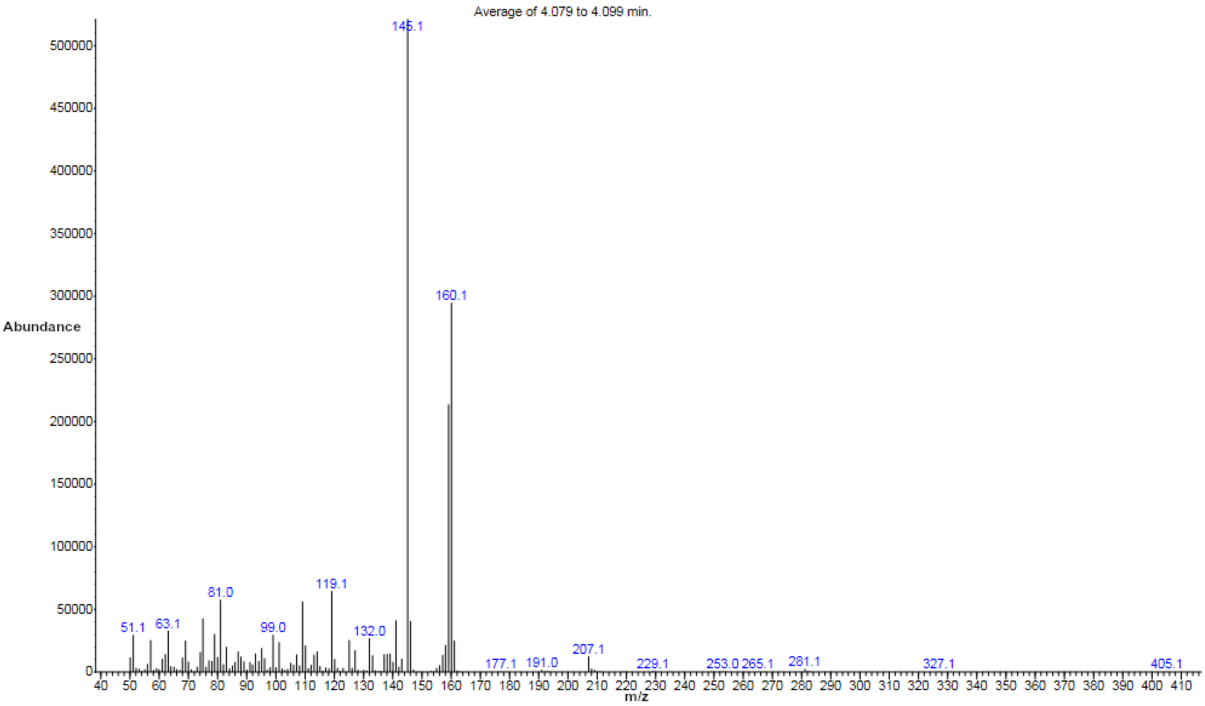

Figure S12: Extracted mass spectrum of 4,6-dimethyl-1,2,3-trifluorobenzene.

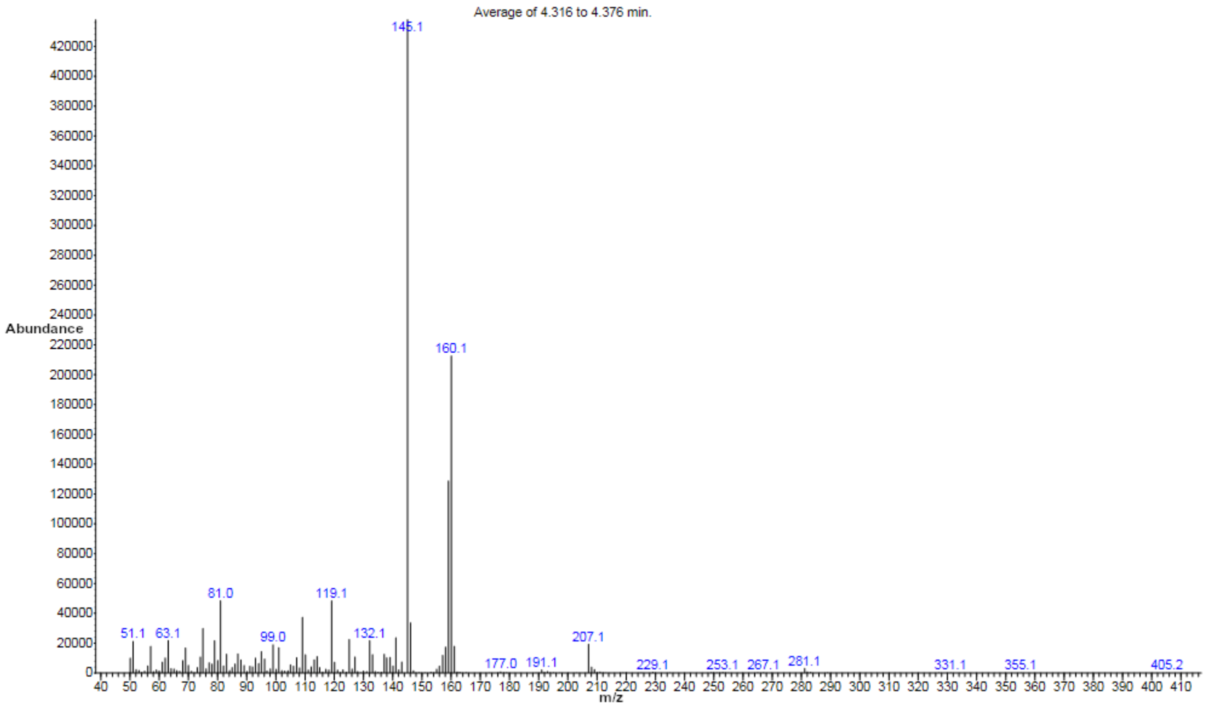

Figure S13: Extracted mass spectrum of 4,5-dimethyl-1,2,3-trifluorobenzene.

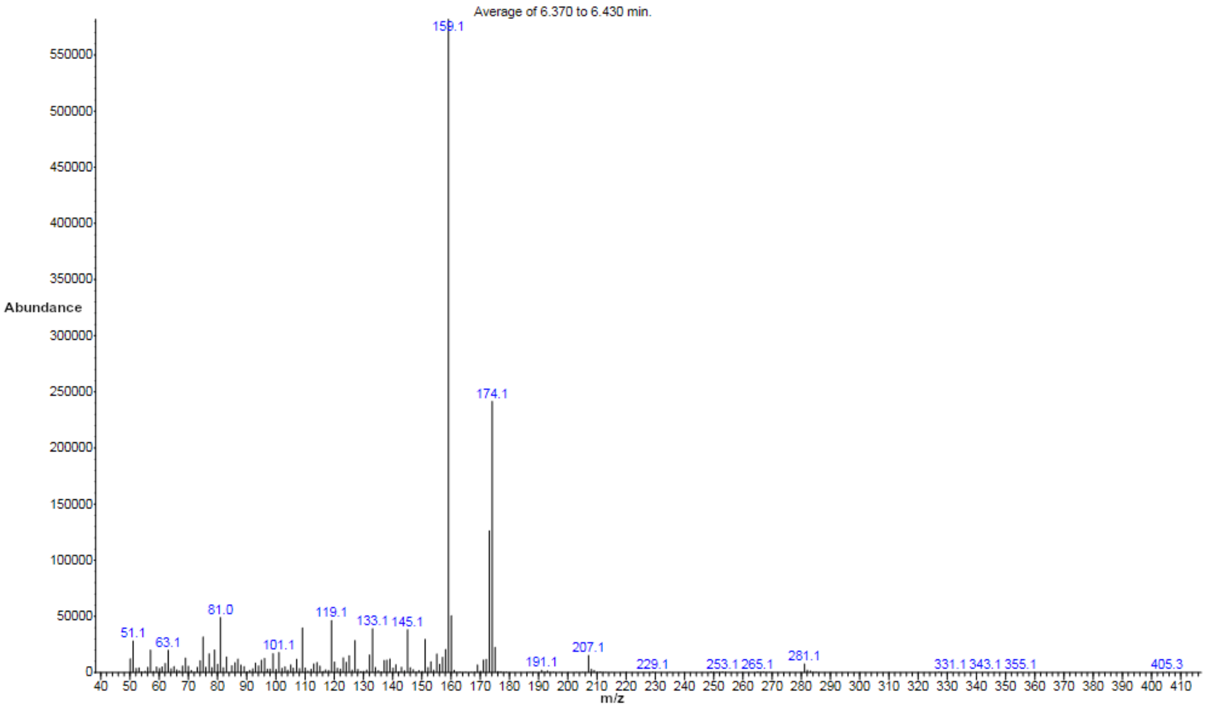

Figure S14: Extracted mass spectrum of trimethyl-1,2,3-trifluorobenzene.

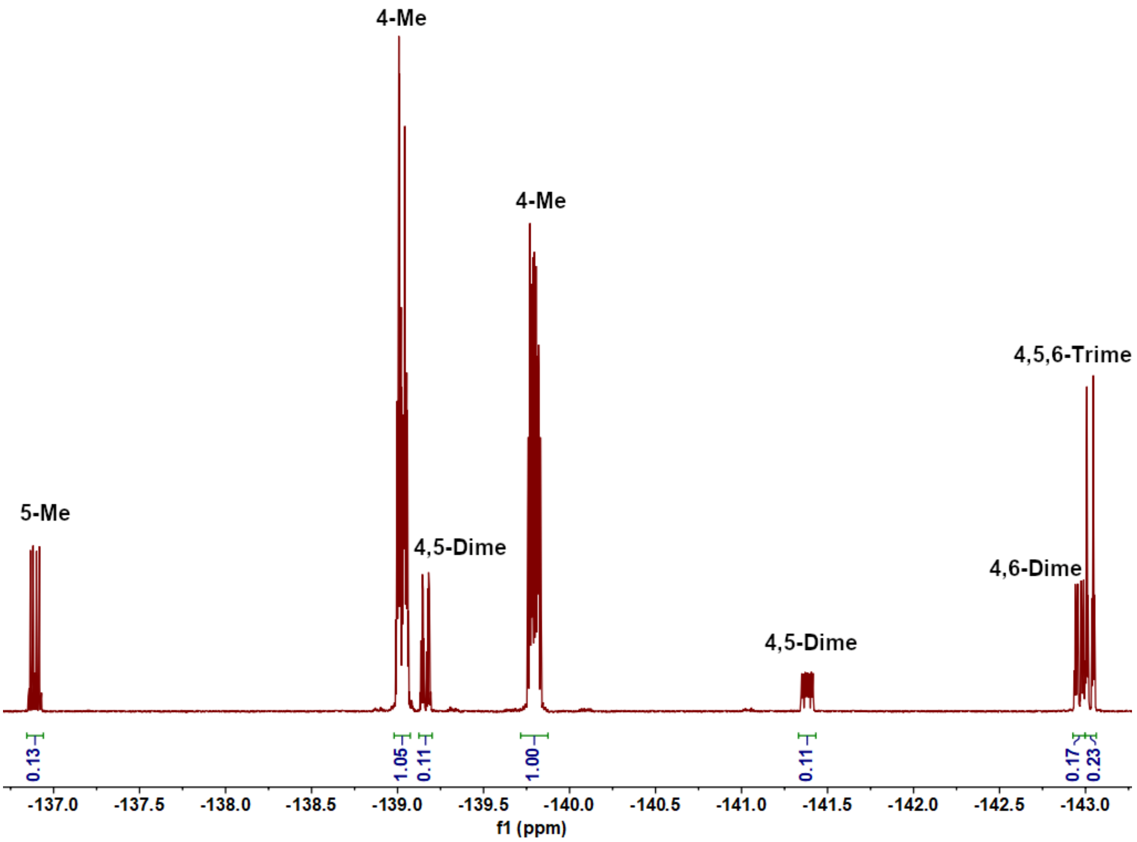

Figure S15: Section of ^{19}F NMR spectrum (565 MHz, CD_2Cl_2, r.t.) of product mixture.

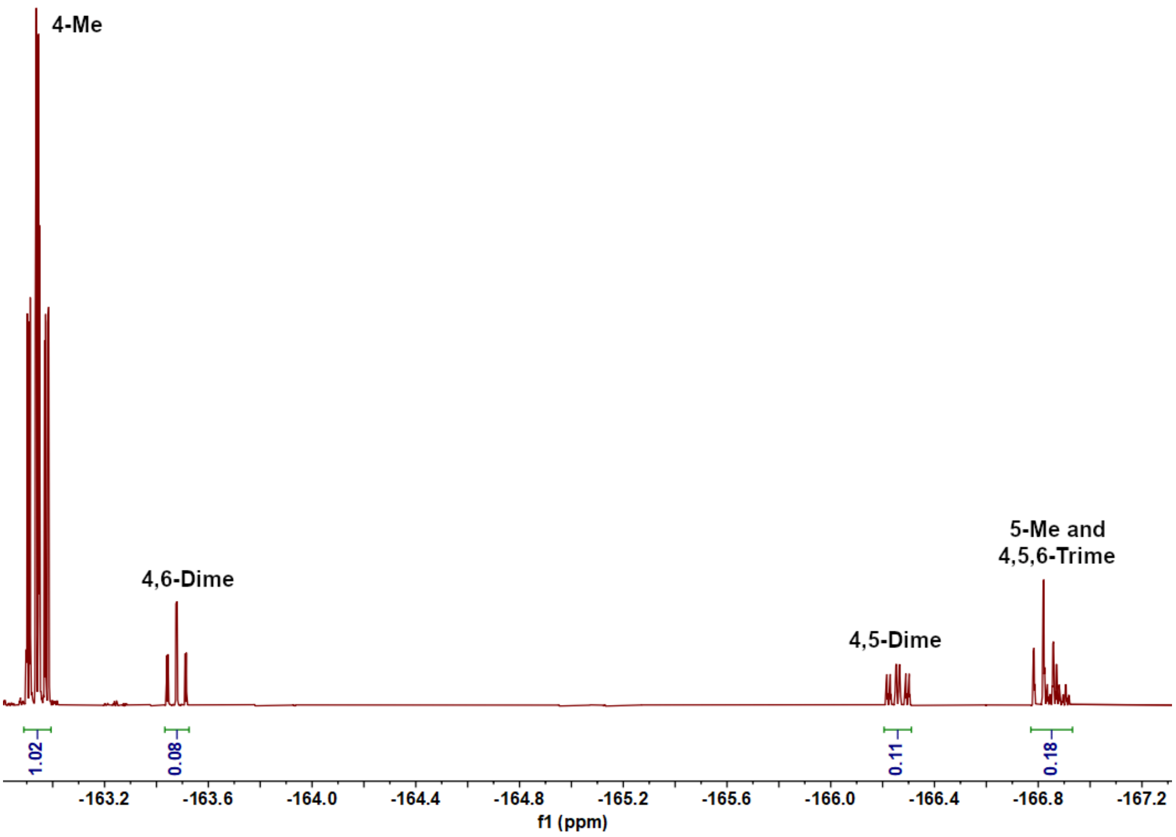

Figure S16: Section of ^{19}F NMR spectrum (565 MHz, CD_2Cl_2, r.t.) of product mixture.

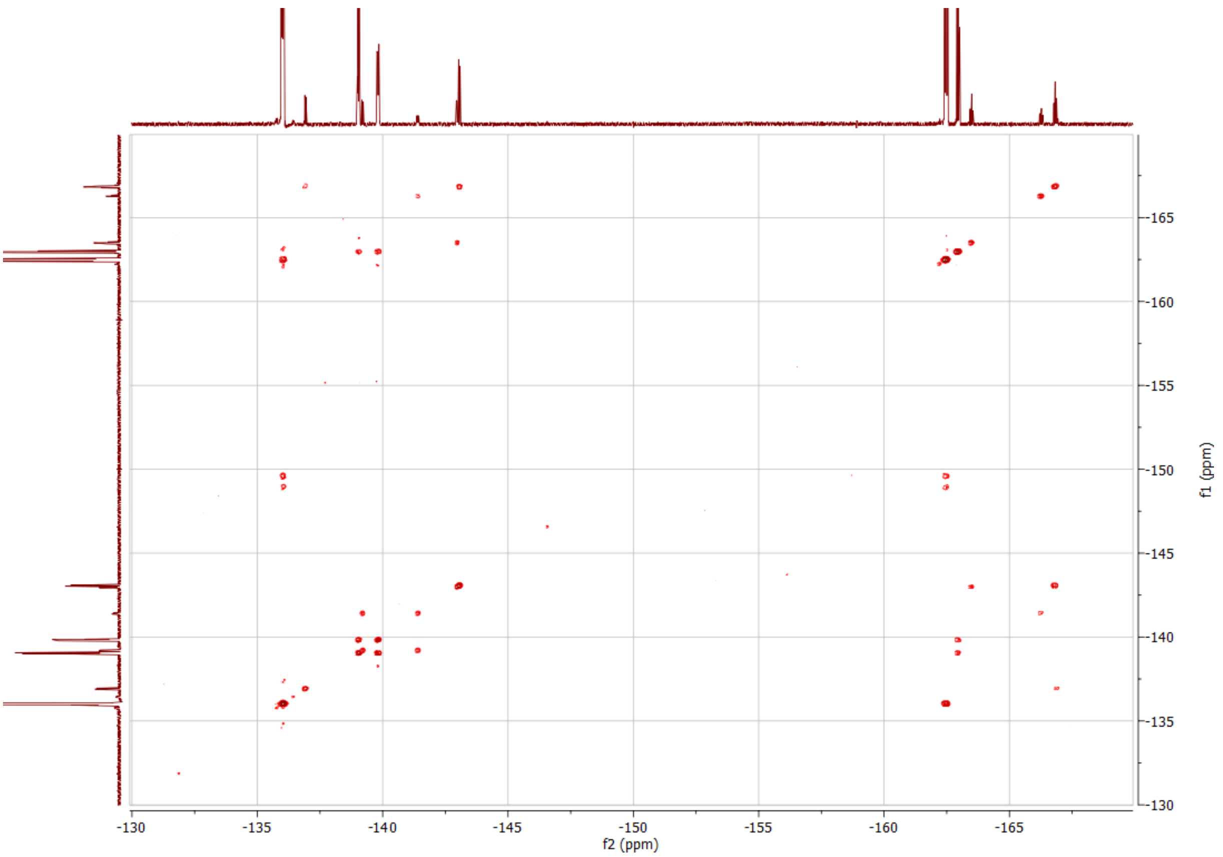

Figure S17: ^{19}F,^{19}F COSY NMR spectrum (377 MHz, CD_2Cl_2, r.t.) of product mixture.

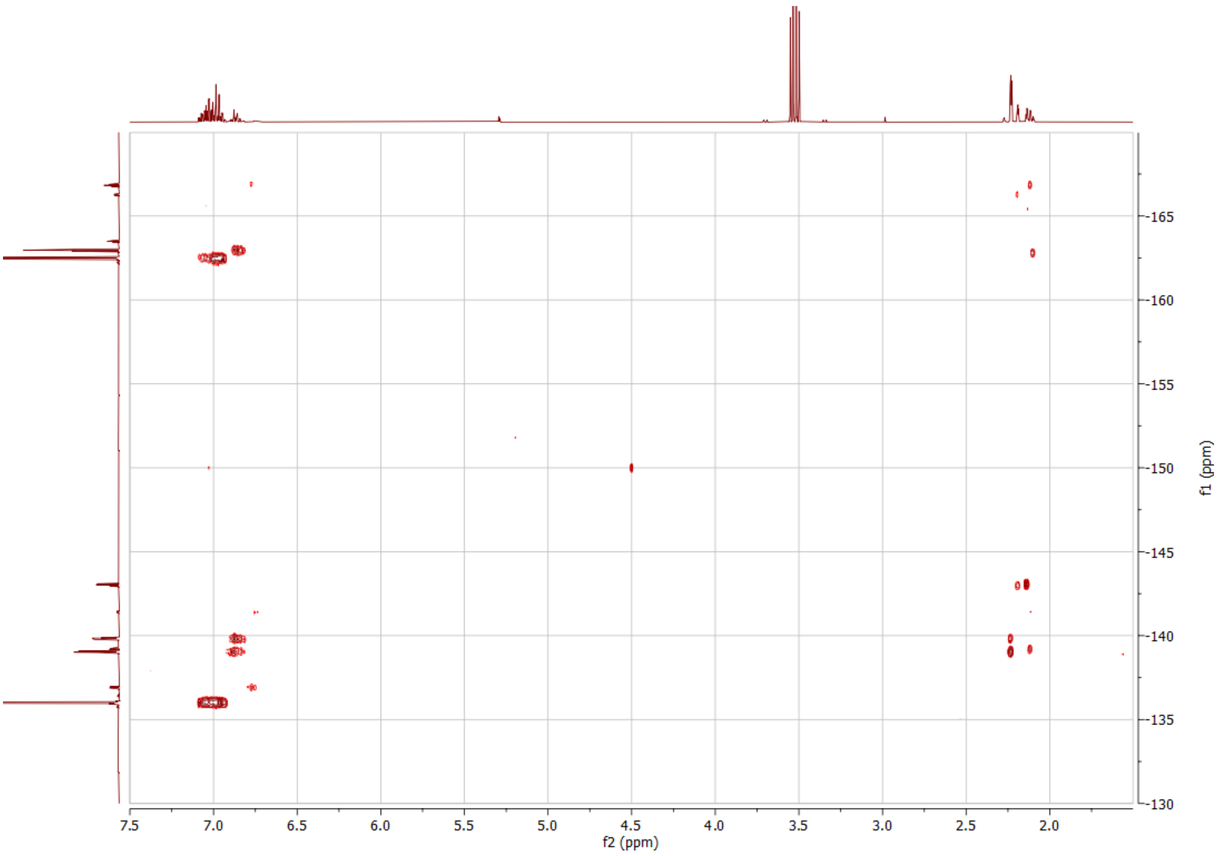

Figure S18: ^{1}H,^{19}F HETCOR NMR spectrum (400 MHz/377 MHz, CD_2Cl_2, r.t.) of product mixture.

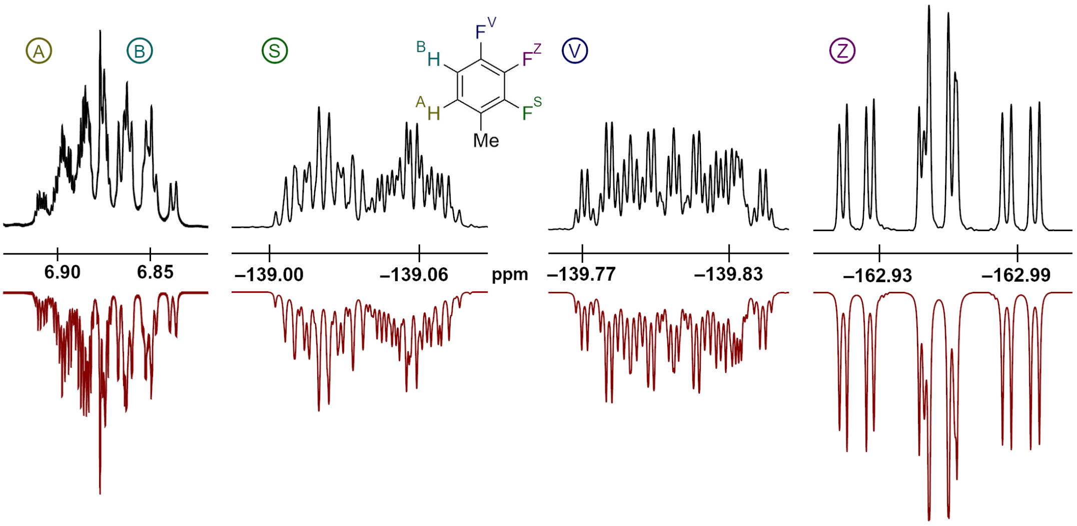

Figure S19: Experimental (top) and iterated (bottom) ^{1}H NMR (700 MHz, CD_2Cl_2, r.t.) and ^{19}F NMR (565 MHz, CD_2Cl_2, r.t.) resonances of 4-methyl-1,2,3-trifluorobenzene.

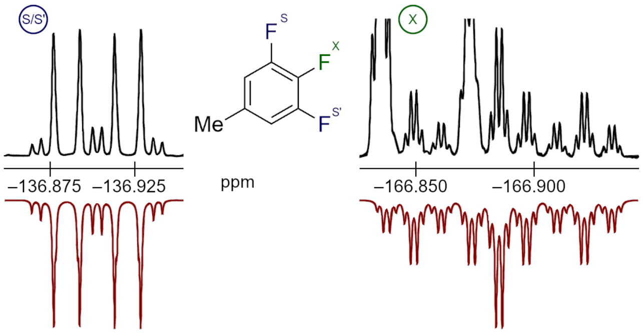

Figure S20: Experimental (top; 565 MHz, CD_2Cl_2, r.t.) and iterated (bottom) ^{19}F NMR resonances of 5-methyl-1,2,3-trifluorobenzene (partially overlapped by low-field resonance of trimethyl-1,2,3-trifluorobenzene). AA'M_3SS'X spin system: $\delta_{S/S'}$ = −136.9 (F1/F3) ppm, δ_X = −166.9 (F2) ppm, $\delta_{A/A'}$ = 6.77 (H6/H4) ppm, δ_M (CH_3) not observable due to overlap/low intensity, $J_{SS'}$ = $^4J(^{19}F,^{19}F)$ = 6.00 Hz, J_{SX} = $J_{S'X}$ = $^3J(^{19}F,^{19}F)$ = −20.34 Hz, J_{SA} = $J_{S'A'}$ = $^3J(^{19}F,^1H)$ = 10.77 Hz, $J_{S'A}$ = $J_{SA'}$ = $^5J(^{19}F,^1H)$ = −2.11 Hz, J_{XA} = $J_{XA'}$ = $^4J(^{19}F,^1H)$ = 6.52 Hz, $J_{AA'}$ = $^4J(^1H,^1H)$ = 2.09 Hz, J_{XM} = $^6J(^{19}F,^1H)$ = 1.50 Hz.

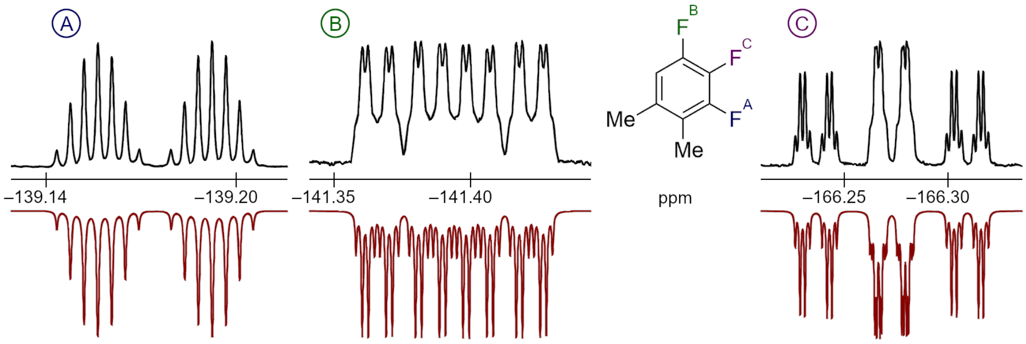

Figure S21: Experimental (top; 565 MHz, CD_2Cl_2, r.t.) and iterated (bottom) ^{19}F NMR resonances of 4,5-dimethyl-1,2,3-trifluorobenzene. $\delta(^{19}F)$ = −139.2 (dddq, 1F, *F*3, $^3J(^{19}F,^{19}F)$ = −20.88 Hz, $^4J(^{19}F,^{19}F)$ = 4.95 Hz, $^4J(^{19}F,^1H)$ = 2.38 Hz, $^5J(^{19}F,^1H)$ = 2.49 Hz (H6)), −141.4 (dddq, 1F, *F*1, $^3J(^{19}F,^{19}F)$ = −20.36 Hz, $^4J(^{19}F,^{19}F)$ = 4.95 Hz, $^3J(^{19}F,^1H)$ = 11.03 Hz, $^6J(^{19}F,^1H)$ = 1.28 Hz), −166.3 (dddq, 1F, *F*2, $^3J(^{19}F,^{19}F)$ = −20.88 Hz (F3), $^3J(^{19}F,^{19}F)$ = −20.36 Hz (F1), $^4J(^{19}F,^1H)$ = 7.32 Hz, $^6J(^{19}F,^1H)$ = 1.34 Hz) ppm. $\delta(^1H$, from $^1H,^{19}F$-HETCOR 400 MHz/377 MHz, CD_2Cl_2, r.t.) = 6.76 (Ar*H*), 2.20 (5-CH_3), 2.11 (4-CH_3) ppm.

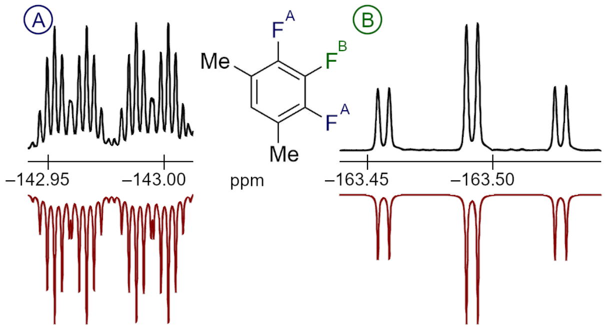

Figure S22: Experimental (top; 565 MHz, CD_2Cl_2, r.t.) and iterated (bottom) ^{19}F NMR resonances of 4,6-dimethyl-1,2,3-trifluorobenzene. $\delta(^{19}\text{F})$ = −143.0 (ddsept, 2F, *F*1/*F*3, $^3J(^{19}\text{F},^{19}\text{F})$ = −19.95, $^4J(^{19}\text{F},^{1}\text{H})$ = 7.81 Hz (5*H*), $^4J(^{19}\text{F},^{1}\text{H})$ = 1.80 Hz (C*H*3)), −163.5 (dd, 1H, F2, $^3J(^{19}\text{F},^{19}\text{F})$ = −19.95, $^5J(^{19}\text{F},^{1}\text{H})$ = 2.55 Hz) ppm. $\delta(^{1}\text{H}$, from ^{1}H,^{19}F-HETCOR 400 MHz/377 MHz, CD_2Cl_2, r.t.) = 2.19 (C*H*3) ppm. Ar*H* not observable due to overlap/low intensity.

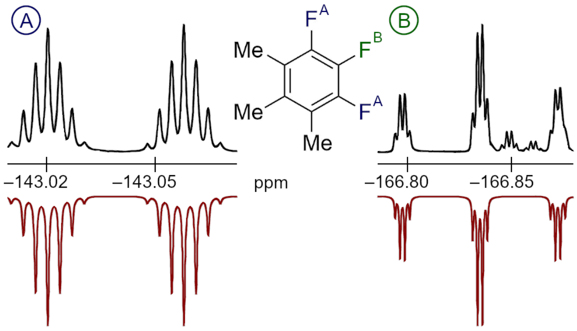

Figure S23: Experimental (top; 565 MHz, CD_2Cl_2, r.t.) and iterated (bottom) ^{19}F NMR resonances of trimethyl-1,2,3-trifluorobenzene (partially overlapped by low-field resonance of 5-methyl-1,2,3-trifluoro-benzene): $\delta(^{19}\text{F})$ = −143.0 (dsept, 2F, *F*1/*F*3, $^3J(^{19}\text{F},^{19}\text{F})$ = −21.17 Hz, $^4J(^{19}\text{F},^{1}\text{H})$ = 1.89 Hz), −166.8 (tq, 1F, *F*2, $^3J(^{19}\text{F},^{19}\text{F})$ = −21.17 Hz, $^4J(^{19}\text{F},^{1}\text{H})$ = 1.33 Hz). $\delta(^{1}\text{H}$, from ^{1}H,^{19}F-HETCOR 400 MHz/377 MHz, CD_2Cl_2, r.t.) = 2.14 (4-C*H*3/6-C*H*3), 2.12 (5-C*H*3).

2.3.3 Methylation of 1,2-difluorobenzene

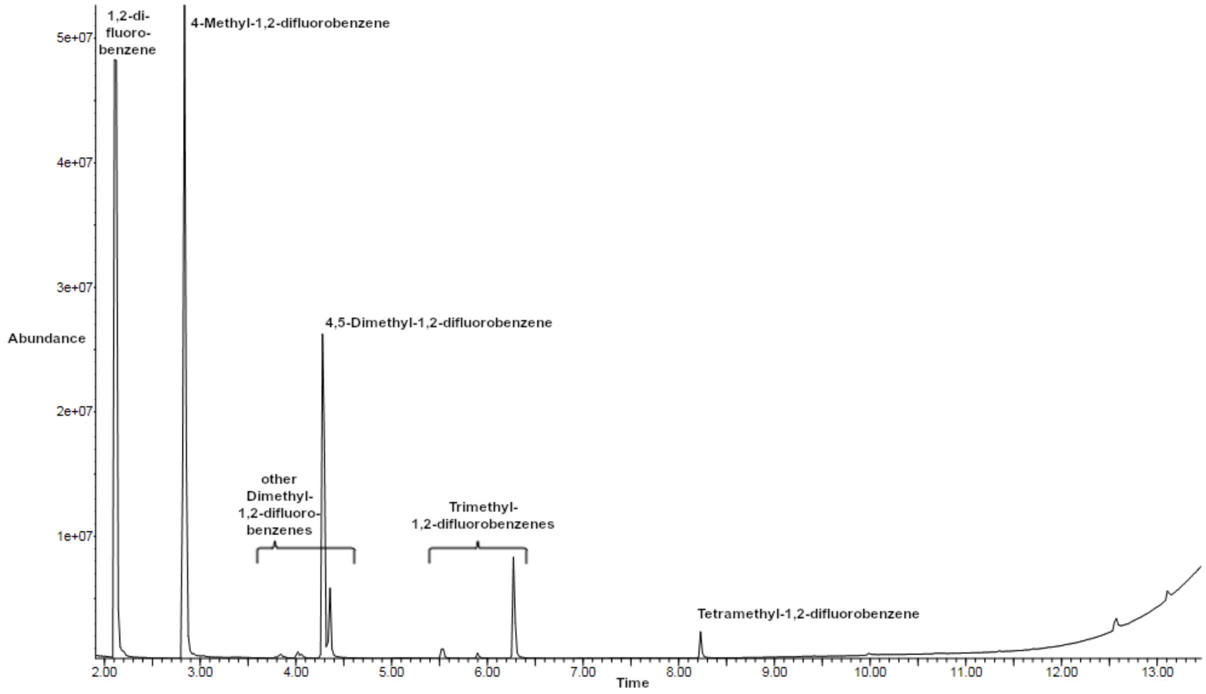

Figure S24: GC/MS TIC of reaction products.

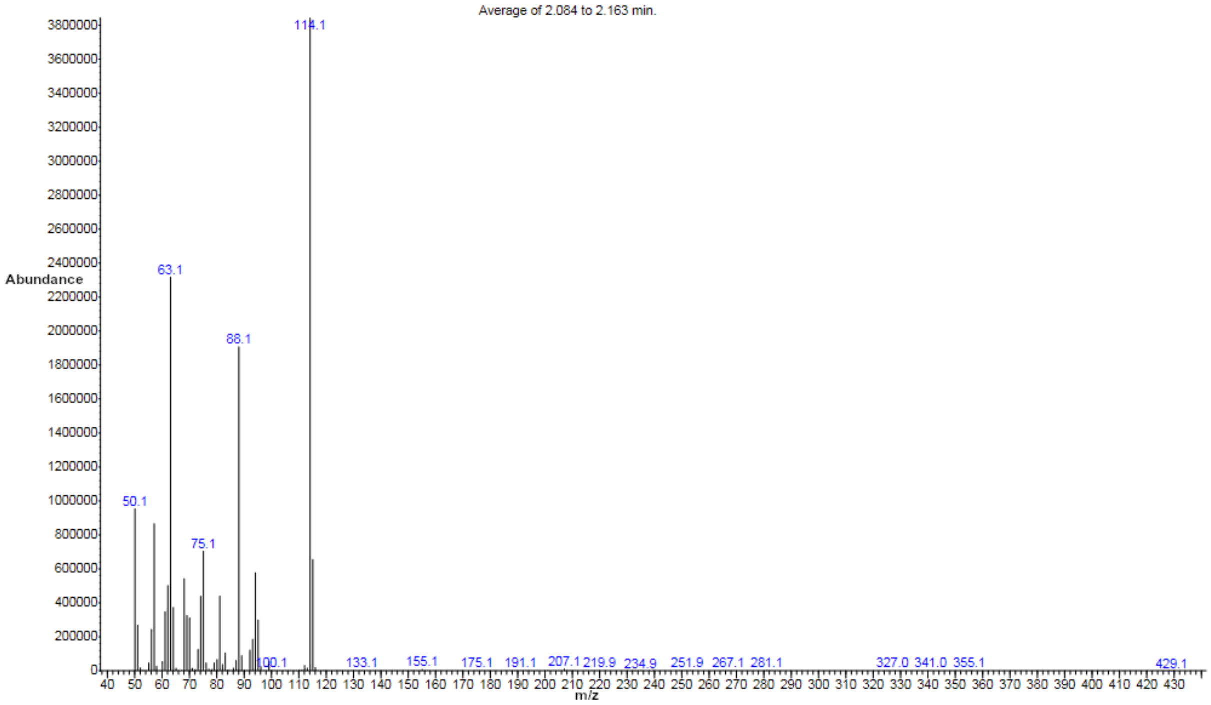

Figure S25: Extracted mass spectrum of 1,2-difluorobenzene.

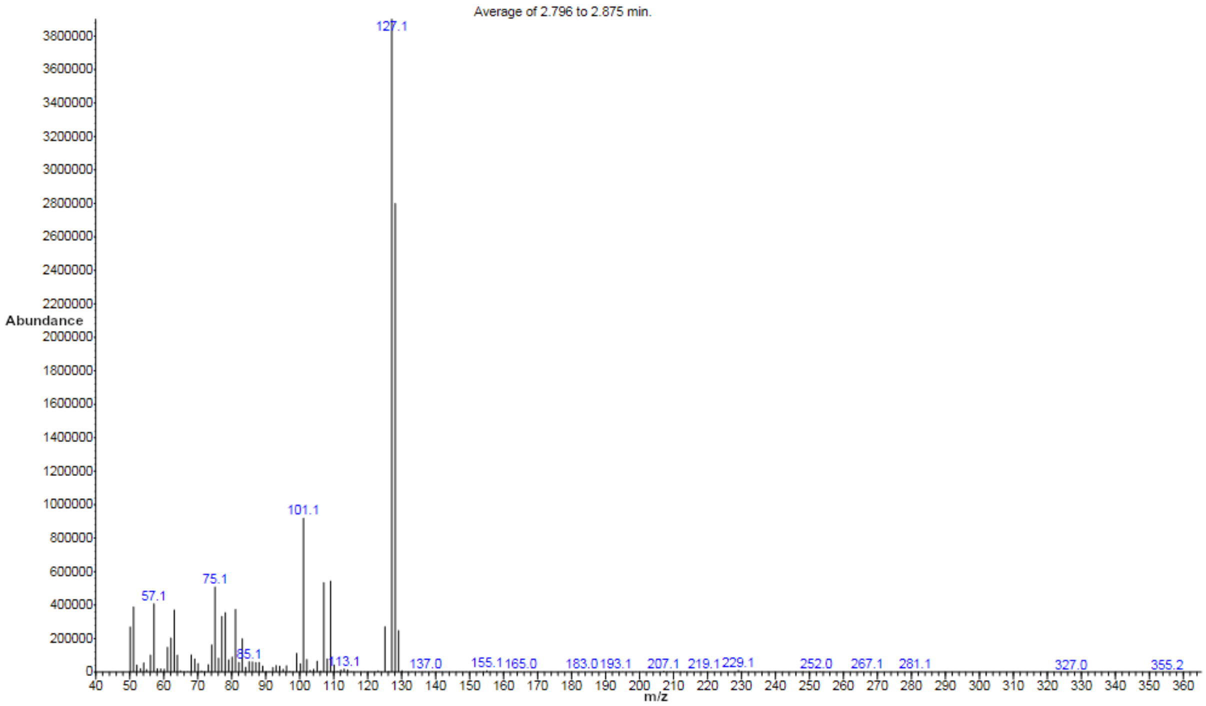

Figure S26: Extracted mass spectrum of 4-methyl-1,2-difluorobenzene.

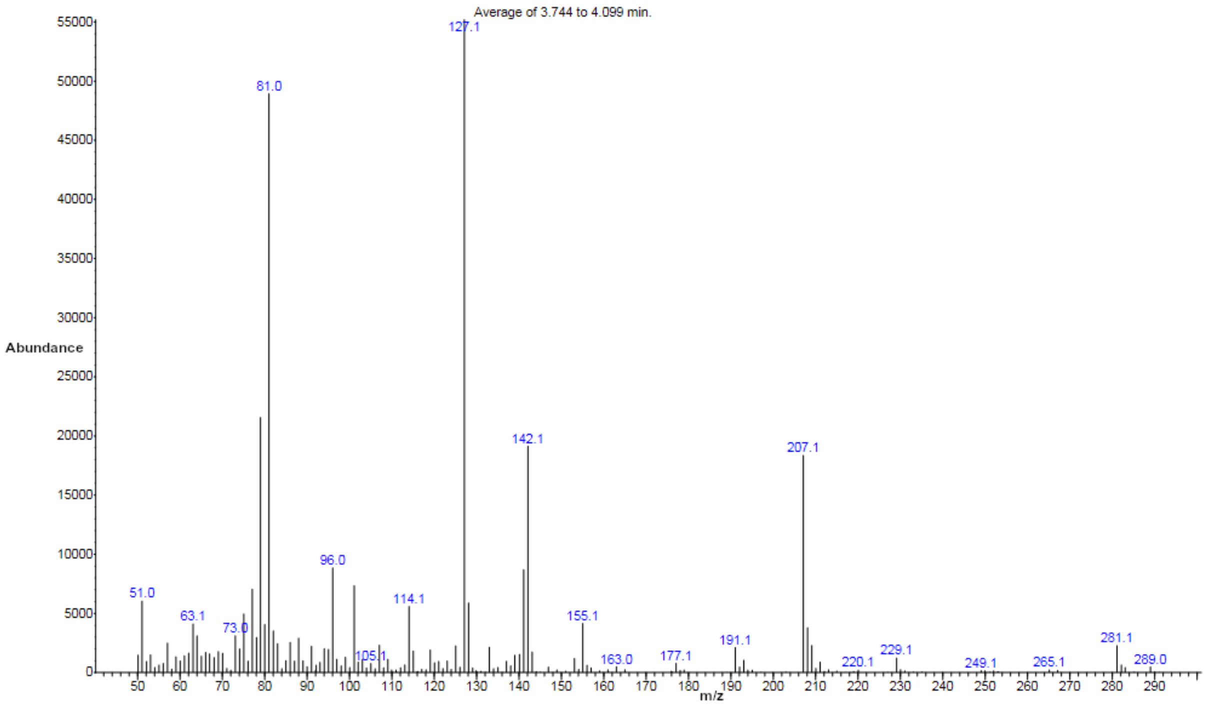

Figure S27: Extracted mass spectrum of dimethyl-1,2-difluorobenzenes, not 4,5-dimethyl-1,2-difluoro-benzene.

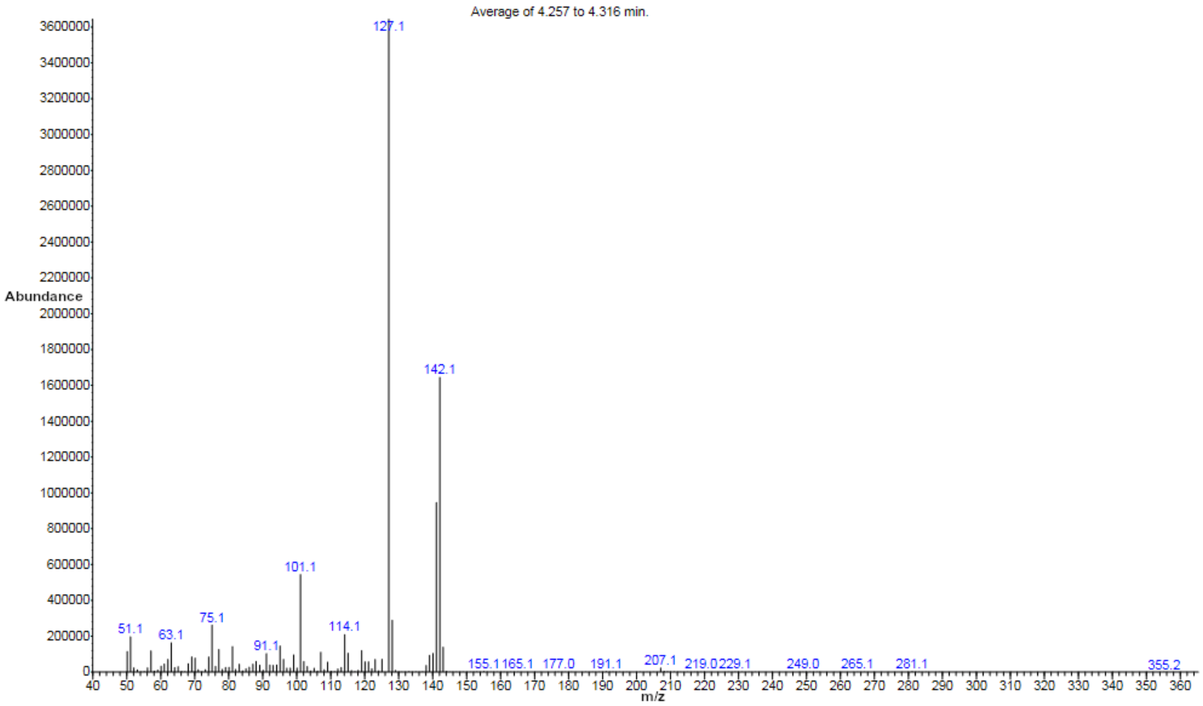

Figure S28: Extracted mass spectrum of 4,5-dimethyl-1,2-difluorobenzene.

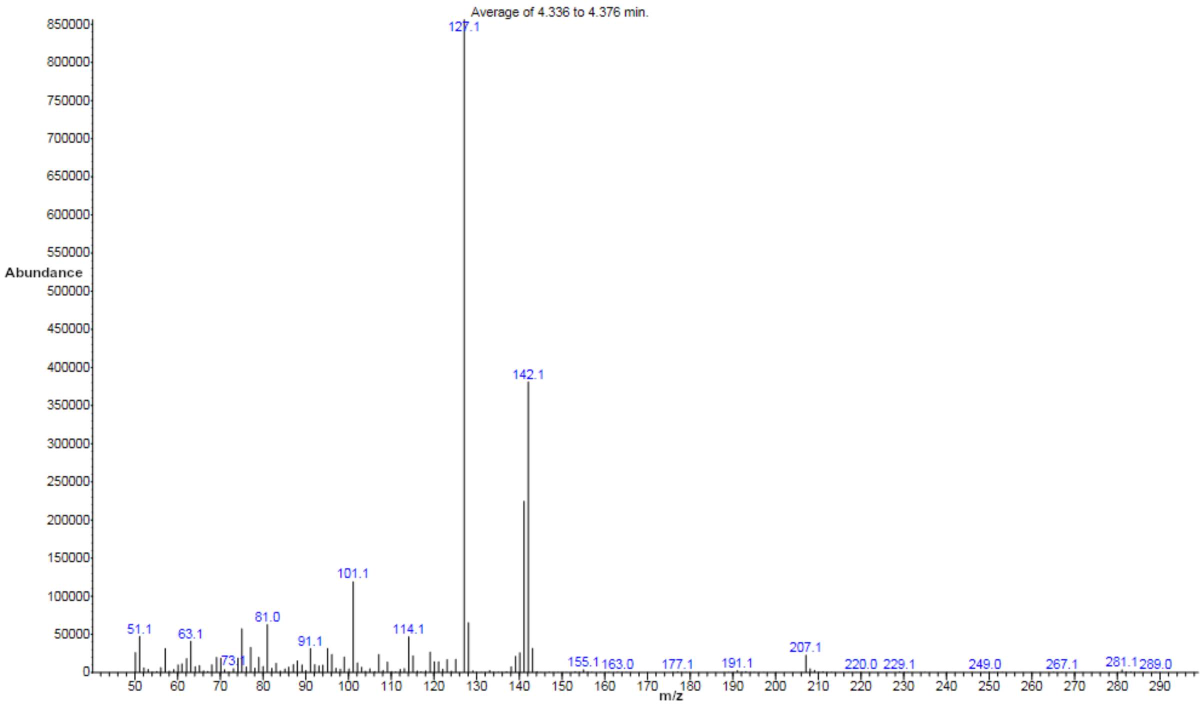

Figure S29: Extracted mass spectrum of the fourth species of dimethyl-1,2-difluorobenzene.

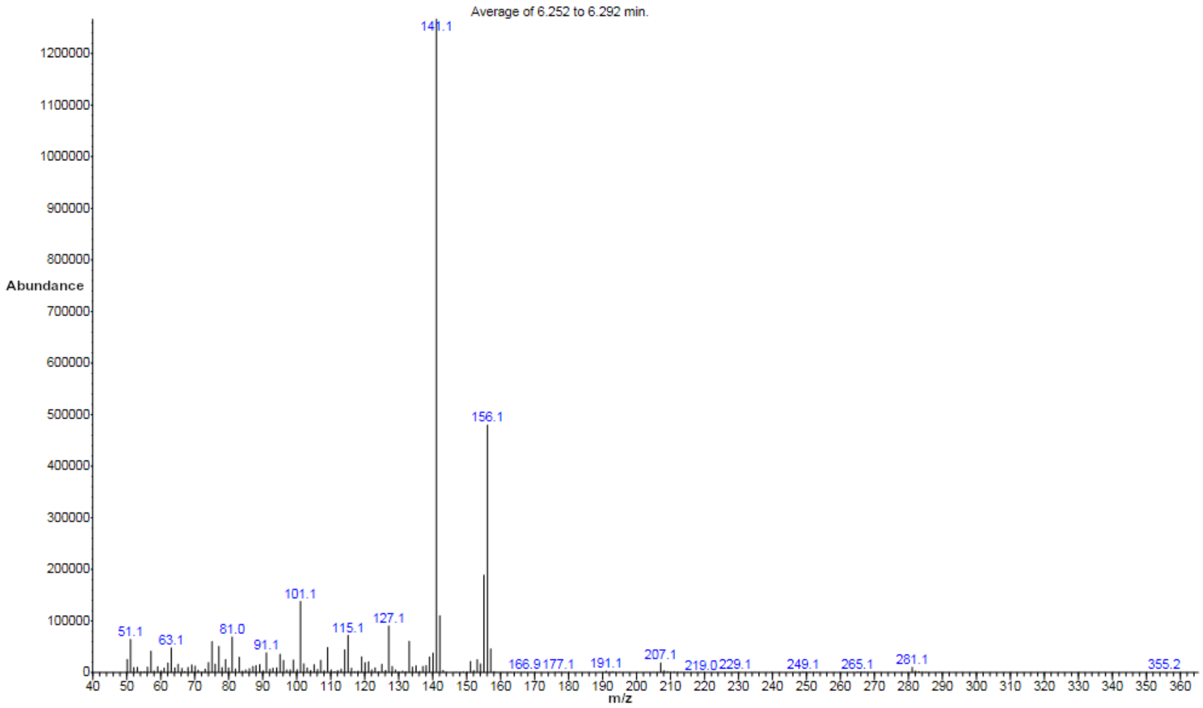

Figure S30: Extracted mass spectrum of the more abundant trimethyl-1,2-difluorobenzene.

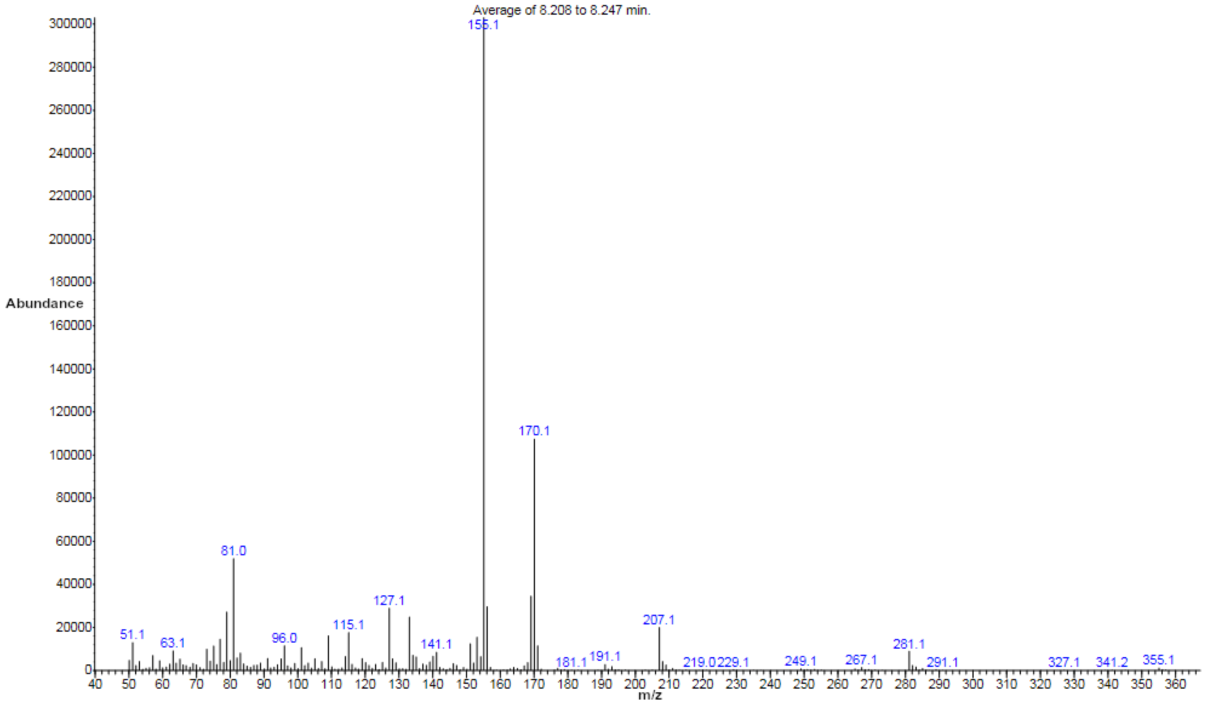

Figure S31: Extracted mass spectrum of tetramethyl-1,2-difluorobenzene.

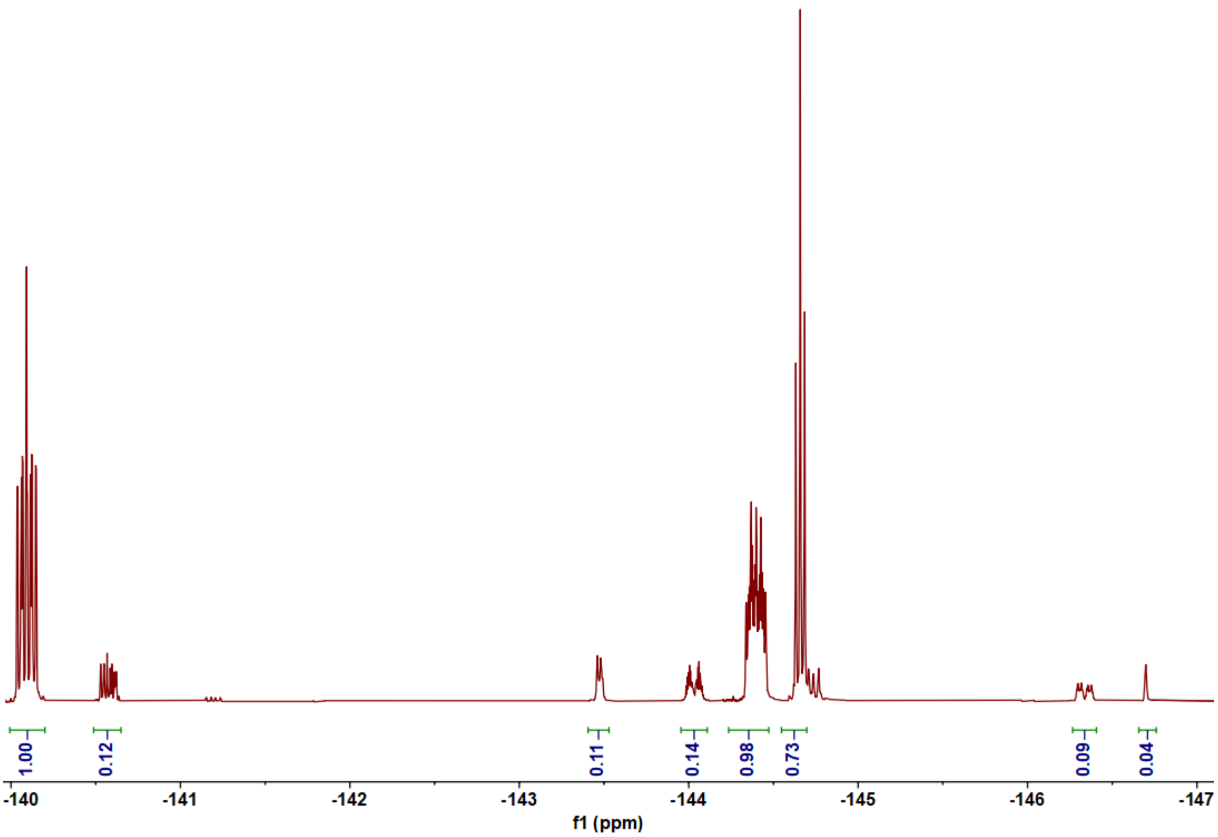

Figure S32: Section of ^{19}F NMR spectrum (377 MHz, CD_2Cl_2, r.t.) of product mixture.

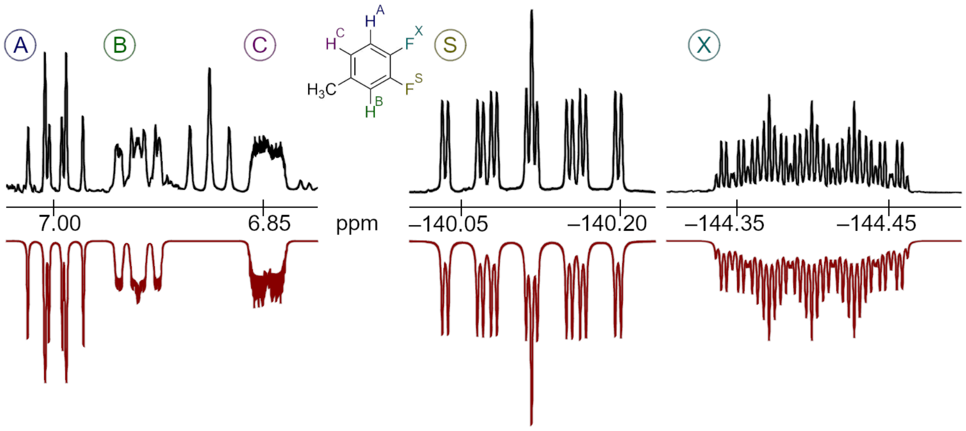

Figure S33: Experimental (top) and iterated (bottom) ^{1}H NMR (700 MHz, CD_2Cl_2, r.t.) and ^{19}F NMR (377 MHz, CD_2Cl_2, r.t.) resonances of 4-methyl-1,2-difluorobenzene (^{1}H partially overlapped by resonance of 4,5-dimethyl-1,2-trifluorobenzene).

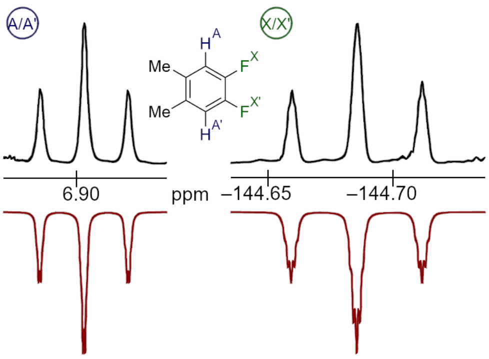

Figure S34: Experimental (top) and iterated (bottom) ^{1}H NMR (700 MHz, CD_2Cl_2, r.t.) and ^{19}F NMR (377 MHz, CD_2Cl_2, r.t.) resonances of 4,5-dimethyl-1,2-difluorobenzene.

2.4 [$MeNC_5F_4I$][$Al(OTeF_5)_4$] (2I)

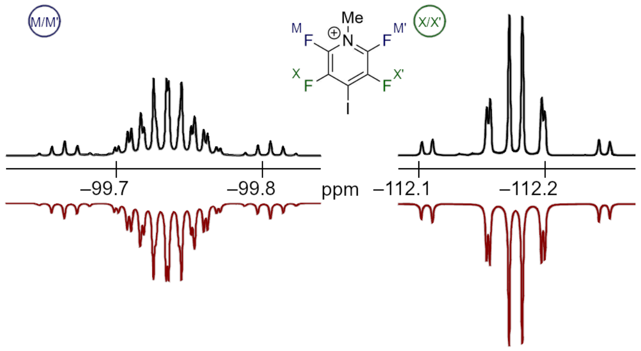

Figure S35: Experimental (top; 377 MHz, CD_2Cl_2, r.t.) and iterated (bottom) ^{19}F NMR resonances of [$MeNC_5F_4I$]$^+$.

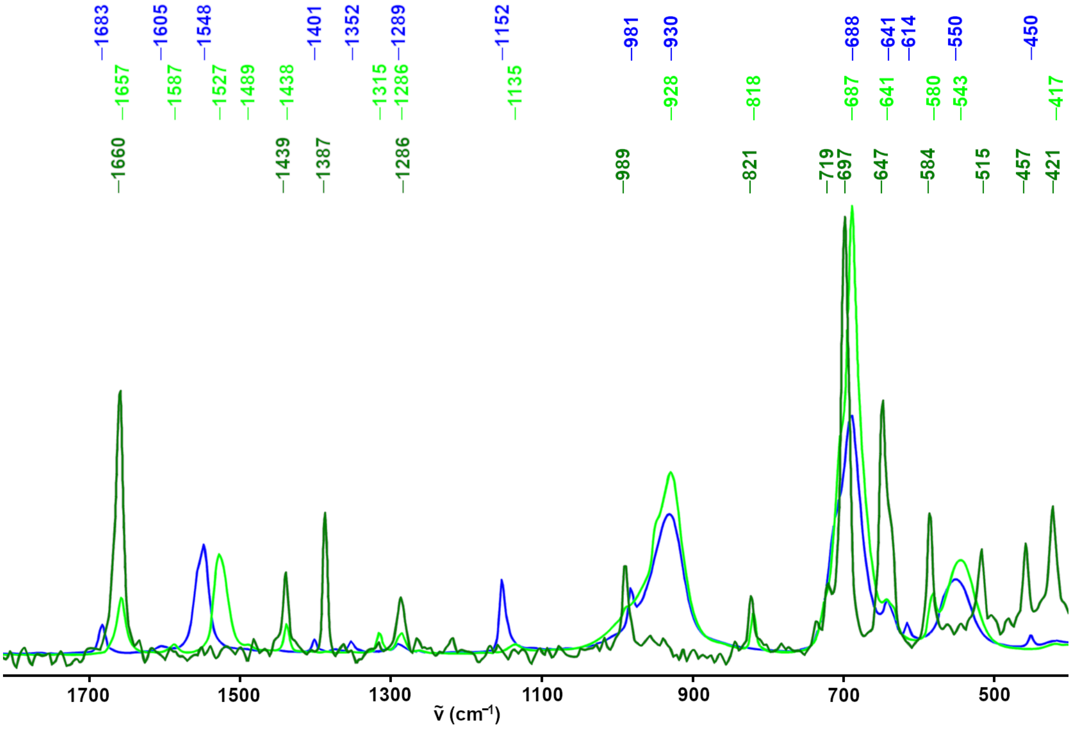

Figure S36: Section of IR spectra of [$MeNC_5F_5$][$Al(OTeF_5)_4$] (blue), [$MeNC_5F_4I$][$Al(OTeF_5)_4$] (light green) and section of Raman spectrum of [$MeNC_5F_4I$][$Al(OTeF_5)_4$] (dark green).

2.5 [MeNC₅F₅][Al(OTeF₅)₄] (2F)

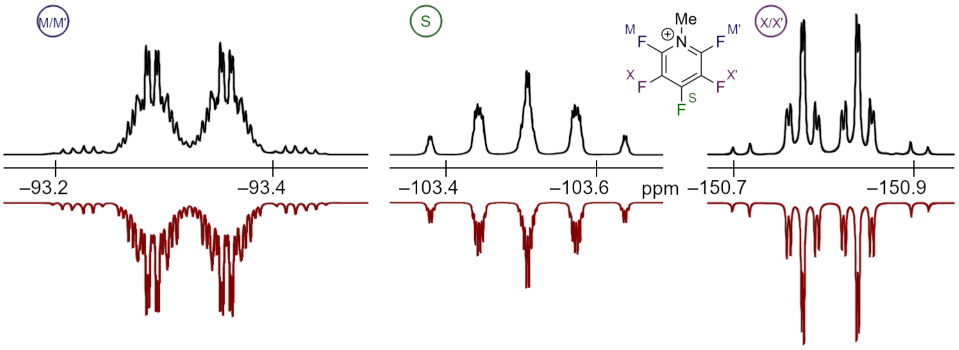

Figure S37: Experimental (top; 377 MHz, CD_2Cl_2, r.t.) and iterated (bottom) ^{19}F NMR resonances of $[MeNC_5F_5]^+$.

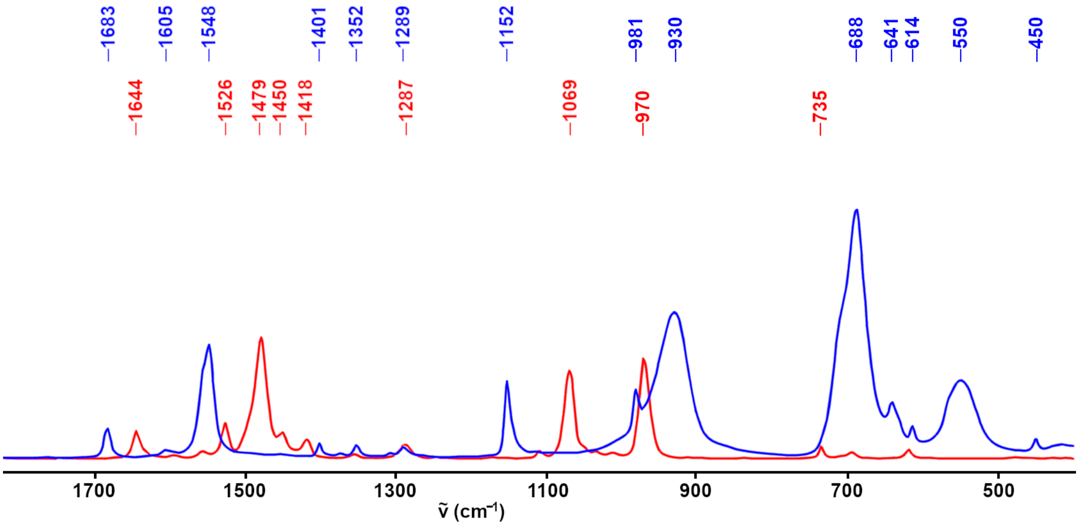

Figure S38: Section of IR spectra of $[MeNC_5F_5][Al(OTeF_5)_4]$ (blue) and NC_5F_5 (red). For comparison IR bands of $[HNC_5F_5][SbF_6]$: 1639, 1538, 1346, 1124, 1075, 1031, 980, 877, 735, 660, 546, 478 cm^{-1}[1].

3 Quantum Chemical Calculations

3.1 Reaction path for methylation of *o*DFB with $AlCl_3$/MeCl

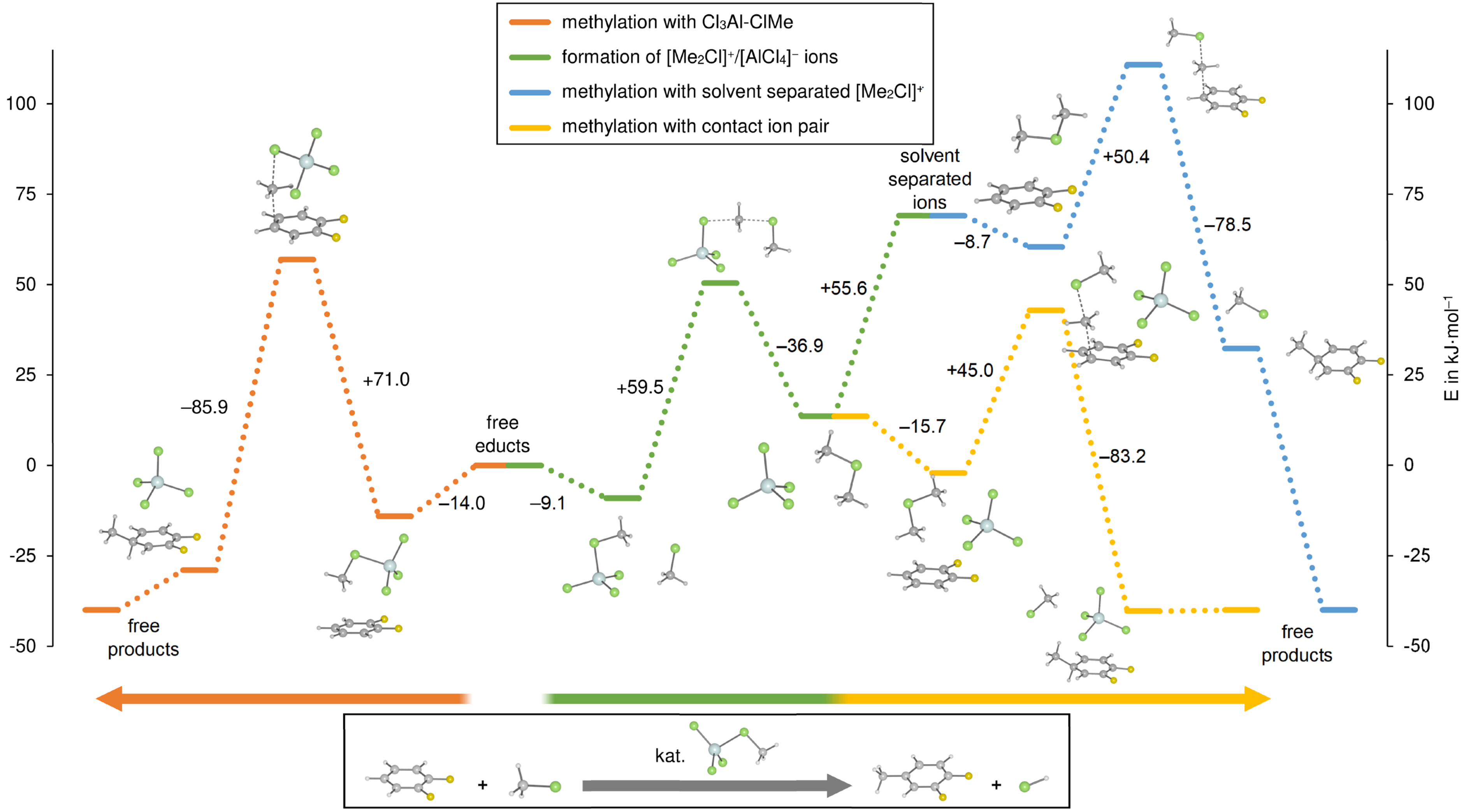

Figure S39: Possible reaction paths for the methylation of 1,2-difluorobenzene with $AlCl_3$/MeCl on the RI-B3LYP-D3/def2-TZVPP level of theory with COSMO (ε_R MeCl, ZPE corrected energies). The final steps (rearomatization, HCl elimination, recovery of $AlCl_3$-MeCl) which are common for all routes are not shown in detail for clarity.

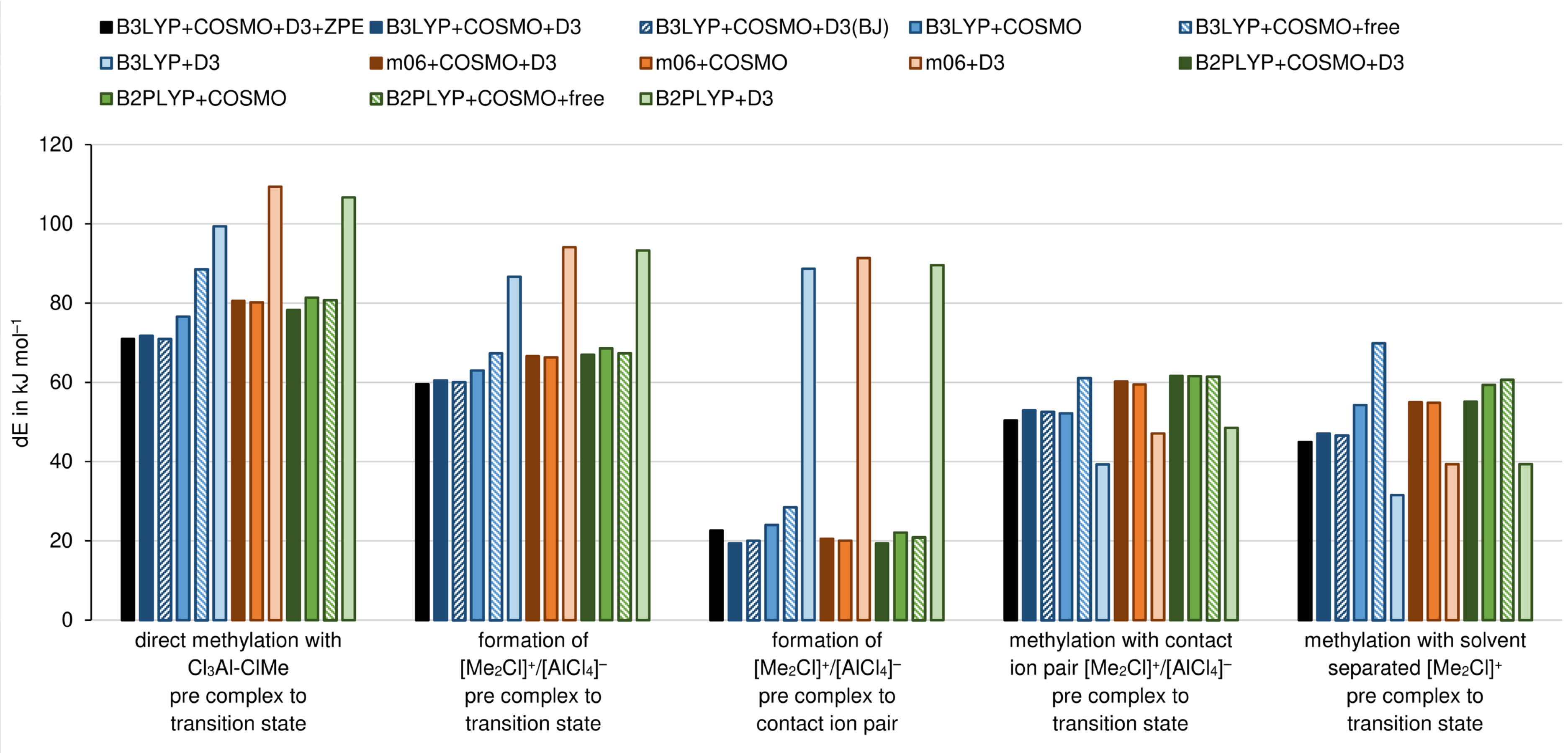

Figure S40: Selected energies from single-point calculations (def2-TZVPP basis set) of structures optimized on the RI-B3LYP-D3/def2-TZVPP level of theory with COSMO (ε_R MeCl). Values in black are equivalent to those in Figure S39. Values named with "+free" are referenced to non-interacting molecules instead of pre-complexes, since the formation of the pre-complexes is disfavored without dispersion correction. Note that even if the formation of the contact ion pair is more disfavored without a solvent model, the relative trend of the transition state is the same.

3.2 Reaction path for methylation with $[Me_2X]^+$

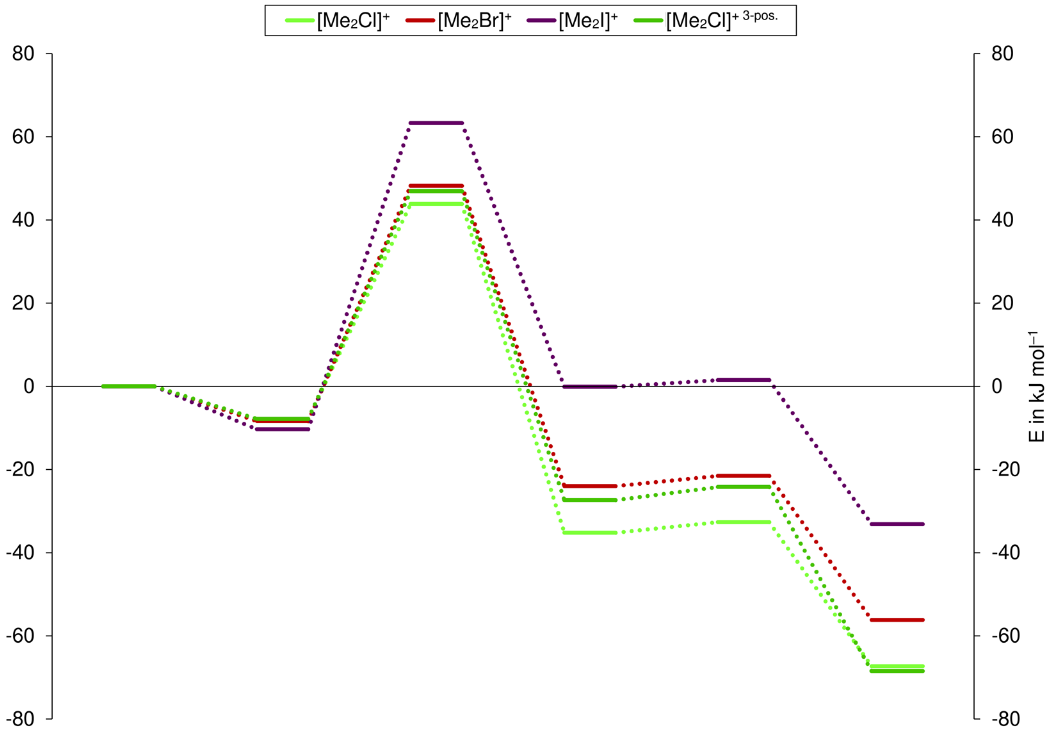

Figure S41: Reaction paths for the methylation of 1,2-difluorobenzene with $[Me_2X]^+$ on the RI-B3LYP-D3/def2-TZVPP level of theory with COSMO (ε_R SO_2, ZPE corrected energies).

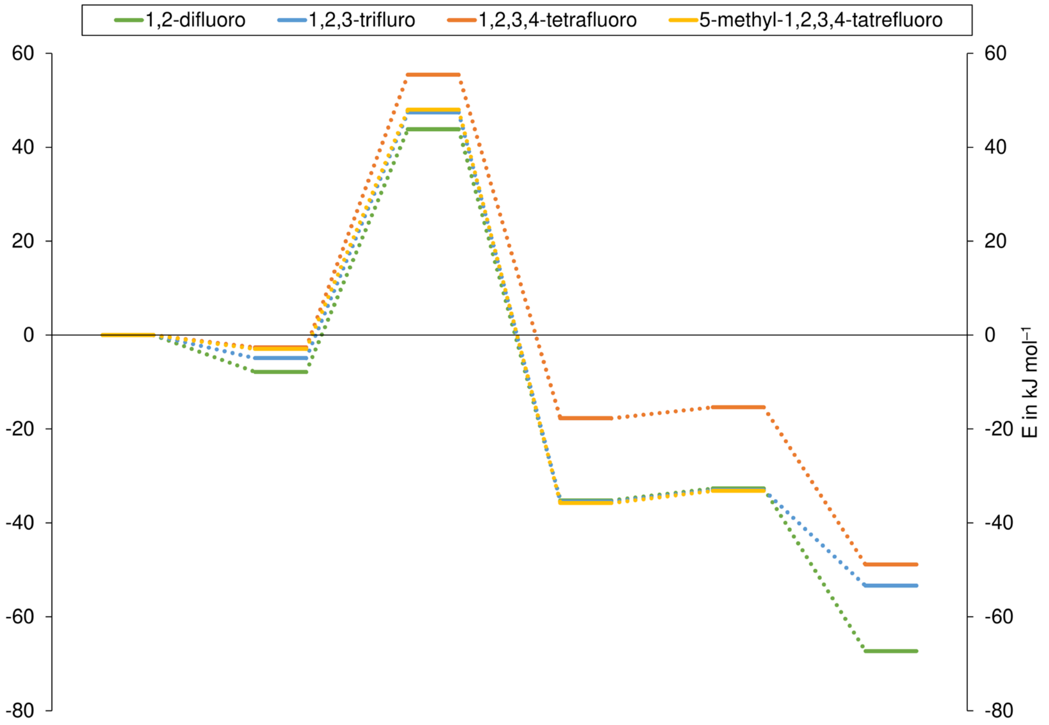

Figure S42: Reaction paths for the methylation of oligofluorobenzenes with $[Me_2Cl]^+$ on the RI-B3LYP-D3/def2-TZVPP level of theory with COSMO (ε_R SO_2, ZPE corrected energies).

Table S1: Relative energies in kJ·mol^{-1} of reaction paths for the methylation with $[Me_2X]^+$ on the RI-B3LYP-D3/def2-TZVPP level of theory with COSMO (ε_R SO_2, ZPE corrected energies).

compounds	not interacting reactants to pre-complex	pre-complex to transition state	transition state to product complex	product complex to not interacting products	H^+ shift of Wheland cation
1,2-difluorobenzene $[Me_2Cl]^+$	−7.8	+51.7	−79.0	2.5	−34.6
1,2-difluorobenzene $[Me_2Br]^+$	−8.4	+56.6	−72.2	3.4	−34.6
1,2-difluorobenzene $[Me_2I]^+$	−10.3	+73.6	−63.4	1.6	−34.6
1,2-difluorobenzene $[Me_2Cl]^+$ 3 position	−7.8	+54.7	−74.2	3.1	−44.3
1,2,3-trifluorobenzene $[Me_2Cl]^+$	−4.9	+52.3	−82.9	2.5	−20.4
1,2,3,4-tetrafluoroben-zene $[Me_2Cl]^+$	−2.6	+58.1	−73.1	2.4	−33.5
5-methyl-1,2,3,4-tetra-fluorobenzene $[Me_2Cl]^+$	−3.0	+50.9	−83.7	2.6	n/a

3.3 Fluoride ion affinity of $[MeN_3C_3F_{3-x}(OTeF_5)_x]^+$

Table S3: Fluoride ion affinity (FIA) of the ions $[MeN_3C_3F_{3-x}(OTeF_5)_x]^+$ (x = 0-3) and methyl cation affinity (MCA) of $N_3C_3F_{3-x}(OTeF_5)_x$ in $kJ\cdot mol^{-1}$ on the RI-B3LYP-D3/def2-TZVPP level of theory and NPA charge at the carbon atoms of the cations. For the FIA Me_3SiF/Me_3Si^+[2] was used as anchor point. Note that the basicity of the molecules increases with introduction of pentafluoro-*ortho*tellurates when they are not incorporated in a hypervalent 3-center-4-electron bond. This was already shown in our previous publication[3].

**x**	**MCA**	**FIA**	**NPA charge at**		
			**C2**	**C4**	**C6**
0	358.5	778.6	0.843	0.829	0.843
1	390.4	752.0	0.762 (C-O)	0.826	0.841
2	413.4	722.4	0.762 (C-O)	0.827	0.761 (C-O)
3	427.1	713.0	0.756	0.743	0.764

3.4 Optimized structures for reaction paths in the $AlCl_3$ system – COSMO MeCl

All structures on RI-B3LYP-D3/def2-TZVPP level of theory with COSMO (ε_R = 10 for MeCl at 20 °C[4]).

MeCl

```
$coord
  -0.00000000000000    0.00000000000000    0.30503759877231  c
  -0.97535892335006   -1.68937121085797    0.93667402965344  h
  -0.97535892335006    1.68937121085797    0.93667402965344  h
   1.95071784670010    0.00000000000000    0.93667402965344  h
   0.00000000000000    0.00000000000000   -3.11505968773260  cl
$end
```

E(COSMO) = -500.0657429646 H

ZPE = 98.07 kJ/mol

enthalpy = 108.53 kJ/mol

chem. pot. = 38.47 kJ/mol

$[Me_2Cl]^+$

```
$coord
  -1.98360120477685   -0.02090808471483    1.83393088418715  c
  -2.26106970734948   -1.97835324876680    1.29937005869955  h
  -0.51359934238604    0.26073558779988    3.23186952105531  h
  -3.73568619042416    0.92615434172789    2.32054135723187  h
  -0.93222622329660    1.62285488433355   -1.02150424329913  cl
   2.00744317945521   -0.02066111904581   -1.80773532536949  c
   2.66308371405147    0.94019287189733   -3.49613040468612  h
   3.25789959918452    0.24403272350288   -0.20752278047561  h
   1.49775617554186   -1.97404795673409   -2.15281906734350  h
$end
```

E(COSMO) = -539.7233454861 H

ZPE = 200.6 kJ/mol

enthalpy = 215.57 kJ/mol

chem. pot. = 129.95 kJ/mol

1,2-difluorobenzene

```
$coord
   1.31162206342536    0.00000000000000   -2.16918401906098  c
  -1.31162206342536    0.00000000000000   -2.16918401906098  c
  -2.64335468403867    0.00000000000000    0.07812658019130  c
  -1.31495430401535    0.00000000000000    2.35048561207893  c
   1.31495430401535    0.00000000000000    2.35048561207893  c
   2.64335468403867    0.00000000000000    0.07812658019130  c
  -4.68602689915431    0.00000000000000    0.02353198005730  h
  -2.34404762081789    0.00000000000000    4.11557725031850  h
   2.34404762081789    0.00000000000000    4.11557725031850  h
   4.68602689915431    0.00000000000000    0.02353198005730  h
   2.54850372039953    0.00000000000000   -4.39867022285120  f
  -2.54850372039953    0.00000000000000   -4.39867022285120  f
$end
```

E(COSMO) = -430.6907320838 H

ZPE = 219.4 kJ/mol

enthalpy = 237.56 kJ/mol

chem. pot. = 142.04 kJ/mol

4-methyl-1,2-difluorocyclohexa-2,6-dien-5-ylium cation
$coord
-0.76808470465442 -3.26992964724035 -0.65035289894714 c
1.90350838286613 -3.20628585560641 -0.73715293516839 c
3.29793006754614 -0.96169305739365 -0.66387065334508 c
2.00489473399269 1.25411332698597 -0.47771842898981 c
-2.07900993647128 -1.08740610804448 -0.46165711301768 c
5.33543224627457 -1.06074598843354 -0.75008731974105 h
3.01846674288333 3.02881546128205 -0.41630978306196 h
-4.12180046146039 -1.12363579348292 -0.38262068111546 h
-1.88647287207475 -5.51980447595306 -0.74179166752216 f
3.09096438634723 -5.35667962166329 -0.90238987156938 f
-0.76448213198015 1.35806516802001 -0.37485397380760 c
-1.32505398493185 2.35089701316919 -2.13445713868449 h
-1.75220113846926 3.09272121955992 1.79610943839738 c
-0.88775835816601 4.94978737359004 1.63333161256084 h
-3.79286263845939 3.27952410793295 1.64533959925994 h
-1.27347033324256 2.27225687727765 3.61848181475212 h
$end
E(COSMO) = -470.3614095711 H
ZPE = 323.1 kJ/mol
enthalpy = 345.77 kJ/mol
chem. pot. = 238.30 kJ/mol

$[AlCl_4]^-$
$coord
-0.00000000000000 0.00000000000000 0.00000000000000 al
2.36258413755740 2.36258413755740 -2.36258413755740 cl
-2.36258413755740 -2.36258413755740 -2.36258413755740 cl
2.36258413755740 -2.36258413755740 2.36258413755740 cl
-2.36258413755740 2.36258413755740 2.36258413755740 cl
$end
E(COSMO) = -2083.5272602171 H
ZPE = 15.12 kJ/mol
enthalpy = 36.36 kJ/mol
chem. pot. = -67.25 kJ/mol

$[AlCl_4H]$
$coord
-0.14432999888736 1.30703967655044 -0.18792593092383 al
1.79476690217931 2.72010320877650 -3.37551990998299 cl
1.15529159189850 2.49290806338780 3.39284139321171 cl
-4.07421841778577 0.69559438060665 -0.52562226494805 cl
1.43632835722188 -3.06504760550980 -0.39676021602734 cl
-0.16783843462657 -4.15059772381169 1.09298692867036 h
$end
E(COSMO) = -2083.9064867914 H
ZPE = 36.39 kJ/mol
enthalpy = 59.96 kJ/mol
chem. pot. = -54.75 kJ/mol

$[AlCl_4Me]$
$coord
0.08160305304419 2.81462867175809 -1.32924821692563 al
2.01079003955966 4.30680626447988 -4.49396450869580 cl
1.29799866919275 4.03504814493417 2.28983508224994 cl

```
  -3.88521369212266    2.35068914892103   -1.66360978335807  cl
   1.53478733879697   -1.43483879080622   -1.41774476130605  cl
  -0.06805329439596   -2.83722498505905    1.31994844488605  c
  -2.07887609199732   -2.67144485712392    0.97003335201242  h
   0.56226415510593   -4.78457646772645    1.36216498099658  h
   0.54469982281643   -1.77908712937756    2.96258541014056  h
$end
```

E(COSMO) = -2123.2102972575 H
ZPE = 115.3 kJ/mol
enthalpy = 142.89 kJ/mol
chem. pot. = 18.26 kJ/mol

[$AlCl_4$Me]/MeCl pre complex

```
$coord
   1.28736292935101    3.12804407677777   -2.90146007394298  al
   2.82941031929878    5.43080977931968   -5.77641796275453  cl
   2.44093343283996    3.89791681074282    0.86277020815303  cl
  -2.58003062372487    2.12176415435079   -3.24908412457407  cl
   3.34329614553940   -0.76636735379228   -3.72891453485583  cl
   2.04697321381006   -2.79992833830572   -1.22833496062150  c
   0.01975559734740   -2.84399101793353   -1.50184978053147  h
   2.91801822495316   -4.62892308477621   -1.52346192061738  h
   2.58728590458502   -1.95163892590004    0.55351310302765  h
  -3.07436417631915   -0.02573250317277    3.67998821853283  c
  -4.36900609844131    0.32057965777495    5.23101438657380  h
  -1.42305209374479    1.18113046519912    3.80928843314894  h
  -4.00476821952228    0.21209894037005    1.86965602082179  h
  -2.02181455597239   -3.27576266065451    3.90329298763965  cl
$end
```

E(COSMO) = -2623.2807162483 H
ZPE = 216.6 kJ/mol
enthalpy = 256.75 kJ/mol
chem. pot. = 96.65 kJ/mol

[$AlCl_4$]$^-$/[Me_2Cl]$^+$ product complex

```
$coord
  -0.04862037764516    0.12802308424633   -2.55988593555717  al
  -0.09280682419995    1.23094091805765   -6.46473684173792  cl
   3.24874086988418    1.65788886809153   -0.65612402038177  cl
  -3.39641649903897    1.51718765522227   -0.62799342059195  cl
   0.03731574046811   -3.94394875170477   -2.16384585018870  cl
   0.00853498019337   -2.34303563453237    4.15267765715521  c
  -1.67763755131100   -1.96915501085085    3.05604628879740  h
   0.02013014239759   -4.20199911129009    5.01501399375952  h
   1.74923667748934   -1.89525112839738    3.17353837386664  h
   0.08049503172068    2.94413018627940    5.39064764607791  c
  -0.08204374509593    4.23348839455753    6.97575745653485  h
   1.90832107064855    3.01796498467959    4.47224199453289  h
  -1.49048738352629    3.07137057509755    4.08264326011822  h
  -0.13351743752434   -0.18157624488120    6.86877586759051  cl
$end
```

E(COSMO) = -2623.2733213067 H
ZPE = 219.8 kJ/mol
enthalpy = 257.79 kJ/mol
chem. pot. = 107.26 kJ/mol

[$AlCl_4Me$]/MeCl to [Me_2Cl]$^+$/[$AlCl_4$]$^-$ transition state

```
$coord
  -0.00009461363604    0.08496282481222   -2.73249799222143  al
  -0.00548297131103   -0.23709328344099   -6.74678630737370  cl
   3.33034390120885    1.87560823645434   -1.23391822222236  cl
  -3.31632122428977    1.89480957101589   -1.22690314690663  cl
  -0.00784284726355   -3.80845450304230   -1.09800413919473  cl
   0.02072839952804   -2.30405872268007    3.20985811174003  c
  -1.73471239508545   -1.36646303563462    2.79641466691607  h
  -0.00511022908978   -4.20777648708403    3.93067803671709  h
   1.79869859830249   -1.41360721292710    2.78969566046712  h
   0.00517271744059    2.80322046112228    6.05063241211374  c
   0.05846801555196    3.98072885016167    7.72611887564188  h
   1.66532953449278    3.06947949368848    4.87897349586707  h
  -1.73310608489105    3.05692537574324    4.99573475318852  h
   0.05409759603688   -0.44917097867435    7.16761452449441  cl
$end
```

E(COSMO) = -2623.2576558948 H
ZPE = 215.6 kJ/mol
enthalpy = 253.20 kJ/mol
chem. pot. = 102.51 kJ/mol

[$AlCl_4$]$^-$/4-methyl-1,2-difluorocyclohexa-2,6-dien-5-yliumcation product complex

```
$coord
  -0.53139047228232   -3.47126527299218   -1.04795161140233  c
   1.95283993880297   -3.52005285613964   -0.07842920747424  c
   3.58493571898297   -1.45176567971916   -0.22172967255746  c
   2.74991791462736    0.67822296543344   -1.40251663149193  c
  -1.39190860086465   -1.38307416176644   -2.24294469461553  c
   5.43848712029238   -1.59723398684958    0.62003174302582  h
   3.97147646634391    2.30828889479392   -1.55836481744976  h
  -3.28610076496307   -1.34998341871542   -3.00444863747524  h
  -1.96048093035088   -5.51751173919455   -0.72189112970510  f
   2.74490649062217   -5.61659324311425    0.95637728070957  f
   0.27474907996175    0.80364755023929   -2.65111737479572  c
   0.84319499878899    0.55346442089762   -4.66957512481775  h
  -1.04882210447531    3.40041851950261   -2.57926388055275  c
   0.26033427596622    4.86117395046737   -3.19579347943928  h
  -2.67476745565922    3.38415581936519   -3.83722468876522  h
  -1.67251417394045    3.80886016903879   -0.66856056073185  h
  -1.86729181906643    0.83375974371700    5.29952004047781  al
   1.03046167982298    3.41519155545673    3.97887168981371  cl
  -4.85498223996796    0.65850312087247    2.50060540543357  cl
  -0.21406771265398   -2.89145500562919    5.65641492817675  cl
  -3.34897740998737    2.09324865433605    8.86799042363699  cl
$end
```

E(COSMO) = -2553.9140237952 H
ZPE = 339.9 kJ/mol
enthalpy = 386.34 kJ/mol
chem. pot. = 217.26 kJ/mol

[$AlC_{l4}Me$]/ 1,2-difluorobenzene pre complex

```
$coord
  -0.71606574143950   -1.54560211121765   -5.24184154634909  c
   1.50546066361727   -0.38374028460119   -6.01664472913306  c
   1.51119997604033    2.12263317817555   -6.75594290623937  c
  -0.74885555914126    3.47206472016229   -6.71974304044262  c
```

```
  -2.96349702396884   -0.21467167987414   -5.19222790617758  c
   3.26852078554104    2.97872664314395   -7.35076288338538  h
  -0.76006974494703    5.43155754870103   -7.29801443395492  h
  -4.65795827417610   -1.15800192633840   -4.55467227199195  h
  -0.65583162554755   -3.98965438383008   -4.54625100797786  f
   3.66297603645156   -1.73491039595028   -6.04812152726723  f
  -2.97556570587271    2.30731660424462   -5.94309684418603  c
  -4.72799580875577    3.35697390204502   -5.90833550195647  h
  -0.05099113788327    4.11081953114004   -0.30568670778022  c
   0.14087950188514    6.14560151249581   -0.41289096647210  h
  -1.92294910768623    3.46655626505592   -0.81932223127755  h
   1.42553466200186    3.12928156961120   -1.32821929740423  h
   0.02913873929451   -1.14413280206121    2.99810778179168  al
   0.37694080124950    3.33490458822305    3.04944446325148  cl
  -3.64006261879178   -1.67281319464712    1.47511037232051  cl
   3.01887040404648   -2.15455318306748    0.53516661725184  cl
   0.50383629067753   -1.96737491326731    6.88075551916669  cl
$end
E(COSMO) = -2553.9073609475 H
ZPE = 337.3 kJ/mol
enthalpy = 386.14 kJ/mol
chem. pot. = 206.25 kJ/mol
```

$[AlCl_4Me]$/ 1,2-difluorobenzene to $[AlCl_4]^-$/4-methyl-1,2-difluorocyclohexa-2,6-dien-5-yliumcation transition state

```
$coord
  -1.22528932886222   -1.17039593230465   -4.93019140964077  c
   1.15175527573226   -0.66690520225056   -5.97659201125054  c
   1.96776801145497    1.77961877186910   -6.39185797304977  c
   0.38708388160233    3.76478237060373   -5.72724049744851  c
  -2.80598651541094    0.77313522746639   -4.26938774525757  c
   3.80959698001485    2.09464504166967   -7.21414208408886  h
   0.98307586584435    5.69139016386601   -6.04609995135933  h
  -4.63217298455279    0.35851596475195   -3.45534429801676  h
  -1.91403082750461   -3.57953296607869   -4.59737803070531  f
   2.63394008667032   -2.61396292098322   -6.57068625821160  f
  -1.97075433281409    3.28662589683879   -4.59426264994715  c
  -3.28735494480462    4.81942353480740   -4.29528789393954  h
  -0.16572430326727    3.72592869149962   -0.73839698705096  c
   0.62903564867470    5.58071526904937   -1.00329161509299  h
  -2.03282663013244    3.54399940443334    0.03974114185186  h
   0.98382407527169    2.08318493706222   -1.06216723722430  h
   0.19149640599338   -0.52939636321266    4.00815467029647  al
   1.40546564846146    3.49172727736304    3.66780633055674  cl
  -3.78247836818533   -0.55012026000093    3.13343473496998  cl
   2.23223604894047   -2.54473486640140    1.12022993959317  cl
   1.01984000324391   -1.79797578282732    7.74361914648245  cl
$end
E(COSMO) = -2553.8800120345 H
ZPE = 336.5 kJ/mol
enthalpy = 382.76 kJ/mol
chem. pot. = 211.78 kJ/mol
```

MeCl/4-methyl-1,2-difluorocyclohexa-2,6-dien-5-yliumcation product complex

```
$coord
   0.76948454182133   -5.56629692184129   -1.80278767280899  c
   3.43919227299942   -5.48547431145900   -1.68487853101357  c
```

4.82647313743952	-3.24400665522078	-1.88172535240271	c
3.52589303493779	-1.04508252330760	-2.18221924189085	c
-0.54901949925134	-3.39987688856244	-2.09681061222167	c
6.86405687979841	-3.33098258925351	-1.78396464150460	h
4.53318783073132	0.72691854298678	-2.34259857457801	h
-2.59137848464948	-3.44708347069551	-2.17776122421921	h
-0.34170909089584	-7.81257161221133	-1.59868542533689	f
4.63324490584134	-7.61847195893954	-1.38747881948004	f
0.75827762950659	-0.96033673518189	-2.32533697367683	c
0.34789390778888	-0.29296321544218	-4.27040536844688	h
-0.40432864165762	1.11147168726950	-0.57923631177186	c
0.47363665138859	2.92354803368411	-0.98610203185355	h
-2.42397408171107	1.24662242116057	-0.92765253290673	h
-0.08252394202991	0.62499171458449	1.39085981760995	h
-3.43204679921443	4.63010275995609	3.99086563239410	cl
-4.88651843546651	7.35941416868696	5.45971083360773	c
-3.65949161759616	8.97087985783851	5.14278793624380	h
-6.71703681554717	7.64321085210919	4.58138762267572	h
-5.08331338423342	6.96598684383887	7.46203147158124	h

$end
E(COSMO) = -970.4294204007 H
ZPE = 423.5 kJ/mol
enthalpy = 459.38 kJ/mol
chem. pot. = 311.69 kJ/mol

$[Me_2Cl]^+$/1,2-difluorobenzene pre complex
$coord

-1.66891517764385	-1.89832024000188	-0.69061499404700	c
0.04648127235565	-2.31721135942973	1.25342522938950	c
2.58111720746947	-2.69815052774330	0.73780350054484	c
3.39273566831381	-2.66040199098982	-1.76731600191432	c
-0.87567929906650	-1.85616190399333	-3.18030992076471	c
3.87475265133222	-3.02072743579766	2.28587424697474	h
5.36912349594858	-2.95983516996494	-2.18726579698011	h
-2.24892343097575	-1.52651323552372	-4.65671511139368	h
-4.11761429579647	-1.51504902569378	-0.11011235076103	f
-0.79652934340545	-2.32761797613324	3.65722563019927	f
1.67254461983709	-2.24309068071536	-3.71463592184018	c
2.30680021659724	-2.21490172118539	-5.65611319574297	h
1.54166202831379	4.17360098461031	-0.77773023620976	c
3.06780854342557	5.40748268628194	-0.19388081863597	h
0.46568886758513	4.86995012464748	-2.37397806868961	h
2.13976913494171	2.22632041450162	-1.00434081754039	h
-0.65623105243260	4.01521523621535	1.88873014917118	cl
-1.58222558337190	7.32176852323199	2.33689827755285	c
0.13786886223661	8.33950676062753	2.78372864224563	h
-2.46971321796648	7.90672959102522	0.58643866426687	h
-2.89549321078583	7.24464593871715	3.90948359108410	h

$end
E(COSMO) = -970.4182898177 H
ZPE = 422.4 kJ/mol
enthalpy = 458.28 kJ/mol
chem. pot. = 315.64 kJ/mol

$[Me_2Cl]^+$/1,2-difluorobenzene to MeCl/4-methyl-1,2-difluorocyclohexa-2,6-dien-5-yliumcation transition state
$coord

```
   -1.33152625302950    -3.67539316609001    -0.81735914932688  c
    0.74595549699146    -4.38199765701428     0.65148282888412  c
    3.04907295762989    -3.15921036969960     0.42905727920354  c
    3.27411964984753    -1.19654289202474    -1.29017945657141  c
   -1.13149559060092    -1.73346079196900    -2.52785973274950  c
    4.61730867705318    -3.76863158436189     1.58611267563279  h
    5.06353437872414    -0.23673630896757    -1.50815398097098  h
   -2.74794122432703    -1.22165873687220    -3.66694784260579  h
   -3.50725592816887    -4.93707592531629    -0.53453698897053  f
    0.47195359625916    -6.27493659610349     2.28994006747475  f
    1.17818055906611    -0.42315435419031    -2.73239015212237  c
    1.42620390342530     0.92674474676695    -4.24592147855944  h
    0.17172145905272     2.80360186034597     0.05054453014585  c
    1.98978722968425     3.66434047741117    -0.24265431330357  h
   -1.38499674394893     3.28128279417278    -1.16575304987843  h
   -0.09348025203183     1.47827039283612     1.57032745099976  h
   -0.97529817499185     6.18378483198970     2.84042743267312  cl
   -0.41254443792056     8.89500160827697     0.80875515602002  c
    1.58474791399877     8.91757977009705     0.35324099509930  h
   -1.58791429957144     8.67120807090046    -0.85435219493068  h
   -0.95706426285152    10.54165944690571     1.90079067768982  h
$end
```
E(COSMO) = -970.3981142369 H
ZPE = 419.8 kJ/mol
enthalpy = 454.47 kJ/mol
chem. pot. = 315.07 kJ/mol

HCl

```
$coord
    0.00000000000000     0.00000000000000    -1.21390505723017  cl
    0.00000000000000     0.00000000000000     1.21390505723017  h
$end
```
E(COSMO) = -460.7717134059 H
ZPE = 17.28 kJ/mol
enthalpy = 25.95 kJ/mol
chem. pot. = -29.81 kJ/mol

4-methyl-1,2-difluorobenzene

```
$coord
   -0.65023211120992    -3.30409011592959     0.03045925414114  c
    1.97246505952747    -3.32516658175968     0.00283088970811  c
    3.29675802508388    -1.07883416235667    -0.02769726121707  c
    1.96937907264004     1.19556579446449    -0.03025784245751  c
   -0.66633094511619     1.24539133724800    -0.00279974645640  c
   -1.96938802955267    -1.05426720372143     0.02779706123429  c
    5.33961549233345    -1.12557251407358    -0.04933281709629  h
    3.01459838100913     2.95267878691404    -0.05438511902524  h
   -1.90578610211027    -5.52524353740089     0.05973058016550  f
    3.20271867705380    -5.56175247667663     0.00579030928515  f
   -4.01352389978290    -1.11712665933995     0.04945686702332  h
   -2.10841487214619     3.69940439600604    -0.00334532959747  c
   -3.36167973161540     3.82002399585194    -1.63805125709458  h
   -3.28636810917644     3.86409008244769     1.68296417071912  h
   -0.83381090693777     5.31489885832611    -0.05315975933212  h
$end
```
E(COSMO) = -469.9964338503 H
ZPE = 290.9 kJ/mol

enthalpy = 313.46 kJ/mol
chem. pot. = 206.28 kJ/mol

$[AlCl_4]^-/[Me_2Cl]^+$/1,2-difluorobenzene pre complex

```
$coord
  -0.67673053920736   -2.70146625521096   -3.58794647026301  c
   1.79596848128220   -2.21821899879709   -4.31948121460451  c
   2.48906537139214    0.14258332307882   -5.19764278826589  c
   0.66678578980866    2.03615601841502   -5.34986999482817  c
  -2.49309732248165   -0.83000667835549   -3.72498065369329  c
   4.42936289383184    0.46694319084435   -5.74983358646377  h
   1.19340894461372    3.88584795338300   -6.04081609070934  h
  -4.39621724138329   -1.25336803327321   -3.11963602861011  h
  -1.28130520138649   -5.01496904838430   -2.73086038424758  f
   3.51841621259906   -4.08327811686596   -4.16555873645893  f
  -1.81343537391210    1.55097264639672   -4.61838795088639  c
  -3.22552647480873    3.02333770034363   -4.73519447344227  h
   0.11140525406233    3.37949634215398    0.94702172235953  c
   0.22816537043532    4.90278089633757   -0.41546665869150  h
  -1.78247939429917    2.64299863717930    1.18441772411410  h
   1.51645369299733    1.91945159497601    0.65858309001109  h
  -0.72527944056840   -4.30352035499201    4.12308565515521  al
   2.68641633280542   -2.96574893138570    2.28085674415127  cl
  -3.86473171304520   -1.95714181055970    2.91184448377739  cl
  -1.41312882173167   -8.20895280513716    3.28148762129834  cl
  -0.25465859769555   -3.74765782981526    8.15864064268014  cl
   0.92494936322967    4.93541093699972    3.94639112816194  cl
   0.62421825515469    2.38470105924223    6.28439574121878  c
   2.04871183199891    1.00083353006869    5.79472248942940  h
   0.98649180980547    3.32322010966250    8.06993071487190  h
  -1.29322948349722    1.68959492369524    6.11429727393572  h
$end
```

E(COSMO) = -3053.9703637609 H
ZPE = 440.1 kJ/mol
enthalpy = 499.98 kJ/mol
chem. pot. = 294.64 kJ/mol

$[AlCl_4]^-$/MeCl/4-methyl-1,2-difluorocyclohexa-2,6-dien-5-yliumcation product complex

```
$coord
   0.79826366844593   -5.13871714120132   -0.85918695864815  c
   3.11581791054971   -4.88315005156298    0.43487812569676  c
   4.68294024042465   -2.77875897862708    0.15960018032045  c
   3.95564902349966   -0.92462734042236   -1.47233226239319  c
   0.04943588235952   -3.32896521677092   -2.50108049464667  c
   6.39942721002448   -2.67583765117998    1.25884632571046  h
   5.13230100868250    0.72423061441788   -1.73866560303443  h
  -1.71483756006708   -3.53167066419925   -3.50910581290850  h
  -0.59414225024670   -7.18176536333343   -0.38583373391829  f
   3.81640005846986   -6.72730179040595    1.91960099614065  f
   1.67915325364414   -1.14411102868611   -3.04316725914930  c
   2.53057002875902   -1.67589992042048   -4.90110375627164  h
   0.26594847340598    1.35202632367587   -3.57491967325943  c
   1.59549757589956    2.77071803803121   -4.24339281396154  h
  -1.16572313029659    1.04446796251347   -5.01784874977357  h
  -0.63680648268904    2.02851506683656   -1.86385038671189  h
  -1.61237071297707    0.01694364669791    4.40864132473545  al
   1.38158852464094    2.48917729139529    3.10054384289391  cl
```

-4.13855450949656	-0.79178630723221	1.28540784063958	cl
0.05715697407189	-3.49441550923331	5.66507522266912	cl
-3.67175307989656	1.82993364846983	7.40709842425696	cl
-3.51868680161185	6.95745953483951	-1.44445688194799	cl
-4.47328978479417	6.35326378832602	1.78553118518597	c
-2.77598137418716	6.24688963524971	2.92770546839621	h
-5.68151292970764	7.89613646077567	2.38745666467001	h
-5.47649121690736	4.56724495204642	1.81455878530886	h

$end
E(COSMO) = -3053.9853590830 H
ZPE = 441.3 kJ/mol
enthalpy = 500.34 kJ/mol
chem. pot. = 296.98 kJ/mol

$[AlCl_4]^-/[Me_2Cl]^+$/1,2-difluorobenzene to $[AlCl_4]^-$/MeCl/4-methyl-1,2-difluorocyclohexa-2,6-dien-5-yliumcation transition state

$coord

-0.89152623391072	-1.73936947352121	-4.47005392932207	c
1.58242161532642	-1.63330449568052	-5.37912918097226	c
2.52956495743174	0.53292806118710	-6.49801611469624	c
0.96932786404011	2.62498940735877	-6.71381634441234	c
-2.45167344033428	0.32448176731470	-4.66051169035700	c
4.45425257213743	0.54367682346309	-7.17940233693123	h
1.66673502878211	4.32496187437744	-7.60520215945181	h
-4.35914474421413	0.21124929561622	-3.94630832476001	h
-1.70353527422853	-3.88241775580134	-3.40390678947271	f
3.04118415871636	-3.67386990809565	-5.14318887869662	f
-1.51002844583016	2.55954206786429	-5.75506465015156	c
-2.80557060666712	4.08319449710587	-6.17216387681665	h
-0.34410689729383	4.74767500965895	-2.12289016139380	c
0.18611860348944	6.32620900295590	-3.28774072108073	h
-2.26238847765384	4.55280668834228	-1.47808105227660	h
1.03727659818835	3.35644688864499	-1.57395275881513	h
-0.73627014888883	-1.75146176831346	3.23936968339059	al
2.60146964339917	-0.78132483055608	1.04764048407033	cl
-3.89200237750276	0.43546047794567	1.78469835571055	cl
-1.51054159795250	-5.72591070680549	2.96902189450050	cl
-0.08762106627529	-0.66116424788123	7.12240231863973	cl
0.68348883226529	7.37299552131424	1.46272349297823	cl
0.65727267029162	5.27711255161650	4.19084701921326	c
2.07751969190660	3.84031962417371	3.86630641784904	h
1.11412450183108	6.45516933398691	5.80453931093650	h
-1.22261874256523	4.48190865837170	4.32910589159936	h

$end
E(COSMO) = -3053.9524216110 H
ZPE = 438.0 kJ/mol
enthalpy = 496.20 kJ/mol
chem. pot. = 297.47 kJ/mol

Single-point energies for above shown structures at different levels of theory, all with def2-TZVPP basis set.

		E(B3LYP+COSMO) in eV	E(B3LYP+D3) in eV
1	MeCl	-13607,45371355	-13607,37439064
2	$[Me_2Cl]^+$	-14686,52112381	-14684,36967036
3	*o*DFB	-11719,52257631	-11719,56811474
4	[4Me4H-*o*DFB]$^+$	-12798,89355985	-12797,13395657
5	$[AlCl_4]^−$	-56695,50503494	-56693,82848653
6	$[AlCl_4H]$	-56705,80303746	-56705,61738367
7	$[AlCl_4Me]$	-57775,21886443	-57775,17952447
8	$[AlCl_4Me]$/MeCl pre complex	-71382,62671960	-71382,81087141
9	$[AlCl_4]^−/[Me_2Cl]^+$ product complex	-71382,37748959	-71381,89128726
10	8 to 9 transition state	-71381,97419864	-71381,91260346
11	$[AlCl_4]^−$/[4Me4H-*o*DFB]$^+$ product complex	-69494,68370425	-69494,61946133
12	$[AlCl_4Me]$/oDFB pre complex	-69494,61727191	-69494,97268284
13	11 to 12 transition state	-69493,82325323	-69493,94201240
14	MeCl/[4Me4H-*o*DFB]$^+$ product complex	-26406,34985256	-26404,71301909
15	$[Me_2Cl]^+$/*o*DFB pre complex	-26405,95195835	-26404,34104829
16	14 to 15 transition state	-26405,41057723	-26403,93371057
17	HCl	-12538,23753085	-12538,12054729
18	4Me-*o*DFB	-12789,01029192	-12789,13082377
19	$[AlCl_4]^−/[Me_2Cl]^+$/oDFB pre complex	-83101,73898705	-83101,72378767
20	$[AlCl_4]^−$/MeCl/[4Me4H-*o*DFB]$^+$ product complex	-83102,07502133	-83102,29653641
21	19 to 20 transition state	-83101,17595363	-83101,39633469

		E(m06+COSMO+D3) in eV	E(m06+COSMO) in eV	E(m06+D3) in eV
1	MeCl	-13608,07750839	-13608,07716469	-13607,97360666
2	$[Me_2Cl]^+$	-14686,95542403	-14686,94767236	-14684,70938636
3	*o*DFB	-11718,59339329	-11718,56993779	-11718,47389979
4	[4Me4H-*o*DFB]$^+$	-12797,92279323	-12797,88234089	-12795,87493845
5	$[AlCl_4]^-$	-56699,60575555	-56699,60247722	-56697,76612536
6	$[AlCl_4H]$	-56709,88163445	-56709,87363482	-56709,52497370
7	$[AlCl_4Me]$	-57779,19804589	-57779,18128883	-57778,88893103
8	$[AlCl_4Me]$/MeCl pre complex	-71387,38214596	-71387,32799465	-71387,09619894
9	$[AlCl_4]^-/[Me_2Cl]^+$ product complex	-71387,16927269	-71387,11923233	-71386,14937886
10	8 to 9 transition state	-71386,69170374	-71386,64094566	-71386,12135410
11	$[AlCl_4]^-$/[4Me4H-*o*DFB]$^+$ product complex	-69498,26809507	-69498,15777638	-69497,33996958
12	$[AlCl_4Me]$/oDFB pre complex	-69497,99155033	-69497,87727532	-69497,61647468
13	11 to 12 transition state	-69497,15670142	-69497,04648510	-69496,48216919
14	MeCl/[4Me4H-*o*DFB]$^+$ product complex	-26406,03845814	-26405,98510227	-26404,02353602
15	$[Me_2Cl]^+$/*o*DFB pre complex	-26405,64140045	-26405,56559970	-26403,56303808
16	14 to 15 transition state	-26405,01699309	-26404,94932157	-26403,07454117
17	HCl	-12539,02198809	-12539,02198735	-12538,90742872
18	4Me-*o*DFB	-12787,99006632	-12787,95474678	-12787,86971897
19	$[AlCl_4]^-/[Me_2Cl]^+$/oDFB pre complex	-83105,97122999	-83105,81408200	-83104,92749343
20	$[AlCl_4]^-$/MeCl/[4Me4H-*o*DFB]$^+$ product complex	-83106,50553312	-83106,35092567	-83105,61865513
21	19 to 20 transition state	-83105,40156803	-83105,24546064	-83104,51876417

		E(B2PLYP+COSMO+D3) in eV	E(B2PLYP+COSMO) in eV	E(B2PLYP+D3) in eV
1	MeCl	-13604,95165110	-13604,93490416	-13604,84282181
2	$[Me_2Cl]^+$	-14683,70139576	-14683,64564825	-14681,44889938
3	*o*DFB	-11717,00284693	-11716,91318180	-11716,87693925
4	[4Me4H-*o*DFB]$^+$	-12796,06583343	-12795,90899103	-12794,01682563
5	$[AlCl_4]^-$	-56684,90854128	-56684,81565870	-56683,07439325
6	$[AlCl_4H]$	-56695,18437993	-56695,08082080	-56694,81062065
7	$[AlCl_4Me]$	-57764,34087157	-57764,18348373	-57764,01944394
8	$[AlCl_4Me]$/MeCl pre complex	-71369,39441307	-71369,13092714	-71369,09491334
9	$[AlCl_4]^-$/$[Me_2Cl]^+$ product complex	-71369,19288576	-71368,90137769	-71368,16624920
10	8 to 9 transition state	-71368,70020288	-71368,41972609	-71368,12160122
11	$[AlCl_4]^-$/[4Me4H-*o*DFB]$^+$ product complex	-69481,64435697	-69481,17803069	-69480,69477926
12	$[AlCl_4Me]$/oDFB pre complex	-69481,50359791	-69481,10262276	-69481,11151262
13	11 to 12 transition state	-69480,69204835	-69480,25921922	-69480,00516253
14	MeCl/[4Me4H-*o*DFB]$^+$ product complex	-26401,07523758	-26400,86928622	-26399,05874770
15	$[Me_2Cl]^+$/*o*DFB pre complex	-26400,81591894	-26400,55980536	-26398,72524165
16	14 to 15 transition state	-26400,17597040	-26399,92171659	-26398,22263472
17	HCl	-12536,06264571	-12536,06260569	-12535,94399596
18	4Me-*o*DFB	-12786,24343889	-12786,11265666	-12786,11808462
19	$[AlCl_4]^-$/$[Me_2Cl]^+$/oDFB pre complex	-83086,35600067	-83085,80117660	-83085,29768970
20	$[AlCl_4]^-$/MeCl/[4Me4H-*o*DFB]$^+$ product complex	-83086,72546311	-83086,12970134	-83085,82268845
21	19 to 20 transition state	-83085,78461439	-83085,18569752	-83084,88887254

3.5 Optimized structures for reaction paths in the $[Me_2X]^+$ system – COSMO SO_2

All structures on the RI-B3LYP-D3/def2-TZVPP level of theory with COSMO (ε_R = 17.6 for SO_2 at 20 °C[5]).

MeCl

```
$coord
   0.00000000000000    0.00000000000000    0.30637490808900    c
  -0.97553808240623   -1.68968152344587    0.93647831279739    h
  -0.97553808240623    1.68968152344587    0.93647831279739    h
   1.95107616481245    0.00000000000000    0.93647831279739    h
   0.00000000000000    0.00000000000000   -3.11580984648114    cl
$end
```

E(COSMO) = -500.0660811218 H
ZPE = 97.25 kJ/mol
enthalpy = 107.74 kJ/mol
chem. pot. = 34.92 kJ/mol

$[Me_2Cl]^+$

```
$coord
  -1.98180326193356   -0.02131516935396    1.83118016465804  c
  -2.26083864247687   -1.97763232425139    1.29387300540267  h
  -0.50898001143314    0.25727900959446    3.22657509888194  h
  -3.73226963863705    0.92616396555571    2.32187918357650  h
  -0.93309760175267    1.62423891208472   -1.02242347055642  cl
   2.00548255869734   -0.01993956196023   -1.80522521091350  c
   2.66159808781640    0.93702686410424   -3.49538742550695  h
   3.25490150105379    0.24800375524369   -0.20492583481124  h
   1.49500700866568   -1.97382545101722   -2.14554551073098  h
$end
```

E(COSMO) = -539.7291366492 H
ZPE = 201.1 kJ/mol
enthalpy = 215.91 kJ/mol
chem. pot. = 130.66 kJ/mol

MeBr

```
$coord
   0.00000000000000    0.00000000000000    0.38271196959917  c
  -0.97823546024063   -1.69435351890230    0.98820497983510  h
  -0.97823546024063    1.69435351890230    0.98820497983510  h
   1.95647092048124    0.00000000000000    0.98820497983510  h
   0.00000000000000    0.00000000000000   -3.34737403120829  br
$end
```

E(COSMO) = -2613.9491087438 H
ZPE = 96.37 kJ/mol
enthalpy = 107.04 kJ/mol
chem. pot. = 33.51 kJ/mol

$[Me_2Br]^+$

```
$coord
   0.05314153946580    1.62387123353746    2.34114357175232  c
  -0.17471397329586    3.54793028441753    3.00877559884958  h
   1.96158661042506    0.91914650526824    2.56666725811094  h
  -1.39505996628559    0.35706564101903    3.03962613056818  h
  -0.51090740645040    1.88835889209863   -1.34805855222387  br
  -0.03160704956732   -1.70205915411860   -2.28531297103378  c
```

-0.33563765027720 -1.72248444144125 -4.31187563134162 h
1.89113707682529 -2.16689743641332 -1.75908908036529 h
-1.45793918083973 -2.74493152436763 -1.25187632431644 h
$end
E(COSMO) = -2653.6152620210 H
ZPE = 196.4 kJ/mol
enthalpy = 212.49 kJ/mol
chem. pot. = 121.72 kJ/mol

MeI
$coord
0.00000000000000 0.00000000000000 0.46073540816364 c
-0.97909680493718 -1.69584541167952 1.05930323410005 h
-0.97909680493718 1.69584541167952 1.05930323410005 h
1.95819360987433 0.00000000000000 1.05930323410005 h
0.00000000000000 0.00000000000000 -3.63869723988383 i
$end
E(COSMO) = -337.6136671023 H
ZPE = 95.20 kJ/mol
enthalpy = 106.04 kJ/mol
chem. pot. = 30.18 kJ/mol

$[Me_2I]^+$
$coord
-0.00003414751966 0.00000962792181 -0.53430831156309 i
-3.03179189830635 0.00007607482261 2.18416695708707 c
-2.83178447291680 1.72852531835198 3.26310876171838 h
-4.73840548409708 -0.02881085111426 1.04846728813510 h
-2.79720893198294 -1.70157949952102 3.29824532506166 h
3.03208958518733 0.00006829674999 2.18386918207193 c
4.73901276542950 -0.01251021485839 1.04832085530059 h
2.82214332704637 1.72078136933296 3.27321739911238 h
2.80699469891293 -1.70933864042406 3.28806624420272 h
$end
E(COSMO) = -377.2883560804 H
ZPE = 194.6 kJ/mol
enthalpy = 210.81 kJ/mol
chem. pot. = 117.90 kJ/mol

1,2-difluorobenzene
$coord
1.31150582329906 0.00000000000000 -2.16856719252402 c
-1.31150582329906 0.00000000000000 -2.16856719252402 c
-2.64406784552604 0.00000000000000 0.07811315992355 c
-1.31503671216498 0.00000000000000 2.35030960861693 c
1.31503671216498 0.00000000000000 2.35030960861693 c
2.64406784552604 0.00000000000000 0.07811315992355 c
-4.68677896087381 0.00000000000000 0.02420734630269 h
-2.34371171713491 0.00000000000000 4.11565141404106 h
2.34371171713491 0.00000000000000 4.11565141404106 h
4.68677896087381 0.00000000000000 0.02420734630269 h
2.54760811961374 0.00000000000000 -4.39984715562640 f
-2.54760811961374 0.00000000000000 -4.39984715562640 f
$end
E(COSMO) = -430.6911045433 H
ZPE = 219.5 kJ/mol
enthalpy = 237.60 kJ/mol

chem. pot. = 142.09 kJ/mol

4-methyl-1,2-difluorocyclohexa-2,6-dien-5-yliumcation

$coord
-0.76806534264309 -3.26708652465925 -0.64733265745518 c
1.90283027456452 -3.20349476513272 -0.73238009326137 c
3.29703864394969 -0.95954126357027 -0.66491650794870 c
2.00320778998631 1.25647792372052 -0.48481101212735 c
-2.08018116047832 -1.08495767555115 -0.46830312742700 c
5.33449360997307 -1.05775363521029 -0.74936751913608 h
3.01621952450661 3.03143051472277 -0.42563926331447 h
-4.12297644170358 -1.12018072585804 -0.39257448436682 h
-1.88654594725865 -5.51922664475955 -0.73207908257566 f
3.09080193288858 -5.35570913499161 -0.88902143921712 f
-0.76561800803187 1.35976111190817 -0.38200012527424 c
-1.32956512984975 2.35920268838582 -2.13607544184348 h
-1.74955315075034 3.08637693423215 1.79795403522237 c
-0.87844172676384 4.94108787895174 1.64484906264279 h
-3.78944083389397 3.27965372317119 1.64596095602338 h
-1.27420403449539 2.25395959464048 3.61573670005898 h
$end
E(COSMO) = -470.3667835484 H
ZPE = 323.0 kJ/mol
enthalpy = 345.76 kJ/mol
chem. pot. = 237.87 kJ/mol

4-methyl-1,2-difluorocyclohexa-2,6-dien-4-yliumcation

$coord
-2.04882273049047 -3.36225380451209 -0.03195777903573 c
0.49322163204851 -3.41410183196194 -0.07285965615982 c
0.51557080057887 1.36209129953975 0.04825551221889 c
-3.31632475590583 -1.00017813894110 0.06530584214579 c
1.50334084184167 -5.18936564078304 -0.13074618391208 h
-5.78525011924492 -1.04590567763304 0.11363618463998 f
-3.46432665112606 -5.44506017053635 -0.07037664803823 f
1.94953306064026 3.76740926653727 -0.01249531664278 c
2.52121877861267 4.13330235723909 -1.96869230639879 h
0.81150401523410 5.35436906021092 0.62701267623432 h
3.67776030232281 3.63758370110859 1.10033380707393 h
1.92949837839217 -1.03894507005532 -0.01001424588958 c
3.20573951366684 -1.08733621890213 1.63655463897617 h
3.29193350123573 -1.00595890360556 -1.58095207393942 h
-2.08019190395826 1.31527536190953 0.10916277651411 c
-3.20440466384805 3.01907441038566 0.17783277221324 h
$end
E(COSMO) = -470.3788746487 H
ZPE = 320.1 kJ/mol
enthalpy = 343.45 kJ/mol
chem. pot. = 234.95 kJ/mol

3-methyl-1,2-difluorocyclohexa-1,5-dien-4-yliumcation

$coord
-0.08404418850664 -2.26209822396932 -0.52676492982544 c
2.50036879391992 -2.27167386526424 -0.70938356521369 c
3.80395428971337 0.00851344076391 -0.74433612406772 c
2.55031954436830 2.35004115037201 -0.61418136822234 c
-0.01485349752180 2.40448646780844 -0.43643109071428 c

5.84470131032029	-0.06221293502643	-0.88323593166515	h
3.64744618330584	4.06975505366389	-0.65415160911885	h
-1.02772690031400	4.17696923531417	-0.33809601705184	h
-1.32582271460365	-4.39894102934166	-0.48242079974917	f
3.75263839835753	-4.45015867877545	-0.83546708554500	f
-1.54561469517157	0.08501766331842	-0.38737489908910	c
-2.67740460967384	0.13075181644245	-2.14815805833003	h
-3.51180384357039	0.05480670993919	1.81333787834744	c
-4.67796149135065	1.73979882525994	1.67878638101869	h
-4.70144181158832	-1.61017807140046	1.64847720597703	h
-2.53275476768440	0.03512244089505	3.61940001324947	h

$end
E(COSMO) = -470.3636036517 H
ZPE = 323.1 kJ/mol
enthalpy = 346.05 kJ/mol
chem. pot. = 237.98 kJ/mol

3-methyl-1,2-difluorocyclohexa-1,5-dien-3-yliumcation
$coord

-0.05960005099312	-2.36390782114664	-0.03215139045722	c
2.59041461605517	-2.32391743518297	-0.05112854781688	c
4.02974874098377	-0.09348210100530	-0.02376896740327	c
2.76589840699763	2.13476929190543	0.03199568141180	c
-1.41586418031518	-0.16917839682096	0.02390976992054	c
6.06618365437362	-0.23225393015556	-0.04565254682716	h
3.78003704516022	3.90817816041554	0.06027577722073	h
-4.21773618484117	-0.16991745191720	0.00241386081464	c
-4.97175180718344	1.66985255932801	0.51834362483028	h
-4.87471789397975	-0.63545618467424	-1.90352658077024	h
-4.96349844118307	-1.61943248374464	1.26188637390617	h
-1.20450156916335	-4.61149302442163	-0.07657443845666	f
3.76501231630293	-4.49756890024348	-0.10080449540246	f
-0.00256711410057	2.22936963776407	0.07259075091230	c
-0.60986241469344	3.32609517009480	1.73765847886231	h
-0.67719512342020	3.44834290980464	-1.47546735074492	h

$end
E(COSMO) = -470.3791394158 H
ZPE = 319.6 kJ/mol
enthalpy = 343.06 kJ/mol
chem. pot. = 234.48 kJ/mol

$[Me_2Cl]^+$/1,2-difluorbenzene pre complex
$coord

1.99009994250624	-1.87137788902849	-2.60751811307687	c
-0.56469852760366	-2.39913880499734	-2.90609512928192	c
-1.82506493472790	-3.89798914580658	-1.17668792733569	c
-0.49397327295916	-4.87619729349828	0.87364491731018	c
2.06405707930428	-4.34076870978220	1.17707849013439	c
3.32163174005978	-2.82445604333577	-0.57063543864442	c
-3.81331534291054	-4.27915077396817	-1.45152393603610	h
-1.46569742407680	-6.05127648303227	2.23284402398546	h
3.09135474761421	-5.09735811959729	2.77224343379269	h
5.30640586658318	-2.37839830269507	-0.38069420374337	h
3.15023610195760	-0.38924692224770	-4.32651536893425	f
-1.79897132724640	-1.41369461502593	-4.90305306480046	f
-1.01961465870669	2.03885868705789	2.51541916982546	c
-0.24593545937240	0.14306570483650	2.40096319040638	h

-0.33852130909536	3.06880505094833	4.14887681494930	h
-3.05410477728309	2.08337005775552	2.29320899325154	h
0.29674440540789	3.63829140151735	-0.25057114658074	cl
-1.00708270611725	6.83378021321277	0.02398829436666	c
-0.23449554354587	7.80944646197289	-1.60504618362121	h
-3.04226561798223	6.62481433170509	-0.06222864832824	h
-0.31678898180586	7.57862119400878	1.80230183236122	h

$end
E(COSMO) = -970.4239864498 H
ZPE = 422.6 kJ/mol
enthalpy = 458.43 kJ/mol
chem. pot. = 315.63 kJ/mol

MeCl/4-methyl-1,2-difluorocyclohexa-2,6-dien-5-yliumcation product complex
$coord

0.75915934637169	-5.55527931493427	-1.69576170311792	c
3.42798494726331	-5.48337796365936	-1.56832197717986	c
4.82743250541091	-3.25859713786056	-1.84027182785870	c
3.53930349643698	-1.06578979211531	-2.22814685577591	c
-0.54786769895697	-3.39558440201020	-2.07510555363493	c
6.86385275117824	-3.35073968324306	-1.72829810258907	h
4.55649081242985	0.69392268532006	-2.44865269443517	h
-2.59008433457076	-3.43492494735372	-2.16315401661417	h
-0.36473320550752	-7.78844652936523	-1.41271889162280	f
4.60953152209386	-7.61131513621929	-1.18594683049783	f
0.77356039365954	-0.97401008620570	-2.39385440145301	c
0.38766406431582	-0.39777580341908	-4.37388325616761	h
-0.39518935327737	1.17992882278607	-0.75950701327182	c
0.49843806804366	2.96642210106762	-1.23963429192896	h
-2.41031145260581	1.30844230887519	-1.13740995293383	h
-0.09749215551521	0.78196968260770	1.23411393935883	h
-3.50092758310924	4.81995456914065	3.66814213383265	cl
-4.89292406113767	7.30037782323603	5.57517034409024	c
-3.62887638674274	8.91358533657098	5.52572546443232	h
-6.71619807903976	7.76762388335063	4.76275444101374	h
-5.09881359674050	6.58361358343054	7.48476104635393	h

$end
E(COSMO) = -970.4348227302 H
ZPE = 423.7 kJ/mol
enthalpy = 459.46 kJ/mol
chem. pot. = 312.14 kJ/mol

$[Me_2Cl]^+$/1,2-difluorbenzene to MeCl/4-methyl-1,2-difluorocyclohexa-2,6-dien-5-yliumcation transition state
$coord

-1.33182419449973	-3.68077807704182	-0.82709927213673	c
0.74783907742453	-4.38417541779778	0.64086042533519	c
3.04441967442020	-3.14948417568782	0.42976372757511	c
3.26116290153852	-1.17684612246478	-1.27948139799879	c
-1.14111721448837	-1.73019267524313	-2.52695638889973	c
4.61339429932684	-3.75339082126031	1.58851341387068	h
5.04342130247407	-0.20162758147471	-1.48549875748449	h
-2.75863299691846	-1.21754914925191	-3.66400046777810	h
-3.50194654929278	-4.95631940223310	-0.55047339690733	f
0.47913497950402	-6.28744387156020	2.27013417810836	f
1.16358609807556	-0.40759569404644	-2.72268157295280	c
1.40754777359649	0.94862966620164	-4.23098509641730	h

0.18951403495068	2.78516076800300	0.05688606328290	c
2.01271731361393	3.64323886588075	-0.21089866530604	h
-1.35645467948589	3.28937371471888	-1.16228974450178	h
-0.10324109025465	1.47058782511830	1.58088798035769	h
-0.96132650396897	6.19304333610437	2.85533872739120	cl
-0.40960450604346	8.88402419143914	0.79464663605749	c
1.58345916963921	8.89613127684599	0.32063647248627	h
-1.59895199122049	8.64630717718051	-0.85652737986800	h
-0.94002824410121	10.54358178366292	1.87379526961943	h

$end
E(COSMO) = -970.4033517994 H
ZPE = 420.1 kJ/mol
enthalpy = 454.67 kJ/mol
chem. pot. = 315.52 kJ/mol

MeCl/3-methyl-1,2-difluorocyclohexa-1,5-dien-4-yliumcation product complex
$coord

2.84734372191151	-2.28703867209017	-2.22486959932576	c
5.37771995903123	-2.28555266638436	-1.66801673371347	c
6.58139815062437	-0.01614140802523	-1.12000359000317	c
5.28364675431035	2.30481794626431	-1.13028224776552	c
2.77202230187567	2.34839494822425	-1.68290504436259	c
8.57984582352311	-0.07838084048805	-0.68237937634848	h
6.30388066651363	4.01739183502017	-0.69535468256690	h
1.72842157094988	4.10553959101509	-1.70993592238671	h
1.69670390869990	-4.41531727634824	-2.73133633606064	f
6.66832257242418	-4.44538026195302	-1.63580952189456	f
1.34946784648739	0.03964420569553	-2.29122748919727	c
0.77141543106569	0.26015934201673	-4.29179477192604	h
-1.16514622635129	-0.19612368432792	-0.76641911849354	c
-2.28222553448270	1.49985157606734	-1.06640329710269	h
-2.22173591847785	-1.82792371209531	-1.42485457883538	h
-0.74841031610117	-0.40175784760252	1.23396320767363	h
-5.85165081024008	0.95596065367934	3.73612123331749	cl
-8.99113291841805	0.20833332371786	4.88080605762175	c
-9.01051096106014	0.51324414529964	6.90797168135066	h
-10.31645490855913	1.45439900586031	3.93553019542817	h
-9.37292111372652	-1.75412020354590	4.42719993459079	h

$end
E(COSMO) = -970.4315342973 H
ZPE = 422.9 kJ/mol
enthalpy = 459.27 kJ/mol
chem. pot. = 309.33 kJ/mol

$[Me_2Cl]^+$/1,2-difluorbenzene to MeCl/3-methyl-1,2-difluorocyclohexa-1,5-dien-4-yliumcation transition state
$coord

2.67974148539883	-1.19895338464756	-1.24865971533633	c
4.11559915819411	-0.74815633505807	0.88995616971620	c
4.56335213748251	1.70554193103076	1.68379255058985	c
3.56621025208277	3.73465915130910	0.32651815388191	c
2.12711474322455	3.30208975345264	-1.81289754381143	c
5.68744259491851	2.00258473773256	3.36448880418074	h
3.93427986713840	5.64156405579987	0.95454017450230	h
1.35740765863727	4.85893917429317	-2.88655248491614	h
2.26083204368889	-3.56850238411717	-1.99939007488998	f
5.07414803539403	-2.70950496416895	2.17348519410523	f

1.59819270494123 0.80899973785086 -2.59391976196718 c
0.73665406130456 0.42395737919383 -4.40669334346107 h
-2.19459005062088 0.63461622263629 -0.59687916271176 c
-2.90313049965310 2.14377708712127 -1.75976179692054 h
-2.38146365558044 -1.29452552993475 -1.21496438850812 h
-1.38362521097757 1.04941850969264 1.22218740688745 h
-6.28954630584057 0.31231081353115 1.39022011015995 cl
-8.17193668053100 -0.79561587674850 -1.26002298801238 c
-10.09501595661696 -0.94275039917998 -0.56696395784206 h
-8.01252213633712 0.59894582967433 -2.75241772830741 h
-7.42860652470511 -2.62082380858602 -1.81971985207865 h
$end
E(COSMO) = -970.4021506663 H
ZPE = 420.0 kJ/mol
enthalpy = 454.56 kJ/mol
chem. pot. = 315.35 kJ/mol

$[Me_2Br]^+$/1,2-difluorobenzene pre complex
$coord
-1.59173185327548 -3.59353482930626 0.52779231882701 c
0.75723108694219 -3.49346626553744 1.69913077462211 c
2.95197950960157 -3.41227005451037 0.28188333473324 c
2.77470980706224 -3.44556548950169 -2.34416991077257 c
-1.78172179831781 -3.61567090601062 -2.07890916213938 c
4.75422460754629 -3.33074602761995 1.24056152324517 h
4.48063809434932 -3.38741320471629 -3.46609097351289 h
-3.63187093377881 -3.69051626064665 -2.94209633516373 h
-3.68657534420692 -3.65443173864455 1.97598419397817 f
0.86202379805287 -3.46065984537241 4.24467327755276 f
0.42126050950613 -3.54700942748548 -3.51770576073780 c
0.28990251474988 -3.56818390826210 -5.55597189759411 h
0.36763134846199 3.22899785327689 -1.08596552728291 c
2.02502956488992 4.38746779304843 -0.76972777122630 h
-1.08267516331759 4.12794419853182 -2.21611480122703 h
0.82950582159808 1.34021171728576 -1.73464758393009 h
-1.81921378049632 6.23650963089477 3.24702788906560 c
-2.67953090255942 6.08375488119464 5.10066665480110 h
0.01037546481097 7.15347124529136 3.28228596798594 h
-3.09828909485750 6.96508971434804 1.82489849473405 h
-1.15290325676152 2.67602092374216 2.28649529404164 br
$end
E(COSMO) = -3084.3106534837 H
ZPE = 418.8 kJ/mol
enthalpy = 455.68 kJ/mol
chem. pot. = 308.04 kJ/mol

MeBr/4-methyl-1,2-difluorocyclohexa-2,6-dien-5-yliumcation product complex
$coord
1.54901864882099 -5.61214770037832 -2.03365129125503 c
4.08376218293536 -5.23477239405218 -1.27348544226796 c
5.18080265517065 -2.84216696610578 -1.03999074491497 c
3.71569656403417 -0.79073110767351 -1.55257996933724 c
0.06967551094717 -3.59682404866925 -2.54908643675178 c
7.13261837147954 -2.70118820239093 -0.45812102864922 h
4.49703555721805 1.09164652308393 -1.39136877619545 h
-1.87379722147606 -3.87097052029370 -3.12159504509788 h
0.71684117103885 -7.98110935549200 -2.19222266875814 f

5.43677146600386 -7.23409266437072 -0.78239540008962 f
1.07206976034418 -1.01397260013164 -2.35746750704270 c
1.05437375133063 -0.26896569968153 -4.31793272574227 h
-0.72492936260021 0.78573733382677 -0.86983114547595 c
-0.00720699116581 2.70796630922930 -0.97515460687747 h
-2.60288864477365 0.71770012975270 -1.69967044229694 h
-0.82507150460675 0.19746132553950 1.09663454154531 h
-5.91421974038567 7.38184703107752 5.29389828890461 c
-4.69822571552461 8.35540719773405 6.62319241841755 h
-6.69815680962635 8.66488926953367 3.90363720367569 h
-7.37687944921632 6.31208394142291 6.24773899541236 h
-3.78729019994768 4.93220219803908 3.44945178279695 br
$end
E(COSMO) = -3084.3178570300 H
ZPE = 422.1 kJ/mol
enthalpy = 458.34 kJ/mol
chem. pot. = 307.46 kJ/mol

[Me$_2$Br]$^+$/1,2-difluorobenzene to MeBr/4-methyl-1,2-difluorocyclohexa-2,6-dien-5-yliumcation transition state

$coord
-1.00526061551273 -4.91323047277864 -1.26948174795438 c
0.18578658511013 -5.62992833336855 0.97704886396716 c
2.54637315412729 -4.70537503217577 1.62540381190522 c
3.73146063834338 -3.03137306197601 -0.00067126550521 c
0.14869383151008 -3.25642920423521 -2.89647185352162 c
3.41174311843412 -5.31347922139999 3.37191910826717 h
5.58103322209831 -2.30044186071281 0.46237582407570 h
-0.78257220428869 -2.73217339649225 -4.63709556145291 h
-3.28062473133386 -5.88804101347659 -1.80175312495939 f
-1.00487615793913 -7.23831947726978 2.50551605203721 f
2.52067012462144 -2.23255560544547 -2.23353076780054 c
3.56297510557011 -1.16040310610367 -3.62567175595761 h
1.09318348751973 1.36988739560487 -0.43165314632189 c
2.97702490893003 1.99586068311747 0.00442881440044 h
0.23832192350974 1.89441438209875 -2.19979857583117 h
0.00290433780890 0.36835448620438 0.96234048824177 h
0.65857332393074 7.85340561231413 -1.15904112198807 c
0.24672862546127 9.74178943031780 -0.48152364762039 h
2.67146228807194 7.52159096547335 -1.33068623599482 h
-0.36441707618116 7.40968091931895 -2.87565453652639 h
-0.60134589582557 5.53767641597671 1.48466606122985 br
$end
E(COSMO) = -3084.2884993251 H
ZPE = 417.2 kJ/mol
enthalpy = 452.76 kJ/mol
chem. pot. = 308.24 kJ/mol

[Me$_2$I]$^+$/1,2-difluorobenzene pre complex

$coord
-1.69927770189475 -5.27476462719825 0.36974525678203 c
0.63113223948906 -4.77834132866019 1.47644934261035 c
2.82955569489707 -4.88057030635190 0.06442318703669 c
2.67706491247769 -5.52007363151978 -2.48461278773841 c
-1.86306974366263 -5.90315776084704 -2.16138821641410 c
4.61546613243583 -4.47778495265602 0.97065900685109 h
4.38620546549455 -5.61349695964199 -3.59892703683196 h

-3.69742791995836 -6.28832102781911 -2.97375362844854 h
-3.79784165255791 -5.12415016465466 1.80229884015006 f
0.71521334529523 -4.17283995816943 3.95133783115457 f
0.34442325860960 -6.03386737347322 -3.58905542665606 c
0.23144458185066 -6.53411444628167 -5.56639769535687 h
-0.01196383956687 1.11724936204883 -3.37036890193383 c
1.48111737756280 2.43483311182200 -3.84471356246294 h
-1.77784348682704 1.48365826662018 -4.33958497203405 h
0.58682197911809 -0.83703044050222 -3.53564559303904 h
-0.76428408280351 1.63338336421918 0.59603408940873 i
-1.91963055640630 5.53291451817286 0.32347024214387 c
-2.43303929481245 6.04799817112403 2.24029370763597 h
-0.28882488245687 6.56210407338727 -0.36361717768466 h
-3.51758331637032 5.55786458187137 -0.95661070101980 h
$end
E(COSMO) = -807.9841536595 H
ZPE = 416.1 kJ/mol
enthalpy = 453.36 kJ/mol
chem. pot. = 303.50 kJ/mol

MeI/4-methyl-1,2-difluorocyclohexa-2,6-dien-5-yliumcation product complex
$coord
1.33920863763666 -5.56679304478438 -2.10351112472438 c
4.00114963456343 -5.40271748620562 -1.92776388265558 c
5.30058695589082 -3.10578689650979 -1.79558283605480 c
3.91735457566099 -0.93666325110028 -1.81978828865132 c
-0.06079292119222 -3.43236683254365 -2.12887321778008 c
7.33837134538005 -3.12773378169648 -1.67321967412868 h
4.85582572407043 0.87705562865923 -1.72097136225567 h
-2.09832979939841 -3.54425025829370 -2.25506478739702 h
0.31488118956349 -7.86184736269268 -2.22340614323449 f
5.27475986704285 -7.51175689353714 -1.89947555854277 f
1.15160370431328 -0.93767939642013 -2.00285155081552 c
0.74292103380943 -0.03041902734447 -3.84879400488529 h
-0.12050358943752 0.83630362005051 -0.02036706533810 c
0.69146035590347 2.71765085909230 -0.16404522015392 h
-2.13787063505176 0.94226820858323 -0.39020396789400 h
0.18632836939348 0.10097991010587 1.87362278631864 h
-3.07252046095874 5.24410246028114 4.95936791756945 i
-6.52894874329802 7.44467958549076 4.80227896999065 c
-6.43905876653190 8.80627878511838 6.32875425846450 h
-6.57352006434868 8.35573276830555 2.96914393492554 h
-8.08290641301113 6.13296240544145 5.04075081724306 h
$end
E(COSMO) = -807.9818923994 H
ZPE = 420.4 kJ/mol
enthalpy = 456.84 kJ/mol
chem. pot. = 303.65 kJ/mol

$[Me_2I]^+$/1,2-difluorobenzene to MeI/4-methyl-1,2-difluorocyclohexa-2,6-dien-5-yliumcation transition state
$coord
-1.50826567576781 -6.42994740334275 0.10543002331102 c
0.96602604223740 -6.94330612228253 0.87899280917957 c
3.02995777481693 -5.93024361911251 -0.37418007346574 c
2.60887896976387 -4.36998139150671 -2.42922777507133 c
-1.95171239003244 -4.88701152637598 -1.92823105046926 c

4.91231646849458 -6.38725039389089 0.27133383463687 h
4.19152730687201 -3.57141523975115 -3.44264467577383 h
-3.87087679726057 -4.51986393636121 -2.52295115554375 h
-3.42199881440861 -7.48967723646522 1.37933952484435 f
1.31121631538132 -8.44422056425702 2.86813488334641 f
0.12261934943419 -3.75570248674553 -3.17550713577673 c
-0.19270107127761 -2.82203226539154 -4.96536002291537 h
0.09242891875011 -0.10516394914428 -1.17776899138680 c
1.66795263864955 0.62690724537162 -2.23231904649572 h
-1.80077331571548 0.28282673645224 -1.81058672332927 h
0.38940328687159 -0.91717048843492 0.66257418647156 h
-0.08884213503714 4.62827592926761 1.28620356824330 i
-2.21604470429481 6.49136900669745 -1.67082804780920 c
-2.32624232655523 8.46099596063258 -1.11997429406883 h
-1.16455589458579 6.23808002474032 -3.40890401023498 h
-4.05910031228900 5.60236420348043 -1.72690919691189 h
$end
E(COSMO) = -807.9561531740 H
ZPE = 416.2 kJ/mol
enthalpy = 451.68 kJ/mol
chem. pot. = 305.98 kJ/mol

1,2,3-trifluorobenzene
$coord
2.28801502948160 0.00000000000000 -1.41087615615178 c
2.25727956942908 0.00000000000000 1.20217068761598 c
0.00000000000000 0.00000000000000 2.53734936609071 c
-2.25727956942908 0.00000000000000 1.20217068761598 c
-2.28801502948160 0.00000000000000 -1.41087615615178 c
0.00000000000000 0.00000000000000 -2.70751194701200 c
4.08114935454070 0.00000000000000 -2.38812944317907 h
-4.08114935454070 0.00000000000000 -2.38812944317907 h
0.00000000000000 0.00000000000000 -4.74965124417619 h
-4.43287406856290 0.00000000000000 2.52094614652681 f
0.00000000000000 0.00000000000000 5.07159135547361 f
4.43287406856290 0.00000000000000 2.52094614652681 f
$end
E(COSMO) = -529.9341046636 H
ZPE = 198.5 kJ/mol
enthalpy = 218.75 kJ/mol
chem. pot. = 117.81 kJ/mol

$[Me_2CI]^+$/1,2,3-trifluorobenzene pre complex
$coord
-2.11066889302329 -3.03264220996156 -0.36862591206716 c
-0.42583753641006 -3.48025437435324 1.59499070838651 c
2.10271588066480 -3.89936517453699 1.02523855020271 c
2.95343613189393 -3.87416210996076 -1.44723395008716 c
-1.31284862376869 -2.99395568437136 -2.85747029725640 c
4.93204371467190 -4.20891029074368 -1.82464011848346 h
-4.54692375862241 -2.61644418943308 0.21873148205592 f
-1.22815427643202 -3.50087088264189 3.99678699011864 f
1.22893810332347 -3.42051679350116 -3.38183899426731 c
1.87439156514420 -3.39563896118550 -5.31860036260438 h
1.06397344832332 3.08937424110357 -0.52180308904243 c
2.58027087403565 4.32964544168289 0.07439892227470 h
-0.02984218856449 3.80405433865757 -2.09744733602240 h

1.69071182780192	1.15702976006031	-0.79682234007568	h
-1.11089811811283	2.86422554679915	2.15582090320088	cl
-2.05283225794565	6.15875838189660	2.66237265284372	c
-0.33374708745809	7.17503129978747	3.11541985617149	h
-2.95133120184705	6.76328665420688	0.92445434085706	h
-3.35690669957376	6.04712949882894	4.24014556758737	h
-2.67340429267604	-2.63348823318346	-4.33666749936219	h
3.70691338857515	-4.33228625915079	2.94278992556947	f

$end
E(COSMO) = -1069.6657950631 H
ZPE = 401.4 kJ/mol
enthalpy = 439.52 kJ/mol
chem. pot. = 291.49 kJ/mol

[$Me_2Cl]^+$/1,2,3-trifluorobenzene to MeCl/4-methyl-1,2,3-trifluorocyclohexa-2,6-dien-5-yliumcation transition state

$coord

-3.06063640361632	1.41692548599516	-2.22613438070131	c
1.11223141045630	3.54680698620121	0.20293782268677	c
-1.34916789521382	4.21291261641790	0.86327880370955	c
-3.44063672494728	3.16838713689360	-0.32877663452286	c
-4.65808081629481	0.59537757172568	-3.19534290821263	h
-5.30950007446205	3.75587746725539	0.24426158601274	h
3.07859145105020	4.59005103287109	1.37836226731286	f
-1.65297211983450	5.89732037221583	2.70315202533722	f
-0.60220469439958	0.63332334801959	-2.89193382836872	c
-0.26377568228447	-0.48477651482452	-4.56772517417659	h
-0.61693294051339	-2.86800940420600	-0.41876963395142	c
-2.16144082929975	-3.64919086073759	-1.48562391335503	h
1.30112992473678	-3.23696814203859	-0.98916501042280	h
-0.99075888904408	-1.79014522562929	1.26548760796934	h
1.46072049864838	1.79551913894252	-1.70093853392296	c
3.80296095454590	1.15328494556881	-2.35494046744859	f
-0.51287955296949	-6.62988824074299	2.13465632454270	cl
0.17583840784937	-8.98932496091195	-0.26380664651445	c
0.26399965016121	-10.78576707936241	0.71872355629704	h
-1.35552435948696	-8.94649836557928	-1.62397939890115	h
1.97821911180681	-8.49972280246716	-1.10584991082671	h

$end
E(COSMO) = -1069.6449846757 H
ZPE = 399.1 kJ/mol
enthalpy = 435.88 kJ/mol
chem. pot. = 291.62 kJ/mol

MeCl/4-methyl-1,2,3-trifluorocyclohexa-2,6-dien-5-yliumcation product complex

$coord

-3.38482538975231	0.77293396881671	-2.28799827000506	c
-0.77743512043883	5.42428819667932	-2.54781452318278	c
-3.42539404267351	5.24518439577639	-2.46611882717011	c
-4.74394299393562	2.94263418836913	-2.34493375497420	c
-4.32895945386655	-1.03694772663925	-2.19627605483004	h
-6.78430417169913	2.98255604798726	-2.30245313204760	h
0.31644052755226	7.67189082117223	-2.66717927387837	f
-4.69209298322229	7.35495301618790	-2.51504241419872	f
-0.60102368172632	0.72993990563241	-2.33337867532701	c
-0.05081861383340	-0.28430382312509	-4.07124218609133	h
0.50610725805408	-0.84967134722197	-0.09479582876553	c

-0.27494557115788 -2.74629570030003 -0.17433467054198 h
2.54804735505880 -0.95119009453016 -0.27391179912885 h
0.00754943442285 0.02680660901600 1.69511096929802 h
0.58157407976089 3.23513791119605 -2.49175007443267 c
3.05013008773838 3.33572168356616 -2.56819372023737 f
2.85517980471407 -4.23634018415716 4.99753326920576 cl
4.56463640061401 -7.02139703103626 6.02048529962113 c
5.20855218141064 -6.69490653371420 7.93915171881659 h
3.27662347037818 -8.61404377025427 5.93634210457788 h
6.14890142260151 -7.28695053342115 4.74679984329213 h
$end
E(COSMO) = -1069.6780406515 H
ZPE = 403.0 kJ/mol
enthalpy = 441.35 kJ/mol
chem. pot. = 286.84 kJ/mol

4-methyl-1,2,3-trifluorocyclohexa-2,6-dien-5-yliumcation
$coord
-2.00529333361741 -1.34032467157481 -0.41363620776387 c
0.59972480954493 3.30876640828500 -0.72792317658376 c
-2.04872943478210 3.12731701063364 -0.68472502537807 c
-3.36590811794954 0.82572502143940 -0.53476989205577 c
-2.94924225660538 -3.14907575352205 -0.29891195587407 h
-5.40671992129867 0.86362142449759 -0.52043908044709 h
1.69248364963355 5.55522626491216 -0.87492926115340 f
-3.31731287220510 5.23338188300256 -0.79889520972628 f
0.77927054483339 -1.38179145478342 -0.43258814481881 c
1.32928177436146 -2.38847143625185 -2.17756902413206 h
1.88487925078511 -2.96920043557819 1.79533011119556 c
1.10172354067314 -4.86570576549375 1.70397632622722 h
3.92762518613406 -3.07000998253337 1.61434515114223 h
1.38676805862437 -2.10222052443932 3.59020991770043 h
1.96115371860268 1.12374519906235 -0.60347179160819 c
4.43029540326545 1.22901681234407 -0.63600273672410 f
$end
E(COSMO) = -569.6102582690 H
ZPE = 302.9 kJ/mol
enthalpy = 327.82 kJ/mol
chem. pot. = 214.75 kJ/mol

4-methyl-1,2,3-trifluorocyclohexa-1,3-dien-5-yliumcation
$coord
0.83904361582463 -1.40919660441531 -0.00018592630730 c
2.08361358057330 0.95816609361468 0.01802848644426 c
0.87538392189973 3.32254217783264 0.01778777162455 c
-1.69951975037438 3.36664877378488 -0.00004619456397 c
2.24848754678546 5.41566548969376 0.03446295837105 f
4.55010938925479 0.99097396244240 0.03596111769506 f
-2.90494567967852 5.52333164694063 -0.00119749986488 f
2.37536259441412 -3.78831943319751 0.00218849791560 c
3.62088283131238 -3.84874018046896 -1.63836549044994 h
3.55716296507017 -3.88482061418957 1.68780787925621 h
1.14031422484708 -5.42935363261115 -0.03899122011196 h
-1.73126780199920 -1.32809852524739 -0.01777546096693 c
-2.81382770406872 -3.06116992773608 -0.03225761089104 h
-3.17921805231649 1.04042219540794 -0.01783404402332 c
-4.47183598811057 1.07216016463937 -1.64980981467229 h

-4.48974569343380 1.05978841350961 1.60022655054476 h
$end
E(COSMO) = -569.6169951187 H
ZPE = 300.2 kJ/mol
enthalpy = 325.62 kJ/mol
chem. pot. = 212.27 kJ/mol

4-methyl-1,2,3-trifluorobenzene
$coord
-2.01095197696475 -1.18772444151316 -0.01392540202605 c
0.62463021125639 -1.26864520386724 -0.00240174143826 c
1.88509360446129 1.03608273479329 0.00946456392096 c
0.61507155613896 3.32724646029438 0.00872980207006 c
-2.00632073609738 3.32790425388557 -0.00134595159772 c
-3.33784055035715 1.08383979311326 -0.01266428888956 c
-3.05270186440602 -2.94626997634383 -0.02455598852554 h
-5.37965800691240 1.13068977321062 -0.02169087029020 h
1.90681704658258 5.50982499966815 0.01710068545811 f
4.43381798989923 1.08997390403208 0.02061949662978 f
-3.21864428791539 5.56820551466859 -0.00102340628679 f
2.09501266677373 -3.69894874224357 0.00293393942825 c
3.38822681233566 -3.78876714983554 -1.60094767533181 h
3.23461889375397 -3.87124454735048 1.71405341433309 h
0.82282864145121 -5.31216737251219 -0.09434657745428 h
$end
E(COSMO) = -569.2408597212 H
ZPE = 270.5 kJ/mol
enthalpy = 295.18 kJ/mol
chem. pot. = 183.23 kJ/mol

1,2,3,4-tetrafluorobenzene
$coord
-1.31186114772797 0.00000000000000 -2.19721006997244 c
1.31186114772797 0.00000000000000 -2.19721006997244 c
2.60130765809146 0.00000000000000 0.08767333830251 c
1.31501378087919 0.00000000000000 2.35976982237568 c
-1.31501378087919 0.00000000000000 2.35976982237568 c
-2.60130765809146 0.00000000000000 0.08767333830251 c
2.37041068243037 0.00000000000000 4.10718956182616 h
-2.37041068243037 0.00000000000000 4.10718956182616 h
-5.14333888018899 0.00000000000000 0.03660453164630 f
-2.56191708329083 0.00000000000000 -4.39402718417821 f
2.56191708329083 0.00000000000000 -4.39402718417821 f
5.14333888018899 0.00000000000000 0.03660453164630 f
$end
E(COSMO) = -629.1755247502 H
ZPE = 177.3 kJ/mol
enthalpy = 199.88 kJ/mol
chem. pot. = 93.62 kJ/mol

$[Me_2CI]^+$/1,2,3,4-tetrafluorobenzene pre complex
$coord
-2.18488986516411 -2.98249004848241 -0.40312450660763 c
-0.54518160173094 -3.44504607390978 1.59316835711142 c
1.99791516130193 -3.88255225000001 1.10388782010976 c
2.91783640356702 -3.86588465662108 -1.34141651997350 c
-1.25547354542019 -2.96648872748818 -2.85871621348359 c

4.90266355607508	-4.21438466730007	-1.66681518426905	h
-4.62674591343191	-2.54303258998023	0.06043303447800	f
-1.42546396272234	-3.44674105086650	3.96130897870916	f
1.27356562954242	-3.40477329344540	-3.34247409359900	c
1.94262025175746	-3.37965504624427	-5.27084164494208	h
1.15363408651399	3.14999428933707	-0.60876745183025	c
2.45753065194194	4.71194952490670	-0.37780557948273	h
-0.26435373005994	3.45876301396657	-2.05262460204472	h
2.12088294234199	1.35180127893145	-0.80275144113451	h
-0.54673975497254	2.86190124255076	2.38732159047951	cl
-1.97184371917933	5.99817804882016	2.76569468574851	c
-0.40872005468866	7.31582641376775	2.88191705085042	h
-3.17994066234576	6.27065103941722	1.13500343554285	h
-3.01910094783267	5.83811424826992	4.52071972511019	h
3.54012512887923	-4.32112673046870	3.06937884689540	f
-2.87832005437269	-2.50500396516101	-4.75349628766812	f

$end
E(COSMO) = -1168.9063177589 H
ZPE = 381.3 kJ/mol
enthalpy = 421.41 kJ/mol
chem. pot. = 268.35 kJ/mol

$[Me_2Cl]^+$/1,2,3,4-tetraflurobenzene to MeCl/5-methyl-1,2,3,4-tetrafluorocyclohexa-1,3-dien-6-yliumcation transition state

$coord

1.19813818442210	2.48018342083545	-2.22702604364857	c
0.55345103943093	4.17808222982430	-3.15831107803295	h
3.11344455890256	2.60493397149467	-0.48677239654612	c
4.19970412715639	4.80258659038705	0.11196681406728	f
4.00271131173844	0.42059792332749	0.70711606836234	c
5.84160380289933	0.57201713113471	2.38961462064487	f
2.94948944423944	-1.92096777261165	0.15058953209186	c
3.80406354990215	-3.98509744976735	1.29654720588927	f
1.01780194263228	-2.05577725424069	-1.60892908040351	c
0.01700135675526	-4.29461759925526	-2.14705416638086	f
0.05629966721942	0.12824196944253	-2.75698306003903	c
-1.18417785603878	-0.07628797322989	-4.36693244804101	h
-3.24795306364820	0.46336574944382	-0.14481204152993	c
-3.92927817666769	2.06797009421812	-1.19105044648140	h
-3.85423783637003	-1.41111530213349	-0.65406453842910	h
-2.09845405524641	0.75087952006325	1.50922002899158	h
-6.97192177518199	0.77768001187689	2.55344395273395	cl
-9.44126208550937	0.23490394301911	0.22960692828679	c
-11.21496445449324	0.32207129621668	1.25257145741474	h
-9.30469089568757	1.72368159353172	-1.17112623929880	h
-9.12758774990930	-1.62157309460572	-0.57825444262853	h

$end
E(COSMO) = -1168.8832430307 H
ZPE = 377.6 kJ/mol
enthalpy = 416.65 kJ/mol
chem. pot. = 267.09 kJ/mol

MeCl/(5-methyl-1,2,3,4-tetrafluorocyclohexa-1,3-dien-6-yliumcation product complex

$coord

3.46625171204221	1.11141528437772	-2.00182569721321	c
3.48082020739088	3.14170108859726	-1.77356178425571	h
5.65051431134921	-0.19744626326516	-2.04798470815602	c

7.90112773035165	0.88803619896334	-1.82073908711308	f
5.61817365806750	-2.85972039934535	-2.34950997950027	c
7.74162159765463	-4.07875259818926	-2.38892851630844	f
3.36737243930616	-4.22231625739346	-2.61762526219363	c
3.45507216715451	-6.70159614553282	-2.89821781036288	f
1.13765633615683	-2.91414881782628	-2.57575789573857	c
-0.98451375787475	-4.14274808821638	-2.82870345489798	f
1.00573094271726	-0.16569361272789	-2.25185260041647	c
0.13671964979814	0.56671948567536	-4.00317319946616	h
-0.84410540638930	0.57678007243610	-0.06716686075061	c
-1.03921496508986	2.61964093317681	-0.03893545906487	h
-2.67875715804580	-0.27604330594564	-0.40955729895373	h
-0.09535150747554	-0.07276548611537	1.73143955813947	h
-4.70126732974677	2.06260661662995	5.01093902230214	cl
-7.73713827665390	3.47227097345419	5.73343839041311	c
-7.93508827692936	3.52515511892909	7.77323067089803	h
-7.75515497728962	5.36341730029849	4.94224127212601	h
-9.19046909649401	2.30348790201915	4.88225070051278	h

$end
E(COSMO) = -1168.9127414870 H
ZPE = 381.9 kJ/mol
enthalpy = 422.45 kJ/mol
chem. pot. = 263.03 kJ/mol

5-methyl-1,2,3,4-tetrafluorocyclohexa-1,3-dien-6-yliumcation
$coord

1.10760415325323	2.17274977017047	-0.22734600811322	c
1.11118842757617	4.19921774389199	0.03333908718736	h
3.30065202133194	0.88397701593580	-0.35088900482797	c
5.54719005549813	1.98506807775534	-0.16462731506661	f
3.28264070891965	-1.77388372615014	-0.69222295819746	c
5.41401353122630	-2.97416380976929	-0.79846141898405	f
1.03724749390771	-3.15139376509266	-0.92895415662019	c
1.13855570132074	-5.62480137418048	-1.25179289943287	f
-1.20132507970363	-1.86353448976918	-0.81044054481430	c
-3.31888149859947	-3.10575077279216	-1.03194003785516	f
-1.34839521668828	0.87792376380993	-0.43307965928813	c
-2.27291009739800	1.63446341165564	-2.14503552287159	h
-3.13960718241177	1.56027898364605	1.81916031793523	c
-3.34842062558967	3.60167007195757	1.89111681754306	h
-4.97606426817703	0.69661540766466	1.50881295410149	h
-2.33348812446612	0.88156369126656	3.58236034930450	h

$end
E(COSMO) = -668.8448828622 H
ZPE = 281.5 kJ/mol
enthalpy = 308.72 kJ/mol
chem. pot. = 190.08 kJ/mol

5-methyl-1,2,3,4-tetrafluorocyclohexa-1,3-dien-5-yliumcation
$coord

3.37791238643561	0.51275610223834	0.24878868894068	c
3.41351773995110	-2.04624267731601	0.00979772872621	c
-1.36962838944185	0.54075329140116	-0.05532385008264	c
5.48921378856172	1.77092451090661	0.50075330764675	f
5.52650607155431	-3.38116149278517	0.02146656749474	f
-1.24518467958033	-2.02060810677864	-0.29351792788789	c
-3.31595449929445	-3.42540881302041	-0.55247579989610	f

1.08341012601034	-3.29703735219717	-0.26609950095429	c
1.10299110302445	-5.74048926357811	-0.50266681046070	f
-3.81102964715645	1.91162117501219	-0.02479264347090	c
-5.12347201269548	1.10885782808707	-1.39325135925713	h
-4.67412697265393	1.69370497197662	1.84379060229135	h
-3.54931391754148	3.91717775392737	-0.38401721331251	h
1.02003593824480	1.95989953266843	0.21899420221757	c
1.14328054763095	3.36718559302089	-1.30819558941495	h
0.93184241695067	3.12806694643689	1.93674959741992	h

$end
E(COSMO) = -668.8565410952 H
ZPE = 278.6 kJ/mol
enthalpy = 306.32 kJ/mol
chem. pot. = 187.36 kJ/mol

5-methyl-1,2,3,4-tetrafluorobenzene
$coord

3.34336367417292	0.74291934354424	0.18122725437157	c
3.41293598887834	-1.86924987487687	0.00486649790376	c
-1.21377102214689	0.74862747112952	0.00277818126727	c
1.07724347473379	2.04166177819308	0.18112436356376	c
5.54768706529058	2.00287015055288	0.35407015216836	f
5.61139581575932	-3.12393717432800	0.00495794427788	f
-1.11586472528387	-1.86910284327882	-0.17247069136871	c
-3.27304771386796	-3.21328725833710	-0.34891599540586	f
1.11312593608335	4.07943680922416	0.32143050267188	h
1.15115154864404	-3.18274486145155	-0.17410213675344	c
1.17455952949597	-5.70694669960904	-0.34669414581913	f
-3.71891677189674	2.08598181885663	-0.00132962688771	c
-4.78086564728577	1.65460873100215	-1.71550110352829	h
-4.87424178290557	1.48729370234187	1.59882634573370	h
-3.45475536967150	4.12186890703683	0.10973245780487	h

$end
E(COSMO) = -668.4825368978 H
ZPE = 249.1 kJ/mol
enthalpy = 276.10 kJ/mol
chem. pot. = 158.67 kJ/mol

$[Me_2Cl]^+$/5-methyl-1,2,3,4-tetrafluorobenzene pre complex
$coord

-2.27419346522672	-2.75677262577688	0.05243473668203	c
0.02908820224624	-3.36782032783714	1.14674660940174	c
-2.43967143662287	-1.38128190472986	-2.17882414558352	c
0.11759636950491	-4.69372286298548	3.29431859074126	f
-4.39241841783465	-3.54278681952851	1.22040871729883	f
-4.97765976564425	-0.76611634957099	-3.29194095216456	c
-5.99096529878102	-2.48973878939410	-3.79697557503766	h
-6.15035515637189	0.25351902835121	-1.93662231007506	h
-4.76917902929083	0.38701184045502	-4.98090301663392	h
2.12073968812215	-1.22351153055001	-2.22717051603577	c
4.28887741025030	-0.46054967891079	-3.31326963502008	f
2.25880451128735	-2.59850097836501	-0.00126085404186	c
-0.18031900616145	-0.61911139003595	-3.30809172744308	c
-0.19777419778448	0.45449111196233	-5.04606634913779	h
-0.19449447243694	3.99754054435151	0.76591639051729	c
-0.62838343485523	2.26990118750762	1.77885916332164	h
1.67418914270652	3.98740409929573	-0.06757605833157	h

-1.67799303241462	4.55520605577435	-0.52901341523492	h
4.48605541568310	-3.17539567813624	1.04350225348090	f
-0.12587054399608	6.40369435105602	3.24844004036031	cl
0.60250149577544	9.27921976601100	1.47129746237083	c
0.65021338516784	10.73955955576720	2.90924689587239	h
2.42703058606370	8.96345699335943	0.59636573347999	h
-0.93027419490951	9.52883247101548	0.13620109204593	h

$end
E(COSMO) = -1208.2139317966 H
ZPE = 453.3 kJ/mol
enthalpy = 497.83 kJ/mol
chem. pot. = 335.05 kJ/mol

$[Me_2Cl]^+$/5-methyl-1,2,3,4-tetrafluorobenzene to MeCl/5,6-dimethyl-1,2,3,4-tetrafluorocyclohexa-1,3-dien-6-yliumcation transition state

$coord

-2.29303842654565	-2.95419774552121	0.29657340276332	c
0.06301848660306	-3.80609220781875	1.09718497802384	c
-2.54588425992449	-1.16235452881928	-1.58895998944473	c
0.22723629060807	-5.51967368448597	2.91280519975368	f
-4.34431352248136	-3.94362505188077	1.39983733887967	f
-5.10380547429747	-0.31928751994855	-2.44773330301861	c
-6.24413147497788	-1.94463489796324	-3.00114955585152	h
-6.11004317770717	0.63083098584605	-0.91688423143410	h
-4.95803850590473	0.96277730139984	-4.04690658362487	h
2.06104652754034	-1.06616526524930	-1.86438794620876	c
4.15038028214896	-0.15362671882429	-2.92884040440736	f
2.26867249123863	-2.86275757160031	0.01286436284584	c
-0.30213348985947	-0.13518695884312	-2.61772629273931	c
-0.38735905479106	1.02008755196709	-4.30081635186388	h
-0.21894401558903	3.31688876650565	-0.05230125383541	c
-0.24238262526155	2.16898546581332	1.62693171021696	h
1.55322699307318	3.88307844048354	-0.87587980850501	h
-1.96649934567608	3.95735106567801	-0.87159512842234	h
4.50926591347699	-3.70094402622820	0.78725007046354	f
-0.13359988826110	6.99432162845478	2.60106601296069	cl
0.30465451186351	9.50328871421210	0.29655609959382	c
0.34443186443418	11.24928906074040	1.36954175142901	h
2.08669409457832	9.15996717029914	-0.65376667982119	h
-1.28931681406344	9.44267821183594	-0.98869699908407	h

$end
E(COSMO) = -1208.1931702507 H
ZPE = 449.6 kJ/mol
enthalpy = 493.06 kJ/mol
chem. pot. = 335.05 kJ/mol

MeCl/5,6-dimethyl-1,2,3,4-tetrafluorocyclohexa-1,3-dien-6-yliumcation product complex

$coord

0.93486321773763	-4.85146465564746	-2.50293772508132	c
3.59087474337458	-4.83794755840331	-2.51665994746006	c
-0.41889742409030	-2.66460057603012	-2.39896988880722	c
4.79361218753093	-6.97373675778634	-2.63459877363579	f
-0.18312474786794	-7.10438822454525	-2.61255966654252	f
-3.22101373169092	-2.67824502826437	-2.39019171897031	c
-3.96052255068198	-4.35392977456949	-3.32182018598067	h
-3.88922955181840	-2.71021367106041	-0.43093912315612	h
-3.97447928953381	-0.98431078625486	-3.27988299423327	h

3.73050261034487	-0.37231857868201	-2.31578561244611	c
4.98402253756507	1.75884425313114	-2.25268409537144	f
5.01202133224302	-2.59710095582593	-2.42550797811892	c
0.95542914894573	-0.21829491196087	-2.24324824784905	c
0.40403269515066	0.87870100912369	-3.92341436199737	h
0.09497665785814	1.36295094925538	0.10380876306259	c
0.52954558216172	0.33899724812246	1.83125055387528	h
1.07341641652711	3.16674372003109	0.09824855689703	h
-1.92414369415964	1.70341937531239	-0.01287183169108	h
7.50853780675068	-2.72421337856199	-2.46484000379093	f
-1.87374850829545	4.63628670168936	5.30401433962755	cl
-3.34034528722458	6.98393010690901	7.32064459567907	c
-3.18896673517133	6.32941567256856	9.25742426926508	h
-2.33515977545609	8.75128082068311	7.05875659287695	h
-5.30220364019964	7.16019500076613	6.75276448384851	h

$end
E(COSMO) = -1208.2267758589 H
ZPE = 454.1 kJ/mol
enthalpy = 498.70 kJ/mol
chem. pot. = 331.39 kJ/mol

5,6-dimethyl-1,2,3,4-tetrafluorocyclohexa-1,3-dien-6-yliumcation
$coord

0.10489881218615	-3.05556515932371	-0.65616077171742	c
2.76084022572940	-3.02687319529008	-0.66666953162648	c
-1.26130579932867	-0.87718194328006	-0.53872760786116	c
3.97578770279331	-5.15461806600140	-0.80126490061008	f
-1.00043440640810	-5.31359001917625	-0.78839508810221	f
-4.06330157142789	-0.90657115729688	-0.54497820331203	c
-4.78701315146192	-2.56819906091521	-1.51403929469587	h
-4.74302912510982	-0.98265421387788	1.40899843558686	h
-4.82159380321122	0.79975773718204	-1.40623494967111	h
2.87447782394460	1.43790874148198	-0.43287465251505	c
4.11478663531453	3.57642031577868	-0.35778472540942	f
4.16914771487894	-0.77843579383541	-0.55839118685118	c
0.09899901555784	1.57429969118302	-0.34857710162061	c
-0.47044446142504	2.70525163129469	-1.99865763302881	h
-0.75449799900583	3.09644330041052	2.04377901461367	c
-0.29389293111991	2.03890772038839	3.74419264676333	h
0.20922929245672	4.90882477255041	2.06599642440738	h
-2.77894925330352	3.41686135628953	1.95118640093268	h
6.66629527894055	-0.89098665756235	-0.60139727528262	f

$end
E(COSMO) = -708.1587189642 H
ZPE = 353.4 kJ/mol
enthalpy = 384.88 kJ/mol
chem. pot. = 257.69 kJ/mol

5,6-dimethyl-1,2,3,4-tetrafluorobenzene
$coord

0.27926738809246	-3.13826485390892	0.17467231690146	c
2.90202944459205	-3.18092809764393	0.14733072256673	c
4.20803343476472	-0.92362923447851	0.00847174014009	c
2.86200396914936	1.32477477480835	-0.10302321899743	c
0.24274104327716	1.39425148750655	-0.08133511874599	c
-1.08841535139390	-0.90564799420912	0.06424283131807	c
4.21675319692064	3.48302938821790	-0.23602833357984	f

6.74121579421680	-0.91761901125740	-0.01803303649447	f
4.16135157443013	-5.37664101425115	0.25537534326354	f
-0.91690383921371	-5.38818276683859	0.31518807134874	f
-3.92889564513059	-0.94722776889046	0.09796717756321	c
-4.68916534548614	-0.11108015363836	-1.62798347136714	h
-4.65333090209274	-2.86148891251733	0.25988788063196	h
-4.65435651659078	0.15694283260039	1.68174598300556	h
-1.13900630087626	3.87304724569132	-0.21047132132694	c
-2.40064461012495	3.91808880883284	-1.84149042795918	h
-2.30418033879537	4.14571347377992	1.46994579037771	h
0.16150300426107	5.45486179619672	-0.35646292864620	h

$end
E(COSMO) = -707.7882986168 H
ZPE = 322.1 kJ/mol
enthalpy = 353.06 kJ/mol
chem. pot. = 227.75 kJ/mol

1,2,3,4-tetrafluorocyclohexa-1,3-dien-6-yliumcation
$coord

2.37266442878536	1.43924826840307	0.00000000000000	c
2.49328725949797	-1.20420006636665	0.00000000000000	c
0.26907348893040	-2.52375323993505	0.00000000000000	c
-2.16033955495244	1.50997334178807	0.00000000000000	c
0.04962322392130	2.77805067649926	0.00000000000000	c
-3.94182982221494	2.50861907825911	0.00000000000000	h
0.18919459429177	5.28189011570471	0.00000000000000	f
4.46873577546719	2.70574672531152	0.00000000000000	f
4.71731292777216	-2.33585703333157	0.00000000000000	f
0.30741216730202	-4.98548311749059	0.00000000000000	f
-2.18545214118271	-1.26460083652097	0.00000000000000	c
-3.28984117380900	-1.95481695616052	1.62553493748310	h
-3.28984117380900	-1.95481695616052	-1.62553493748310	h

$end
E(COSMO) = -629.5425176308 H
ZPE = 207.1 kJ/mol
enthalpy = 230.32 kJ/mol
chem. pot. = 120.82 kJ/mol

$[Me_2Cl]^+$/MeCl complex
$coord

-1.54522947259892	0.96231876107205	-0.07822117683925	c
-1.72177323167473	-0.93059147001155	-0.83428858875768	h
0.10257811687449	1.24252572447253	1.10147829501796	h
-3.27373062012690	1.64020689008797	0.78732431158931	h
-1.13234993061059	3.05473953819922	-2.81770096433479	cl
1.80004145444409	1.87317746498459	-4.21462435927506	c
2.08287238929767	3.07700205238938	-5.84957026674554	h
3.25082515449250	2.11455350074954	-2.78951325537880	h
1.44708823022527	-0.08014221000759	-4.71850742232719	h
-4.18166565102906	-1.48986881032891	6.95309350839407	c
-5.79422395075398	-2.74726289256634	7.09496096939281	h
-3.23179483994577	-1.29818838273607	8.75934537812686	h
-4.73797145938160	0.34116559935938	6.21734158998845	h
-1.96106878871953	-2.88345149673957	4.74923697498550	cl

$end
E(COSMO) = -1039.7975255479 H
ZPE = 299.9 kJ/mol

enthalpy = 328.09 kJ/mol
chem. pot. = 201.17 kJ/mol

[Me₂Cl]⁺/MeCl transition state

$coord
-0.52662500053527 -0.01190298882634 0.00069208348257 c
-1.58508412716897 -1.70707050764275 -0.38145665177104 h
1.50614445725943 -0.04179274018784 -0.04766998768279 h
-1.51625434492849 1.71541263939896 0.42111090038089 h
-0.59485176869276 1.02635384896473 -4.33585323042164 cl
0.97265933701034 -1.73491478712020 -5.64886303193124 c
1.07229515677123 -1.41813591771629 -7.67101608276342 h
2.83739009875485 -1.83803570751594 -4.80660789627491 h
-0.17990935283807 -3.36619322077463 -5.19351736057163 h
1.04513553305295 1.77011850918601 5.60926853581475 c
-0.33707081681473 3.27866052026512 5.51436722561681 h
1.54251613624735 1.31155240616619 7.54352189214005 h
2.70734478467393 2.16329939326391 4.47742341998049 h
-0.42802566798657 -1.05365572822647 4.31824163325702 cl
$end
E(COSMO) = -1039.7811630978 H
ZPE = 299.8 kJ/mol
enthalpy = 325.49 kJ/mol
chem. pot. = 208.69 kJ/mol

[Me₂Cl]⁺/MeBr pre complex

$coord
-0.26942577811238 0.47838963481014 -1.28234127893977 c
-0.65658108465314 -1.37076319121949 -2.06792495407680 h
1.34639329930123 0.53233597110183 -0.02845014366731 h
-1.93296958208021 1.37242833919281 -0.48899349091559 h
0.53867645652154 2.51317662712171 -3.97999340895223 cl
3.32157880873151 0.93464215028826 -5.28772325405321 c
3.82696030641574 2.09214136227458 -6.90240572232998 h
4.74259718889816 0.96383133267619 -3.81342878945493 h
2.71372369164680 -0.94725920482938 -5.81960104875702 h
-2.85953270166998 -1.06376816571121 6.19383517578743 c
-4.07932861701945 -2.11657779847337 7.45748462361663 h
-1.23981638789354 -0.28605506401794 7.17610584522945 h
-3.89511520282440 0.37997803972439 5.17523541533750 h
-1.55716039726190 -3.48250003293856 3.66820103117589 br
$end
E(COSMO) = -3153.6803945333 H
ZPE = 300.2 kJ/mol
enthalpy = 328.02 kJ/mol
chem. pot. = 200.46 kJ/mol

[Me₂Cl]⁺/MeBr to MeCl/[Me₂Br]⁺ transition state

$coord
-0.79321355193281 0.69626211595754 -5.74298928894403 cl
1.71315906913821 -1.43677897375829 -6.73314819501504 c
1.79032026372903 -1.29244845035571 -8.77596115446231 h
3.44828322110720 -0.78684953697758 -5.85966333859459 h
1.18698270353891 -3.32055232555844 -6.12378146790749 h
1.27267266444429 2.71768681013674 4.11722374144189 c
1.06670135869998 2.98833839684529 6.13666798407529 h
3.22606161182339 2.44890545381022 3.56717921763444 h

0.34494929033244	4.19515304600100	3.04606240940176	h
-0.52110857012877	-0.46506737236525	3.33654097163410	br
-0.68555410380054	0.11415693157062	-1.41929389627779	c
-2.15830888000897	1.49197798014440	-1.15572366615103	h
-1.16928607472829	-1.85325064319443	-1.60374785297127	h
1.26659836744441	0.68067762517915	-1.34683839165785	h

$end
E(COSMO) = -3153.6660212423 H
ZPE = 296.0 kJ/mol
enthalpy = 323.06 kJ/mol
chem. pot. = 200.10 kJ/mol

MeCl/[Me$_2$Br]$^+$ product complex

$coord

-1.38483745590539	0.44339140814728	-5.17522345577451	cl
0.90458730151504	-1.92437799314802	-6.11196181489708	c
1.05667538974843	-1.87066241318890	-8.15542461130876	h
2.69442754145151	-1.45416800015876	-5.22965645000459	h
0.22117129741017	-3.74875449562125	-5.47363366883889	h
0.40327372756972	2.37170572412455	5.68789388745144	c
0.54423601771316	2.23627295243242	7.72770311453117	h
2.22496471125321	2.53378094928891	4.76865770394512	h
-0.96990250674657	3.75903732513475	5.07175433916942	h
-0.96938477756705	-0.95343507538748	4.66155330662731	br
-1.15594325168460	-0.39329990671275	0.95134389462536	c
-2.46082427132230	1.15955049422063	0.69030447521259	h
-1.86722129454078	-2.17180069483788	0.22819153952454	h
0.75877757110541	0.01275972570655	0.35849773973681	h

$end
E(COSMO) = -3153.6836741088 H
ZPE = 297.0 kJ/mol
enthalpy = 326.14 kJ/mol
chem. pot. = 194.53 kJ/mol

[Me$_2$Cl]$^+$/MeI pre complex

$coord

-0.31279252113030	0.46654819614972	-1.57278936626335	c
-0.49785775362728	-1.43702769666724	-2.29974498949126	h
1.15631481615969	0.68549818723645	-0.16524622228908	h
-2.10344800827936	1.28129211065342	-1.00195561982061	h
0.65000676617364	2.42743561209574	-4.27916003979189	cl
3.62293027809454	0.94958935073938	-5.24041499068248	c
4.21006703902611	2.04616650129995	-6.86984578228208	h
4.90003935129551	1.14136543520929	-3.65128007377131	h
3.16892191538624	-0.99088706262192	-5.71208986058570	h
-3.05064425211036	-1.00976866422550	6.49368233856138	c
-4.39796931093179	-1.91076074490148	7.74471281200453	h
-1.28064945422577	-0.60694924999311	7.44058406878131	h
-3.83784465221866	0.65683102975037	5.60197284093579	h
-2.22707421361214	-3.69933300472500	3.51157488469484	i

$end
E(COSMO) = -877.3446760280 H
ZPE = 299.3 kJ/mol
enthalpy = 327.10 kJ/mol
chem. pot. = 197.83 kJ/mol

$[Me_2Cl]^+$/MeI to MeCl/$[Me_2I]^+$ transition state

```
$coord
   -0.76965583094040     0.66766515499490    -6.71130403596138  cl
    1.75197928535888    -1.42949773312369    -7.74227265751095  c
    1.74236136257212    -1.33546570335309    -9.78940581023238  h
    3.49903254852475    -0.70881459674193    -6.95218856067138  h
    1.29275542959134    -3.30631701460045    -7.06199161767032  h
    0.97561803992636     3.52703211261329     3.24161024293923  c
    0.51824576996152     3.98745643552332     5.18350377426741  h
    2.98677615496525     3.62370711269186     2.87420373932000  h
   -0.11603122480293     4.63903853982821     1.91269256373296  h
   -0.14019985958232    -0.37454125495430     2.70601123429205  i
   -0.49721621476492     0.16605536017676    -2.53876264192541  c
   -2.01628391698928     1.46268135086008    -2.14944103265826  h
   -0.88868000692608    -1.82961382759310    -2.59532710479289  h
    1.42904942216214     0.81127571021214    -2.43902369123543  h
$end
```

E(COSMO) = -877.3331928894 H
ZPE = 295.3 kJ/mol
enthalpy = 322.11 kJ/mol
chem. pot. = 198.51 kJ/mol

MeCl/$[Me_2I]^+$ product complex

```
$coord
    0.05185204542540     0.61508304478526    -7.24898297758901  cl
    1.93397454782122    -1.68173308299097    -8.95415756628785  c
    1.65933010804332    -1.34687080595429   -10.95793832674055  h
    3.89491950540002    -1.40695093935284    -8.42353863956664  h
    1.28031263382339    -3.55087369465281    -8.42344758765111  h
    0.67950242590421     3.90419242041838     3.58614739687062  c
    0.61085050836226     4.24025200824313     5.60729102830270  h
    2.59360208987887     3.83544643468455     2.86297604356366  h
   -0.55508969327799     5.15501968858413     2.53596151783728  h
   -0.84056507656453     0.15528910416052     3.11819220469638  i
   -0.43116862654454     0.03687752181858    -0.94150087090685  c
   -1.55922991884486     1.58263570215007    -1.66507415915064  h
   -1.16698476775864    -1.79794157801266    -1.47938561857892  h
    1.57076822505211     0.22882436029182    -1.31669822902474  h
$end
```

E(COSMO) = -877.3565011208 H
ZPE = 295.5 kJ/mol
enthalpy = 324.38 kJ/mol
chem. pot. = 192.72 kJ/mol

$P(CF_3)_3$

```
$coord
   -0.00000000000000     0.00000000000000    -1.91758940520388  p
    2.61378536549920    -1.74359822575141    -0.12890077771495  c
    0.20310767474458     3.13540363943801    -0.12890077771495  c
   -2.81689304024381    -1.39180541368660    -0.12890077771495  c
    2.55338301441296     4.06937273423644    -0.39689482701746  f
   -1.42269989139021     4.77612625232786    -1.18628491747469  f
   -0.31574950426779     3.04429819244972     2.34864066949273  f
   -4.80087167252302     0.17660818895508    -0.39689482701746  f
   -3.42489672050263    -3.62015737406920    -1.18628491747469  f
   -2.47856481922264    -1.79559618815315     2.34864066949273  f
    2.24748865811002    -4.24598092319158    -0.39689482701746  f
```

2.79431432349043   -1.24870200429660   2.34864066949273 f
4.84759661189280   -1.15596887825869   -1.18628491747469 f
$end
E(COSMO) = -1354.2988114124 H
ZPE = 113.6 kJ/mol
enthalpy = 149.39 kJ/mol
chem. pot. = 11.03 kJ/mol

$[Me_2CI]^+/P(CF_3)_3$ pre complex
$coord
-1.06712297044588   0.20071910611732   5.76297566783424 c
0.09419237312285   1.82179620784379   5.29620745470695 h
-0.37393230423606   -1.57770983007680   5.02048233741905 h
-3.05095386470790   0.50999200026991   5.35105650683582 h
-0.97187118264924   -0.08969778257325   9.20145678514564 cl
2.39847724279174   -0.56338790427978   9.81526367610647 c
2.50562916153435   -0.68901115908561   11.85874962942956 h
2.91016772922550   -2.31487912561138   8.88549616917262 h
3.35246618974172   1.09006252840480   9.07308313983740 h
2.30008725139305   -1.17831120779485   -1.00187630317536 c
-2.61824408826758   -1.57563241058299   -3.34599100205580 c
0.16332886028741   3.11647949181875   -3.58542741078743 c
-2.72080823636738   -3.87035942480360   -2.25545434898945 f
-4.98757946112900   -0.71118353618736   -3.60736116150128 f
-1.64086348839327   -1.85337250231207   -5.66186640101537 f
-1.92077010846130   4.00338573773139   -4.73081165057192 f
1.28813463601172   5.04279672928425   -2.37454097539604 f
1.75547354971119   2.30068320595291   -5.37567047108761 f
4.16673400107873   0.46529351899198   -0.46999838363377 f
2.94904961750921   -2.46752846630089   -3.07022945653908 f
2.15900816015518   -2.83719043387046   0.93094604944368 f
-0.81825392812045   0.64488286158017   -1.13824251608722 p
$end
E(COSMO) = -1894.0266534562 H
ZPE = 316.4 kJ/mol
enthalpy = 370.37 kJ/mol
chem. pot. = 179.63 kJ/mol

$[Me_2CI]^+/P(CF_3)_3$ to $MeCl/[MeP(CF_3)_3]^+$ transition state
$coord
-0.64478695343174   0.95104387164974   4.04900918248671 c
1.28734608888893   1.59353126724308   4.07862687085077 h
-1.08796070441263   -1.03062633162845   4.22497769392059 h
-2.14114425687520   2.28169910141041   3.68173115855842 h
-1.02407195389762   1.52119420449843   8.31624570398120 cl
1.32128408588507   -0.72427258605035   9.44762893997144 c
1.31303473805862   -0.54875669900888   11.48944699249521 h
0.72134044983673   -2.58839865057104   8.84563485456134 h
3.12607780340812   -0.17590718081375   8.64800021591326 h
1.54940268885132   -2.90287499431947   -1.08512640354266 c
-3.06703689225909   -0.22529120387084   -2.67404558106830 c
1.83998493505943   2.43986813895200   -2.61512016085781 c
-4.22428693852172   -2.36031052058394   -1.97366495718896 f
-4.63245932550779   1.72053509817596   -2.31086621504269 f
-2.51015230005621   -0.36591876731365   -5.12760112499314 f
0.43369885951603   4.43088538362901   -3.27091567324090 f
3.80275813588559   3.22903252383310   -1.23452476889271 f

```
   2.71829164328377    1.33889307629770   -4.70536999654188  f
   3.98423736097972   -2.57695620059880   -0.47599591893082  f
   1.40493272886961   -3.79664782205086   -3.43173304024552  f
   0.53944587022051   -4.60678621776893    0.49333353302257  f
  -0.16329197636013    0.22983488423407   -0.57731494386423  p
$end
```

E(COSMO) = -1894.0049365533 H
ZPE = 313.1 kJ/mol
enthalpy = 366.08 kJ/mol
chem. pot. = 180.37 kJ/mol

MeCl/[MeP(CF$_3$)$_3$]$^+$ product complex

```
$coord
  -0.96958212938615    0.26807225089679    2.56428486184604  c
   0.39437279941145    1.50997254251111    3.48101584709820  h
  -0.90870025403529   -1.61990734375044    3.38647438367977  h
  -2.86545730714424    1.05642351560577    2.74296449534333  h
  -1.10234104633289    0.87496904180131    8.97715660549791  cl
   1.95537190284853   -0.37487518548729    9.88003640659931  c
   2.12300863821612   -0.20661344153580   11.91598968881008  h
   2.03010029673102   -2.33955010772004    9.29868763029527  h
   3.39549468329095    0.73555644519804    8.93298421848887  h
   3.12649665075787   -1.23924475855179   -1.18014098454581  c
  -2.44373464904373   -2.14618648435811   -2.47901770398435  c
  -0.33990686627668    3.25042127462653   -2.34064736151137  c
  -1.93915222774981   -4.50792523311745   -1.79939032035113  f
  -4.79710619775715   -1.55040396137057   -1.85809554392662  f
  -2.10383786091257   -1.86671098167613   -4.94608754757873  f
  -2.74811427074847    3.91946426117176   -2.55827219352511  f
   0.89705031941930    4.91497743437597   -0.93350399414671  f
   0.71686232808482    3.08040919324377   -4.60642735837098  f
   4.75871613706228    0.54209754030861   -0.49630000699202  f
   3.46651215089085   -1.84847192333030   -3.58451156371041  f
   3.40938999306933   -3.26287440866661    0.27597932650932  f
  -0.20630124806673    0.04600402451276   -0.68988126050480  p
$end
```

E(COSMO) = -1894.0512600713 H
ZPE = 314.6 kJ/mol
enthalpy = 369.39 kJ/mol
chem. pot. = 176.29 kJ/mol

[MeP(CF$_3$)$_3$]$^+$

```
$coord
   0.00000000000000    0.00000000000000   -0.20073307180163  p
   2.99191455534240    1.49797735993008    1.14720164165633  c
  -2.79324372566457    1.84208533091389    1.14720164165633  c
  -0.19867082967783   -3.34006269084395    1.14720164165633  c
   0.00000000000000    0.00000000000000   -3.54487418605717  c
  -1.66841934477985   -1.01784430009498   -4.20198224508443  h
  -0.04726934858958    1.95381568679222   -4.20198224508443  h
   1.71568869336942   -0.93597138669724   -4.20198224508443  h
   2.72300113353208    1.88024746034860    3.60881432650833  f
   0.26684149929699   -3.29831188634689    3.60881432650833  f
  -2.98984263282910    1.41806442599833    3.60881432650833  f
  -4.87557502128860    1.08808211792741   -0.02751718359643  f
  -2.51125516136462   -4.21765063881813    0.72189341920067  f
   1.49548075511560   -4.76641288545641   -0.02751718359643  f
```

4.90822017818649	-0.06598544571748	0.72189341920067	f
-2.39696501682181	4.28363608453559	0.72189341920067	f
3.38009426617298	3.67833076752901	-0.02751718359643	f

$end
E(COSMO) = -1393.9845428342 H
ZPE = 216.4 kJ/mol
enthalpy = 257.02 kJ/mol
chem. pot. = 108.86 kJ/mol

PF_3
$coord

-0.00000000000000	0.00000000000000	-1.12715514069517	p
-1.29845499870669	2.24899002910176	0.37569638140390	f
-1.29845499870669	-2.24899002910176	0.37569638140390	f
2.59690999741333	0.00000000000000	0.37569638140390	f

$end
E(COSMO) = -641.0037880711 H
ZPE = 21.50 kJ/mol
enthalpy = 34.65 kJ/mol
chem. pot. = -47.21 kJ/mol

$[Me_2Cl]^+/PF_3$ pre complex
$coord

-0.63633618724142	1.14239181165750	-0.75486665533527	c
-0.89264826040884	-0.82445802110260	-1.26118758204035	h
0.83618640590520	1.46338902088325	0.63060926802621	h
-2.39541753797434	2.08176576989144	-0.28318584619206	h
0.39694118741338	2.75002046759692	-3.64316097433791	cl
3.37341874366153	1.14736471270734	-4.36761146318393	c
4.00810772973768	2.05727140217463	-6.09153506880945	h
4.61548650239405	1.50080626745741	-2.77817986363600	h
2.90788227464783	-0.82855074452530	-4.63771221061837	h
-4.67491471429694	-0.77603701095827	6.71477829673964	f
-4.11054387263810	-4.72868133297112	4.64431073923370	f
-0.71313843847085	-2.89331749960040	6.95107601555665	f
-2.71502383272933	-2.09196484321082	4.87666534459717	p

$end
E(COSMO) = -1180.7336967125 H
ZPE = 224.6 kJ/mol
enthalpy = 255.41 kJ/mol
chem. pot. = 121.14 kJ/mol

$[Me_2Cl]^+/PF_3$ to MeCl/$[MePF_3]^+$ transition state
$coord

-0.56003080230850	0.02544089033023	-1.71138014916596	c
0.53090190880697	-1.69431128907402	-1.72917529489872	h
0.38145469940719	1.83115697903880	-1.70295092283358	h
-2.57167825558428	-0.06220671235951	-1.40884359275605	h
-1.03440428212054	0.02795387733696	-5.92922847751214	cl
2.25038499339827	-0.01055522680812	-6.94762238680872	c
2.18912400146756	-0.06237335806971	-8.99529642104405	h
3.12072882125219	1.70849637541277	-6.25264091672549	h
3.10608379581680	-1.70053078282308	-6.16770990637650	h
-2.29311196325482	0.91986615003272	4.70983651016458	f
0.52037581331741	-2.59447824880768	4.31932535973437	f
2.13685102967098	1.62239274270600	4.16547320962080	f
-0.04918683399156	-0.00207215294513	3.03642811721077	p

$end
E(COSMO) = -1180.7161849712 H
ZPE = 223.0 kJ/mol
enthalpy = 252.11 kJ/mol
chem. pot. = 124.81 kJ/mol

MeCl/[$MePF_3$]$^+$ product complex

$coord

-0.31719976101327	0.07523762958525	-0.34323269509123	c
1.46357182146025	-0.63789750880570	-1.09249023582624	h
-0.63611155024198	2.01871365760234	-0.94443724097925	h
-1.88518048503054	-1.12771103650933	-0.92275625860666	h
-1.02404460106646	0.97704152677654	-6.67391947551243	cl
2.16019144017251	-0.17646803945012	-7.17038201035874	c
2.59419651840779	0.01696702367340	-9.16435798157141	h
3.43029377756232	0.96386194003690	-6.03379908870505	h
2.21532082838904	-2.14443827582772	-6.59731350951602	h
-2.51347989873005	0.95862065147675	4.19873684414341	f
0.32699503573965	-2.58907655380492	3.98530934661296	f
1.97719013894090	1.65010253778080	3.95618710921545	f
-0.13319936857481	0.02373811658796	2.92712500401788	p

$end
E(COSMO) = -1180.7775677172 H
ZPE = 226.9 kJ/mol
enthalpy = 256.72 kJ/mol
chem. pot. = 124.59 kJ/mol

[$MePF_3$]$^+$

$coord

-0.00000000000000	0.00000000000000	1.45831898393350	p
-1.31320135025189	2.27453145920433	2.57973023514718	f
-1.31320135025189	-2.27453145920433	2.57973023514718	f
2.62640270050377	0.00000000000000	2.57973023514718	f
-0.00000000000000	0.00000000000000	-1.81661533796142	c
0.97908842385820	-1.69583089522493	-2.46029811713787	h
-1.95817684771637	0.00000000000000	-2.46029811713787	h
0.97908842385820	1.69583089522493	-2.46029811713787	h

$end
E(COSMO) = -680.7100058177 H
ZPE = 126.9 kJ/mol
enthalpy = 143.53 kJ/mol
chem. pot. = 53.98 kJ/mol

NC_5F_4I

$coord

0.00000000000000	0.00000000000000	-2.09734291699559	c
2.24418715692416	0.00000000000000	-0.73036352038678	c
2.12127184307240	0.00000000000000	1.88790052132779	c
-2.12127184307240	0.00000000000000	1.88790052132779	c
-2.24418715692416	0.00000000000000	-0.73036352038678	c
-4.26691404306162	0.00000000000000	3.21390000792119	f
-4.48863308257502	0.00000000000000	-1.88190867074750	f
4.48863308257502	0.00000000000000	-1.88190867074750	f
4.26691404306162	0.00000000000000	3.21390000792119	f
0.00000000000000	0.00000000000000	3.15820480113705	n
0.00000000000000	0.00000000000000	-6.03991856037077	i

$end

E(COSMO) = -942.3366496984 H
ZPE = 119.5 kJ/mol
enthalpy = 145.74 kJ/mol
chem. pot. = 25.80 kJ/mol

$[Me_2Cl]^+/NC_5F_4I$ pre complex

```
$coord
  -3.92326701306712   -0.52224897422803    1.11795358322606  c
  -5.32556514723404   -1.53004206568443    3.08870885183164  c
  -4.06877953545334   -2.32022989262512    5.25763342523044  c
  -1.45542904844036   -2.04304651542413    5.30554218503364  c
  -0.26149463888695   -1.00806341436476    3.21554243313069  c
  -1.46321204656929   -0.27061274371653    1.18312231447966  n
   2.24602358559912   -0.72933203707069    3.23623929993174  f
  -5.10541303146022    0.24453445975322   -0.97396025236789  f
  -7.82971850542581   -1.72258376872969    2.87287018445399  f
  -0.09193500960852   -2.74819306851880    7.30522295142091  f
   1.32551916102691    1.94686504375680   -3.14592866206949  c
   1.18173264117673   -0.05479678197655   -3.53987683489945  h
   2.41897732144976    2.39860188893792   -1.47775065419066  h
  -0.48265290402493    2.90572402446226   -3.19373914252882  h
   3.05324680399803    3.33058146546487   -5.82913848103927  cl
   6.08582912561217    1.68103972154957   -5.68608121025845  c
   7.13999984437789    2.45301170221874   -7.26563663190012  h
   6.91381336721891    2.15678923737651   -3.87436064268249  h
   5.66615325232650   -0.31081377814096   -5.91096228097984  h
  -6.02382822261554   -3.85718450304010    8.31459956417780  i
$end
```

E(COSMO) = -1482.0690356021 H
ZPE = 321.5 kJ/mol
enthalpy = 366.15 kJ/mol
chem. pot. = 195.11 kJ/mol

$[Me_2Cl]^+/NC_5F_4I$ to $MeCl/[MeNC_5F_4I]^+$ transition state

```
$coord
  -2.36752156449527    0.07655812075697   -1.65091268342147  c
  -2.34130290497655    0.01088846770325    0.96538523442412  c
  -0.03180981352405    0.05064643892786    2.22142674557703  c
   2.14403164295279    0.15539839573412    0.74885360536207  c
   1.91341581225724    0.21341756979633   -1.85713502998125  c
  -0.28871956742531    0.17692455056574   -3.01435238544095  n
   3.96049567037909    0.31165709599550   -3.30187968991075  f
  -4.54570041877383    0.04006427829029   -2.89072073092960  f
  -4.51993957723701   -0.08870019353568    2.21454647757349  f
   4.43483862820303    0.19925911156350    1.78201998983779  f
  -0.48462038948550    0.24981113928829   -7.14295183309354  c
  -0.45401554243502   -1.78118204074437   -7.07435343683215  h
   1.25114631727653    1.30626730162864   -7.11896883805608  h
  -2.25340575789808    1.23733089287972   -6.96918535544016  h
  -0.68254503624143    0.29570308037012  -11.38820745646760  cl
   2.22550362589684   -1.36714769594627  -12.16034017915656  c
   2.28347435416678   -1.46068143503341  -14.20650846956097  h
   3.77574293170209   -0.26098802121423  -11.40556514787634  h
   2.10177665192181   -3.22959195250138  -11.31615067021018  h
   0.16084942564817   -0.04273700329461    6.14989734793647  i
$end
```

E(COSMO) = -1482.0570305965 H

ZPE = 321.2 kJ/mol
enthalpy = 363.96 kJ/mol
chem. pot. = 201.08 kJ/mol

MeCl/[MeNC$_5$F$_4$I]$^+$ product complex

```
$coord
  -2.77599818507812    0.30207421524760    2.73452614012826  c
  -2.70350262239649    0.24741760906548    5.33908278476068  c
  -0.39693973509395    0.25948242875862    6.60254229952493  c
   1.77899292197409    0.32487355974709    5.12655741865083  c
   1.60292875931875    0.37731355205438    2.52703567652013  c
  -0.64834510924304    0.38263106560157    1.34013103315282  n
   3.62324461220929    0.42139869186820    1.10637648839229  f
  -4.92115846773428    0.27524091546965    1.51151934399924  f
  -4.87745368795330    0.17795050970826    6.57216606137009  f
   4.06080242861062    0.33262357928961    6.14879532731167  f
  -0.78465217646019    0.42639372058880   -1.47237773664126  c
  -0.80189149198918   -1.50971355746778   -2.15023192424915  h
   0.84684555461855    1.43759500562518   -2.17766874088337  h
  -2.49871918011277    1.39965125433254   -2.01754882005581  h
  -0.94250108956437    0.49000857900072   -7.81841415491892  cl
   2.00593028905731   -1.15282519722090   -8.39054360568488  c
   2.19330211378656   -1.41577656858944  -10.41481170589916  h
   3.52818817047166    0.00931901295910   -7.65862349608628  h
   1.92119253454799   -2.95774311742386   -7.42164824293771  h
  -0.21026563896906    0.17208474138515   10.51313585354565  i
$end
```

E(COSMO) = -1482.1027275993 H
ZPE = 327.3 kJ/mol
enthalpy = 371.77 kJ/mol
chem. pot. = 199.14 kJ/mol

[MeNC$_5$F$_4$I]$^+$

```
$coord
   3.82611986786914    0.00042783111210    0.00000000000000  c
   2.44240474920006   -2.23790857137076    0.00000000000000  c
  -0.16018159025949   -2.16552494829211    0.00000000000000  c
  -0.14150458220348    2.21952445839645    0.00000000000000  c
   2.46691656608226    2.25068562098977    0.00000000000000  c
  -1.43677452681822    4.32143092457801    0.00000000000000  f
   3.60955292561297    4.47496553018088    0.00000000000000  f
   3.55865276026342   -4.47476883504005    0.00000000000000  f
  -1.51491420402654   -4.23094038525708    0.00000000000000  f
   7.74216386961531   -0.02156367026763    0.00000000000000  i
  -4.25584933639165   -0.02814618902740    0.00000000000000  c
  -4.87055811149920   -1.01739535318928   -1.68899209167588  h
  -4.87055811149920   -1.01739535318928    1.68899209167588  h
  -4.95223105468931    1.89302385733541    0.00000000000000  h
  -1.44323922125604    0.03358508304105    0.00000000000000  n
$end
```

E(COSMO) = -982.0347429190 H
ZPE = 226.9 kJ/mol
enthalpy = 257.87 kJ/mol
chem. pot. = 126.45 kJ/mol

NC_5F_5

$coord
-2.12894023509184	0.00000000000000	-1.76122167788659	c
-2.26491957747676	0.00000000000000	0.85410839222701	c
0.00000000000000	0.00000000000000	2.18045570997674	c
2.26491957747676	0.00000000000000	0.85410839222701	c
2.12894023509184	0.00000000000000	-1.76122167788659	c
0.00000000000000	0.00000000000000	-3.02175284453659	n
4.26848649923801	0.00000000000000	-3.09050040918762	f
-4.26848649923801	0.00000000000000	-3.09050040918762	f
-4.46600322017884	0.00000000000000	2.07670173720560	f
0.00000000000000	0.00000000000000	4.68312104984302	f
4.46600322017884	0.00000000000000	2.07670173720560	f

$end

E(COSMO) = -744.4708147403 H

ZPE = 126.5 kJ/mol

enthalpy = 150.81 kJ/mol

chem. pot. = 39.70 kJ/mol

/home/haemmers/transP/Me2ClNC5F5

$[Me_2Cl]^+/NC_5F_5$ to $MeCl/[MeNC_5F_5]^+$ transition state

$coord
-3.96261230009506	-0.54926100380518	1.16924343028384	c
-5.37663602134386	-1.55782030237363	3.12810364223730	c
-4.08046733706562	-2.33379878260613	5.27588402720827	c
-1.46937105564236	-2.07095437083777	5.37020797019387	c
-0.28982392373162	-1.03186965054817	3.27690348422118	c
-1.50187474373004	-0.29772138315934	1.24812178115604	n
2.21367627061152	-0.74723403751700	3.29319239541494	f
-5.13614034301332	0.21798363509986	-0.92332796511476	f
-7.87749173941519	-1.78684858306449	2.98704304226154	f
-5.32068091749482	-3.31287446596327	7.21307155932544	f
-0.18011965246254	-2.79759407511117	7.40418861998969	f
1.29067496128810	1.92989747920538	-3.09439273512799	c
1.14266531287285	-0.07231495873002	-3.48444590674863	h
2.38458593219798	2.38175054196652	-1.42637108475920	h
-0.51574740738269	2.89214007413465	-3.14381000367665	h
3.02199525366774	3.30394823916312	-5.77873806637944	cl
6.04946565272340	1.64519813317471	-5.63112737541431	c
7.10814531397248	2.41332313426956	-7.20954865516039	h
6.87610266165529	2.11941108096579	-3.81840622344320	h
5.62365408238788	-0.34536070426361	-5.85579193646767	h

$end

E(COSMO) = -1284.2030207190 H

ZPE = 329.1 kJ/mol

enthalpy = 371.42 kJ/mol

chem. pot. = 210.96 kJ/mol

$[Me_2Cl]^+/NC_5F_5$ to $MeCl/[MeNC_5F_5]^+$ transition state

$coord
-2.25871683935557	0.03169652279088	1.24383240725456	c
-2.16606775720358	-0.06878660335718	3.85532120940142	c
0.20223223230078	-0.07301743645303	4.99245591338066	c
2.35772353988412	0.02201159282724	3.49335502984749	c
2.02930892140303	0.11704954006060	0.90111031922723	c
-0.21534672398607	0.12372241996623	-0.17615467648858	n
4.02340391240904	0.20754581035365	-0.60920795533395	f

-4.46925066510121 0.03979036394554 0.07107929558345 f
-4.25475179773833 -0.15943385781396 5.23834257624586 f
0.40267529166813 -0.16662271335833 7.47356485862801 f
4.64176627101817 0.01992814226224 4.52624840354851 f
-0.53779854635798 0.26736930070022 -4.28003207133871 c
-0.50367965769547 -1.76448058800235 -4.25583593967059 h
1.19572629049454 1.32765250851158 -4.29827594955785 h
-2.30208410993630 1.24904547280048 -4.04124971354428 h
-0.86855413345543 0.39250222235033 -8.52860317732157 cl
2.04984620504152 -1.18733296233457 -9.42534709711232 c
2.04849086717070 -1.23299708587416 -11.47397121187701 h
3.59849253666302 -0.06482744250399 -8.69161637253126 h
1.99197038573960 -3.07101226513801 -8.62233103596283 h
$end
E(COSMO) = -1284.1906191687 H
ZPE = 327.8 kJ/mol
enthalpy = 368.59 kJ/mol
chem. pot. = 214.73 kJ/mol

MeCl/[MeNC$_5$F$_5$]$^+$ product complex
$coord
-2.32198586291784 0.00129491581923 1.38481279908570 c
-2.16046819190569 -0.08891871972632 3.98404901489827 c
0.21524267469686 -0.06247474271356 5.10582486788025 c
2.35690184345156 0.04979655208377 3.58699286404534 c
2.05550091771834 0.13553342576165 1.00084316530296 c
-0.24734192288762 0.12801245904799 -0.08648036150349 n
4.00938114406562 0.22615512622545 -0.50012554581172 f
-4.51124604580246 -0.03608946088787 0.25345569665431 f
-4.23136849933088 -0.20462594416765 5.36470692992762 f
0.43425651010522 -0.15051097472673 7.56688981604860 f
4.63964133207806 0.06823732078205 4.58538350170228 f
-0.48889016986019 0.20923366134664 -2.89565927939960 c
-0.41645076652621 -1.71343685708939 -3.60588091964604 h
1.05383947119173 1.32933436174714 -3.63628884318940 h
-2.27510055313681 1.08874489808914 -3.36030384416743 h
-0.92056762470093 0.39469424213272 -9.21945409995121 cl
2.04652860006614 -1.12161353027274 -10.00566328901263 c
2.16393355066020 -1.24473130294981 -12.04845130955779 h
3.56406861702799 0.03331699808719 -9.25285392685317 h
2.04568191445179 -2.98782791757769 -9.15676301392436 h
$end
E(COSMO) = -1284.2348579072 H
ZPE = 336.4 kJ/mol
enthalpy = 378.01 kJ/mol
chem. pot. = 217.44 kJ/mol

[MeNC$_5$F$_5$]$^+$
$coord
-0.06913972885531 -2.17207126289841 0.00000000000000 c
2.53230608132830 -2.26409902246921 0.00000000000000 c
3.87173686514306 -0.00156421054387 0.00000000000000 c
2.55921896316173 2.27336920054527 0.00000000000000 c
-0.04824550362310 2.22496112593508 0.00000000000000 c
-1.42384626721800 -4.23172713156481 0.00000000000000 f
3.71393712850763 -4.45707500843225 0.00000000000000 f
6.34400218410371 -0.01667454071423 0.00000000000000 f

3.76843655168690 4.45212199707694 0.00000000000000 f
-1.34085694489662 4.32321081302511 0.00000000000000 f
-4.16080544697616 -0.02743530364836 0.00000000000000 c
-4.85512062749183 1.89448772760175 0.00000000000000 h
-4.77385085877403 -1.01566862335716 -1.68985435275253 h
-4.77385085877403 -1.01566862335716 1.68985435275253 h
-1.34392153732225 0.03383286280137 0.00000000000000 n
$end
E(COSMO) = -784.1667443792 H
ZPE = 235.2 kJ/mol
enthalpy = 263.83 kJ/mol
chem. pot. = 142.43 kJ/mol

$N_3C_3F_3$
$coord
-1.19485458287165 2.06954884519022 0.00000000000000 c
-1.19485458287165 -2.06954884519022 0.00000000000000 c
2.38970916574332 0.00000000000000 0.00000000000000 c
-2.43620426143100 -4.21962955841431 0.00000000000000 f
-2.43620426143100 4.21962955841431 0.00000000000000 f
4.87240852286201 0.00000000000000 0.00000000000000 f
1.29092931060605 -2.23595515494956 0.00000000000000 n
1.29092931060605 2.23595515494956 0.00000000000000 n
-2.58185862121213 0.00000000000000 0.00000000000000 n
$end
E(COSMO) = -578.1103723863 H
ZPE = 109.0 kJ/mol
enthalpy = 127.40 kJ/mol
chem. pot. = 31.84 kJ/mol

$[Me_2Cl]^+/N_3C_3F_3$ pre complex
$coord
2.47286344344696 -0.01153269599390 -3.82740627219348 c
0.37470507727976 -3.48649321845747 -3.00013816403742 c
3.18559552472681 -3.43563659200813 -6.03965249422789 c
-1.32595293825569 -4.69971739493798 -1.65913202287737 f
2.94743686538400 2.37693697932914 -3.33632055563310 f
4.40747451519309 -4.60414102073141 -7.85295076292106 f
1.51287984756978 -4.79392936187785 -4.78622098289253 n
3.77517614648147 -1.03977374154647 -5.68141606060074 n
0.74353271861918 -1.09603248461492 -2.39760755301778 n
-2.28606858135870 2.47675754026101 6.96429835727060 c
-3.11633391393305 0.60799892312633 6.85269821774178 h
-0.23904770455962 2.47414294250257 7.02444295640919 h
-3.12230835820350 3.63143764865583 8.43719303295951 h
-3.16430907891309 4.10061072956696 4.04291018106928 cl
-1.70360860384154 2.09653605465486 1.60906052589009 c
-2.20469087986508 3.02695717397684 -0.14465139101280 h
0.30603283403523 2.12472131964810 1.98830078729834 h
-2.56337691380622 0.25115719844644 1.80659220077532 h
$end
E(COSMO) = -1117.8420146940 H
ZPE = 312.3 kJ/mol
enthalpy = 348.45 kJ/mol
chem. pot. = 201.40 kJ/mol

$[Me_2Cl]^+/N_3C_3F_3$ to $MeCl/[MeN_3C_3F_3]^+$ transition state

```
$coord
   0.04382413003357     2.11480555879995    -2.19797685480042  c
  -0.22645875903132    -2.06841518502577    -2.07820416229819  c
   0.15119457205151    -0.09388961231466    -5.70383671814688  c
  -0.44860981156970    -4.14147805636503    -0.75517655128637  f
   0.09501547702199     4.26825977837990    -0.99202480108679  f
   0.31906115565482    -0.17531076017233    -8.16380308829831  f
  -0.06768653377984    -2.29100236330102    -4.53886873240607  n
   0.22027117800955     2.17592839890737    -4.66675596153360  n
  -0.18413270895998     0.06831382705223    -0.76881136940258  n
   2.11271205774201    -1.22632686366761     8.47489442237168  c
   1.91600150590287    -3.13071881147678     7.74611649933940  h
   3.71133584593154    -0.23509843131978     7.66346447954741  h
   2.15766505155534    -1.19580506722864    10.52326957976737  h
  -0.71990337950540     0.50445037820839     7.58021534653225  cl
  -0.44981329188893     0.27371841075776     3.30889113354270  c
  -2.19210066389469     1.29696902073529     3.08016281244039  h
   1.32373481949407     1.26611739724698     3.29538253292116  h
  -0.48013947975316    -1.75826529162654     3.34713750279023  h
$end
```

E(COSMO) = -1117.8283760645 H
ZPE = 310.6 kJ/mol
enthalpy = 345.28 kJ/mol
chem. pot. = 204.67 kJ/mol

$MeCl/[MeN_3C_3F_3]^+$ product complex

```
$coord
   0.04005304430946     2.19612612053287    -2.28390486221331  c
  -0.23997646534316    -2.09716643336424    -2.04939092966613  c
   0.14330519959632    -0.15114797848295    -5.71374507659157  c
  -0.46186857779746    -4.10142320322668    -0.68107701420678  f
   0.08604556729681     4.32901783627479    -1.10500263243619  f
   0.30902739012889    -0.29168522473115    -8.14946917835416  f
  -0.07704864626216    -2.31496605157962    -4.48515791082097  n
   0.21293658722503     2.14506770231880    -4.72418897561705  n
  -0.19475136381903     0.12915962282306    -0.79173646561674  n
   2.18208407755935    -1.16225402892882     9.00228547652532  c
   2.03672097547211    -3.03040559518471     8.17023800423759  h
   3.72872304048780    -0.10166179078435     8.17382396970256  h
   2.38070519271856    -1.27997665939175    11.03906946289828  h
  -0.72478309728037     0.51823223640138     8.33184078684308  cl
  -0.39131194865125     0.36767682982745     2.00591169092982  c
  -2.06364037098623     1.47461621893623     2.43166950510448  h
   1.30209055581349     1.30421162220689     2.68257852160184  h
  -0.55095056172743    -1.50563838324016     2.80490091280555  h
$end
```

E(COSMO) = -1117.8712507004 H
ZPE = 316.9 kJ/mol
enthalpy = 353.13 kJ/mol
chem. pot. = 203.49 kJ/mol

$[MeN_3C_3F_3]^+$

```
$coord
   0.63981853156088     2.17913369090099     0.00000000000000  c
   0.61473743271864    -2.13013665993227     0.00000000000000  c
   4.18822447298945    -0.00542204091949     0.00000000000000  c
```

-0.69334685349152	-4.18682458773677	0.00000000000000	f
-0.60755346591904	4.27221284370346	0.00000000000000	f
6.63352521817212	-0.02309609512168	0.00000000000000	f
3.05919367210505	-2.23741268961845	0.00000000000000	n
3.08994001006522	2.23813135401465	0.00000000000000	n
-3.56445982301526	-0.02340292186672	0.00000000000000	c
-4.25098265992170	1.90239273493435	0.00000000000000	h
-4.17772114416574	-1.00977249090429	-1.69109694263308	h
-4.17772114416574	-1.00977249090429	1.69109694263308	h
-0.75365424693234	0.03396935345041	0.00000000000000	n

$end
E(COSMO) = -617.8032586206 H
ZPE = 217.0 kJ/mol
enthalpy = 239.75 kJ/mol
chem. pot. = 130.97 kJ/mol

3.6 Optimized structures for methyl cation affinities

All structures on the RI-B3LYP-D3/def2-TZVPP level of theory

$[CH_3]^+$

```
$coord
  -0.00000000000000    0.00000000000000    0.00000000000000  c
  -2.06235047617564    0.00000000000000    0.00000000000000  h
   1.03117523808783   -1.78604790387505    0.00000000000000  h
   1.03117523808783    1.78604790387505    0.00000000000000  h
$end
```

Etot = -39.46715352066 H
ZPE = 81.16 kJ/mol
enthalpy = 91.14 kJ/mol
chem. pot. = 35.41 kJ/mol

MeCl

```
$coord
  -0.00000000000000    0.00000000000000    0.29006239050744  c
  -0.97329303629883   -1.68579298952253    0.93930266393883  h
  -0.97329303629883    1.68579298952253    0.93930266393883  h
   1.94658607259765    0.00000000000000    0.93930266393883  h
   0.00000000000000    0.00000000000000   -3.10797038232392  cl
$end
```

Etot = -500.0618405244 H
ZPE = 97.67 kJ/mol
enthalpy = 108.12 kJ/mol
chem. pot. = 38.11 kJ/mol

$[Me_2Cl]^+$

```
$coord
  -2.01009662813659   -0.02094899151435    1.86375766979853  c
  -2.29344353102282   -1.98477813268909    1.34843687309117  h
  -0.56144975887149    0.27199353001293    3.28425698425437  h
  -3.76842717334130    0.93571042311285    2.32059560985027  h
  -0.92302783894159    1.61101633773991   -1.01147226602583  cl
   2.03643101733984   -0.02475746657740   -1.83486094206800  c
   2.67497678341471    0.96114150920980   -3.51889446760592  h
   3.30574936895173    0.22909766985084   -0.24522570703507  h
   1.53928776060745   -1.97847487914549   -2.20659375425948  h
$end
```

Etot = -539.6407755530 H
ZPE = 197.3 kJ/mol
enthalpy = 213.53 kJ/mol
chem. pot. = 124.52 kJ/mol

MeBr

```
$coord
   0.00000000000000    0.00000000000000    0.36653570605589  c
  -0.97575396449039   -1.69005544218411    0.99180936958102  h
  -0.97575396449039    1.69005544218411    0.99180936958102  h
   1.95150792898076    0.00000000000000    0.99180936958102  h
   0.00000000000000    0.00000000000000   -3.34201093690278  br
$end
```

Etot = -2613.944610948 H
ZPE = 96.18 kJ/mol
enthalpy = 106.83 kJ/mol

chem. pot. = 33.36 kJ/mol

$[Me_2Br]^+$

```
$coord
   0.04789614574710    1.64928687896576    2.38264994513806  c
  -0.16780778353817    3.58823999332894    3.01936307167497  h
   1.95191308250680    0.93836850344589    2.64100308685095  h
  -1.41743681256646    0.41134390886083    3.10209012922331  h
  -0.49775657260575    1.87416190647732   -1.33819294991203  br
  -0.02526064512017   -1.73106046202290   -2.32429097590120  c
  -0.36567822413537   -1.72571328922035   -4.34802985293239  h
   1.90914821402826   -2.20763175714291   -1.84669183618641  h
  -1.43501740431623   -2.79699568269250   -1.28790061795526  h
$end
```

Etot = -2653.528959047 H
ZPE = 194.3 kJ/mol
enthalpy = 211.23 kJ/mol
chem. pot. = 117.52 kJ/mol

MeI

```
$coord
   0.00000000000000    0.00000000000000    0.44732670999904  c
  -0.97665414516943   -1.69161460085620    1.06401535131976  h
  -0.97665414516943    1.69161460085620    1.06401535131976  h
   1.95330829033885    0.00000000000000    1.06401535131976  h
   0.00000000000000    0.00000000000000   -3.63942489337836  i
$end
```

Etot = -337.6094070615 H
ZPE = 94.67 kJ/mol
enthalpy = 105.52 kJ/mol
chem. pot. = 29.67 kJ/mol

$[Me_2I]^+$

```
$coord
   0.32008448734126   -0.15711294507039   -2.61837542073267  i
  -3.05227046163348    0.24759220766944   -0.31961401722891  c
  -2.82540281804022    1.98215882640034    0.74665887349443  h
  -4.58652170958249    0.36183470037218   -1.67779765451929  h
  -3.16517118724529   -1.43342445205559    0.84582212115579  h
   3.03614162003359   -0.23616455548949    0.45446694876844  c
   4.83235114799044   -0.62644242293036   -0.45924496802060  h
   2.98768081884942    1.61663906672972    1.32748811228572  h
   2.45310810228676   -1.75508042562596    1.70059600479705  h
$end
```

Etot = -377.2045158694 H
ZPE = 191.5 kJ/mol
enthalpy = 208.93 kJ/mol
chem. pot. = 111.71 kJ/mol

1,2,3,4-tetrafluorobenzene

```
$coord
  -1.31272981463121    0.00000000000000   -2.20059788013987  c
   1.31272981463121    0.00000000000000   -2.20059788013987  c
   2.60574453109002    0.00000000000000    0.08665000556445  c
   1.31466146990386    0.00000000000000    2.35716523186852  c
  -1.31466146990386    0.00000000000000    2.35716523186852  c
  -2.60574453109002    0.00000000000000    0.08665000556445  c
```

2.37649039425456	0.00000000000000	4.10020580088497	h
-2.37649039425456	0.00000000000000	4.10020580088497	h
-5.13954910275675	0.00000000000000	0.04373279833752	f
-2.56553514203162	0.00000000000000	-4.38715595651556	f
2.56553514203162	0.00000000000000	-4.38715595651556	f
5.13954910275675	0.00000000000000	0.04373279833752	f

$end
Etot = -629.1706470358 H
ZPE = 176.4 kJ/mol
enthalpy = 199.21 kJ/mol
chem. pot. = 92.21 kJ/mol

1H-1,2,3,4-tetrafluorobenzeniumcation
$coord

2.28954803246724	1.58837198548894	0.07027723345826	c
2.33552724925786	-0.98256201221449	-0.19585650569223	c
-2.44310170485375	1.48724027473053	0.04185601736477	c
-0.09463207538054	2.78086042895021	0.18186551756249	c
-4.15484506462752	2.60413954966138	0.05657213744876	h
-0.11234365623503	5.21246336664826	0.37500571385452	f
4.35817275681073	2.95575182074224	0.10424678306789	f
4.43384542684879	-2.19840581087914	-0.44278961777940	f
-2.43329170178720	-1.05051352598421	-0.19548890655939	c
-4.15080796340851	-2.14331304409054	-0.39934607672897	h
-0.03769174127683	-2.51267599411748	-0.01438966315706	c
-0.02051795117207	-3.18348527113194	1.98362015968337	h
0.03013839335681	-4.55787176780380	-1.56557279252306	f

$end
Etot = -629.4395305796 H
ZPE = 206.6 kJ/mol
enthalpy = 230.30 kJ/mol
chem. pot. = 119.08 kJ/mol

2H-1,2,3,4-tetrafluorobenzeniumcation
$coord

2.27597312609111	1.63017557356880	0.06732489452344	c
2.37076467962668	-0.93931945244938	-0.20215642064741	c
-2.40813258101580	1.58098297772934	0.06738685554498	c
-0.08066341992401	2.84327792913736	0.19226445368458	c
-4.17251408335482	2.61003993587423	0.08787373400958	h
4.36491124716903	2.98654252031553	0.09989535320352	f
4.46624021882434	-2.16205971786128	-0.45746802933482	f
-2.38529224859325	-0.98093401571700	-0.19044765185555	c
0.00817167717605	-2.49160152592541	-0.01302052701596	c
0.00751815227183	-3.13680635188319	1.99252469239336	h
0.03376583255867	-4.53112729327146	-1.55155865286025	f
-4.42851733894252	-2.29571679349579	-0.44841182711886	f
-0.05222526188733	4.88654621397822	0.35579312547338	h

$end
Etot = -629.4371169585 H
ZPE = 206.3 kJ/mol
enthalpy = 230.07 kJ/mol
chem. pot. = 118.62 kJ/mol

5H-1,2,3,4-tetrafluorobenzeniumcation

```
$coord
   2.38264471878560     1.44201628903182     0.00000000000000  c
   2.50781755048801    -1.21073717469164     0.00000000000000  c
   0.27630611864908    -2.53282057813071     0.00000000000000  c
  -2.16607886043588     1.51991561132040     0.00000000000000  c
   0.04416291783354     2.79140478407268     0.00000000000000  c
  -3.94122826806161     2.53420860863057     0.00000000000000  h
   0.18669662406973     5.27552264704331     0.00000000000000  f
   4.46409222384391     2.70695018776659     0.00000000000000  f
   4.71735634489212    -2.33595734644438     0.00000000000000  f
   0.30232451157052    -4.98534990617028     0.00000000000000  f
  -2.19470326104532    -1.26864718338989     0.00000000000000  c
  -3.28969531029482    -1.96825296951928     1.62870661787406  h
  -3.28969531029482    -1.96825296951928    -1.62870661787406  h
$end
```

Etot = -629.4543062214 H
ZPE = 205.2 kJ/mol
enthalpy = 229.05 kJ/mol
chem. pot. = 117.93 kJ/mol

5-methyl-1,2,3,4-tetrafluorobenzene

```
$coord
   3.35058739770098     0.74407009338070     0.18243558207677  c
   3.41666619170583    -1.87125379203802     0.00532711538608  c
  -1.20999894103874     0.74799867076507     0.00338259394753  c
   1.08061233509760     2.03922564872730     0.18228446745415  c
   5.54351612815493     2.00653544697407     0.35390423090093  f
   5.60713696307055    -3.12223484060062     0.00424460984706  f
  -1.11822637180169    -1.87030587615503    -0.17285586103845  c
  -3.27602348578673    -3.20125298546250    -0.35013022097460  f
   1.12119557449289     4.07665362457751     0.32301321352800  h
   1.15124661944244    -3.18580611872586    -0.17417702807208  c
   1.17254881441186    -5.70149935157396    -0.34746122582609  f
  -3.71780547000306     2.08279562014658    -0.00148322407970  c
  -4.78454024997159     1.64741673021913    -1.71140585196263  h
  -4.87569040445418     1.48704864349886     1.59770093675288  h
  -3.46122510102110     4.12060848626673     0.10522066206007  h
$end
```

Etot = -668.4778011746 H
ZPE = 247.9 kJ/mol
enthalpy = 275.60 kJ/mol
chem. pot. = 155.92 kJ/mol

1H-5-methyl-1,2,3,4-tetrafluorobenzeniumcation

```
$coord
   3.18169615764827    -2.08041967827029     0.19090613686944  c
  -1.44722708949259     0.79901755652058    -0.01812426608845  c
   5.29495726444279    -3.27588550595237     0.43530661724152  f
  -1.28269763011560    -1.88156844770036    -0.32195205352314  c
  -3.35719717375961    -3.13054404103448    -0.66764656000076  f
   0.95953194211022    -3.32448534207094    -0.22754543052183  c
   0.83887429636394    -5.79644834254363    -0.43006603879580  f
  -3.99917414253087     2.03195089296900    -0.05458504090041  c
  -4.92535943626592     1.72782409537720    -1.87136553702425  h
  -5.21869746734283     1.22823370130927     1.39976690298791  h
  -3.82677538254876     4.05352616636209     0.26640075587935  h
```

0.76892154433582 2.02663696627917 0.35655397472651 c
0.83962100229196 4.04589931283356 0.68054232721785 h
3.26115569556756 0.73971506241141 0.18081941110238 c
3.91949421306141 1.18714909324202 -1.76992230219355 h
4.99287620623426 1.64939851026774 1.85091110302324 f
$end
Etot = -668.7540890076 H
ZPE = 278.3 kJ/mol
enthalpy = 306.49 kJ/mol
chem. pot. = 185.46 kJ/mol

2H-5-methyl-1,2,3,4-tetrafluorobenzeniumcation
$coord
-1.41278641060312 0.89524651235487 -0.04092909708742 c
-1.40913678810314 -1.78958273798531 -0.16469564320506 c
-3.60328238434850 -2.98003715960197 -0.32916808624383 f
0.78155116810929 -3.11831588871987 -0.03846102693679 c
0.83250398033818 -5.56812867722195 -0.05533211405525 f
-3.87382747903922 2.20767393702667 -0.14333839042891 c
-4.92357732324969 1.60694761783592 -1.82261843032254 h
-5.04232674273625 1.59489777800368 1.45497107417561 h
-3.68160604015909 4.24934613666587 -0.11928785124292 h
0.87538954599087 2.25668781878441 0.22150045923754 c
0.85276763080531 4.29295220680856 0.38363185989131 h
3.09385779920248 0.98531837566800 0.35557397650095 c
3.29791802768418 -1.81681063693741 -0.03729340042011 c
4.03795304363431 -2.03599086892827 -1.98538989358007 h
5.23309214463412 2.12920727106490 0.71450655881754 f
4.94150982784031 -2.90941168481812 1.60633000490005 f
$end
Etot = -668.7608188934 H
ZPE = 277.1 kJ/mol
enthalpy = 305.65 kJ/mol
chem. pot. = 183.10 kJ/mol

3H-5-methyl-1,2,3,4-tetrafluorobenzeniumcation
$coord
-1.36979795652403 0.94976984974266 -0.02719842167814 c
-1.28406077562810 -1.63890220553507 -0.02656246315319 c
-3.33051204941490 -2.99102610324865 -0.02014955952147 f
-3.83006998454898 2.36738920612451 -0.03198020336528 c
-5.13576650999758 1.56490467659591 -1.40584625341701 h
-4.72755069206550 2.25020532421334 1.82131996772439 h
-3.53326778488288 4.35068332049728 -0.48179358820868 h
0.99205560255066 2.16871628193956 0.09187323435897 c
1.04245722424702 4.21896179176127 0.11937416406782 h
3.34981519594623 0.95271050736212 0.22722740385226 c
5.42175400448059 2.32616689477906 0.43434353422886 f
1.11516924406496 -3.13061204224995 -0.22708192372710 c
1.22224185855303 -3.58288977738540 -2.28063792984482 h
3.46133287303247 -1.62370910479903 0.23785972768345 c
5.55197740445804 -2.86860636211593 0.46264780432468 f
1.05422234572886 -5.31376225768166 1.10660450667523 f
$end
Etot = -668.7507228122 H
ZPE = 277.2 kJ/mol
enthalpy = 306.25 kJ/mol

chem. pot. = 178.06 kJ/mol

4H-5-methyl-1,2,3,4-tetrafluorobenzeniumcation
$coord
-1.13246924617471 1.00346429605151 -0.00997829565143 c
-3.61463560947854 2.26153035407453 0.21365048364594 c
-4.87755486047265 1.68780536417023 -1.31802736968483 h
-4.53457983544176 1.62353772646483 1.95595696708679 h
-3.44057075967820 4.30710965857036 0.23999810308851 h
1.12932349072291 2.23803934340017 -0.01254332260928 c
1.25953345757134 4.27634110772177 0.07060221744955 h
3.39630962626681 0.85241914781824 0.02457337064612 c
5.51659458086219 2.07185580114981 0.07472359532450 f
3.54916447476477 -1.82357151712941 0.07947136053997 c
5.76956594868194 -2.92600618649117 0.22892521767178 f
1.34032186048807 -3.13569800200061 0.06197756188439 c
-1.13209022456488 -1.81743676234732 -0.34567673780576 c
-1.48064857293843 -2.06284946582687 -2.40280473264658 h
1.28496123208143 -5.57823567784794 0.19488218053730 f
-3.03322556269028 -2.97830518777820 0.94426940052305 f
$end
Etot = -668.7612731895 H
ZPE = 277.9 kJ/mol
enthalpy = 306.05 kJ/mol
chem. pot. = 185.05 kJ/mol

5H-5-methyl-1,2,3,4-tetrafluorobenzeniumcation
$coord
3.40221194274794 0.50846110402208 0.23371640352468 c
3.43334048260710 -2.05848784952632 0.00567808167716 c
-1.37308706372256 0.54525812510658 -0.04673549962115 c
5.50125033042261 1.77202460215166 0.47010813543001 f
5.52949396215284 -3.38989355450914 0.01339158318048 f
-1.25125461927575 -2.01815225028925 -0.27304974728488 c
-3.31252035695227 -3.41334656721799 -0.51360428657549 f
1.08865743796887 -3.30628737174379 -0.25172628066081 c
1.09607070240893 -5.73829293035781 -0.47327875206623 f
-3.82469474783150 1.91303155320799 -0.02316963864856 c
-5.09178046544203 1.17273348514306 -1.47096055463536 h
-4.77074352700023 1.59987954070662 1.79170116351575 h
-3.57799772594255 3.93685852908298 -0.28177486145778 h
1.03257045378337 1.96666463216217 0.21064263021493 c
1.15581761683771 3.37185533333727 -1.31941818172735 h
0.96266557723749 3.13769361872398 1.92847980513467 h
$end
Etot = -668.7731691228 H
ZPE = 276.0 kJ/mol
enthalpy = 304.99 kJ/mol
chem. pot. = 180.39 kJ/mol

6H-5-methyl-1,2,3,4-tetrafluorobenzeniumcation
$coord
1.10829506586842 2.18927030671206 -0.27239277296787 c
1.12776983628626 4.21967710380016 -0.02867565475379 h
3.30370985489693 0.89505226150447 -0.34702522231819 c
5.53636343709574 1.98230591329529 -0.14096290119065 f
3.28112013930946 -1.78166527220403 -0.66383982220396 c

5.40340900576529	-2.97959661064829	-0.72480987927126	f
1.02978575605679	-3.16083211555398	-0.92119783381073	c
1.12392423074949	-5.62378278051807	-1.21950619842904	f
-1.21051502585259	-1.85590351349198	-0.85567741350653	c
-3.32658509940159	-3.08005830851461	-1.11197443692594	f
-1.35807683737756	0.89524185779637	-0.47448823816930	c
-2.33342009899763	1.66141970195421	-2.15113838729817	h
-3.11466781424123	1.54084277325305	1.83421807228870	c
-3.35814931609141	3.57837072033953	1.93311521237736	h
-4.94674116015864	0.65287909137419	1.56339456996970	h
-2.26622197390777	0.86677887090167	3.58096090620974	h

$end
Etot = -668.7623293583 H
ZPE = 279.6 kJ/mol
enthalpy = 307.34 kJ/mol
chem. pot. = 187.28 kJ/mol

dimethyl-1,2,3,4-tetrafluorobenzene
$coord

0.27740142023480	-3.14217322830333	0.17408489755863	c
2.90292307695971	-3.18382843152168	0.14514692769478	c
4.21083463897980	-0.92280132983951	0.00863747808816	c
2.86449293697783	1.32835489777717	-0.10109320195857	c
0.24372160691414	1.39251027675546	-0.08075175963194	c
-1.08635867459652	-0.90530128780691	0.06371685547230	c
4.20620884383498	3.48619967515528	-0.23011523096220	f
6.73469980725887	-0.91589874201998	-0.01660342586380	f
4.15692982890829	-5.37213430947921	0.25084848058061	f
-0.92498179145194	-5.38051663334003	0.31617959083946	f
-3.92827825609968	-0.95357769428862	0.09811306123771	c
-4.69921174904081	-0.12640251146202	-1.62807653022297	h
-4.64248696339120	-2.87126367189643	0.26349903298996	h
-4.66447925292437	0.14796249047458	1.67936393936116	h
-1.13304807735939	3.87564571199614	-0.21114321003346	c
-2.39876827935573	3.92980402733888	-1.83928299006989	h
-2.29493484461902	4.16326557131975	1.46966154678390	h
0.17533572877020	5.45015518914066	-0.36218546186392	h

$end
Etot = -707.7836024446 H
ZPE = 320.6 kJ/mol
enthalpy = 352.36 kJ/mol
chem. pot. = 224.32 kJ/mol

1H-dimethyl-1,2,3,4-tetrafluorobenzeniumcation
$coord

0.14425217598261	-3.16731522501599	-0.05105847966836	c
2.81976865533535	-3.31079359061527	0.12169849661818	c
4.13488315684947	-1.11487348723648	0.23071721941770	c
-0.01883700574231	1.32130598233482	0.00960567163367	c
-1.31218731446061	-0.92896326456087	-0.08759533971445	c
6.57256521672009	-1.05676812704474	0.47820523606906	f
3.90917760215849	-5.54204448541580	0.24963977746939	f
-1.00981866701209	-5.33221589162122	-0.12935312344202	f
-4.15055525775077	-1.07077915784837	-0.13081466978081	c
-4.87634445002117	-0.34092720459369	-1.91821891487583	h
-4.82594910726754	-2.99516638876366	0.09293180438419	h
-4.94902470359401	0.07464292861348	1.38305083830382	h

-1.29763601421178	3.81025149405185	0.14970657093347	c
-2.84980699343053	3.92441291174452	-1.20425844057621	h
-2.14792008354563	4.02209749615129	2.02804641465537	h
-0.00139173862596	5.37639248874488	-0.13010168658655	h
2.81203294425212	1.34498301729043	-0.16160449407037	c
3.16335612118261	1.77452852965425	-2.18920801258727	h
3.88343546318179	3.21123197413059	1.25861113181700	f

$end
Etot = -708.0717749888 H
ZPE = 349.5 kJ/mol
enthalpy = 382.47 kJ/mol
chem. pot. = 249.67 kJ/mol

2H-dimethyl-1,2,3,4-tetrafluorobenzeniumcation
$coord

0.03086263516935	-3.19060577008159	0.17546598951349	c
2.59271643293982	-3.25922813860127	0.26608431861734	c
-0.01269848870285	1.51773963589846	-0.03802535599160	c
-1.29803606374549	-0.85861036172993	0.02946017845558	c
3.84549354383210	-5.35377019477123	0.49832550071474	f
-1.26563262278267	-5.32960586306473	0.29948345415915	f
-4.09262587652020	-0.92603498459415	-0.03597245051583	c
-4.74089972852782	-0.14774027928665	-1.84413202672059	h
-4.85720398714628	-2.81235338920551	0.19400997094989	h
-4.86603739843612	0.32970627907240	1.41101096256025	h
-1.43599085667478	3.97282736683171	-0.12240319848289	c
-2.71925137337338	4.01704226900653	-1.73375867020414	h
-2.56321216614757	4.22485715458074	1.58533356383450	h
-0.15143055067390	5.56564217399961	-0.27832660487641	h
4.11604691315191	-0.90755504999241	-0.06477572823354	c
4.83989161391314	-0.98806206630911	-2.02929142924888	h
2.55693974882541	1.45435176375998	0.04457691176055	c
3.90608223212290	3.51712816207708	0.09128940363346	f
6.11498599277648	-0.82572870758997	1.55164521007485	f

$end
Etot = -708.0722515383 H
ZPE = 349.8 kJ/mol
enthalpy = 382.52 kJ/mol
chem. pot. = 250.70 kJ/mol

6H-dimethyl-1,2,3,4-tetrafluorobenzeniumcation
$coord

0.09494204729406	-3.05152021260256	-0.62876333868481	c
2.76474975214770	-3.02293003958434	-0.62876203897488	c
-1.27267874686352	-0.87009068611921	-0.55584465630414	c
3.97031417398312	-5.14649496427633	-0.71781073456344	f
-1.00383075706076	-5.29965445905674	-0.73141819970521	f
-4.08169794986484	-0.91079023885134	-0.58687199541050	c
-4.78937887650203	-2.56936643984168	-1.57566578142656	h
-4.79477633150975	-1.01579749587330	1.35470041580530	h
-4.85414440050459	0.79358724149303	-1.44194379620102	h
2.87586427018075	1.45406730177088	-0.48283939705289	c
4.10003080464720	3.59475416597855	-0.45438140278594	f
4.17928604848997	-0.76788678820652	-0.55972671464690	c
0.09163594215240	1.58780711604228	-0.37367799833495	c
-0.50501734020240	2.75890430590229	-1.98485904186374	h
-0.71640353671406	3.07270510500977	2.07066600439904	c

-0.23076948882178	1.98884548616975	3.74943204911126	h
0.25175985454184	4.88315329772624	2.11548565869723	h
-2.74254138740422	3.39856015660207	2.02839607462178	h
6.66265592201092	-0.87785285228276	-0.59611510667972	f

$end
Etot = -708.0802768351 H
ZPE = 351.5 kJ/mol
enthalpy = 383.70 kJ/mol
chem. pot. = 253.91 kJ/mol

1,2,3-trifluorobenzene
$coord

2.28472225443595	0.00000000000000	-1.40994405688642	c
2.25961459270098	0.00000000000000	1.20522230901210	c
0.00000000000000	0.00000000000000	2.54262230555036	c
-2.25961459270098	0.00000000000000	1.20522230901210	c
-2.28472225443595	0.00000000000000	-1.40994405688642	c
0.00000000000000	0.00000000000000	-2.70806066465918	c
4.08112044180603	0.00000000000000	-2.38008632236133	h
-4.08112044180603	0.00000000000000	-2.38008632236133	h
0.00000000000000	0.00000000000000	-4.75024652248982	h
-4.43242061134481	0.00000000000000	2.50934077110240	f
0.00000000000000	0.00000000000000	5.06661947986518	f
4.43242061134481	0.00000000000000	2.50934077110240	f

$end
Etot = -529.9292766527 H
ZPE = 197.5 kJ/mol
enthalpy = 218.01 kJ/mol
chem. pot. = 116.63 kJ/mol

1H-1,2,3-trifluorobenzeniumcation
$coord

0.22266535616007	-2.42273897696534	-1.45696109486181	c
0.09819008130458	-2.51099928173640	1.17047527225719	c
-0.25222487676855	-0.29572488610098	2.50175655935946	c
0.42126325501374	-4.21075003665054	-2.43958190789167	h
-0.55224796074696	-0.27396041926541	4.91602663502436	f
0.16242461822300	-4.69089698878771	2.37020284734881	f
-0.24998373719149	2.08956542682994	-1.62135812710849	c
-0.45585824276244	3.88220860882776	-2.58505688825369	h
0.04713649551605	-0.14378645312949	-2.84931773653920	c
0.08285446664058	-0.22526274415877	-4.88976796908865	h
-0.07665468858587	2.17583513034605	1.17030018319479	c
1.99018855246717	2.60256716058950	1.41821232104389	h
-1.43775331926991	4.02394346020142	2.29506990551499	f

$end
Etot = -530.1933016924 H
ZPE = 226.0 kJ/mol
enthalpy = 247.46 kJ/mol
chem. pot. = 142.01 kJ/mol

2H-1,2,3-trifluorobenzeniumcation
$coord

0.19838456041537	-2.47359257525920	-1.43079851210179	c
0.07251212261846	-2.59131060072974	1.22121586067771	c
-0.19056206435267	-0.38465892846682	2.51408631008402	c
0.35527695545211	-4.24132512121867	-2.45338914400575	h

```
   -0.46041828330717    -0.30390298384379     4.94381685107549  f
   -0.19012918417571     1.98762329270351    -1.58694692762633  c
    0.07037572498350    -0.23345280963959    -2.85519260756655  c
    0.08368180201031    -0.26606950021835    -4.89754886675396  h
   -0.00767815282770     2.14139147488558     1.23877997508471  c
    1.99362106998965     2.70505870844049     1.56509763728074  h
   -1.55564270439919     3.90344518224242     2.25786281801112  f
   -0.45618372865266     4.13469261123421    -2.72829187706514  f
    0.08676188224567    -4.37789875012997     2.21130848290575  h
$end
```
Etot = -530.2061392753 H
ZPE = 227.8 kJ/mol
enthalpy = 249.30 kJ/mol
chem. pot. = 143.44 kJ/mol

4H-1,2,3-trifluorobenzeniumcation

```
$coord
   -0.00043814777272    -2.52281980901371    -1.33149991987799  c
    0.00009203601749    -2.44212373343399     1.34170367921979  c
    0.00039065381844    -0.18913120913455     2.76102489470825  c
   -0.00038695599129     2.04853298737781     1.46099170300268  c
   -0.00013869884256    -0.31620080551303    -2.62277420011230  c
   -0.00032126236164    -4.35258232518631    -2.24111383773514  h
   -0.00052773749609    -0.29900954699381    -4.66731681420613  h
   -0.00201038744158     4.18231986953471     2.67681075177607  f
    0.00100816957479    -0.28324612652270     5.24177039369346  f
    0.00027018401297    -4.54886614600568     2.58613214093896  f
    0.00028949599461     2.14867769814723    -1.31599586890376  c
    1.62809076139351     3.28665198902624    -1.94439363392547  h
   -1.62631811090598     3.28779715771781    -1.94533928857839  h
$end
```
Etot = -530.2218454026 H
ZPE = 226.4 kJ/mol
enthalpy = 248.11 kJ/mol
chem. pot. = 142.44 kJ/mol

5H-1,2,3-trifluorobenzeniumcation

```
$coord
    0.00000000000000    -2.37666351569655    -1.09432266552255  c
    0.00000000000000    -2.34414515206907     1.46914929147357  c
    0.00000000000000     0.00000000000000     2.76822291453691  c
    0.00000000000000     2.34414515206907     1.46914929147357  c
    0.00000000000000    -4.16248204404300    -2.09239404238054  h
    0.00000000000000     4.41542865783279     2.84759162333735  f
    0.00000000000000     0.00000000000000     5.20305339954338  f
    0.00000000000000    -4.41542865783279     2.84759162333735  f
    0.00000000000000     2.37666351569655    -1.09432266552255  c
    0.00000000000000     4.16248204404300    -2.09239404238054  h
    0.00000000000000     0.00000000000000    -2.52365269714496  c
    1.60967242947668     0.00000000000000    -3.85383601537549  h
   -1.60967242947668     0.00000000000000    -3.85383601537549  h
$end
```
Etot = -530.2132744326 H
ZPE = 224.7 kJ/mol
enthalpy = 246.41 kJ/mol
chem. pot. = 142.59 kJ/mol

4-methyl-1,2,3-trifluorobenzene

```
$coord
  -2.01335628206396    -1.18760357124420    -0.01360847000023  c
   0.61985411678631    -1.26701892494540    -0.00366021120754  c
   1.88602662059909     1.03624816008690     0.00727909794043  c
   0.61792849605238     3.33149732589792     0.00659991349626  c
  -2.00685021707846     3.33228560326554    -0.00087758802340  c
  -3.33555250077998     1.08458810122298    -0.01112931517650  c
  -3.05452682795949    -2.94671999934391    -0.02367839500696  h
  -5.37663244912480     1.14045188062362    -0.01824411520884  h
   1.90572621808045     5.50415737712283     0.01344370841306  f
   4.42685971778268     1.07554362796209     0.01809843400421  f
  -3.22311339411335     5.55824528251535     0.00100830659610  f
   2.09492762314938    -3.69514200809958     0.00321718079000  c
   3.39878663055776    -3.78093930137002    -1.59179212870214  h
   3.22988002229210    -3.87143345661254     1.71678429459376  h
   0.83004222581986    -5.31416009708163    -0.10344071250818  h
$end
```

Etot = -569.2361291878 H
ZPE = 269.1 kJ/mol
enthalpy = 294.37 kJ/mol
chem. pot. = 180.50 kJ/mol

1H-4-methyl-1,2,3-trifluorobenzeniumcation

```
$coord
  -1.95156826965264    -1.44396596456250     0.04234223880826  c
   0.74999982462455    -1.47563480279099    -0.05487158847625  c
   2.11278943907748     0.81289087534721     0.02878203122577  c
   0.88314905257112     3.07654687347733     0.22319641089671  c
  -3.24641485068361     0.74916427067070     0.20351418548698  c
  -2.93407472139850    -3.23532385113938     0.05482194452632  h
  -5.28443701984704     0.82090780866911     0.36415471931567  h
   2.09694118757534     5.18623769010873     0.44325108848654  f
   4.61319502823585     0.74489963033193     0.04011942857856  f
   2.17884132470536    -3.87088438017461    -0.17222015246251  c
   3.49837481586708    -3.83302915905952    -1.76590914362163  h
   3.38228045058885    -4.03694805801661     1.50685806063183  h
   0.95401099360181    -5.51074757208893    -0.30751497322197  h
  -1.92289994304512     3.21311512684241    -0.01357427141329  c
  -2.19834685750555     3.73009170472747    -2.03936342893071  h
  -2.93184045471483     5.07267980765755     1.44641345016979  f
$end
```

Etot = -569.5163864054 H
ZPE = 297.0 kJ/mol
enthalpy = 323.32 kJ/mol
chem. pot. = 205.80 kJ/mol

2H-4-methyl-1,2,3-trifluorobenzeniumcation

```
$coord
  -1.98656885935798    -1.34703945937437    -0.14674015768685  c
   0.67443264312254    -1.50846607048431    -0.01448350762895  c
   1.91100254749918     0.75006922439667     0.20623543350467  c
  -3.43070126149193     0.88379250086843    -0.05236061018147  c
  -3.01573411138013    -3.11432029439114    -0.28018049616142  h
  -5.47231704663556     0.82575023453517    -0.06985308311567  h
   4.35229041545776     0.87397800195301     0.45266256906475  f
   2.05101849090569    -3.99187720814611    -0.03635726933568  c
```

2.60293859536673 -4.48484589271577 -1.96222342659064 h
3.76088435085388 -3.90355650380853 1.10213942987500 h
0.85290213024541 -5.49638916272911 0.69196105777936 h
0.62894122957874 3.26707959871299 -0.00322700016468 c
0.95321002545809 3.81350260189855 -2.00912219408911 h
-2.18920949767987 3.11806171242872 0.18031922470501 c
1.65199794203713 5.04939087182588 1.52451859999996 f
-3.34508759397970 5.26486984502991 0.41671143002575 f
$end
Etot = -569.5190609535 H
ZPE = 298.8 kJ/mol
enthalpy = 325.50 kJ/mol
chem. pot. = 204.58 kJ/mol

3H-4-methyl-1,2,3-trifluorobenzeniumcation
$coord
-2.13416793253276 -1.30758662905101 -0.25513844114038 c
0.44607760725858 -1.44213777080475 -0.01339929957274 c
0.43122108820262 3.36538136447756 0.29258363835897 c
-2.14344765609847 3.34787159503286 0.04556791725509 c
-3.38724952666864 1.02726382585373 -0.22806145630622 c
-3.24311249813597 -3.01998793837946 -0.35950655909431 h
-5.43104773429964 1.08829442825016 -0.36821607884501 h
1.70509575872572 5.41845401524898 0.66055259380635 f
-3.45414515232263 5.46584360931462 0.17256107685722 f
1.90499724386726 -3.80701218725719 0.22908868020849 c
3.39002044373963 -3.91538876048697 -1.20503953011605 h
2.89821534298343 -3.80780511851349 2.04636339434463 h
0.70146460533098 -5.46535318497943 0.10001054905251 h
1.88972698333520 0.98619668347418 -0.11771721437672 c
2.32480317077380 1.11266229189574 -2.18159807479307 h
4.10154825584084 0.95330377592436 1.18194880436128 f
$end
Etot = -569.5149232775 H
ZPE = 297.4 kJ/mol
enthalpy = 323.43 kJ/mol
chem. pot. = 207.91 kJ/mol

4H-4-methyl-1,2,3-trifluorobenzeniumcation
$coord
-2.00979853128861 -1.35707239188885 -0.46661812185526 c
0.61287227628407 3.31470491483906 -0.71470238502453 c
-2.03956255609992 3.13272696109776 -0.64614232409934 c
-3.36027707106849 0.81400366497720 -0.52772684744973 c
-2.96013367516344 -3.16607114287768 -0.37597691003243 h
-5.40263572570357 0.86515526439602 -0.49472136020391 h
1.70451321808524 5.54461428160308 -0.83485010336637 f
-3.31385510436205 5.22336023737629 -0.70566221270167 f
0.78314459283327 -1.40209539767651 -0.48452052426728 c
1.37715823952270 -2.45097650015413 -2.18675362661571 h
1.86424181687776 -2.93390849932441 1.81446097211136 c
1.11403901559013 -4.84590894548771 1.75661649301683 h
3.91225534776716 -3.01037807030535 1.68004822231944 h
1.32093401614048 -2.03989930967755 3.58409632197558 h
1.96949346919183 1.11196629428176 -0.65837460979552 c
4.42761067139349 1.19977863882111 -0.73917298401145 f
$end

Etot = -569.5294647194 H
ZPE = 300.9 kJ/mol
enthalpy = 326.44 kJ/mol
chem. pot. = 211.74 kJ/mol

5H-4-methyl-1,2,3-trifluorobenzeniumcation
$coord

0.85733722445299	-1.17235233644777	-0.00566140016058	c
2.09925534182395	1.10240390325532	-0.00157017145675	c
0.76131424078031	3.39226818815241	0.00139832820533	c
-1.92871601194624	3.48091904074039	0.00394472325829	c
-3.25908909215082	1.30466364147476	0.00009625655249	c
-5.30343277741224	1.34662197054922	-0.00018863907784	h
2.00321603982398	5.49550103672102	0.00183147866161	f
4.59663722196810	1.21787666427099	-0.00051728892785	f
-2.99041107762028	5.73517489731005	0.00745725700882	f
2.26851718731290	-3.59479633547781	0.00193035249870	c
3.62303277762466	-3.64178265751470	-1.55706092129826	h
3.39431657463844	-3.74609935197750	1.73074682937757	h
1.01646484691285	-5.21812808348863	-0.12667192374987	h
-1.93466357544764	-1.14257729075924	-0.01043793191546	c
-2.60733745230616	-2.29356445746128	1.59164967639854	h
-2.59644146845491	-2.26612882934722	-1.63694662537466	h

$end
Etot = -569.5329284615 H
ZPE = 295.8 kJ/mol
enthalpy = 322.40 kJ/mol
chem. pot. = 204.80 kJ/mol

6H-4-methyl-1,2,3-trifluorobenzeniumcation
$coord

0.83173488808393	-1.41774226140950	-0.00109879620423	c
2.08713135050095	0.96288120170786	0.01636865173438	c
0.88378372150097	3.33719227096095	0.01583219299782	c
-1.69969869062214	3.38119029772454	-0.00002333217601	c
2.24930499410107	5.41410355860795	0.03065639728254	f
4.54077849050920	0.98355119608663	0.03387433929505	f
-2.90957077773454	5.52160827843202	-0.00082217076572	f
2.37856831274389	-3.79337641975292	0.00211429445018	c
3.63809278657651	-3.84703423903663	-1.62846504938123	h
3.55728061079329	-3.89252218752288	1.69043822315421	h
1.16164010241904	-5.44781011681281	-0.04963571966960	h
-1.73568265816591	-1.33677573303859	-0.01729442441869	c
-2.81511893629595	-3.07413497815460	-0.03106089798187	h
-3.19002498074348	1.04383539043650	-0.01642851009669	c
-4.48152432334974	1.08864622125419	-1.64990833543861	h
-4.49669489031704	1.07638752051725	1.60545313721837	h

$end
Etot = -569.5354845601 H
ZPE = 298.2 kJ/mol
enthalpy = 324.35 kJ/mol
chem. pot. = 208.86 kJ/mol

5-methyl-1,2,3-trifluorobenzene

$coord

-0.70971566412244	-1.23463390814548	-0.03462727215667	c
1.92608978516312	-1.20760162471483	-0.02163169235912	c
3.21489376414347	1.06712856984017	0.00505754679092	c
1.93950629866613	3.35874718994361	0.01784222210133	c
-0.68381372006162	3.31504060654438	-0.00091772603210	c
-2.00687976709826	1.06142630978260	-0.02788864801130	c
3.01029431920150	-2.93955125732386	-0.03578410792292	h
-4.04907736873064	1.13200098013915	-0.04669661059502	h
-1.92884965508458	5.52409824898153	0.00010029979914	f
3.20108626179766	5.54737203310469	0.03473053577016	f
5.75047046608123	1.09434630921693	0.01111552126068	f
-2.13535027343562	-3.69783499353271	0.00662166785510	c
-1.02772272703004	-5.22123685814902	-0.82569402127305	h
-2.58993151134790	-4.24554404062576	1.94531843969456	h
-3.91100020814198	-3.55375756506133	-1.02754615492183	h

$end

Etot = -569.2352927214 H

ZPE = 268.5 kJ/mol

enthalpy = 294.02 kJ/mol

chem. pot. = 178.20 kJ/mol

1H-5-methyl-1,2,3-trifluorobenzeniumcation

$coord

-0.77412266777001	-1.44690040936363	-0.00670088692682	c
1.91667146164112	-1.35636730603626	-0.02431236645768	c
3.32952300303910	0.85752211269017	0.09711499782197	c
2.10029468490689	3.14678711905803	0.27794042019569	c
2.97832920153478	-3.11007986840345	-0.09520842410395	h
3.29830983627475	5.24786801717059	0.56371649004038	f
5.81893063001491	0.76331506450344	0.17935017608244	f
-2.07966214317236	-3.96590066571090	-0.04361856697143	c
-1.66606064251915	-4.96397687137311	-1.80070682227022	h
-1.41963750177835	-5.14704432901226	1.51200503260651	h
-4.11771470216343	-3.75654702659231	0.11522309041518	h
-2.03777273446400	0.79555452356511	0.11450893462441	c
-4.07939223105373	0.90946593972961	0.18806813930066	h
-0.68248614388361	3.23562404648186	-0.07375907272533	c
-0.79540944151220	3.60247781178481	-2.16187117239609	h
-1.78980060909476	5.18820184150823	1.15825003076425	f

$end

Etot = -569.5061284416 H

ZPE = 297.1 kJ/mol

enthalpy = 323.24 kJ/mol

chem. pot. = 207.25 kJ/mol

2H-5-methyl-1,2,3-trifluorobenzeniumcation

$coord

-0.83028622776626	-1.45401095823090	-0.09803695716927	c
1.85128449258006	-1.49247133914480	-0.00894464295134	c
3.13232444385328	0.71379599489101	0.14952246839424	c
-0.94949702266978	3.06218723885675	0.14809441316375	c
-2.21179525657922	0.84631159555035	-0.00930170901253	c
2.86970336185962	-3.26362000878813	-0.00146300298845	h
-4.25503364278736	0.83536826585983	-0.00202127669977	h
-2.10897689940158	5.21883845084016	0.34069858081225	f

```
   5.57872184706355    0.79482434749485    0.34527105565945  f
  -2.23135539154356   -3.87413056134539   -0.17037939446532  c
  -1.10930513940065   -5.41154089768226   -0.94707318960952  h
  -2.70136875706323   -4.38402173955260    1.78928569922589  h
  -4.01913498689043   -3.70148738426380   -1.17603940424952  h
   1.87498427420869    3.24968858446839   -0.03267868001003  c
   2.26472471156558    3.92496267663384   -1.97570982567380  h
   2.84501019297124    4.93530573441278    1.64877586557399  f
$end
Etot = -569.5275946370 H
ZPE = 298.0 kJ/mol
enthalpy = 324.65 kJ/mol
chem. pot. = 203.00 kJ/mol
```

4H-5-methyl-1,2,3-trifluorobenzeniumcation

```
$coord
  -0.49039296018711   -1.40555609921739   -0.02734938710642  c
   2.08674773007485   -1.33181136623609   -0.10202157110937  c
   3.34446451537212    1.00595436415672   -0.08011881279513  c
   2.11768151205836    3.37700008781796    0.01052445475330  c
  -0.45524084516227    3.36718978977491    0.07695694155766  c
   3.22961438530948   -3.02461051720774   -0.17229744577436  h
  -1.71549907068060    5.48159278349879    0.15429145541813  f
   3.45940225402514    5.46997502152775    0.02395342257053  f
   5.79557739744922    1.02856681710320   -0.14585746180820  f
  -1.93924489162511   -3.81038141818488   -0.00403359213051  c
  -0.72203845172534   -5.44433266520470   -0.26367922615604  h
  -2.93260672358642   -4.01260949836004    1.79932949391126  h
  -3.39111631036742   -3.80828444318577   -1.47221849363562  h
  -1.93672988910751    1.00780204114766    0.05539355211522  c
  -3.24780618281476    1.08506515108181   -1.55649218745381  h
  -3.20281246903268    1.01443995148779    1.70361885764337  h
$end
Etot = -569.5397864749 H
ZPE = 297.6 kJ/mol
enthalpy = 324.13 kJ/mol
chem. pot. = 206.71 kJ/mol
```

5H-5-methyl-1,2,3-trifluorobenzeniumcation

```
$coord
   1.99859406859623   -1.27880295690221   -0.49230612049071  c
   3.23898541141483    0.96073991510480   -0.61440660443224  c
  -0.83066869537727    3.26043286544909   -0.68550575412812  c
  -2.11236244936346    1.04416762325916   -0.56483679318528  c
   3.06837232322016   -3.02165882947565   -0.42019294413837  h
  -4.15848087471557    1.06192933547352   -0.54820659335744  h
  -1.96514240028692    5.47659762608177   -0.78481190816253  f
   5.72450077371802    1.13140743943567   -0.65021795503441  f
  -0.77733513096296   -1.38934780037659   -0.43453962644442  c
  -1.34941852885717   -2.45563135731026   -2.14258867873739  h
  -1.75892207244900   -3.05541351725033    1.82245047078265  c
  -3.79780215350866   -3.26742647237426    1.67887960130237  h
  -0.88384197958096   -4.91245972290871    1.73188667681165  h
  -1.28786749461744   -2.16333246044364    3.61291750715275  h
   1.84607969934653    3.24498151136577   -0.70120475555869  c
   3.04530950342361    5.36381680087182   -0.80731652237973  f
$end
```

Etot = -569.5197701889 H
ZPE = 299.2 kJ/mol
enthalpy = 324.77 kJ/mol
chem. pot. = 210.04 kJ/mol

4,5-dimethyl-1,2,3-trifluorobenzene

$coord
```
   2.90823125797853    -1.37928415430422    -0.04615882799575  c
   4.20871038182591     0.88656229307589     0.03049680672910  c
   0.29481207295791     3.11270678815541     0.10691057573564  c
  -1.07741242365690     0.87641970766113     0.03034544756096  c
   3.98594817308192    -3.11418791575051    -0.10601477731050  h
  -0.90813426205851     5.35319987085356     0.18358840561284  f
   6.74656209207625     0.91130053978217     0.02949724066424  f
   2.92032226583484     3.16088688857905     0.10883281569917  c
   4.16302756134548     5.36081786660039     0.18483771894522  f
   0.27608304341476    -1.40924590485256    -0.04679795740126  c
  -3.91981894751701     0.93340786350576     0.02992294322579  c
  -4.63412695578935     2.85721988192446     0.10068925314810  h
  -4.67536311240219     0.03057216448291    -1.66508330282618  h
  -4.67905564429094    -0.09298210913830     1.65127936697292  h
  -1.11955887517678    -3.88665359763188    -0.13151415347538  c
  -2.33857454397281    -4.11873359068899     1.51836295044427  h
  -2.33733635085773    -4.00619279714833    -1.79423811427498  h
   0.18568426720668    -5.47581379510599    -0.18495639145426  h
```
$end
Etot = -608.5414383100 H
ZPE = 341.3 kJ/mol
enthalpy = 370.61 kJ/mol
chem. pot. = 248.50 kJ/mol

1H-4,5-dimethyl-1,2,3-trifluorobenzeniumcation

$coord
```
   2.60580261755756    -1.49716572233956    -0.21762336358431  c
   0.03570477371650     3.16556145521692     0.08157556403895  c
  -1.28479040429569     0.85093839142446     0.08557659222881  c
   3.70200779506591    -3.21285623096890    -0.41858727169585  h
  -1.23465063854687     5.32352035667934     0.13189097184859  f
   2.61233162849977     3.23002973397990    -0.08983198277735  c
   3.84366636649373     5.33722672768128    -0.23456348681456  f
   0.04694125850908    -1.52834891593480    -0.09430598364114  c
  -4.07685169845684     0.90320453460447     0.20369986337426  c
  -4.83370367597662     2.78143550198405     0.51759535666545  h
  -4.83793160473882     0.16421829969656    -1.57712327053335  h
  -4.75120963349750    -0.38860382802873     1.66767302176431  h
  -1.41691126157406    -3.95760634944135    -0.21152425262565  c
  -2.48747631935929    -4.25984011840003     1.52554055596873  h
  -2.76323434638385    -3.94594445574578    -1.77209755111752  h
  -0.15478434926821    -5.55887158081338    -0.46459287474905  h
   4.09732199163664     0.85379189488528     0.09151637292292  c
   4.66588921142392     0.81525210041116     2.12123412630904  h
   6.23187828919456     0.92405820510904    -1.34605238758228  f
```
$end
Etot = -608.8272965980 H
ZPE = 369.6 kJ/mol
enthalpy = 399.80 kJ/mol
chem. pot. = 274.82 kJ/mol

2H-4,5-dimethyl-1,2,3-trifluorobenzeniumcation

```
$coord
   2.81152469253966    -1.61085454542698     0.09450985293267  c
   0.03072613028932     2.90143603575617    -0.31000692589775  c
  -1.30772489435659     0.72049081590221    -0.08191717297494  c
   3.81737596749573    -3.38640862452117     0.18521467387265  h
  -1.07956544152642     5.08587678705251    -0.60395835504356  f
   0.12828486934738    -1.56483884172537     0.14313081228368  c
  -4.14682104327113     0.70973861303624    -0.13016218718566  c
  -4.90222845649254    -0.03584181293173     1.63725260792431  h
  -4.89082714209848     2.60408737545138    -0.39385707480011  h
  -4.85202853531318    -0.47456523273715    -1.66193585035033  h
  -1.23589765549297    -3.99730788409614     0.38141821006965  c
  -2.54847386211006    -3.92570436772903     1.97820892375251  h
  -2.42521906309394    -4.29502959275688    -1.28721711370797  h
   0.02488999596003    -5.60017684440625     0.60364760121048  h
   2.84863644279753     3.09560848281979    -0.09411643051570  c
   3.20319810611356     3.84194803088621     1.83015074073499  h
   4.11409315399556     0.57117576332141    -0.15224592290812  c
   6.56470517719882     0.63192575344609    -0.32337997058682  f
   3.84535155801778     4.72844008865868    -1.81473641880997  f
$end
```

Etot = -608.8391758041 H
ZPE = 371.1 kJ/mol
enthalpy = 401.39 kJ/mol
chem. pot. = 275.75 kJ/mol

3H-4,5-dimethyl-1,2,3-trifluorobenzeniumcation

```
$coord
   2.83842411484141    -1.48551028016768     0.12721744540678  c
  -1.17538594596097     0.59745634105041    -0.04039708150948  c
   3.93398223805893    -3.21557998424311     0.22423408971226  h
   0.18138212308157    -1.63376052876310     0.12891709410622  c
  -3.94922897163448     0.78392424713949    -0.31379317403980  c
  -4.74281886763392     2.15485028141934     1.01040426400033  h
  -4.37671744876341     1.54005103600491    -2.19662646982478  h
  -4.89526485164484    -1.01989829267368    -0.08775890144964  h
  -1.08595452240841    -4.18304204030136     0.17335623784688  c
  -2.39603416548356    -4.33051519626633     1.75666558653736  h
  -2.15684361304536    -4.50230458967942    -1.55855626123839  h
   0.29322317029379    -5.69657449162841     0.34798641721999  h
   4.24401186928353     0.75089593135148    -0.06415987713099  c
   6.73619576059931     0.64313517462125    -0.17686671315158  f
   3.00101811309282     3.00491091589038    -0.22832092687178  c
   0.21814809464906     3.04598391172398     0.17434186110984  c
   0.10276967354691     3.39100314594862     2.25587337673946  h
  -0.92026291987548     5.00523236276238    -1.03613505806815  f
   4.14935614900322     5.14974205581085    -0.49638190939447  f
$end
```

Etot = -608.8245848083 H
ZPE = 368.7 kJ/mol
enthalpy = 399.54 kJ/mol
chem. pot. = 272.01 kJ/mol

4H-4,5-dimethyl-1,2,3-trifluorobenzeniumcation

```
$coord
   2.74627136804179   -1.53321101031697    0.53463291868911  c
   3.86186834937147   -3.24481228808351    0.58695348973357  h
   0.16535268079451   -1.56694886876505    0.52170619905169  c
  -1.27287721423076   -3.98145616883507    0.55542319740111  c
  -2.90600208629130   -3.87097672450733    1.80874990247382  h
  -2.00265414792493   -4.38439730018342   -1.33886228819714  h
  -0.08690206167117   -5.56220568826438    1.11844016791350  h
   4.04314817661261    0.77799638394599    0.50323844307375  c
   6.49733605627213    0.76296723016669    0.51031781235304  f
   2.84371598687219    3.15975138925006    0.49320162242503  c
   4.20718570906911    5.24165360991701    0.50657029383904  f
  -1.28268755731808    0.85536885560465    0.42084494140668  c
  -2.51430245434302    0.91862679028940    2.09520185233479  h
  -3.08536810576040    0.96090016706552   -1.93182512641450  c
  -4.44462192988507   -0.57321645617168   -1.82186670160583  h
  -4.10123455192284    2.74566606434735   -1.93395033981179  h
  -1.99659228050647    0.79046366646923   -3.66840498261367  h
   0.27041648091516    3.17696035931697    0.50018365710588  c
  -0.94205241809492    5.32686998875458    0.53944494084200  f
$end
```

Etot = -608.8462322807 H
ZPE = 372.8 kJ/mol
enthalpy = 402.66 kJ/mol
chem. pot. = 278.31 kJ/mol

5H-4,5-dimethyl-1,2,3-trifluorobenzeniumcation

```
$coord
   2.94362758756586   -1.42826646649955    0.46936902687466  c
   4.01771363306194   -3.16909478658675    0.46234302111678  h
   4.16269370730992    0.81104996412741    0.47158067513616  c
   6.64904163626064    1.01970638630629    0.47490929356196  f
   2.73645363189530    3.08628857336346    0.47570053717270  c
   3.92261022894575    5.22243587466963    0.47823986281584  f
   0.08430262558246    3.07841447079511    0.49738670180339  c
  -1.06581573325850    5.30235903544570    0.52320490060130  f
  -1.25201620708162    0.85928807979694    0.51102405013945  c
  -4.05734791688111    0.88087014092901    0.56357003465572  c
  -4.77520534942819    0.95154694111194   -1.37921231400131  h
  -4.81500798575169   -0.81575933748850    1.44458540454623  h
  -4.77617228080653    2.55201167162976    1.52321060280052  h
   0.15750750853281   -1.56553981841320    0.42548565974464  c
  -0.41602578210664   -2.63263168594254    2.12185341185166  h
  -0.72651491593453   -3.23886437210480   -1.86660598611692  c
   0.21071789142821   -5.06528616895518   -1.77828349570386  h
  -2.75629071163504   -3.53331918046655   -1.77932161411771  h
  -0.24427156769896   -2.31520932171822   -3.63903977288125  h
$end
```

Etot = -608.8383017022 H
ZPE = 371.3 kJ/mol
enthalpy = 401.27 kJ/mol
chem. pot. = 276.83 kJ/mol

6H-4,5-dimethyl-1,2,3-trifluorobenzeniumcation

$coord

4.10820939268861	1.10136076700101	0.02309292360856	c
0.12001354835713	3.13420774933854	-0.02658441632337	c
-1.31463084909864	0.88371674853696	-0.02029489386027	c
-1.08596956924857	5.27957517316268	-0.05421569271599	f
6.57441174674850	1.09535135078099	0.04292017154458	f
2.78668296603869	3.30273168288973	-0.00621799739320	c
3.87878956995985	5.53869859793127	-0.01575580901706	f
0.00630378890583	-1.35463825492900	0.01287872307002	c
-4.14587518011512	1.11719184365367	-0.04532226639977	c
-4.80585914524534	2.09282610989210	1.64697452251309	h
-4.76678162381856	2.22805770050126	-1.66636410254293	h
-5.05985233952160	-0.71593744518555	-0.13089000756719	h
-1.25708834645403	-3.87332480569810	0.02517026736391	c
-2.51773357270001	-4.04628708031275	1.65123125679607	h
-2.43155581304070	-4.10078587220303	-1.65873878126849	h
0.09833006777382	-5.41680573314910	0.08382464079773	h
2.81671144663039	-1.35579692051152	0.03549061354980	c
3.48513729999909	-2.43936498065076	1.68044429969472	h
3.51075661214064	-2.47077663104836	-1.57764345185011	h

$end

Etot = -608.8503522643 H

ZPE = 369.4 kJ/mol

enthalpy = 400.51 kJ/mol

chem. pot. = 268.22 kJ/mol

4,6-dimethyl-1,2,3-trifluorobenzene

$coord

1.83931601292887	-1.44585849344794	0.07051627195986	c
3.10255701791368	0.85133038280362	-0.03510664214058	c
5.64553775290080	0.90586293516238	-0.03526384205854	f
1.81045464724049	3.12947916676221	-0.14221028934710	c
3.07488441215010	5.31521504587968	-0.24232588428237	f
-0.81077229178748	3.11019728530003	-0.14443477177147	c
-2.03345749624828	5.33823479502038	-0.24925737806060	f
-2.17249833111385	0.86973597514928	-0.04197361709896	c
-5.01338701382353	0.93430423043952	-0.04556810960166	c
-5.73506754989747	1.88710060088137	-1.72620059113258	h
-5.78502951309417	-0.97237025892736	0.00079651575108	h
-5.73703675808719	1.96697335145693	1.58684702698671	h
-0.79529811756125	-1.37493707354956	0.06430489819158	c
-1.81918180508234	-3.14460768257980	0.14567505487650	h
3.31355391463566	-3.87264833871411	0.17882169496219	c
4.47281311987379	-4.11525481765979	-1.51016996604316	h
4.59499403927037	-3.89546410300743	1.79424425825529	h
2.04761795978186	-5.48729300096936	0.33130537055376	h

$end

Etot = -608.5428781342 H

ZPE = 340.7 kJ/mol

enthalpy = 370.75 kJ/mol

chem. pot. = 245.99 kJ/mol

1H-4,6-dimethyl-1,2,3-trifluorobenzeniumcation
$coord
1.59633810745181 -1.60369135487205 0.06259070713240 c
1.54552650836730 3.16969120580885 -0.36162615620236 c
2.80715231688464 5.24515220900018 -0.70685781923795 f
-1.01889970977846 3.12765028867812 -0.24506531543585 c
-2.33629509170228 5.24447357870562 -0.48322758461794 f
-2.32677130334350 0.80868684692341 0.04560389735792 c
-5.12114324326528 0.87649555683278 0.11676379177700 c
-5.83824758631111 1.67299331585229 -1.65523111062186 h
-5.94162668947664 -0.97889978460369 0.42096020628617 h
-5.75984271082738 2.18347075630230 1.58725011395447 h
3.08175974452080 -3.96756312019445 -0.05164140959267 c
4.17191110486157 -4.00204144904021 -1.81036696037788 h
4.48096885091230 -4.03634115103661 1.46807141779329 h
1.88226615883046 -5.63128762923498 0.04556827736460 h
-0.97798312211045 -1.49802954520012 0.18610247338734 c
-2.05655167304564 -3.23075956609558 0.29410269492507 h
3.04137723393206 0.83110799106676 0.14547282200624 c
3.55245065679774 1.01654966690295 2.17559140534989 h
5.21761044730193 0.77234218420458 -1.23406145124787 f
$end
Etot = -608.8366224308 H
ZPE = 368.7 kJ/mol
enthalpy = 399.40 kJ/mol
chem. pot. = 273.01 kJ/mol

2H-4,6-dimethyl-1,2,3-trifluorobenzeniumcation
$coord
1.83417054290148 -1.61086719250693 0.02898800336363 c
-1.00015485996107 3.00291031505710 -0.19140354590096 c
-2.11582596836675 5.18457752545155 -0.41578729143981 f
-2.31366935768460 0.79412476104281 0.01891922509761 c
-5.15103692940207 0.67562966061000 0.03035249672409 c
-5.83450673705313 -0.26986622259456 -1.66945598665931 h
-5.81437705082647 -0.39551257221331 1.66047597881701 h
-5.98226996325297 2.55200199957939 0.10093728907628 h
3.15722036792230 -4.12405091924877 0.03166031714683 c
3.03224927562025 -4.99993975929934 -1.83154532999347 h
5.14599604117613 -3.92240207079987 0.50563630977483 h
2.28209219267408 -5.39739481467059 1.39174983941911 h
-0.82682167699456 -1.42242258732730 0.13699706400102 c
-1.85452071341898 -3.19240765985388 0.28771963149073 h
1.81566996285302 3.13998717451921 0.01502976579819 c
2.13797921729615 3.70135213882577 2.01510547504825 h
3.09614331181550 0.62870718934050 -0.18475275174889 c
5.54302791273945 0.73989843932636 -0.40859127800724 f
2.84863443196220 4.91567459476183 -1.52203521200795 f
$end
Etot = -608.8315987317 H
ZPE = 370.1 kJ/mol
enthalpy = 401.79 kJ/mol
chem. pot. = 268.95 kJ/mol

4H-4,6-dimethyl-1,2,3-trifluorobenzeniumcation

```
$coord
  -0.83092409087799    3.00907518258007    0.37655322057744  c
  -2.05772826379590    5.13595776553273    0.32014995618357  f
  -2.23777726880331    0.72259510407164    0.50729854208268  c
  -5.06986777754869    0.84393938498032    0.58447971850183  c
  -5.80725737966000    1.77526802638710   -1.10011123540870  h
  -5.87065851838422   -1.04477896855525    0.69623139387631  h
  -5.71045165974768    1.93043387904986    2.21470680150204  h
  -0.85698902037420   -1.44350420057839    0.55265015663729  c
  -1.81152726398049   -3.25118578443448    0.64724176215958  h
   3.16358061316230    0.95732574478793    0.36877017084998  c
   5.62602367872179    1.01882271929530    0.33021507938825  f
   1.82260703334235    3.16487732166264    0.31272705718333  c
   1.93303753088293   -1.53275660882483    0.44439307101605  c
   2.59463319731222   -2.46011263553549    2.19123319581803  h
   2.88096218843763   -3.24890566855862   -1.78062521334183  c
   4.93179260763659   -3.35404663008208   -1.73925840835758  h
   2.10580168981007   -5.13746109127570   -1.54805837947303  h
   2.26622572048457   -2.47726721422115   -3.58429678569196  h
   2.92851698338203    5.39172367371844    0.20569989649677  f
$end
```

Etot = -608.8426189999 H
ZPE = 372.7 kJ/mol
enthalpy = 402.71 kJ/mol
chem. pot. = 278.23 kJ/mol

5H-4,6-dimethyl-1,2,3-trifluorobenzeniumcation

```
$coord
   2.01020594998613   -1.36491676377714   -0.02208476959534  c
   3.22334635393806    0.90760450698795   -0.02741823325834  c
   5.72156867379296    1.07023720342473   -0.05027690648342  f
   1.84610809661709    3.18763659971069    0.00437076243235  c
   3.07383783793567    5.30529688825098    0.00579810148808  f
  -0.81868668371146    3.25094980327841    0.03318698829035  c
  -1.91143519968269    5.50348734649816    0.04779599845593  f
  -2.18915534702346    1.07035388855344    0.03222957172332  c
  -4.99766329163062    1.08625970630983   -0.00179340854091  c
  -5.65689220951148    1.34917597243840   -1.94716414637159  h
  -5.77707268236771   -0.68556751628895    0.69273354549329  h
  -5.74908848853514    2.65892158430338    1.09342952784113  h
   3.42687887992875   -3.79039811722038   -0.00566458512978  c
   4.97140330397853   -3.74659169989695   -1.37092563689550  h
   4.30163734559649   -4.07129700954396    1.84870502485746  h
   2.20350332604780   -5.39511170023634   -0.39562795029019  h
  -0.78993357956452   -1.35599288864966    0.01244137622359  c
  -1.47016744898744   -2.49277544921346   -1.59258176467343  h
  -1.41839483680698   -2.48727235492922    1.64284650443294  h
$end
```

Etot = -608.8506930910 H
ZPE = 367.0 kJ/mol
enthalpy = 398.71 kJ/mol
chem. pot. = 267.14 kJ/mol

trimethyl-1,2,3-trifluorobenzene

$coord

-0.41731945717032	-0.74882837176417	0.05015869314247	c
2.23733402930282	-0.77117352205294	0.01520428625269	c
3.49129239880619	1.53613928991057	-0.08184121531829	c
2.20786874109374	3.81406481902700	-0.15270953674392	c
-0.40854929857513	3.78351338008915	-0.12914042690478	c
-1.76450524886703	1.53999458703749	-0.03413678468067	c
-1.61554022637487	6.02432333917113	-0.20332248735073	f
3.47077637965272	6.00339775669749	-0.24430409003971	f
6.03648856496801	1.62439755370933	-0.11340556810838	f
-4.60897079696887	1.60948644944390	-0.02391717066276	c
-5.36299579417067	0.90121751733295	1.76294842753714	h
-5.31066736541494	3.52073394700953	-0.29078720189130	h
-5.38528652660394	0.42317195031763	-1.52159743980508	h
-1.89230705784792	-3.18613896853561	0.14914638365772	c
-3.35156526442087	-3.11682650274301	1.60404466143036	h
-2.85671974227044	-3.54060011019758	-1.64416617033825	h
-0.70201443248627	-4.81075339575232	0.54191144816940	h
3.76256833161413	-3.17842255621963	0.06784747517692	c
3.43546387657940	-4.23332179024161	1.81038008066842	h
3.26133327737385	-4.41230200177445	-1.50628873277855	h
5.77331561178050	-2.78207337046495	-0.05602463141268	h

$end

Etot = -647.8451155601 H

ZPE = 413.7 kJ/mol

enthalpy = 447.48 kJ/mol

chem. pot. = 314.93 kJ/mol

1H-trimethyl-1,2,3-trifluorobenzeniumcation

$coord

-0.48160971009341	-0.95701365636280	-0.00751918617398	c
2.22147794085122	-0.90624092211619	-0.09390060654049	c
3.58979897620767	1.39335937143099	0.00041623683911	c
2.36398116157964	3.64313738746739	0.11085671494642	c
-1.80617052174094	1.27551030493268	0.07945758823277	c
3.55231204708795	5.78325847219354	0.28883053732660	f
6.09582244688140	1.34175339378773	0.06284304137968	f
-4.60691276584370	1.46283491466160	0.27119391923409	c
-5.23835382259091	0.65738832547865	2.06749717575415	h
-5.25668761297578	3.40713486520051	0.18657624800658	h
-5.52498668740603	0.37434075853813	-1.22258550599047	h
-1.79200505135999	-3.48156047100088	0.05340318256435	c
-3.80289736959818	-3.29921213608383	0.40554077333958	h
-1.56030739245121	-4.46983288483272	-1.74355807653745	h
-1.00844205736119	-4.69231556026662	1.52406184745098	h
3.69330416640230	-3.29027950266401	-0.20085353883261	c
3.56596550915580	-4.23577274383238	1.63983424568852	h
2.87545577019561	-4.59707796525189	-1.56846224646229	h
5.67549282351532	-2.97766519757449	-0.61962083310595	h
-0.42425404728332	3.73228286712773	-0.19436368093315	c
-0.67584675185909	4.18667423571335	-2.23259024785433	h
-1.45513705131322	5.64929614345345	1.19294241166791	f

$end

Etot = -648.1425063730 H

ZPE = 441.6 kJ/mol

enthalpy = 476.30 kJ/mol

chem. pot. = 340.84 kJ/mol

2H-trimethyl-1,2,3-trifluorobenzeniumcation

```
$coord
  -0.49707998539353   -0.90939646185419    0.00785819640392  c
   2.21221717861809   -0.99499034341031    0.05129656722747  c
   3.44250098461921    1.25826727827491    0.07367246760112  c
  -1.94040752602348    1.38926930611814   -0.00814951643894  c
   5.90493751479503    1.40439452206237    0.20359552466990  f
  -4.78225086768928    1.38391895291262    0.02137900740428  c
  -5.49928880957346    0.56834803576660    1.77384590451572  h
  -5.52245512165008    3.29166249426960   -0.13547208828011  h
  -5.53433501480950    0.27283504564326   -1.54142925633077  h
  -1.93910176078824   -3.31257206978148   -0.00839132616617  c
  -3.40298954323213   -3.28720612601258    1.44703563267712  h
  -2.96210834008610   -3.46695276517255   -1.80474626476160  h
  -0.77933605668848   -4.98111886488913    0.22380773812592  h
   3.66775261925535   -3.43833651629846    0.13160559190536  c
   3.21155444588827   -4.51454489580355    1.82837947425456  h
   3.22697001776315   -4.60782041832409   -1.50611592773138  h
   5.68943466061216   -3.08533121495506    0.13338607292261  h
  -0.62908830039358    3.59255640142888    0.01783854268647  c
  -1.75628817120741    5.78982049359025    0.10339520169694  f
   2.17611881250219    3.76239819226903   -0.20621992686729  c
   2.54063971257556    4.34840210516841   -2.18267388818435  h
   3.17260355090636    5.53639684899731    1.37610227266910  f
$end
```

Etot = -648.1485567012 H
ZPE = 443.8 kJ/mol
enthalpy = 478.34 kJ/mol
chem. pot. = 342.58 kJ/mol

4H-trimethyl-1,2,3-trifluorobenzeniumcation

```
$coord
  -0.53371324018049   -0.89903237375317   -0.42765323495350  c
   3.57463773635666    1.46040342636671   -0.58791174582592  c
  -1.82788514421608    1.35973517888545   -0.46689086505286  c
   6.04480206148169    1.42979096361405   -0.67226438555171  f
  -4.66635783130216    1.51706285346500   -0.46039383226105  c
  -5.44475004218113    0.45105803379670    1.12109514932231  h
  -5.32519278205946    3.45479019973754   -0.31513348894667  h
  -5.43814573401414    0.71078241513809   -2.19470069979700  h
  -1.91762654553463   -3.34856321915714   -0.43706939240076  c
  -2.97338371068411   -3.58611183663272    1.32610229578155  h
  -3.32164672621679   -3.37236960288587   -1.94977656792252  h
  -0.67070140412705   -4.96270982308872   -0.67229956663830  h
  -0.36938043121753    3.58617013909322   -0.56810123719823  c
  -1.51340495255591    5.76697849309867   -0.62753865934143  f
   2.27896150036662   -0.98906060758060   -0.32705517384096  c
   2.94756920581249   -2.21335212408873   -1.86508646370229  h
   3.18974625505309   -2.26242469005859    2.20481802873544  c
   5.24130277114166   -2.36063351561028    2.20705761784132  h
   2.55318310277872   -1.15934685591942    3.81940385982989  h
   2.43098019788122   -4.16457679279760    2.33675462502959  h
   2.29919396490525    3.68586649063229   -0.64957061227427  c
   3.44181174851199    5.89554324774519   -0.79378565083270  f
$end
```

Etot = -648.1564288299 H
ZPE = 444.8 kJ/mol
enthalpy = 479.13 kJ/mol
chem. pot. = 345.14 kJ/mol

5H-trimethyl-1,2,3-trifluorobenzeniumcation

```
$coord
   3.47878273410451    1.36895608496161   -0.38929523939041  c
  -1.91647409491802    1.50286482947762   -0.48462563111169  c
   5.97965003123751    1.54716281113533   -0.39006286795515  f
  -4.72437839564557    1.55378814623232   -0.60391424727995  c
  -5.50101735268892    1.81510532798600    1.29733048562947  h
  -5.38232600064747    3.14705870363210   -1.72926716200804  h
  -5.48799335453636   -0.19572780379861   -1.36703800643215  h
  -0.55986705975210    3.69097352196818   -0.42468963460065  c
  -1.66707738976826    5.94011631234792   -0.45830058226863  f
   2.09997658688117    3.64556880477953   -0.37129244618903  c
   3.32029826420665    5.76847584645994   -0.34297287182744  f
   2.27192306983319   -0.90511347949171   -0.44430023749672  c
   3.72693956264338   -3.30919406654873   -0.51345035047363  c
   4.21314417923629   -3.88660142680730    1.41459511949013  h
   2.64046204872291   -4.83048199327816   -1.37066813272720  h
   5.50326085894254   -3.06892379302063   -1.52369742195698  h
  -0.54031034810223   -0.94756690190548   -0.35580553368793  c
  -1.18608424700945   -2.09497002910314   -1.96188556545801  h
  -1.41250082608226   -2.42312934697782    2.07933499443040  c
  -0.57269606389909   -4.29617563981835    2.09453748435800  h
  -0.82807362568419   -1.40120042491625    3.76579393289974  h
  -3.45563857707431   -2.62098548331440    2.07967391405580  h
$end
```

Etot = -648.1553202684 H
ZPE = 443.5 kJ/mol
enthalpy = 477.86 kJ/mol
chem. pot. = 343.85 kJ/mol

1,2-difluorobenzene

```
$coord
   1.31302604494639    0.00000000000000   -2.17453051221147  c
  -1.31302604494639    0.00000000000000   -2.17453051221147  c
  -2.63792204727101    0.00000000000000    0.07840144570120  c
  -1.31381884439180    0.00000000000000    2.35109250678418  c
   1.31381884439180    0.00000000000000    2.35109250678418  c
   2.63792204727101    0.00000000000000    0.07840144570120  c
  -4.67999866420115    0.00000000000000    0.01811387504152  h
  -2.34289136743646    0.00000000000000    4.11612150823824  h
   2.34289136743646    0.00000000000000    4.11612150823824  h
   4.67999866420115    0.00000000000000    0.01811387504152  h
   2.55314575760179    0.00000000000000   -4.38933164281983  f
  -2.55314575760179    0.00000000000000   -4.38933164281983  f
$end
```

Etot = -430.6862496890 H
ZPE = 218.6 kJ/mol
enthalpy = 236.85 kJ/mol
chem. pot. = 141.08 kJ/mol

1H-1,2-difluorobenzeniumcation
$coord

1.43961770171065	2.44371060680316	0.23399361053900	c
-1.19091304801471	2.57145263747046	0.10097479848088	c
-2.48906388428733	0.35451838703823	-0.23703189187247	c
2.47680210229449	4.19942751026511	0.42778768420807	h
-2.19024048064422	4.35341660349366	0.13823447843325	h
-4.91024925714493	0.30974611231579	-0.54565004277557	f
1.59584549723031	-2.06360726034010	-0.24752515454687	c
2.54924409685642	-3.85961426004499	-0.47585626624596	h
2.83538372138096	0.15810323830655	0.05223650861046	c
4.87651929788282	0.22806139725225	0.07154530537844	h
-1.19890902215781	-2.14094264103195	-0.06338562594812	c
-1.47180690393541	-2.57714682364984	1.99384232573473	h
-2.32222982117119	-3.97712550787829	-1.44916572999583	f

$end
Etot = -430.9612355453 H
ZPE = 247.5 kJ/mol
enthalpy = 266.79 kJ/mol
chem. pot. = 166.86 kJ/mol

3H-1,2-difluorobenzeniumcation
$coord

-2.25324116324002	-1.08116076022719	0.00000000000000	c
2.45918475197037	-0.94155943767794	0.00000000000000	c
2.34726254274225	1.62554751438135	0.00000000000000	c
-0.02870852891567	2.84431280840541	0.00000000000000	c
-2.30350811692733	1.52304275031290	0.00000000000000	c
4.24862886815044	-1.93057359419247	0.00000000000000	h
4.04561119481173	2.75941104147036	0.00000000000000	h
-0.13705332112947	4.89094305258357	0.00000000000000	h
-4.47666027106348	2.74306274722699	0.00000000000000	f
-4.33788172260019	-2.37373188023507	0.00000000000000	f
0.13892767457475	-2.47898105737823	0.00000000000000	c
0.14871904581332	-3.79015659233485	-1.62176316129782	h
0.14871904581332	-3.79015659233485	1.62176316129782	h

$end
Etot = -430.9757903162 H
ZPE = 246.0 kJ/mol
enthalpy = 265.66 kJ/mol
chem. pot. = 165.19 kJ/mol

4H-1,2-difluorobenzeniumcation
$coord

-2.39842950984992	-1.34233094714170	0.00000000000000	c
2.05716727498228	1.61153866630302	0.00000000000000	c
-0.21081282970076	2.82006531547465	0.00000000000000	c
-2.42017001977315	1.34457218930175	0.00000000000000	c
3.80067563574832	2.68174428764069	0.00000000000000	h
-0.39527490710075	4.85549410654609	0.00000000000000	h
-4.59603560736488	2.46090232696769	0.00000000000000	f
-4.59300479949517	-2.52090701007372	0.00000000000000	f
2.22712772106607	-1.15833167373883	0.00000000000000	c
3.40642947449169	-1.77302568387852	-1.60888369000245	h
3.40642947449169	-1.77302568387852	1.60888369000245	h
-0.15907159377998	-2.58045747863103	0.00000000000000	c
-0.12503031371540	-4.62623841489157	0.00000000000000	h

$end
Etot = -430.9801399267 H
ZPE = 246.1 kJ/mol
enthalpy = 265.58 kJ/mol
chem. pot. = 165.48 kJ/mol

3-methyl-1,2-difluorobenzene

```
$coord
  -0.01391340887065    -2.14757050322651    -0.13262637512623  c
   2.60812416359910    -2.16920269231710    -0.12876322485065  c
   3.95985557646030     0.06378488708711     0.00219575386376  c
   2.63771148718880     2.33016512436973     0.13414020876107  c
   0.01051152584858     2.33968523949196     0.13884012552823  c
  -1.37153194850509     0.09704698759768     0.00604483108055  c
   6.00089006326918    -0.00952789229163     0.00204778415750  h
   3.66554528548985     4.09301800420504     0.23745954823264  h
  -0.99999909156407     4.11405892544991     0.24922871467159  h
  -4.21149467165560     0.03314914002404     0.00395556970986  c
  -4.98423765630580     1.91922483852626     0.28304428911930  h
  -4.93585091105424    -0.70747351886472    -1.78044319666929  h
  -4.92826824520192    -1.19575063163510     1.49624488169151  h
  -1.26006119878552    -4.36355392269122    -0.26403701790587  f
   3.82271903008716    -4.39705398572555    -0.24733189226406  f
$end
```

Etot = -469.9933163800 H
ZPE = 290.3 kJ/mol
enthalpy = 313.18 kJ/mol
chem. pot. = 204.94 kJ/mol

3H-3-methyl-1,2-difluorobenzeniumcation

```
$coord
  -0.11093197381230    -2.26348832939520    -0.60918386795884  c
   2.49199737877306    -2.28437094999058    -0.68880752722190  c
   3.78933048303658     0.00147735921125    -0.67914456779449  c
   2.53195937006666     2.35340535882228    -0.60061696418266  c
  -0.03589779017273     2.41588220590557    -0.51486863057773  c
   5.83591505721058    -0.07809480176363    -0.74368385602024  h
   3.63468353599404     4.07212335793213    -0.61116976316984  h
  -1.04580202094847     4.19355038072287    -0.45239453266532  h
  -1.35939209538718    -4.38109968374082    -0.63390777476016  f
   3.73491242514255    -4.44676283033637    -0.77372848198318  f
  -1.57230149442500     0.09221623290020    -0.47008820620450  c
  -2.79817969061824     0.12317676581092    -2.16021026750134  h
  -3.44617380359071     0.05350890598362     1.84152247447205  c
  -4.64675141259593     1.71775282520168     1.75142548381078  h
  -4.61338148875918    -1.63286385375863     1.74045409376264  h
  -2.38998647991379     0.06358705649474     3.60440238799476  h
$end
```

Etot = -470.2831995481 H
ZPE = 320.7 kJ/mol
enthalpy = 344.23 kJ/mol
chem. pot. = 234.52 kJ/mol

4H-3-methyl-1,2-difluorobenzeniumcation

```
$coord
  -0.06652546109389    -2.37028520385666    -0.03168375098941  c
   2.59270086746079    -2.33362850582797    -0.05049310012051  c
```

4.03535156410074	-0.08982340757021	-0.02560579290937	c
2.78199745322380	2.13968595828550	0.03147149114021	c
-1.41850728309880	-0.16686099683680	0.02301465660113	c
6.07286160235095	-0.23886706305453	-0.04933644241566	h
3.80418119844967	3.91072180753541	0.05932543037525	h
-4.22390579731386	-0.17363401353896	0.00168610097507	c
-5.00016723963958	1.65820187895359	0.51693790307806	h
-4.89120609988881	-0.64983272975590	-1.90012568158629	h
-4.96893774566768	-1.63165584296162	1.25377216142620	h
-1.20957793196178	-4.59703066329946	-0.07535596831476	f
3.76545853066842	-4.48821456038879	-0.09618172486294	f
0.00109773153800	2.24187739331539	0.07426047708997	c
-0.60354345092138	3.33104738170954	1.74582924517005	h
-0.67127793820656	3.45829856729130	-1.47751500465700	h

$end
Etot = -470.2991267779 H
ZPE = 317.3 kJ/mol
enthalpy = 341.60 kJ/mol
chem. pot. = 229.50 kJ/mol

5H-3-methyl-1,2-difluorobenzeniumcation
$coord

-0.19062750009017	-2.27992696494176	-0.11166331953672	c
2.49177575383835	-2.38696101082442	-0.12235214279090	c
3.84520598508396	-0.22050314166775	-0.01983759945072	c
-1.62257015118895	-0.02583223117354	0.00326109232695	c
5.88954019193558	-0.28038427431875	-0.02757183166553	h
-4.45377747945245	-0.16386935366408	0.00252469532124	c
-5.27208715077569	1.71632398985867	0.12796719929807	h
-5.13727750153762	-1.06921906244866	-1.71881624642650	h
-5.12899175409128	-1.28752177003034	1.59315507556810	h
-1.38547723620520	-4.42028359658585	-0.21467362292354	f
3.55622110274775	-4.64005830112128	-0.23387668625699	f
-0.25343767265671	2.16135604908681	0.10793130054171	c
-1.22565291074075	3.96005285616015	0.19980170375741	h
2.52009955557131	2.21195857202768	0.10241820419438	c
3.18866628524623	3.28467105599040	1.76325897567642	h
3.17839048231556	3.44019718365269	-1.45152679763339	h

$end
Etot = -470.2938079636 H
ZPE = 317.8 kJ/mol
enthalpy = 341.80 kJ/mol
chem. pot. = 231.72 kJ/mol

6H-3-methyl-1,2-difluorobenzeniumcation
$coord

-0.33937705498782	-2.22337855825689	-0.08233695967153	c
2.24581664223326	-2.27283519935907	-0.09164582325445	c
-1.65031932950971	0.08880393292324	0.02024364767871	c
-4.44765931361002	0.04520807950653	0.01747450158476	c
-5.25541148906989	1.90902221855656	0.30697125924346	h
-5.11565482022222	-0.71335841895870	-1.79132928004647	h
-5.15020885652930	-1.26847470516813	1.44881418473136	h
-1.63433277750546	-4.36469786190849	-0.16760358787907	f
3.45614087881835	-4.41534763842478	-0.17990325618978	f
-0.27161573677304	2.40094950245219	0.10619789308901	c
-1.32419871485405	4.15022664575386	0.18294151560963	h

2.28742087547878	2.41579642605218	0.09503970160574	c
3.33166183006336	4.17246130654462	0.16042752003561	h
3.75608048367876	0.05081293064588	-0.00416511432720	c
5.04977813378282	0.07726814070653	-1.63621085842187	h
5.06187924900631	-0.05245680106565	1.61508465621208	h

$end
Etot = -470.2973419865 H
ZPE = 317.3 kJ/mol
enthalpy = 341.54 kJ/mol
chem. pot. = 229.81 kJ/mol

4-methyl-1,2-difluorobenzene
$coord

-0.65138068139780	-3.31070540390344	0.03129140061203	c
1.97460158633958	-3.33142263582575	0.00360696623377	c
3.29206774880240	-1.07982982704162	-0.02726884585301	c
1.96836591117456	1.19520583629746	-0.03007845988169	c
-0.66456599600877	1.24467424589538	-0.00251140639863	c
-1.96334156181761	-1.05528887005609	0.02786313793122	c
5.33445880674837	-1.13143650325676	-0.05008882734946	h
3.01318517163398	2.95285805197036	-0.05478611912796	h
-1.90897323249740	-5.51712760815824	0.05886663766022	f
3.20713280112883	-5.55295901991100	0.00570004438920	f
-4.00718475663217	-1.12111380838421	0.04873555122822	h
-2.10892416061468	3.69790017097639	-0.00312486968045	c
-3.36124691196777	3.82398397559428	-1.63829341294746	h
-3.28604269081900	3.86861398613129	1.68324618587700	h
-0.83815203407248	5.31664740967184	-0.05315798269306	h

$end
Etot = -469.9919629670 H
ZPE = 289.5 kJ/mol
enthalpy = 312.77 kJ/mol
chem. pot. = 201.70 kJ/mol

3H-4-methyl-1,2-difluorobenzeniumcation
$coord

-1.81961653577995	-3.53468611842758	0.00172313220212	c
0.81965444727820	-3.44948671008902	0.00285192522399	c
2.19496207993205	-1.18379855190342	0.00152560138594	c
0.98223615917842	1.10941662968791	-0.00062074303563	c
-3.13140415824958	-1.30450514507856	-0.00032531453669	c
1.80686256475255	-5.24532315513311	0.00422000720556	h
4.23575672450700	-1.27239099237675	0.00192645840767	h
-5.59204051838574	-1.27394696908896	-0.00359213330272	f
-3.01027015402368	-5.72920600960614	0.00189848884477	f
2.34607201458771	3.56171978822956	-0.00249058096197	c
1.80640710589101	4.68954200209386	-1.64846985034177	h
1.82937120201082	4.67730929468197	1.65949963711455	h
4.38380036185293	3.30454921629959	-0.01665954106921	h
-1.82604229481257	1.14949594212116	-0.00048415920483	c
-2.51277304208608	2.25080598070347	1.62568310018972	h
-2.51297595665305	2.25050479788602	-1.62668602812154	h

$end
Etot = -470.2934741462 H
ZPE = 317.1 kJ/mol
enthalpy = 341.75 kJ/mol
chem. pot. = 228.15 kJ/mol

4H-4-methyl-1,2-difluorobenzeniumcation

```
$coord
  -0.78155023605351    -3.27296812842695    -0.64333524397774  c
   1.90085808968200    -3.20826924902962    -0.69428575768051  c
   3.29290409218720    -0.94839674687059    -0.65548604262021  c
   1.99971906688090     1.26980309778721    -0.53981813666050  c
  -2.08682794013138    -1.07605745366003    -0.52649850933474  c
   5.33294159090798    -1.05486556715443    -0.71596314151782  h
   3.01466150467596     3.04653364642490    -0.50593232160704  h
  -4.13232973880071    -1.11955922203910    -0.47724627966780  h
  -1.89333378276806    -5.50526610641571    -0.70238809681014  f
   3.09214858451231    -5.34438161361488    -0.78881573685518  f
  -0.77667515122859     1.37851649677204    -0.44031729102222  c
  -1.37281586317119     2.43072753189582    -2.14759145420732  h
  -1.72779420235258     3.05387606930082     1.81768100730392  c
  -0.86874586310162     4.91736518892098     1.70211772779366  h
  -3.77025140311059     3.25319134344233     1.70980772230484  h
  -1.22290874812808     2.17975071266722     3.60807155455883  h
$end
```

Etot = -470.2862595296 H
ZPE = 320.7 kJ/mol
enthalpy = 343.97 kJ/mol
chem. pot. = 234.65 kJ/mol

5H-4-methyl-1,2-difluorobenzeniumcation

```
$coord
  -2.05842607199063    -3.38436967674793    -0.03257533497598  c
   0.48805985083202    -3.42711521737251    -0.07113333580504  c
   0.51633752771352     1.36761770654495     0.04713693582309  c
  -3.32714171222556    -1.00890690157725     0.06474633737879  c
   1.48486712820852    -5.21230378458043    -0.12787632863220  h
  -5.77818559883155    -1.04312180712401     0.11252546863616  f
  -3.46102755491007    -5.44694767577501    -0.07270107401390  f
   1.95233627557779     3.77824102193916    -0.01441080696228  c
   2.52187989361965     4.16514085520711    -1.96870211319911  h
   0.82250298585124     5.36845782177710     0.63323513957627  h
   3.68646477109549     3.66236335734739     1.09272609897838  h
   1.93572849464823    -1.04589715514718    -0.00787736034694  c
   3.20652603913435    -1.08838764371649     1.64366929972178  h
   3.29351258461121    -1.01021448036026    -1.58403945320113  h
  -2.07655762683266     1.31172018821163     0.10864532502461  c
  -3.20687698650150     3.01372339137392     0.17663120199753  h
$end
```

Etot = -470.2982377919 H
ZPE = 317.3 kJ/mol
enthalpy = 341.60 kJ/mol
chem. pot. = 229.80 kJ/mol

6H-4-methyl-1,2-difluorobenzeniumcation

```
$coord
  -2.08176045284411    -3.13938183462995     0.00014385085856  c
   0.76725041815330     1.44895974223395     0.00001979665846  c
  -3.32274246287806    -0.85259956587067     0.00088716913257  c
  -5.81669169576537    -0.76625320949760     0.00006728331662  f
  -3.33065195853075    -5.25362136210336    -0.00238958274935  f
   2.08582994820640     3.96199279220746    -0.00182690909270  c
   1.54636988072054     5.06245283906038    -1.66010471180108  h
```

1.56199151117440	5.05674526095311	1.66526494746316	h
4.12866696135841	3.73730342850763	-0.01176364582126	h
-1.91690324781812	1.36766355465606	0.00150219582270	c
-2.97947462981724	3.12203142413407	0.00213157620010	h
2.01666501428953	-0.80931158948648	-0.00001648397206	c
4.06083262777613	-0.87476837964292	-0.00139337538084	h
0.68046068734730	-3.25066718038122	0.00139756062301	c
1.30097696047867	-4.40702288376207	-1.61924810894261	h
1.29918043814893	-4.40352303637838	1.62532843768473	h

$end
Etot = -470.2877719875 H
ZPE = 317.2 kJ/mol
enthalpy = 341.47 kJ/mol
chem. pot. = 230.48 kJ/mol

3,6-dimethyl-1,2-difluorobenzene
$coord

0.00148031733469	-1.31396437637717	-2.33950311678008	c
0.00228171109932	-2.69196820772449	-0.09485803831056	c
0.00055258361243	-1.30924911732291	2.13816943335163	c
-0.00055258361243	1.30924911732291	2.13816943335163	c
-0.00228171109932	2.69196820772449	-0.09485803831056	c
-0.00148031733469	1.31396437637717	-2.33950311678008	c
0.00294063820650	-2.32410985065858	-4.11806944394755	h
-0.00294063820650	2.32410985065858	-4.11806944394755	h
0.00048022956177	-2.53142404910599	4.37305073706866	f
-0.00048022956177	2.53142404910599	4.37305073706866	f
-0.00202084889302	5.53110693104047	-0.01318005090021	c
-1.63664296915288	6.24759816616353	1.02063531504360	h
1.67568524916351	6.24853468376649	0.94929773527259	h
-0.04225675928237	6.31405888745343	-1.91554257079813	h
0.00202084889302	-5.53110693104047	-0.01318005090021	c
1.63664296915288	-6.24759816616353	1.02063531504360	h
-1.67568524916351	-6.24853468376649	0.94929773527259	h
0.04225675928237	-6.31405888745343	-1.91554257079813	h

$end
Etot = -509.3001833682 H
ZPE = 361.9 kJ/mol
enthalpy = 389.52 kJ/mol
chem. pot. = 272.38 kJ/mol

1H-3,6-dimethyl-1,2-difluorobenzeniumcation
$coord

-0.23701933129542	-1.29086497689567	-2.24588567071177	c
-0.00411079177775	-2.76186423491497	-0.03928566398063	c
0.21510992727600	-1.40308910606373	2.15152867791442	c
0.01872729964522	2.77768165354239	-0.18884948667255	c
-0.21554627043928	1.36662962928071	-2.34329128469551	c
-0.37717839467399	-2.29924459872994	-4.02443592136898	h
-0.27671450593087	2.28797895124354	-4.16611572314229	h
0.54056660280652	-2.51423052211934	4.32239142772110	f
0.29915003340504	5.55463208204086	-0.10282654875626	c
-1.18504086706497	6.41158772812708	1.05354445766646	h
2.07903877929644	6.03586184195148	0.83617025727028	h
0.26278902407298	6.38240252072372	-1.98165097998674	h
0.10788130570677	-5.59843294818653	-0.05570390476907	c
1.86462406132211	-6.26514863493477	0.78962593308603	h

-1.44371313787360	-6.39435110892659	1.04289350483942	h
-0.00961979257213	-6.32641770251009	-1.97382579266801	h
-0.14722001936466	1.38864048359666	2.26326360387221	c
-2.21053728106597	1.52283171128329	2.68647341700824	h
1.15065812865460	2.49087956570899	4.18984102961362	f

$end
Etot = -509.5948665398 H
ZPE = 390.2 kJ/mol
enthalpy = 419.00 kJ/mol
chem. pot. = 294.86 kJ/mol

3H-3,6-dimethyl-1,2-difluorobenzeniumcation
$coord

0.41404601114066	-1.25340083346122	-2.42881424936683	c
0.49890564842869	-1.28893960603324	2.23177077184883	c
0.54076656398506	1.29614388085701	2.23002956849476	c
0.51646507989809	2.65051234063698	-0.05603053413846	c
0.46054255358741	1.30559184842676	-2.38542261743382	c
0.36860145086292	-2.26642459363446	-4.20542839982003	h
0.45662177072213	2.38036049118610	-4.12293685927236	h
0.53481222722543	-2.51901529789319	4.37022005367202	f
0.60495572864982	2.55247455953604	4.39849577941572	f
0.56197790591333	5.44711118370981	0.03615485155346	c
-1.02816064119215	6.14128899988403	1.16191318805402	h
2.25525531110144	6.08578997672993	1.03962292842154	h
0.51009218289474	6.28849151651278	-1.83434139613846	h
0.38756423108318	-2.79278898003161	-0.10299013153010	c
2.09768050196702	-3.98648322951711	-0.12221392142589	h
-1.90069261314406	-4.67644074799717	-0.05719519350627	c
-1.80780039989517	-5.88694607422137	-1.71480459364189	h
-1.79647997309291	-5.83884336880449	1.63278568679761	h
-3.67515354013567	-3.63848206588557	-0.07081493198381	h

$end
Etot = -509.6042352800 H
ZPE = 391.8 kJ/mol
enthalpy = 419.97 kJ/mol
chem. pot. = 299.32 kJ/mol

4H-3,6-dimethyl-1,2-difluorobenzeniumcation
$coord

0.00305681279574	-2.67680169187512	0.12785996178486	c
0.00534064719870	-1.28745719656942	2.30470383000505	c
0.00527216837370	1.36945205518103	2.23229058058046	c
-0.00150227340764	2.84202364702441	-0.01201887498072	c
-0.00376686151017	1.50574541678963	-2.20764284223196	c
-0.00800238372664	2.49081566827814	-4.00115790528990	h
0.00725994200625	-2.39653016478196	4.55231779153149	f
0.01105394868365	2.54603546624057	4.39335534134127	f
-0.00456552123889	5.67005271545397	0.17376097450607	c
-1.65523085487729	6.33155343277548	1.21602161771075	h
1.66321933451121	6.33680412613718	1.18520949137955	h
-0.02307881339820	6.51831679616759	-1.69741683911222	h
0.00056733844847	-5.48271436536967	0.17647165831583	c
1.62757395839227	-6.18029957437694	1.24164078560628	h
-1.67044791188386	-6.17397312136552	1.17709185442721	h
0.03345211349945	-6.28428306861197	-1.71476455511960	h
0.00106727918557	-1.27513898232407	-2.28445641436984	c

-1.60911428775533   -1.93074026190755   -3.43382943288008 h
1.61784536470305   -1.92286089686576   -3.42943702320453 h
$end
Etot = -509.6121599805 H
ZPE = 389.0 kJ/mol
enthalpy = 417.88 kJ/mol
chem. pot. = 295.46 kJ/mol

4,5-dimethyl-1,2-difluorobenzene
$coord
0.18841596309976   -2.60723350394525   1.82615611734692 c
0.09464066926585   -1.30447882885128   4.08997417081826 c
-0.09464066926585   1.30447882885128   4.08997417081826 c
-0.18841596309976   2.60723350394525   1.82615611734692 c
-0.09533906349129   1.32524624313681   -0.47303092126669 c
0.09533906349129   -1.32524624313681   -0.47303092126669 c
0.33559708040576   -4.64544313302462   1.89157992964459 h
-0.33559708040576   4.64544313302462   1.89157992964459 h
-0.18651066550281   2.56363470524457   6.29635968176699 f
0.18651066550281   -2.56363470524457   6.29635968176699 f
0.19694700992318   -2.77573099399623   -2.91889261267568 c
0.35870170827473   -4.79945825219663   -2.58342422009160 h
-1.49751371472733   -2.45507037481976   -4.05468296095588 h
1.80626180479413   -2.19362341240564   -4.07403918458686 h
-0.19694700992318   2.77573099399623   -2.91889261267568 c
-1.80626180479413   2.19362341240564   -4.07403918458686 h
-0.35870170827473   4.79945825219663   -2.58342422009160 h
1.49751371472733   2.45507037481976   -4.05468296095588 h
$end
Etot = -509.2977057541 H
ZPE = 362.1 kJ/mol
enthalpy = 388.90 kJ/mol
chem. pot. = 274.47 kJ/mol

1H-4,5-dimethyl-1,2-difluorobenzeniumcation
$coord
0.13906852509176   -2.77646579465707   1.61846477684862 c
-0.13161350427867   -1.52507650938347   3.84685798149976 c
-0.34787642510704   2.55878037214213   1.50021424554825 c
-0.12455132179417   1.31604896751876   -0.72604574838324 c
0.15222783941401   -1.40837739126663   -0.66655297751222 c
0.24803073603975   -4.81691236825850   1.61714991254594 h
-0.62951596264031   4.58388381193019   1.58609050738032 h
-0.30131899011198   -2.71520428575470   5.98476468893940 f
0.38886567377866   -2.81574692136424   -3.07193082604828 c
0.58455880772231   -4.83975639204070   -2.79925294970517 h
-1.25627073448102   -2.44362514059373   -4.27067016021187 h
2.01099757727346   -2.10834712935658   -4.14460193746897 h
-0.24035915628673   2.68781487682211   -3.20928877094593 c
-1.79920203665751   2.00217495067093   -4.37120270447693 h
-0.50008823132539   4.70538009530605   -2.92045632462434 h
1.49657446750783   2.41788217186075   -4.28777436806922 h
-0.05346738388656   1.28049602306463   3.97875672055095 c
1.94712800906797   1.66093277616412   4.51624024997446 h
-1.58318788932634   2.23611788719594   5.81923768415846 f
$end
Etot = -509.5934433373 H

ZPE = 391.0 kJ/mol
enthalpy = 418.83 kJ/mol
chem. pot. = 299.75 kJ/mol

3H-4,5-dimethyl-1,2-difluorobenzeniumcation
$coord
```
  -0.01761692968653   -1.24732079168898    4.21484005317652  c
  -0.05138528889985    1.33209569276894    4.15759924511888  c
  -0.04316103520729    2.55986961058126    1.81543959502332  c
  -0.00077980020549    1.32627900988115   -0.53655881833705  c
   0.03422127124432   -1.29111498469898   -0.55396020036130  c
  -0.07129145771003    4.60861552579690    1.87593369511102  h
  -0.09020911643477    2.67485921471863    6.26564023901146  f
  -0.02194668076573   -2.49640592777606    6.34128748742920  f
   0.08163825877471   -2.76855455749436   -2.94044407445973  c
   0.10617119967124   -4.79749878971575   -2.61717004980999  h
  -1.56100905750716   -2.30125818191920   -4.10587322266282  h
   1.73715991772526   -2.25520976240651   -4.06769878203642  h
   0.00941112901892    2.83471831006191   -2.94452876412829  c
  -1.61597015922783    2.35654081082021   -4.11999598978625  h
  -0.04736164899553    4.85477628132556   -2.57249214346943  h
   1.70260271353903    2.43796792736469   -4.05326122218241  h
   0.02179228629507   -2.70950590636648    1.86051042163657  c
  -1.59745532598856   -4.01942186460867    1.87157321714901  h
   1.65123842422526   -4.00413764904433    1.90856832920254  h
```
$end
Etot = -509.6037054236 H
ZPE = 389.0 kJ/mol
enthalpy = 417.52 kJ/mol
chem. pot. = 297.29 kJ/mol

4H-4,5-dimethyl-1,2-difluorobenzeniumcation
$coord
```
   0.61882771523737   -2.63497925991971    1.84202191127517  c
   0.55936198793413   -1.31495138430279    4.01848266871431  c
   0.41293350922414    1.36836648254698    3.90896841358429  c
   0.34728612750351    2.71899895185759    1.64821333567043  c
   0.39487857843217    1.44784111391275   -0.61365385913705  c
   0.73449971159353   -4.67672849659960    1.89910722458247  h
   0.26968413554292    4.75937000311817    1.75189564129156  h
   0.36097444748436    2.59065021787763    6.03588267824105  f
   0.63676173958635   -2.38734760768243    6.27297153185946  f
   0.31441226587565    2.83785412937044   -3.05295151865262  c
  -1.61579299557225    2.78488644835847   -3.80272074270026  h
   0.83938215349911    4.81200135609307   -2.82635026327477  h
   1.52422589992056    1.96133024111424   -4.47157943017834  h
   0.49881585203360   -1.36337214891947   -0.64369606356077  c
   2.26623098811127   -1.85387557014598   -1.63726817034510  h
  -1.66077685472144   -2.54577741178540   -2.29035020776634  c
  -1.57196123111971   -1.81606940451895   -4.20730222783737  h
  -1.43441930622360   -4.58718367403710   -2.35723934892823  h
  -3.49532472434174   -2.10101398633776   -1.47443157283772  h
```
$end
Etot = -509.6034046010 H
ZPE = 392.6 kJ/mol
enthalpy = 420.21 kJ/mol
chem. pot. = 301.46 kJ/mol

4,6-dimethyl-1,2-difluorobenzene

$coord

-0.74696846341009	-1.37748778745723	0.03494225878800	c
1.89034524791362	-1.41205274296897	0.02428342747748	c
3.18055572018358	0.88245332507759	-0.06101117421670	c
1.83552930311953	3.12261244205994	-0.12739778782336	c
-0.78234689169923	3.11704388533045	-0.11081924319562	c
-2.12979815775275	0.86445775495277	-0.03001486051235	c
-1.76850639712792	-3.15080996732312	0.09147721932752	h
5.22273776259859	0.96594757755656	-0.08169338186057	h
-4.97030337688391	0.91805839570835	-0.02621338553018	c
-5.69013899712513	1.99850454125874	1.57638446568932	h
-5.69762576285258	1.82279635670457	-1.73144757976275	h
-5.73771446465606	-0.98803686484659	0.07609234184994	h
3.31265621985571	-3.87541192168427	0.15773374028770	c
3.46582919522455	-4.53218402148666	2.11001298148520	h
2.35409647798805	-5.35165815147919	-0.91418521624847	h
5.22430364257796	-3.68483857972580	-0.58447866305938	h
-2.03044609833052	5.33699298085274	-0.18620061909739	f
3.06779504037656	5.34361277746994	-0.21746452359845	f

$end

Etot = -509.2989339377 H

ZPE = 361.2 kJ/mol

enthalpy = 389.13 kJ/mol

chem. pot. = 267.54 kJ/mol

1H-4,6-dimethyl-1,2-difluorobenzeniumcation

$coord

-2.40200870157581	-2.00758321851562	0.01897596621431	c
-0.06612282710180	-3.33127965772392	0.07954797642649	c
2.27282941097996	-2.03385599673890	0.00515856555133	c
2.25486206436806	0.51926273516718	-0.16933972296767	c
-2.52067648800651	0.55781295599601	-0.14736187433599	c
-4.12863504702888	-3.08433796741418	0.21964185703444	h
4.04508440856304	-3.03690377516571	0.16960434452860	h
-4.87666901933470	2.05548722178973	0.01030264372807	c
-4.75853712551014	3.34773132846573	1.62120047653873	h
-5.09631021255600	3.26053603219703	-1.65426744880206	h
-6.53261089855569	0.85803737086385	0.21113555584485	h
-0.08075181412068	-6.12745248173543	0.24339074849551	c
-1.69165597919695	-6.82187428580903	1.32250427768323	h
-0.29531689805640	-6.87527322775124	-1.68186951772051	h
1.67283598673077	-6.88483170192749	1.00352865109656	h
4.32568829651759	1.84428533119505	-0.12923780246549	f
-0.11622080701373	1.97890069745852	-0.62827553281969	c
-0.11016535703428	2.24583870154387	-2.71176858076484	h
-0.06355776361746	4.30351576919666	0.48662996818462	f

$end

Etot = -509.6023949982 H

ZPE = 390.0 kJ/mol

enthalpy = 418.49 kJ/mol

chem. pot. = 296.95 kJ/mol

2H-4,6-dimethyl-1,2-difluorobenzeniumcation

```
$coord
  -0.79777582895369   -1.45447708434534   -0.04151771236894  c
   1.89925535481513   -1.56384640256837    0.05130827231864  c
   3.17789155084452    0.66253553175700    0.12719025394501  c
  -0.96420195326157    2.97124601204044    0.10657787884271  c
  -2.28712867722398    0.73135518319998   -0.01421452938974  c
  -1.79546670899908   -3.24640486152370   -0.08087142712497  h
   5.21673875687477    0.77213853077986    0.25280428899155  h
  -5.12532988997281    0.69370292475717    0.01959170222881  c
  -5.84615180620442    1.63274817153407    1.70649144349201  h
  -5.88424806268582    1.69721928026301   -1.61296011946184  h
  -5.83522406730053   -1.23431840692206   -0.00802533365329  h
   3.18084340287052   -4.09487148426942    0.14250052352717  c
   2.47398882944108   -5.21001546279045    1.72622948093350  h
   2.80000247074900   -5.15879840997234   -1.58309596765489  h
   5.21691393961810   -3.90062748869430    0.34290120159208  h
  -2.12716833427183    5.12237143373954    0.29057957781272  f
   1.82279290078186    3.08769553113020   -0.18312982213305  c
   1.98234721046162    3.40951893103102   -2.27103426874563  h
   2.89192091241707    5.08282807085377    1.02867455684815  f
$end
```

Etot = -509.5863677620 H
ZPE = 390.0 kJ/mol
enthalpy = 418.78 kJ/mol
chem. pot. = 296.61 kJ/mol

3H-4,6-dimethyl-1,2-difluorobenzeniumcation

```
$coord
  -0.77233067268395   -1.44782815135509   -0.01141808488795  c
   1.80780783553185   -1.52054056167341    0.05349611431670  c
   1.73530333022466    3.22443044674050   -0.01366161046435  c
  -0.83521787743869    3.18010076678216   -0.07324011012520  c
  -2.13689957163965    0.85032713292954   -0.07368316284200  c
  -1.83862572329177   -3.19146437330921   -0.01305968330989  h
  -4.93649039591743    0.89718337681185   -0.13993813201212  c
  -5.65808459605212    1.97947215570451    1.46811739825778  h
  -5.58024614688255    1.92258729011998   -1.81709208895644  h
  -5.74769880106301   -0.98722227448504   -0.12721120445573  h
   3.26471466864741   -3.92129333935419    0.14047881571202  c
   4.36186405461875   -4.02728731984131    1.88971214356457  h
   2.03955402970817   -5.56681752680265    0.03628604677149  h
   4.62895540346442   -4.00480012672293   -1.40830670920420  h
  -2.14704951741089    5.31612198332096   -0.13176419459371  f
   2.96980300544199    5.36271571148695   -0.01393112805679  f
   3.24653755807933    0.89217304832067    0.04770902236379  c
   4.51197116247058    0.96787205600541    1.69460947297895  h
   4.55875329714458    0.92163894708201   -1.56457244362795  h
$end
```

Etot = -509.6140158644 H
ZPE = 388.8 kJ/mol
enthalpy = 417.76 kJ/mol
chem. pot. = 295.22 kJ/mol

4H-4,6-dimethyl-1,2-difluorobenzeniumcation

$coord

-0.85367849341151	-1.45946729570079	-0.34687472714158	c
3.21924984581247	0.90768877195057	-0.48083436344883	c
1.87391926396760	3.07345341175336	-0.65566496945078	c
-0.80585157435808	2.97256814027428	-0.66352215740459	c
-2.23024655894931	0.71823832009474	-0.52515194364225	c
-1.83193137221924	-3.25430913308357	-0.22796903873117	h
5.26390036761671	0.97534207463929	-0.46331660470078	h
-5.06256585073414	0.84419284237845	-0.57184410711914	c
-5.76867212508257	1.97966535869988	0.99688967461076	h
-5.72384946328355	1.72901180897796	-2.31231471551214	h
-5.87461428090963	-1.03840330922332	-0.44073731432046	h
-2.00206145174006	5.11126767087281	-0.81723775480769	f
2.94700527963305	5.32365076182865	-0.81023607829514	f
1.92418984440160	-1.54360889839838	-0.29024364724986	c
2.49589517167737	-2.64173835364099	-1.97756094358689	h
2.92827865017775	-3.14032193277408	1.99752183094756	c
2.07852429526489	-5.01128439547219	1.96048839134500	h
4.96968953566861	-3.33352816078033	1.85962021589868	h
2.45281891646819	-2.21241768239637	3.76898825260933	h

$end

Etot = -509.5994643977 H

ZPE = 392.4 kJ/mol

enthalpy = 420.19 kJ/mol

chem. pot. = 300.96 kJ/mol

5H-4,6-dimethyl-1,2-difluorobenzeniumcation

$coord

-0.01339993069273	-3.39859576293075	0.02110543824346	c
2.22279041771696	-2.11899155514134	-0.11880598579102	c
2.18895308182665	0.52672913150157	-0.22311938007462	c
-0.04350264394592	1.99261846976352	-0.20779652008430	c
-2.34321209703035	0.85211784185275	-0.06178841732902	c
4.03555139914648	-3.06188166365727	-0.15952158103056	h
-4.74388779627401	2.30868780679685	-0.09186805546121	c
-4.44908574711867	4.28344681079012	0.39049976184357	h
-5.55428127629395	2.23864394820236	-1.99584969512112	h
-6.13308935592361	1.47861280201039	1.18341476991328	h
-0.14388232519324	-6.20407625384682	0.09349917794660	c
-1.32587576770404	-6.84748372958683	1.65875449527166	h
-1.03321267225826	-6.90507195352824	-1.63731311578886	h
1.71579462783733	-7.06070881757340	0.25923951640338	h
0.20491928555272	4.48329168172811	-0.34831007227098	f
4.32911485355117	1.72788517958801	-0.35106400493216	f
-2.41730132110663	-1.94938134611141	0.10811233795928	c
-3.39064087072233	-2.44883181437359	1.87844838275497	h
-3.71920721289700	-2.68290573116062	-1.33368873762357	h

$end

Etot = -509.6159401905 H

ZPE = 388.7 kJ/mol

enthalpy = 418.06 kJ/mol

chem. pot. = 292.82 kJ/mol

6H-4,6-dimethyl-1,2-difluorobenzeniumcation
$coord
```
  -0.77378336700908    -1.51723745367191    -0.40963074300316  c
   1.80679362884247    -1.54571272170493    -0.43266970044829  c
   3.05701874757918     0.82675598502650    -0.51013837945969  c
   1.80119492111429     3.13144539944103    -0.57867106081489  c
  -0.79986313538833     3.16191469237295    -0.55908505655957  c
  -1.82755365875327    -3.27041295333278    -0.34922361574045  h
   5.10657443638676     0.89405588545827    -0.52747694176798  h
   3.34301481491508    -3.93237224343605    -0.37620676490121  c
   4.53520503867547    -3.98760071649899     1.30557928953030  h
   2.13954613861502    -5.59848586315751    -0.36884007051165  h
   4.58477350571952    -4.03723947846745    -2.01874094057130  h
  -2.01599649600257     5.29974556000532    -0.63402529043809  f
   3.09119212304605     5.26981506546012    -0.65944718877103  f
  -2.28290837584472     0.82530488529232    -0.43655373411638  c
  -3.44318409008955     0.79019459414510    -2.17348334683170  h
  -4.23775156288558     0.91174092221837     1.79967187856658  c
  -5.38436165740315     2.60804894473236     1.64151098550383  h
  -5.45306880066642    -0.74047186678565     1.68713150083545  h
  -3.24684221085100     0.91051136290285     3.60029917949931  h
```
$end
Etot = -509.5947172890 H
ZPE = 391.9 kJ/mol
enthalpy = 420.02 kJ/mol
chem. pot. = 299.84 kJ/mol

5,6-dimethyl-1,2-difluorobenzene
$coord
```
   0.28490064962471    -3.22318358201266     0.12121888879871  c
   2.91201984279286    -3.25589088692310     0.01777924870117  c
   4.18629834583403    -0.98827692229630    -0.12721707068083  c
   2.85783714884925     1.27519163492152    -0.16858186667801  c
   0.23661685012814     1.34349499789891    -0.06792514091392  c
  -1.07066883033753    -0.96799420355837     0.08150456640637  c
  -0.72512397632615    -4.99763497832031     0.23596042751491  h
   3.97461609278647    -4.99997960578635     0.04844648820874  h
   4.19045551561620     3.44272170621382    -0.31010719299891  f
   6.72587412179970    -0.93395234322237    -0.22918278244097  f
  -1.13522264793639     3.83277473478561    -0.11227608087165  c
  -2.22222225967263     4.10260572134957     1.62169436447399  h
   0.16581509740679     5.40960243707097    -0.30349290323643  h
  -2.47435286782760     3.90731006828227    -1.68017295615881  h
  -3.91341157750737    -0.98860421728363     0.19608546375753  c
  -4.61361856314665     0.07229606942524     1.82316142113671  h
  -4.74451209484144    -0.11996226427917    -1.48292041952233  h
  -4.63530084724250    -2.91051836626561     0.33602554450382  h
```
$end
Etot = -509.2983907361 H
ZPE = 362.5 kJ/mol
enthalpy = 389.40 kJ/mol
chem. pot. = 273.08 kJ/mol

1H-5,6-dimethyl-1,2-difluorobenzeniumcation

$coord

0.16024984117737	-3.22683369475136	0.00938892500487	c
2.80937006659627	-3.36620881410734	0.10238251191120	c
4.11354174246583	-1.15525153209259	0.13341537439899	c
-0.01034571786898	1.29960940908821	0.00728725839208	c
-1.27835244313900	-0.98322414018007	-0.02581856435475	c
-0.86173509864295	-5.00230876103444	0.02921554346170	h
3.78354687962786	-5.15633413604733	0.23460822300010	h
6.55894621073125	-1.05781368497635	0.33021705084830	f
-1.30014230335160	3.77907200207108	0.15120728401814	c
-2.31594105266314	3.90865726524467	1.95141083914460	h
0.00094353130724	5.36006005047821	0.02758147294131	h
-2.74169420873354	3.93763216761311	-1.32075234156793	h
-4.11774000209755	-1.09251381336372	0.03628991178286	c
-4.85686366801441	-0.12730465773029	1.70196853829723	h
-4.92695046483417	-0.16317285201489	-1.61761251488666	h
-4.79687737698944	-3.03183188676763	0.07291473199924	h
2.79972687572988	1.29478776532653	-0.28909183043513	c
2.99198915416689	1.51213929525263	-2.38189149014265	h
3.98832803453214	3.27084001799155	0.84727907618660	f

$end

Etot = -509.5916875514 H

ZPE = 390.3 kJ/mol

enthalpy = 418.61 kJ/mol

chem. pot. = 297.89 kJ/mol

2H-5,6-dimethyl-1,2-difluorobenzeniumcation

$coord

0.06778015142802	-3.24218073066992	0.21455573190340	c
2.61285754107932	-3.31073686955015	0.26245266946746	c
2.56125298637862	1.40558546205895	0.02959362466235	c
-0.02320643667417	1.47625685733540	-0.03510408812369	c
-1.28071586233413	-0.89679980709549	0.04850691327506	c
-1.01437336173343	-4.96748607803914	0.37701825747263	h
3.67922992426945	-5.04308941859428	0.47339239202593	h
3.89605380744353	3.47408605507797	0.08154572311320	f
-1.43607335823597	3.93801594339217	-0.08423567344510	c
-2.59411925538877	4.15133451549582	1.60809457971750	h
-0.15164495657758	5.53534680146571	-0.18412348579415	h
-2.69464860172223	4.01936059791629	-1.71453651375440	h
-4.07878186704582	-0.95665439250382	0.03059173211590	c
-4.82831101527098	0.17907676364272	1.58896622828670	h
-4.79023987052710	-0.05253198570769	-1.69081253137922	h
-4.83416064381822	-2.86035356813862	0.15146171717509	h
4.07636532796978	-0.96286424795047	-0.15612859301136	c
4.56957538611588	-1.04188101111518	-2.20216532335321	h
6.26316010464375	-0.84448488702042	1.20092663964605	f

$end

Etot = -509.5940764717 H

ZPE = 391.0 kJ/mol

enthalpy = 419.11 kJ/mol

chem. pot. = 299.17 kJ/mol

3H-5,6-dimethyl-1,2-difluorobenzeniumcation

$coord

0.18531356863456	-3.19482517571679	0.12687133382409	c
4.24107615720016	-0.86674004912507	-0.13709945436504	c
2.91004535866151	1.34945089761744	-0.17734022868129	c
0.25292740658741	1.34968684464433	-0.07033533679566	c
-1.13072521102184	-0.98826024096026	0.08603036361762	c
-0.80161566320223	-4.98243953151011	0.24375907199373	h
4.15596103080750	3.52066551893173	-0.31929118714735	f
6.70232299150910	-0.85400210684644	-0.23682101902187	f
-1.10138474017828	3.80140517417385	-0.11631387838767	c
-2.23636884584721	3.99203133584488	1.60291664849517	h
0.15760840743051	5.40980331285214	-0.27781358824333	h
-2.45320895092295	3.82229385935054	-1.68019608927322	h
-3.96726638472061	-0.94434339386521	0.19878063635781	c
-4.63251011655205	0.12440625485164	1.83178140624808	h
-4.76561999350998	-0.05629375839541	-1.48241461670972	h
-4.72503335508440	-2.84964461074757	0.33261703780969	h
2.96451609293722	-3.31174783466738	0.01890391314158	c
3.70431197790099	-4.35341187012844	1.66351166263842	h
3.57277706309000	-4.49252806578805	-1.58506755167089	h

$end

Etot = -509.6075160266 H

ZPE = 389.9 kJ/mol

enthalpy = 417.98 kJ/mol

chem. pot. = 298.67 kJ/mol

4H-5,6-dimethyl-1,2-difluorobenzeniumcation

$coord

3.05761404434735	-3.26144798329091	0.01040407881391	c
4.25552573592881	-1.02292698456363	-0.13137541579682	c
2.81917152333504	1.25908500396018	-0.16731104192017	c
0.17491997087484	1.39159141426436	-0.06645361135047	c
-1.10585841540666	-0.88580867766266	0.08217808352433	c
4.11546650139732	-5.01052762354543	0.03971520192754	h
4.12026903436968	3.34420289161647	-0.30518374671652	f
6.74166022749741	-0.81114950340227	-0.24151818185373	f
-1.15023168909117	3.90260080202741	-0.11692859973857	c
-2.25061075161005	4.16939572668415	1.60626403653704	h
0.17065172461028	5.46364409018263	-0.28140753251115	h
-2.45837193895699	3.99431769264081	-1.70731541340661	h
-3.91030759489168	-0.96948382480045	0.19981158863301	c
-4.59469220272558	0.11530985735757	1.82076114618908	h
-4.72420804771049	-0.06377768416729	-1.47105885865156	h
-4.63415997176282	-2.88777403113855	0.33096091385715	h
0.28156882487818	-3.30990382714254	0.12741989373857	c
-0.45277199482220	-4.51384363159006	-1.40663410286418	h
-0.31801824466138	-4.37598441931868	1.81449682319281	h

$end

Etot = -509.6097623025 H

ZPE = 389.7 kJ/mol

enthalpy = 417.98 kJ/mol

chem. pot. = 297.93 kJ/mol

5H-5,6-dimethyl-1,2-difluorobenzeniumcation

$coord

0.26364299345650	-3.28015613008716	-0.33301449989000	c
2.81904249264738	-3.25011721276124	-0.45137320569431	c
4.02349653397239	-0.88034690885910	-0.60794694621924	c
2.70246618407245	1.42656923799093	-0.66290125407664	c
0.12020923295533	1.48004052741464	-0.56213875638740	c
-0.74043036697400	-5.05876226889104	-0.21141300933445	h
3.97089398875164	-4.93730427690952	-0.43840590963794	h
4.05300767163308	3.53190316260844	-0.83284816899243	f
6.47369290670197	-0.80178255678166	-0.72681842537010	f
-1.27224129899632	3.91655806029945	-0.64439548375604	c
-1.57459582608137	4.59442731685237	1.28990997124264	h
-0.19715033155182	5.36639880878129	-1.62850928421522	h
-3.12295708692373	3.70035925269402	-1.51699115746797	h
-1.27218272946806	-0.95082879397891	-0.32964101451683	c
-2.52260107205341	-1.07076637814105	-1.99495248515521	h
-3.10341238426792	-0.94354682663308	2.00297515967081	c
-4.39162641468918	0.65062300943247	1.88215357221251	h
-4.20538177501341	-2.67827985447984	2.01785266984260	h
-2.02387271817156	-0.81498816855114	3.74845822774517	h

$end

Etot = -509.6042997848 H

ZPE = 392.8 kJ/mol

enthalpy = 420.44 kJ/mol

chem. pot. = 301.54 kJ/mol

6H-5,6-dimethyl-1,2-difluorobenzeniumcation

$coord

0.25583918640608	-3.30133037355336	0.14971035065143	c
2.90051214649709	-3.25730347346576	0.06236044326189	c
4.28139859927375	-1.01706345443318	-0.12272136332645	c
2.99727708625370	1.22601386343081	-0.21021274364813	c
-1.12014335647294	-1.10065746964058	0.08579655773223	c
-0.69309285315009	-5.10780246992698	0.25178496457273	h
3.96145134458237	-5.00943870836242	0.11440334960399	h
4.21993268064679	3.35747941103093	-0.42846431846781	f
6.77763124333109	-1.07869250343357	-0.23659842884922	f
-3.92718331245785	-1.10409148704452	0.18261102071702	c
-4.55437142885468	-0.70467364361482	2.11584085331472	h
-4.74098859965515	0.35744286856467	-1.02031969413800	h
-4.69699605497644	-2.93457736510599	-0.34882507487148	h
0.22103134659936	1.37223636821396	-0.02385140925690	c
-0.43172987022676	2.36826899557861	-1.73026150383017	h
-0.52064532467514	3.12344147266082	2.26288209742813	c
-2.54145376485786	3.47872128731418	2.21926247818276	h
-0.01945836089029	2.21401600480616	4.03753833001087	h
0.47638630814089	4.91101390719980	2.10083764941678	h

$end

Etot = -509.5998223952 H

ZPE = 392.7 kJ/mol

enthalpy = 420.47 kJ/mol

chem. pot. = 301.24 kJ/mol

CH_2Cl_2

$coord
0.00000000000000 0.00000000000000 -0.28350808651116 c
0.00000000000000 -1.69495000015265 -1.43256677274059 h
0.00000000000000 1.69495000015265 -1.43256677274059 h
-2.81447994362993 0.00000000000000 1.57432081599618 cl
2.81447994362993 0.00000000000000 1.57432081599618 cl
$end
Etot = -959.6196464043 H
ZPE = 75.78 kJ/mol
enthalpy = 87.74 kJ/mol
chem. pot. = 6.85 kJ/mol

$[MeCH_2Cl_2]^+$

$coord
-1.82617019480323 0.85527252243172 -1.96769582737317 c
-1.99061835257700 -1.11475766189788 -2.51143791864168 h
-1.84263528975484 2.17055430763157 -3.54366192995546 h
-3.83468977003716 1.69775111419997 0.43292694769967 cl
1.67735713602074 1.12339087245745 -0.97426989331940 cl
1.90491836830991 -0.94837474833839 1.80646346040738 c
1.60463776938902 -2.86527373940098 1.14639058283333 h
3.81890369111291 -0.63164878007545 2.47834824726191 h
0.48829664233965 -0.28691388700803 3.13293633108746 h
$end
Etot = -999.1929682185 H
ZPE = 173.8 kJ/mol
enthalpy = 192.22 kJ/mol
chem. pot. = 93.61 kJ/mol

$[HCH_2Cl_2]^+$

$coord
-0.85633958300171 0.95968521890862 -0.12440301612330 c
-0.39905259055511 1.57985605945970 1.77309057068251 h
-0.46908920386836 2.24196691364704 -1.67593485106395 h
-3.36954290193866 -0.90124573783698 -0.43825333548955 cl
2.43251777697246 -2.72150619830662 1.16911129400408 h
2.66150650239143 -1.15875625587169 -0.70361066200979 cl
$end
Etot = -959.8725970944 H
ZPE = 93.41 kJ/mol
enthalpy = 108.91 kJ/mol
chem. pot. = 18.33 kJ/mol

$CH_2ClOTeF_5$

$coord
0.33519267491892 -2.88454683766945 -1.47125123283604 c
1.87908489606404 -2.49001535594465 -2.76483428251886 h
-1.49208203577579 -2.49499543381484 -2.32373066959910 h
0.66633177644307 -1.51234235887580 0.83519702657854 o
-0.15790229159999 1.97314924960077 1.03316798877056 te
-3.39332807586879 1.40740650114992 -0.24868637851367 f
0.98310303110695 2.56415859942374 -2.25483595722006 f
-0.95872453942594 5.36661734925822 1.28167134855268 f
-1.32759920257812 1.50600428439727 4.29626113848393 f
3.02950364937042 2.70430645937170 2.28820086069889 f
0.43642011734522 -6.13974245689676 -0.67115984239689 cl

$end
Etot = -1341.985524008 H
ZPE = 118.6 kJ/mol
enthalpy = 149.78 kJ/mol
chem. pot. = 14.85 kJ/mol

$[MeClCH_2OTeF_5]^+$
$coord
1.96018985111497	-0.96507830292168	-2.31698887969489	c
3.50817145253533	-2.28978732423588	-2.55291132901901	h
1.06875713328374	-0.17906082942305	-3.99085850700094	h
2.08708523945759	0.34318612600174	-0.25426151537656	o
0.04994180504140	3.47865163884697	0.44409548660994	te
-2.81087564667363	1.69066558396666	-0.50246452125975	f
0.49002033624923	4.40082238000026	-2.89580599263498	f
-1.79617419769646	6.30279680406606	1.15357430792111	f
-0.28865567271157	2.22650623047676	3.67083394025163	f
3.04539065241402	4.99081823445437	1.30187026108317	f
-0.91913420025016	-3.98228017898132	-1.87249514960855	cl
-1.52712428631493	-3.99426309381945	1.52372489545779	c
-3.29949622730110	-3.00954500656821	1.80737753166866	h
-1.61880427884898	-5.97141910155911	2.05716298234000	h
0.05070803970033	-3.04201316030414	2.42714648926230	h

$end
Etot = -1381.565613376 H
ZPE = 216.3 kJ/mol
enthalpy = 254.65 kJ/mol
chem. pot. = 103.90 kJ/mol

$[HClCH_2OTeF_5]^+$
$coord
1.40926930157772	-1.76207548886829	-1.71241422109744	c
2.82584289466447	-3.22385495235496	-1.99524794249481	h
-0.04500229513135	-1.43654838464310	-3.12977138989995	h
1.78721682394333	-0.26410883813728	0.08208887860677	o
-0.46411738880873	2.82657244178329	0.92261985137181	te
-3.20682404491320	0.74227324581586	0.36552625539294	f
-0.42963672571349	3.53072230572151	-2.49323729955186	f
-2.41909025807285	5.54948268029321	1.69392464783316	f
-0.28278589334589	1.69776033922539	4.19042000200380	f
2.52366923465362	4.52793749494885	1.30657572639197	f
-1.63280838709701	-5.29467492488835	-0.09519251204267	cl
-0.06573326175659	-6.89348591889616	0.86470800348640	h

$end
Etot = -1342.256191841 H
ZPE = 135.1 kJ/mol
enthalpy = 170.99 kJ/mol
chem. pot. = 25.77 kJ/mol

NC_5F_5
$coord
-2.13175680248628	0.00000000000000	-1.76609117374860	c
-2.26829141955860	0.00000000000000	0.85422925380889	c
0.00000000000000	0.00000000000000	2.18061115621325	c
2.26829141955860	0.00000000000000	0.85422925380889	c
2.13175680248628	0.00000000000000	-1.76609117374860	c
0.00000000000000	0.00000000000000	-3.02082163234920	n

4.26777532873910 0.00000000000000 -3.08847859869599 f
-4.26777532873910 0.00000000000000 -3.08847859869599 f
-4.46592902999212 0.00000000000000 2.07876752527249 f
0.00000000000000 0.00000000000000 4.68335646286239 f
4.46592902999212 0.00000000000000 2.07876752527249 f
$end
Etot = -744.4652517208 H
ZPE = 125.9 kJ/mol
enthalpy = 150.43 kJ/mol
chem. pot. = 38.86 kJ/mol

$[MeNC_5F_5]^+$
$coord
-0.06297542152629 -2.18170125414344 0.00000000000000 c
2.54419046047328 -2.27402953117702 0.00000000000000 c
3.88696280810764 -0.00138623161991 0.00000000000000 c
2.56969619410052 2.28338683593726 0.00000000000000 c
-0.04276098122677 2.23276738035604 0.00000000000000 c
-1.42330742330835 -4.23481154178539 0.00000000000000 f
3.71891819704425 -4.45635783254555 0.00000000000000 f
6.34583873457141 -0.01495828324914 0.00000000000000 f
3.77117796298255 4.45175820689612 0.00000000000000 f
-1.34370900710712 4.32356064359047 0.00000000000000 f
-4.16859662646559 -0.02574197283846 0.00000000000000 c
-4.86195009198710 1.89831905509271 0.00000000000000 h
-4.79524106205960 -1.01648687840107 -1.68553214861077 h
-4.79524106205960 -1.01648687840107 1.68553214861077 h
-1.34300268153922 0.03216828228849 0.00000000000000 n
$end
Etot = -784.0836887539 H
ZPE = 232.3 kJ/mol
enthalpy = 262.11 kJ/mol
chem. pot. = 135.84 kJ/mol

$[HNC_5F_5]^+$
$coord
-2.29919131249498 0.00000000000000 -1.27674196880174 c
0.00000000000000 0.00000000000000 -2.58961494934371 c
2.29919131249498 0.00000000000000 -1.27674196880174 c
2.23518308047427 0.00000000000000 1.33113270273271 c
-2.23518308047427 0.00000000000000 1.33113270273271 c
0.00000000000000 0.00000000000000 4.48452213106983 h
4.25289924990766 0.00000000000000 2.72652794998835 f
4.45805582862935 0.00000000000000 -2.48586343826002 f
0.00000000000000 0.00000000000000 -5.04402507243016 f
-4.45805582862935 0.00000000000000 -2.48586343826002 f
-4.25289924990766 0.00000000000000 2.72652794998835 f
0.00000000000000 0.00000000000000 2.55900739938550 n
$end
Etot = -744.7763604628 H
ZPE = 160.6 kJ/mol
enthalpy = 185.62 kJ/mol
chem. pot. = 73.29 kJ/mol

NC_5F_4I

```
$coord
   0.00000000000000     0.00000000000000    -2.09313612482296  c
   2.24842271768006     0.00000000000000    -0.73019337137702  c
   2.12575756516087     0.00000000000000     1.89318284731361  c
  -2.12575756516087     0.00000000000000     1.89318284731361  c
  -2.24842271768006     0.00000000000000    -0.73019337137702  c
  -4.26706387205152     0.00000000000000     3.21150995630949  f
  -4.48646458897778     0.00000000000000    -1.88725334624425  f
   4.48646458897778     0.00000000000000    -1.88725334624425  f
   4.26706387205152     0.00000000000000     3.21150995630949  f
   0.00000000000000     0.00000000000000     3.15608010925613  n
   0.00000000000000     0.00000000000000    -6.03743615643678  i
$end
```

Etot = -942.3305763184 H
ZPE = 119.0 kJ/mol
enthalpy = 145.49 kJ/mol
chem. pot. = 24.85 kJ/mol

$[MeNC_5F_4I]^+$

```
$coord
   3.84309722846643     0.00058943999955     0.00000000000000  c
   2.45188909773188    -2.24537682800510     0.00000000000000  c
  -0.15406131639222    -2.17451310967299     0.00000000000000  c
  -0.13584486672565     2.22701663023108     0.00000000000000  c
   2.47533428443425     2.25824054651286     0.00000000000000  c
  -1.44039950543165     4.32232548491605     0.00000000000000  f
   3.61202900198368     4.47302413802316     0.00000000000000  f
   3.56332237891046    -4.47226647751947     0.00000000000000  f
  -1.51642002775640    -4.23352682988385     0.00000000000000  f
   7.75009488583997    -0.02004639403002     0.00000000000000  i
  -4.26234319151568    -0.02662438855754     0.00000000000000  c
  -4.89207227684617    -1.01871947035875    -1.68392379618668  h
  -4.89207227684617    -1.01871947035875     1.68392379618668  h
  -4.95858456245618     1.89630352813081     0.00000000000000  h
  -1.44396885339651     0.03229320057307     0.00000000000000  n
$end
```

Etot = -981.9577117529 H
ZPE = 224.8 kJ/mol
enthalpy = 256.67 kJ/mol
chem. pot. = 121.42 kJ/mol

$[HNC_5F_4I]^+$

```
$coord
   0.00000000000000     0.00000000000000    -2.52409343019722  c
   2.27133951170660     0.00000000000000    -1.15977499090742  c
   2.22741070047243     0.00000000000000     1.44643461994788  c
  -2.22741070047243     0.00000000000000     1.44643461994788  c
  -2.27133951170660     0.00000000000000    -1.15977499090742  c
   0.00000000000000     0.00000000000000     4.60906602941398  h
   0.00000000000000     0.00000000000000     2.68567162442685  n
  -4.47921403934048     0.00000000000000    -2.30178374052736  f
  -4.25232433917254     0.00000000000000     2.84330649595364  f
   4.25232433917254     0.00000000000000     2.84330649595364  f
   4.47921403934048     0.00000000000000    -2.30178374052736  f
   0.00000000000000     0.00000000000000    -6.42700899257716  i
$end
```

Etot = -942.6512551506 H
ZPE = 153.1 kJ/mol
enthalpy = 180.06 kJ/mol
chem. pot. = 58.70 kJ/mol

[$N_3C_3F_3$]
$coord
```
  -1.19604163577979    2.07160488113841    0.00000000000000  c
  -1.19604163577979   -2.07160488113841    0.00000000000000  c
   2.39208327155962    0.00000000000000    0.00000000000000  c
  -2.43613096161762   -4.21950259941334    0.00000000000000  f
  -2.43613096161762    4.21950259941334    0.00000000000000  f
   4.87226192323524    0.00000000000000    0.00000000000000  f
   1.29178260036823   -2.23743309617122    0.00000000000000  n
   1.29178260036823    2.23743309617122    0.00000000000000  n
  -2.58356520073648    0.00000000000000    0.00000000000000  n
```
$end
Etot = -578.1027710451 H
ZPE = 108.7 kJ/mol
enthalpy = 127.22 kJ/mol
chem. pot. = 31.34 kJ/mol

[$MeN_3C_3F_3$]$^+$
$coord
```
   0.65153035098513    2.18790841204294    0.00000000000000  c
   0.62775424070340   -2.14101241369815    0.00000000000000  c
   4.21308130951005   -0.00474739597299    0.00000000000000  c
  -0.68749021098619   -4.19299064101403    0.00000000000000  f
  -0.60610000956939    4.27508673161593    0.00000000000000  f
   6.63764855196507   -0.02094300915328    0.00000000000000  f
   3.06694957666461   -2.23980390163782    0.00000000000000  n
   3.09551224719108    2.24115867616036    0.00000000000000  n
  -3.57465730995438   -0.02199579164784    0.00000000000000  c
  -4.26185132316029    1.90570502411633    0.00000000000000  h
  -4.20419195009216   -1.01028516474308   -1.68664552423017  h
  -4.20419195009216   -1.01028516474308    1.68664552423017  h
  -0.75399352316471    0.03220463867461    0.00000000000000  n
```
$end
Etot = -617.7139873235 H
ZPE = 214.1 kJ/mol
enthalpy = 238.05 kJ/mol
chem. pot. = 123.51 kJ/mol

[$HN_3C_3F_3$]$^+$
$coord
```
   0.00000000000000    0.00000000000000    2.84809479631941  c
   2.18806059877226    0.00000000000000   -0.71468490951850  c
  -2.18806059877226    0.00000000000000   -0.71468490951850  c
   0.00000000000000    0.00000000000000   -3.98694327241193  h
   4.22144081835305    0.00000000000000   -2.04082493188351  f
   0.00000000000000    0.00000000000000    5.26598267278582  f
  -4.22144081835305    0.00000000000000   -2.04082493188351  f
   0.00000000000000    0.00000000000000   -2.06178045829594  n
  -2.25296634313502    0.00000000000000    1.72283297220332  n
   2.25296634313502    0.00000000000000    1.72283297220332  n
```
$end
Etot = -578.4047107374 H

ZPE = 142.4 kJ/mol
enthalpy = 161.50 kJ/mol
chem. pot. = 61.82 kJ/mol

[MeClH]⁺

```
$coord
  -0.56543589300053   -0.36595011229166    0.84366837533605  c
   0.00902878109819   -2.31821496407070    0.60793658359676  h
   0.49975301972127    0.70282796314041    2.22936512447792  h
  -2.59703792136928   -0.12147386391676    1.00418239174122  h
   0.10715085437041    1.20430991727270   -2.28547316478282  cl
   2.54654115917995    0.89850105986599   -2.39967931036916  h
$end
```

Etot = -500.3181038919 H
ZPE = 119.3 kJ/mol
enthalpy = 131.74 kJ/mol
chem. pot. = 54.77 kJ/mol

$[CH_2Cl]^+$

```
$coord
   0.00000000000000    0.00000000000000   -0.24865678245851  c
   1.79435207712726    0.00000000000000   -1.25843652536337  h
  -1.79435207712726    0.00000000000000   -1.25843652536337  h
   0.00000000000000    0.00000000000000    2.76552983318522  cl
$end
```

Etot = -499.0776514221 H
ZPE = 65.24 kJ/mol
enthalpy = 75.39 kJ/mol
chem. pot. = 6.21 kJ/mol

$[CH_2OTeF_5]^+$

```
$coord
  -2.93838527963546   -1.62488838468461   -1.42155449697117  c
  -2.30843900110580   -3.33653502236333   -0.45005207899562  h
  -4.66098325498029   -1.63038952016151   -2.56165608838860  h
  -1.76551698006863    0.38580080250463   -1.29237563887305  o
   1.63697425258115    0.99670558815141    0.76153018728010  te
   2.83708984502832   -1.80347045933679   -0.91927953350751  f
   0.27161530418833   -1.14405583738148    3.14769761938237  f
   4.53774488968276    1.55178986231382    2.50820484479678  f
   2.49049060932415    2.97166859507379   -1.94182946469044  f
  -0.10059038501457    3.63337437588409    2.16931464996713  f
$end
```

Etot = -881.4713170005 H
ZPE = 111.3 kJ/mol
enthalpy = 139.74 kJ/mol
chem. pot. = 15.87 kJ/mol

$[Me_2Cl]^+$ with COSMO (ε_R DCM)

```
$coord
  -1.98405009229510   -0.02098963202409    1.83432600895542  c
  -2.26206448398021   -1.97841915296400    1.29995197966391  h
  -0.51419326178527    0.26049715452948    3.23248347308744  h
  -3.73601684120484    0.92648095396826    2.32071763536219  h
  -0.93210416562440    1.62263899880372   -1.02135660568787  cl
   2.00794224185052   -0.02067827978101   -1.80809485426875  c
   2.66316059735374    0.94035457441494   -3.49659912813942  h
```

3.25878431744139 0.24427253642967 -0.20820464147197 h
1.49854168824412 -1.97415715337698 -2.15322386750094 h
$end
E(COSMO) = -539.7221153427 H
ZPE = 200.5 kJ/mol
enthalpy = 215.54 kJ/mol
chem. pot. = 129.91 kJ/mol

$[MeCl\cdots CH_2Cl]^+$ with COSMO (ε_R DCM)
$coord
-1.74581571389478 0.83827298606909 -1.93478644464126 c
-1.89950779852708 -1.15216934744983 -2.38933623648056 h
-1.82444251466985 2.07709179754972 -3.56542836078661 h
-3.84301373831243 1.73799074760944 0.42009043307158 cl
1.62319869850949 1.23407424029674 -0.90801806056359 cl
1.87197466651857 -0.93933529955150 1.77660982244547 c
1.51812795200163 -2.81525530953295 1.03564168233934 h
3.80412684008161 -0.67839020294229 2.41182797033054 h
0.49535160829285 -0.30227961204843 3.15339919428518 h
$end
E(COSMO) = -999.2732013724 H
ZPE = 177.2 kJ/mol
enthalpy = 194.43 kJ/mol
chem. pot. = 99.44 kJ/mol

$[MeCl\cdots CH_2OTeF_5]^+$ with COSMO (ε_R DCM)
$coord
1.41003836726465 -1.01985872400031 -1.88989615068370 c
2.97596161872049 -2.34323091576330 -1.84484552669293 h
1.01370527271234 -0.20626508449059 -3.73264353675659 h
1.50121972737910 0.53962585913106 0.11750158846426 o
-0.12808032984581 3.83880294266345 0.21487893037597 te
-3.02013492865032 2.50358589970600 -1.25704954360283 f
1.09704457684645 4.57479482037963 -2.98429295394972 f
-1.63419109468748 6.96430603176835 0.39483184397900 f
-1.35064544103051 3.01337795117142 3.37701145743994 f
2.77676341635017 5.10371739867902 1.65952620690663 f
-1.47330259248276 -3.32424401503535 -1.58442059837046 cl
-0.84676968583680 -4.73554504386411 1.51402433213741 c
-0.72754840944797 -3.18135519327657 2.84295513412816 h
-2.48398087888630 -5.92363379028668 1.84549575921246 h
0.88992038159433 -5.80407813678197 1.32692305741238 h
$end
E(COSMO) = -1381.6416963833 H
ZPE = 219.9 kJ/mol
enthalpy = 256.25 kJ/mol
chem. pot. = 114.83 kJ/mol

3.7 Optimized structures for fluoride ion affinities

All structures on the RI-B3LYP-D3/def2-TZVPP level of theory, see also section 3.6 for necessary structures.

2,2,4,6-tetrafluoro-1-methyl-1,3,5-triazine

```
$coord
   0.09941750421381    -0.60449281709045     2.36297232064804  c
   0.02435762263131    -4.16830225816490     0.23692919186585  c
   0.21884590083266     0.73463243030198     4.46759673829239  f
   0.05619209441420    -6.66123314462667     0.38401803906994  f
  -0.08431984242552    -3.19447073845026    -1.96502791986116  n
   0.12271720685180    -3.02726954762072     2.52944623001091  n
   0.03205074601634     3.54476162591456     0.22775963559796  c
   1.97522118831202     4.21365691599137     0.32007491704237  h
  -0.83046742779319     4.18402680235571    -1.51898397769682  h
  -1.03328552660262     4.27853873903208     1.81996581252335  h
  -0.04126164114326     0.77131719121715     0.25438086210968  n
  -0.12993643198738    -0.57801794285471    -2.13854746187593  c
   1.84096973813024     0.28784598465962    -3.58205977231524  f
  -2.25050113145046     0.21900675933532    -3.39852461541137  f
$end
```

Etot = -717.8586031889 H
ZPE = 219.3 kJ/mol
enthalpy = 246.05 kJ/mol
chem. pot. = 126.15 kJ/mol

2,4,4,6-tetrafluoro-1-methyl-1,3,5-triazine

```
$coord
  -0.01669286729766    -0.28260756178390     2.19136241349766  c
  -0.01618735684844     1.11507110363620     4.27640712090085  f
   0.02830305885835    -2.66547977648749    -2.29272305487868  n
   0.00430667072822    -2.64569894162950     2.34119262749899  n
   0.00256370755930     3.92304729952177    -0.05416996801002  c
   1.86994911910782     4.59766236762855    -0.59489823003445  h
  -1.38406189394624     4.59411932640813    -1.41085864678694  h
  -0.46909923639553     4.63419905838426     1.80683018001147  h
  -0.04543325353970     1.14744728522205     0.01355721305261  n
   0.00558740594496    -0.30276940555522    -2.15025661386026  c
   0.02913382587844     1.10135876111549    -4.23113697645830  f
   0.00538439931136    -4.04215272625176     0.03088119608341  c
  -2.06548331517242    -5.57234196623794     0.02629946902500  f
   2.05172973581152    -5.60185482397061     0.04751326995867  f
$end
```

Etot = -717.8487796134 H
ZPE = 218.7 kJ/mol
enthalpy = 245.98 kJ/mol
chem. pot. = 121.17 kJ/mol

2-pentafluoro-*ortho*tellurato-4,6-difluoro-1,3,5-triazine

```
$coord
  -4.67545612319515     1.97927920163241     1.89144421688085  c
  -4.10601684817995    -1.61500473853918    -0.08411967577297  c
  -0.85172962920546     0.88145008988750     0.67000448000783  c
  -5.01348803087810    -3.71908062860258    -1.03226976912062  f
  -6.19142682130341     3.60131697420106     2.99742468406693  f
  -1.64865205792291    -1.27725898354057    -0.31765406545004  n
  -2.26849919033808     2.60548602585784     1.81213294221416  n
```

-5.75102088272372	-0.08527044411069	0.98922483291476	n
1.61404707087723	1.49390244929587	0.56921455282830	o
4.32613082157465	-0.38220979770258	-1.00637031608099	te
2.48632385950757	-1.08582998979905	-3.89530178852532	f
5.35685586804224	2.63250259043368	-2.44199235524164	f
6.24610520030036	0.35755892262107	1.81708170254678	f
7.08586431632219	-1.97346870727660	-2.43245179229806	f
3.39096244712250	-3.41337296435818	0.46363235103002	f

$end
Etot = -1320.779486963 H
ZPE = 146.2 kJ/mol
enthalpy = 185.35 kJ/mol
chem. pot. = 33.23 kJ/mol

2-pentafluoro-*ortho*tellurato-4,6-difluoro-1-methyl-1,3,5-triazinium
$coord

-4.56946207327888	0.91711993836128	1.28319975403742	c
-3.91232766776122	-2.88327149255018	-0.29889088420039	c
-0.57087265331146	-0.32879701465585	0.02151027381275	c
-4.81812590808289	-5.04834820885971	-0.94951664544299	f
-6.00510735348624	2.65404854357292	2.23175464646099	f
-1.46811767201342	-2.53880925509059	-0.64795444719221	n
1.78132830937463	0.31867222392737	-0.18281997490268	o
4.70768215874546	-1.65837762230484	-1.61051613754405	te
2.73584112658322	-2.57504754798663	-4.32467438063191	f
5.51045267255917	1.30920915242863	-3.22643258921011	f
6.42243437395101	-0.55702129228723	1.20122011500883	f
7.47665187975642	-3.28389855415577	-2.87805191737451	f
3.67797486318398	-4.47151763705296	0.15263804584213	f
-5.54605579665575	-1.23239465581871	0.65645985217631	n
-1.02442837743798	4.02728080326772	1.76034590426771	c
0.36001834100854	3.73323481805966	3.24838194458982	h
-0.12000001547689	4.85713193133657	0.11473607051544	h
-2.54384837047348	5.22697028558713	2.42254133428006	h
-2.09403783718414	1.53381558422113	1.02606903550763	n

$end
Etot = -1360.403113666 H
ZPE = 252.5 kJ/mol
enthalpy = 296.89 kJ/mol
chem. pot. = 133.39 kJ/mol

2-pentafluoro-*ortho*tellurato-2,4,6-trifluoro-1-methyl-1,3,5-triazine
$coord

-3.89232412115368	0.63685517612704	2.55439978401198	c
-3.43054487890985	-3.24658104277084	1.16018305012961	c
0.06198406153074	-0.72707197855752	0.88995014814392	c
-4.53162159107779	-5.45259259331032	0.79178950229588	f
-5.21245486235852	2.50820193452150	3.54157147844566	f
-1.10825111531064	-3.05293525962036	0.54346720598014	n
0.84443795185634	0.39486045719977	-1.43013514949172	o
3.28822398336337	-0.90716498035418	-3.75071065016725	te
0.83880525042949	-2.70752508792304	-5.49112102610398	f
2.58725644037199	1.91456486822874	-5.70833441613016	f
5.85000886667367	0.87396895177439	-2.13659863438527	f
5.62144232454358	-1.90472806053512	-6.15822319825619	f
4.09063465309027	-3.74933174561406	-1.87579987455911	f
-4.99885593034164	-1.48863224427064	2.16683533717111	n

-0.39472631608074	3.72395147069860	2.53203439442832	c
1.64603936140255	3.53852239791821	2.55448545756986	h
-0.95135980226707	5.02114093593063	1.03688967475709	h
-1.03596917563196	4.43070318267528	4.34848024461269	h
-1.47712759668293	1.20557207668243	2.08253406628927	n
2.20440249655290	-1.01177845880027	2.34830260525809	f

$end
Etot = -1460.532971900 H
ZPE = 256.7 kJ/mol
enthalpy = 303.92 kJ/mol
chem. pot. = 133.83 kJ/mol

2-pentafluoro-*ortho*tellurato-4,4,6-trifluoro-1-methyl-1,3,5-triazine
$coord

-4.36265837330984	1.10882524275640	1.30988760432440	c
-0.39830348663490	-0.17777330533700	0.04094333049916	c
-5.74520799274379	2.95938411066203	2.29156246236806	f
-1.18751620916634	-2.32258804102652	-0.62486626298274	n
2.05433671803935	0.55269125093409	-0.14910389111520	o
4.78588286361931	-1.43812380526678	-1.53597749516991	te
2.97311150331579	-2.42318643637377	-4.34963012323464	f
5.77029110600116	1.46347417856971	-3.23002730890800	f
6.68616501422710	-0.38589486933582	1.20961988537983	f
7.59045574954821	-3.08370347773845	-2.80598695786778	f
3.92328063287077	-4.33043411461911	0.21157458424903	f
-5.38342347065803	-0.94682551082539	0.72623954244394	n
-0.76331986903289	4.19958997534893	1.78126448342626	c
0.63910609447051	3.93574577654268	3.26065815436322	h
0.12794248398841	5.07757446812600	0.15125434854686	h
-2.26694490379657	5.40451347162238	2.47012545606460	h
-1.84952842804537	1.74740251070587	1.05436334654515	n
-3.81030143985329	-2.86604173468383	-0.32482826548718	c
-4.02347307145871	-4.96941677047377	1.14426201959419	f
-4.75989492138088	-3.50521291958762	-2.63133491303926	f

$end
Etot = -1460.527663808 H
ZPE = 256.8 kJ/mol
enthalpy = 304.45 kJ/mol
chem. pot. = 131.17 kJ/mol

2-pentafluoro-*ortho*tellurato-4,6,6-trifluoro-1-methyl-1,3,5-triazine
$coord

-3.62008225489380	-2.94664019329542	-0.39649770081882	c
-0.33527404602955	-0.38140830192265	-0.06139852668420	c
-4.40047015952060	-5.20941844594801	-1.11038209565037	f
-1.11279145018667	-2.59394370056832	-0.75335476372542	n
2.11210994035014	0.30363863654849	-0.26196400408959	o
4.84616804421860	-1.70300738602729	-1.64078321328546	te
3.03212263637014	-2.69379802357159	-4.45242342188696	f
5.83215625450799	1.19066986907983	-3.34172857158215	f
6.73514850724028	-0.64687390767547	1.10759712692665	f
7.64702070203186	-3.36088648036973	-2.89934685578572	f
3.96852320139891	-4.58698348357740	0.11444501308465	f
-5.26821460248858	-1.44060797649053	0.50736616126265	n
-0.77879835088125	3.92465597059836	1.67397395170088	c
0.54537478104419	3.70464128952658	3.23106712035327	h
0.16766629442004	4.82356233472629	0.08728128216159	h

-2.35645394810589	5.08504603679651	2.27498587692799	h
-1.78283503081489	1.45292334564778	0.90856009309035	n
-4.45901918643368	0.93875131359497	1.25591446958681	c
-5.76875354050539	2.78926821014400	-0.00046973950438	f
-5.00359779172189	1.35041089278362	3.75715779791828	f

$end
Etot = -1460.537410725 H
ZPE = 257.1 kJ/mol
enthalpy = 304.39 kJ/mol
chem. pot. = 133.36 kJ/mol

4-pentafluoro-*ortho*tellurato-2,6-difluoro-1-methyl-1,3,5-triazinium
$coord

-2.73703274819351	2.36047391378223	1.54978581601376	c
-2.24290748701017	-1.43098414069654	-0.46130231605289	c
1.08766339189802	1.02371807876100	0.36318138179026	c
-3.29302442874137	-3.45125584059425	-1.35660061112303	f
-4.24590318576293	3.98108841712978	2.59026661284714	f
0.16365642994421	-1.13315622175560	-0.61570783742925	n
-0.35063599904851	2.80574509279770	1.47017474376743	n
3.47964696547216	1.52185402110910	0.29230688401527	o
6.36767098797592	-0.51872954198320	-1.04962474930072	te
4.53917040498972	-1.26017554940257	-3.92210722878598	f
7.42826795856843	2.43900419853782	-2.52175445700072	f
7.99318791681723	0.35822932635882	1.88717071297696	f
9.11190365813959	-2.26866671739830	-2.21512445563021	f
5.08831235421573	-3.35189557609007	0.52807189971092	f
-6.64612905447811	-0.24451415682353	0.69966224586194	c
-6.98538795189659	-1.86138275094667	1.92032188804292	h
-7.57608673090417	1.41918566271141	1.44478759847507	h
-7.30609686736286	-0.63750460416044	-1.20399019693769	h
-3.87627561462269	0.24896638866313	0.60048206875866	n

$end
Etot = -1360.399094183 H
ZPE = 251.6 kJ/mol
enthalpy = 296.50 kJ/mol
chem. pot. = 127.18 kJ/mol

2,4-bis(pentafluoro-*ortho*tellurato)-6-fluoro-1,3,5-triazine
$coord

-2.21574534077028	4.00154182659088	1.15473462609456	c
-1.44290560462994	-0.07476293944734	0.75458456146917	c
1.50046996164028	2.74099986628717	-0.22199022497725	c
-3.77443427210373	5.82912553589123	1.77297014523565	f
0.88778041645448	0.32288980743241	-0.07095071539918	n
0.03641734389340	4.69342704164725	0.36421471826890	n
-3.10086180270072	1.68248274206900	1.40088023072633	n
3.81513141442203	3.41466679944179	-1.03165806680415	o
6.44379676149969	1.23515924273672	-2.33659586376123	te
4.23707527097515	-0.63808796233645	-4.29930370147221	f
6.79341232406312	3.50990103474723	-4.96685445204546	f
8.70386657745636	3.12826556861244	-0.46220573789348	f
9.10089784735049	-0.62185526466590	-3.63028187254072	f
6.20818680730108	-1.04096406569637	0.30551081282706	f
-2.05379220089365	-2.53595889103140	0.92924501551035	o
-5.34017733727891	-3.97907006987162	1.62149745050017	te
-5.92895250030319	-1.95221407239879	4.40576262362860	f

-6.99367012921816 -1.73874263046065 -0.49559130412967 f
-4.79355413922984 -6.09904222452794 -1.10177684756854 f
-8.37282901821936 -5.59776494261044 2.22460000121569 f
-3.71011237970818 -6.27999640240916 3.68320860111542 f
$end
Etot = -2063.456474298 H
ZPE = 183.6 kJ/mol
enthalpy = 243.49 kJ/mol
chem. pot. = 40.52 kJ/mol

2,6-bis(pentafluoro-*ortho*tellurato)-4-fluoro-1-methyl-1,3,5-triazinium
$coord
-2.11120688909353 0.40998623256463 0.58298306238752 c
-1.65414785842453 -3.55335542002975 -0.73824448874202 c
1.82382260696219 -1.17501648236401 -0.63079610462430 c
-2.67871913948862 -5.71744246045439 -1.22673473606612 f
0.80176096951859 -3.37441766598935 -1.13168489478586 n
4.22331764686037 -0.71708319253676 -0.91886225522548 o
6.93889258378010 -2.99860169998180 -2.24659265072609 te
4.85538931646264 -4.01013044840712 -4.84238494910182 f
7.90900713533040 -0.24514593127569 -4.13350214938496 f
8.82861125589802 -1.79708584904587 0.41026854898886 f
9.55715586815558 -4.91543639774458 -3.43373023659983 f
5.79142629426791 -5.58862813887508 -0.23012540096536 f
-3.18194221451385 -1.76706572819163 0.10135734250084 n
1.53218442768192 3.34385156505087 0.83059513995644 c
3.52322640185896 3.31102303686653 0.36948017118916 h
0.56086802493888 4.76403195902447 -0.28934544976253 h
1.26635375309051 3.73316568777668 2.82957662398817 h
0.42836142463029 0.82947484887390 0.24657436445089 n
-3.33555960117937 2.37120879295023 1.42361043230181 o
-7.04556088261159 2.76029736306375 2.07864596713812 te
-7.08674060664125 -0.26853554008609 3.78732653737771 f
-6.05460807244954 4.36931623677500 4.99446253253188 f
-6.72843450166145 5.76123486245716 0.35366215949438 f
-10.39166417344018 3.32343313123833 2.73238263991194 f
-7.77179376993212 1.15092123834071 -0.91892220623327 f
$end
Etot = -2103.089052987 H
ZPE = 290.2 kJ/mol
enthalpy = 355.51 kJ/mol
chem. pot. = 140.95 kJ/mol

2,4-bis(pentafluoro-*ortho*tellurato)-6-fluoro-1-methyl-1,3,5-triazinium
$coord
-2.54636198699958 2.67073524788625 1.21346891851390 c
-1.69102375455081 -1.39698328571487 0.53123197873988 c
1.30869189182677 1.45930700152146 -0.42921048274550 c
0.62174157096148 -0.90126068465108 -0.35262242087118 n
3.50102509146541 2.23112878978048 -1.24169448009055 o
6.42286561846150 0.16037595195479 -2.47124093332089 te
4.28594340318941 -1.74519029805845 -4.44596185777541 f
6.63705715150863 2.51602264606112 -5.01687870995992 f
8.35254250100528 2.22251393114880 -0.44543263535036 f
9.20041779901753 -1.54877110752196 -3.61490667473852 f
6.01471473890585 -2.03375245188230 0.19820888596578 f
-3.32713619691442 0.36924335546594 1.33517275866974 n

0.59920865907123	6.07780344434994	0.23098378446932	c
2.30903257915616	6.27707639376499	1.34944377453451	h
0.98504963429191	6.55614707391269	-1.72827561301346	h
-0.88420381434506	7.27296341539434	0.97765171157957	h
-0.24675431400634	3.40390702925969	0.35385971704412	n
-4.02628050355368	4.47863685711077	1.95706600508966	f
-2.29120461083136	-3.78170181672484	0.56988844609380	o
-5.45751647352566	-5.46400983221950	1.69438034032357	te
-5.52401012992393	-3.49978541034971	4.57441326165233	f
-7.36835921670809	-3.09872097694668	-0.01169487641219	f
-5.20279130957149	-7.32636408953135	-1.22617586778478	f
-8.29752637644130	-7.17425008718673	2.68686942008651	f
-3.37512195148946	-7.72507109682360	3.31145554930017	f

$end
Etot = -2103.085905928 H
ZPE = 290.1 kJ/mol
enthalpy = 355.30 kJ/mol
chem. pot. = 141.56 kJ/mol

2,4,6-tris(pentafluoro-*ortho*tellurato)-1,3,5-triazine
$coord

-1.31808592834816	2.02580011901524	-0.26982976808768	c
-1.09535140188264	-2.15439595782790	-0.26982976808768	c
2.41343733023082	0.12859583881264	-0.26982976808768	c
1.40068789940917	-2.16509464558390	-0.26994750207650	n
1.17468301496870	2.29557862645372	-0.26994750207650	n
-2.57537091437789	-0.13048398086985	-0.26994750207650	n
4.95545865296241	0.11246554958181	-0.28278078393197	o
7.20494652077188	2.97257793626209	0.07608834915157	te
5.32399938295996	4.40244112491405	2.65464625211414	f
8.94899968268505	1.08598887174079	2.44451982900488	f
9.15549403604211	1.53538925414813	-2.44047471312715	f
9.55842124712063	5.52589647124192	0.42734757425732	f
5.56226539024240	4.88951374815563	-2.33956735011367	f
-2.38033130349278	-4.34778585565973	-0.28278078393197	o
-1.02814525285386	-7.72595568802781	0.07608834915157	te
1.15062616136092	-6.81193927783304	2.65464625211414	f
-3.53400589018777	-8.29305549953453	2.44451982900488	f
-3.24806091923115	-8.69658504648344	-2.44047471312715	f
0.00635609921797	-11.04078385570038	0.42734757425732	f
1.45331042293483	-7.26182000461866	-2.33956735011367	f
-2.57512734946962	4.23532030607793	-0.28278078393197	o
-6.17680126791803	4.75337775176570	0.07608834915157	te
-6.47462554432091	2.40949815291897	2.65464625211414	f
-5.41499379249728	7.20706662779372	2.44451982900488	f
-5.90743311681098	7.16119579233534	-2.44047471312715	f
-9.56477734633863	5.51488738445842	0.42734757425732	f
-7.01557581317722	2.37230625646308	-2.33956735011367	f

$end
Etot = -2806.133839636 H
ZPE = 220.9 kJ/mol
enthalpy = 301.65 kJ/mol
chem. pot. = 52.34 kJ/mol

2,4,6-tris(pentafluoro-*ortho*tellurato)-1-methyl-1,3,5-triazinium

$coord

-0.96804724309378	1.91670509975977	-0.30716634504860	c
-0.34278695444735	-2.22635269963025	0.42325583018152	c
3.08126077183033	0.20718540654846	-0.11686501513544	c
2.17284424428752	-2.03761723327296	0.31391155575961	n
5.49732318244533	0.70141828029856	-0.26251855779737	o
8.41971800089358	-1.65483533841611	0.07363565649867	te
6.86479110158741	-3.94809063271772	-2.03168475014787	f
9.69629841323377	-0.00514426093406	-2.70896982672822	f
9.79226000381301	0.80112016454351	2.11745237071080	f
11.23791424297039	-3.64134433872963	0.34528911072377	f
6.97178165752722	-3.13466532789265	2.86954197800880	f
-1.94413608799778	-0.30500851239732	0.12784570120206	n
2.75193938353187	4.80900646195530	-0.96454539226415	c
3.94477876631980	4.65797972714511	-2.62837959349686	h
1.26462014435752	6.17565368759568	-1.27760455301982	h
3.88928377428613	5.34957458255687	0.65772277949114	h
1.58787383674287	2.30675089633321	-0.46274351319295	n
-1.17949826627487	-4.51496329438937	0.85291166502915	o
-4.67628285501513	-5.70921665686907	1.41931202293603	te
-5.29199880337717	-3.02888389750788	3.56901352495938	f
-5.85522327942935	-3.84876316456363	-1.28727033012779	f
-3.89427699676859	-8.31584528275595	-0.74035733622170	f
-7.84801733382018	-6.98164054512818	1.99005839586802	f
-3.37064223051556	-7.52142353998299	4.08108307985590	f
-2.33986108449170	3.94632555743528	-0.62165274737241	o
-6.08759075018302	4.25306439911973	-0.86185023579586	te
-6.19145717701805	1.50046000800982	-2.99056239557971	f
-6.52090228600500	2.20033139496403	1.91301681471992	f
-5.71709552138887	6.99079863460343	1.24710822041983	f
-9.49887157604953	4.74835228303411	-1.14720386547595	f
-5.44599907795112	6.30906814128475	-3.59178424895985	f

$end

Etot = -2845.771664437 H

ZPE = 327.5 kJ/mol

enthalpy = 413.80 kJ/mol

chem. pot. = 148.33 kJ/mol

2,6-bis(pentafluoro-*ortho*tellurato)-2,4-difluoro-1-methyl-1,3,5-triazine

$coord

-1.81448361776664	0.25003148585672	1.34798958416249	c
-1.45095897768500	-3.78676196425010	0.30898796689016	c
2.17975494602599	-1.47249889710187	0.12063163921340	c
-2.64579340463110	-5.95897518380970	0.04288961637247	f
0.92240319066906	-3.76745735884812	-0.10399765065968	n
3.14450985793020	-0.58400708684651	-2.23645408416510	o
5.49381492792151	-2.28518600232578	-4.39043348160000	te
2.94039077291488	-4.13391117730435	-5.92110290974206	f
4.94992300637300	0.32868438024517	-6.66140028586780	f
8.16037930688140	-0.47529189131027	-2.99200768185385	f
7.75505421815188	-3.69289287834774	-6.65802256214510	f
6.14629631962793	-4.92757870661320	-2.19236795615771	f
-2.98766285028518	-1.86734376791576	1.00487134901859	n
1.88533665344012	3.09404227122758	1.44334750385252	c
3.91254091380576	2.83177397464812	1.31638096681929	h
1.26988570148210	4.42157955891269	-0.00057651845538	h

1.40262469615489 3.81886709994123 3.30288118102048 h
0.67750002962685 0.62154205586167 1.05913231242137 n
-3.03873624249620 2.35900388931415 2.07118707124644 o
-6.62186079876086 2.69397834764515 2.77361444185299 te
-6.79701446279076 -0.24220969038102 4.64889993507134 f
-5.68849596875699 4.43297586597571 5.66056296166053 f
-6.46209827242506 5.69732981907353 0.99092375146725 f
-9.97497087669019 3.32610321416449 3.49473596673004 f
-7.58068612639241 1.04489592612553 -0.14643890552967 f
4.22234705767484 -1.72669328393722 1.71576578837706 f
$end
Etot = -2203.211784854 H
ZPE = 294.3 kJ/mol
enthalpy = 362.30 kJ/mol
chem. pot. = 140.48 kJ/mol

2,6-bis(pentafluoro-*ortho*tellurato)-4,4-difluoro-1-methyl-1,3,5-triazine
$coord
-2.04137710077565 0.51952910344977 0.59416264558993 c
1.85842967345090 -1.04422370742784 -0.61439797697270 c
0.95625462576962 -3.19160627347539 -1.11384176180310 n
4.34843339149247 -0.49035839012024 -0.88317129725907 o
6.91807922807624 -2.76336220983192 -2.14503435437131 te
4.99070671017386 -3.88011799455285 -4.82908802791019 f
8.03615887811685 -0.08880797719884 -4.10872536010546 f
8.93967238192655 -1.58835188256085 0.45963464845656 f
9.58919305988807 -4.68409728648862 -3.31015729410561 f
5.92673980289866 -5.42948837161810 -0.12665627183208 f
-3.14524082778040 -1.54445617077416 0.15961155786304 n
1.59362216828367 3.51090368768678 0.83789856143880 c
3.58532822120568 3.48503886326088 0.37600297055974 h
0.64758443231154 4.95318992954915 -0.27817955248153 h
1.35511482918800 3.93435836490163 2.83500285013913 h
0.50556025327365 1.01563737121857 0.26210877701490 n
-3.24772098524317 2.60159298350040 1.48418793863100 o
-6.83563452445352 2.90386508592966 2.10629593929379 te
-7.04758547700067 -0.09554817278956 3.87362371053791 f
-5.97342039644939 4.55208343160007 5.07096345641092 f
-6.66498768379776 5.97688533311444 0.43666995427466 f
-10.20552109672980 3.50470999875893 2.78918033160253 f
-7.74806805619954 1.36776884919521 -0.88991869060140 f
-1.67939321910612 -3.59758708048297 -0.77482909014774 c
-1.98536366337993 -5.59372161631327 0.82236691045212 f
-2.67656462513973 -4.33383586853101 -3.03371057467480 f
$end
Etot = -2203.205993763 H
ZPE = 294.2 kJ/mol
enthalpy = 362.86 kJ/mol
chem. pot. = 134.88 kJ/mol

2,4,6-tris(pentafluoro-*ortho*tellurato)-2-fluoro-1-methyl-1,3,5-triazine
$coord
-1.26910633926403 2.15611715552684 0.14800002970387 c
-0.25322020193765 -1.89158422439448 1.31733017979989 c
2.94956416196951 0.54631708933675 0.12472782919016 c
2.25594824250321 -1.69000074628376 0.82541702007690 n
5.37925971236498 1.08703930884980 -0.39626189781942 o

8.22927408427902	-1.21511558543074	-0.19000122731824	te
6.58765009252544	-3.76191442820536	-1.91765366345328	f
9.26509334438266	0.14207924509422	-3.24121814569799	f
9.93112802652195	1.35904709067003	1.45550255043289	f
11.11781000701659	-3.16632799681178	-0.09909851104641	f
7.28367923357655	-2.51217162067989	2.90735626327829	f
-1.96231334813248	-0.19970854149034	1.07386675489922	n
2.34488835371609	5.04023140453971	-0.98726462485853	c
3.01894935469776	4.93611607685529	-2.92637172386430	h
0.78440751322606	6.36161351895889	-0.87165016439580	h
3.86521108751810	5.66540572008325	0.24330351402365	h
1.42486374577775	2.55589452592876	-0.14901770750284	n
-0.83416121726960	-4.24125314221874	2.15728653133232	o
-4.06562767289900	-5.36558954596335	3.41513761498910	te
-4.58546279290027	-2.49424666355753	5.34359348577037	f
-5.89045219952708	-4.04668155588826	0.74380503806699	f
-3.57623177816542	-8.30696273939556	1.58731758158707	f
-7.03152357768943	-6.69722790454466	4.69621710483111	f
-2.33365641629809	-6.80581633793690	6.09335376123561	f
-2.28492559227276	2.65251377508880	-2.30008712613644	o
-5.77376641630031	2.62717611645251	-3.24205419260371	te
-5.69463719743424	-0.82430840277736	-3.83968004069219	f
-7.00900449015020	2.08759290457134	-0.01328018215147	f
-5.99724841022392	6.09445770843919	-2.73161637247244	f
-9.06104619214638	2.67449954894764	-4.43515699041814	f
-4.65144847880284	3.20705001416279	-6.51122039423122	f
-2.16389463866190	4.02575823207291	1.71941770544511	f

$end
Etot = -2945.890783857 H
ZPE = 331.6 kJ/mol
enthalpy = 420.46 kJ/mol
chem. pot. = 147.36 kJ/mol

2,4,6-tris(pentafluoro-*ortho*tellurato)-4-fluoro-1-methyl-1,3,5-triazine
$coord

-1.21134170470830	1.75586776701127	0.54545134714267	c
-0.70784841534904	-2.50456960251079	1.15334213124220	c
2.77000382710028	0.07075300412907	1.18968181720805	c
1.89253033694872	-2.09350587434149	1.69371035798396	n
5.23614549876057	0.67282932124772	1.49594962395251	o
8.00901841929667	-1.71446865345158	1.63951465748061	te
6.57263491421814	-3.86573024051842	-0.70317449966096	f
9.46231688089875	0.10213006160274	-0.98074779794940	f
9.50229761732714	0.49186081255972	3.90194963542030	f
10.84447211854438	-3.74207148207014	1.75040574033373	f
6.66021774987057	-3.50742488625278	4.30674530110434	f
-2.27804650100840	-0.32721297130377	1.00999985491373	n
2.50117229481052	4.59415105048434	-0.24218315449587	c
4.38686740297410	4.28965907073763	-0.98109522604043	h
1.34940448653201	5.50720225899671	-1.67035580787810	h
2.59986051443871	5.79011458564411	1.42960233139251	h
1.37444813219937	2.12582778648774	0.37228100067317	n
-0.68087996563567	-3.82873484370937	-1.21582160189860	o
-3.53385571934736	-4.98918617090373	-3.07951056918543	te
-5.90484555782290	-4.30013401532632	-0.59052937900147	f
-4.04024567778734	-1.80156055666210	-4.48798624678885	f
-1.29234990205351	-5.73630956144737	-5.66994097713764	f

-6.13446067970900	-6.16318460275912	-5.10957459430644	f
-3.16326205421518	-8.24527917763066	-1.83107328585643	f
-2.48226630726274	3.94317416544222	0.19957217700054	o
-6.10377813900461	4.43133171754641	0.45740986029487	te
-6.92491421107592	1.56891768148321	-1.34554162594572	f
-6.31502515733937	2.72591838429603	3.49422360572070	f
-5.34136701251859	7.37205947824445	2.19528181126290	f
-9.49121845903692	5.23342272483414	0.67861166778052	f
-5.87950167147044	6.22544741489335	-2.53645767631581	f
-1.67618305857480	-4.08129464675337	2.93025952155375	f

$end
Etot = -2945.882352172 H
ZPE = 331.4 kJ/mol
enthalpy = 420.48 kJ/mol
chem. pot. = 147.07 kJ/mol

$[Me_3Si]^+$
$coord

0.00000000000000	0.00000000000000	-0.02110420009278	si
-0.04206233671061	3.45607112267237	0.00752217593567	c
0.80952485759156	4.12139753112963	1.77643787803367	h
1.14927836938218	4.19455039676384	-1.51371530319622	h
-1.93841292139148	4.23788897881015	-0.15634288320100	h
-2.97201422116478	-1.76446261347011	0.00752217593567	c
-4.20722638574267	-1.10197093447700	-1.51371530319622	h
-2.70091305337194	-3.79765932235410	-0.15634288320100	h
-3.97399738984850	-1.35962967389554	1.77643787803367	h
3.01407655787538	-1.69160850920227	0.00752217593567	c
3.16447253225694	-2.76176785723408	1.77643787803367	h
3.05794801636049	-3.09257946228684	-1.51371530319622	h
4.63932597476342	-0.44022965645604	-0.15634288320100	h

$end
Etot = -408.9218468601 H
ZPE = 282.1 kJ/mol
enthalpy = 305.50 kJ/mol
chem. pot. = 197.92 kJ/mol

Me_3SiF
$coord

0.81615731116996	0.00005535589743	0.00018112347328	si
-0.22554087300660	-2.32641252776354	-2.43937166170793	c
0.47312058200833	-4.21848886017854	-2.00586074877708	h
-2.28562981361037	-2.42002904196625	-2.53732812103838	h
0.47285947901099	-1.80340087207381	-4.30879538846769	h
-0.22539806884423	3.27599394730790	-0.79520332053894	c
-2.28547068457563	3.40762860003922	-0.82809495024528	h
0.47253678124699	4.63377951493220	0.59235579374510	h
0.47395180177276	3.84609617955257	-2.65044905985939	h
-0.22529174018130	-0.94957623231181	3.23478178890625	c
0.47328962399648	-2.82997175916255	3.71652725015561	h
0.47319321556270	0.37201025908984	4.65657749008150	h
-2.28537838303647	-0.98776148856422	3.36473596060196	h
3.87760076848636	0.00007692520159	-0.00005615632901	f

$end
Etot = -509.1365793346 H
ZPE = 293.7 kJ/mol
enthalpy = 318.22 kJ/mol
chem. pot. = 210.19 kJ/mol

(MeO)^{ax}PF$_4$

$coord

-0.45980711572844	1.13516379858483	-1.80522583781724	p
-1.47816008628210	-1.55919371864389	-2.50120172208298	f
-0.25003620459523	1.73593744545735	-4.76679644730418	f
2.48929570006702	1.37644139194551	-1.62965309243785	f
-2.24842004340770	3.46276997384458	-1.56404032614548	f
-0.78585290847361	0.68774423485157	1.19753907243274	o
0.46057089129951	-1.33570380524817	2.49559241308910	c
2.50845183859560	-1.13054292496871	2.38656640272638	h
-0.13409439948970	-1.20763731470978	4.45724965829672	h
-0.10194767198530	-3.16497908111330	1.72996987924285	h

$end

Etot = -856.0114791769 H

ZPE = 147.1 kJ/mol

enthalpy = 169.36 kJ/mol

chem. pot. = 61.87 kJ/mol

(MeO)^{eq}PF$_4$

$coord

0.08717980431942	-2.13514295925814	0.48854060777968	p
-0.91825324262419	-2.99066432626798	3.19553446951168	f
-1.87235691932052	-3.93061564820832	-0.78677129832099	f
1.14261056953783	-1.43355251627652	-2.29194371757110	f
2.72847656193472	-3.27839971813242	1.12569649643098	f
-0.58406126093453	0.68254437542104	1.15230661438440	o
-0.20379276288664	2.86057388848583	-0.45174073589904	c
-1.28615216126866	2.67742159751378	-2.18796675926422	h
-0.88270499480480	4.45415834856472	0.64875056635384	h
1.78905440604738	3.09367695815813	-0.89240624340513	h

$end

Etot = -856.0238831319 H

ZPE = 148.1 kJ/mol

enthalpy = 169.93 kJ/mol

chem. pot. = 63.90 kJ/mol

4 References

[1] S. L. Bell, R. D. Chambers, W.K.R. Musgrave, J. G. Thorpe, *J. Fluor. Chem.* **1971**, *1*, 51.
[2] H. Böhrer, N. Trapp, D. Himmel, M. Schleep, I. Krossing, *Dalton Trans.* **2015**, *44*, 7489.
[3] S. Hämmerling, G. Thiele, S. Steinhauer, H. Beckers, C. Müller, S. Riedel, *Angew. Chem. Int. Ed.* **2019**, *58*, 9807; *Angew. Chem.*, **2019**, *131*, 9912.
[4] A. Guha, R. Pal, *J. Indian Chem. Soc.* **2006**, *83*, 49.
[5] L. Pogliani, *J. Comput. Chem.* **2010**, *31*, 295.
